# Enantioselective photoredox dehalogenative protonation†
†Electronic supplementary information (ESI) available. CCDC 1590512 and 1840318. For ESI and crystallographic data in CIF or other electronic format see DOI: 10.1039/c9sc02000d


**DOI:** 10.1039/c9sc02000d

**Published:** 2019-06-07

**Authors:** Meimei Hou, Lu Lin, Xiangpei Chai, Xiaowei Zhao, Baokun Qiao, Zhiyong Jiang

**Affiliations:** a Henan University , Jinming Campus , Kaifeng , Henan 475004 , China . Email: jiangzhiyong@htu.edu.cn ; Email: chmjzy@henu.edu.cn; b Henan Key Laboratory of Organic Functional Molecules and Drug Innovation , Key Laboratory of Green Chemical Media and Reactions , Ministry of Education , Collaborative Innovation Center of Henan Province for Green Manufacturing of Fine Chemicals , School of Chemistry and Chemical Engineering , Henan Normal University , Xinxiang , Henan 453007 , China

## Abstract

We report an enantioselective photoredox dehalogenative protonation as a new type of asymmetric protonation. As a paradigm, with a cooperative catalytic system consisting of a chiral H-bonding catalyst and a dicyanopyrazine-derived chromophore (DPZ) photosensitizer that is irradiated with visible light, a range of cyclic and acyclic ketones with labile chiral secondary C–F, C–Cl and C–Br bonds at the α-position were obtained in high yields with good to excellent enantioselectivities (up to >99% ee) by using a secondary amine as the terminal reductant. Given the ready accessibility of halides, the success of this work should provide inspiration for constructing diverse chiral α-tertiary carbonyls and their variants.

## Abstract 2

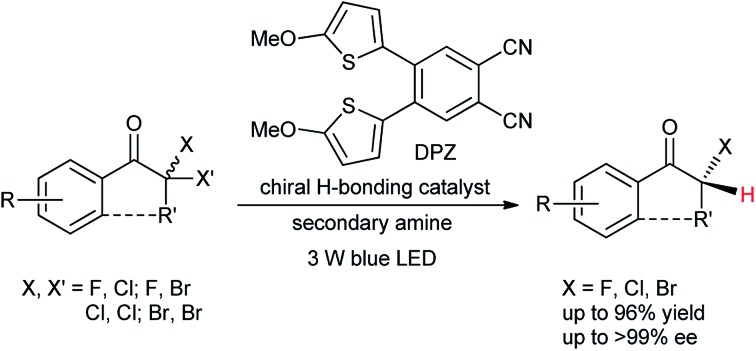
We report an enantioselective photoredox dehalogenative protonation as a new type of asymmetric protonation.

## Introduction

**Scheme 1:**
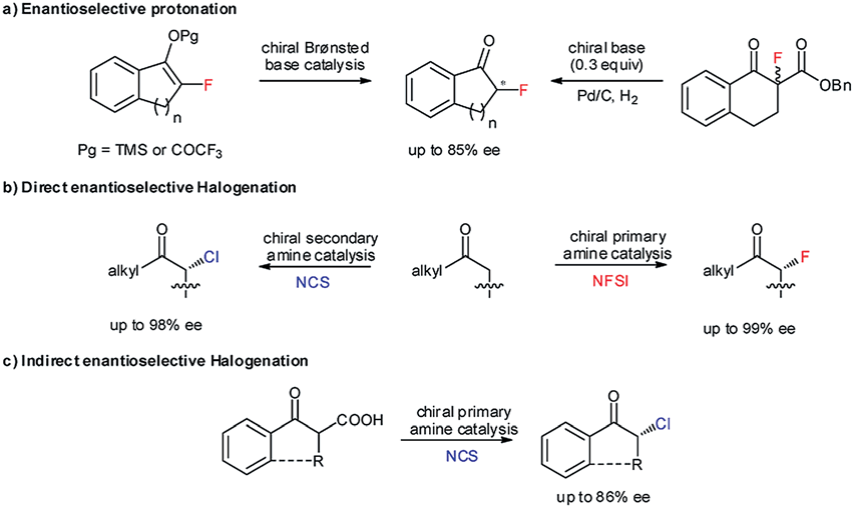
Previous studies on the enantioselective construction of secondary α-C–X bonds for ketones.

Enantioselective protonation is a fundamental method for synthesizing enantioenriched α-tertiary carbonyl compounds.^1^ In the past decade, a number of efficient ground-state catalytic platforms have been established, allowing the precise delivery of a proton to prochiral carbanion intermediates in a highly enantioselective manner.^2^–^8^ Nonetheless, the approach of constructing secondary α-carbon–halogen bonds for ketones still remains underdeveloped, as few examples with insufficient enantioselectivities have been described (Scheme 1a).3b,4a,6a The stereoselective formation of C–F bonds is of critical importance in pharmaceutical chemistry given the ability of fluorine atoms to act as the isostere of hydrogen atoms in decreasing the rate of metabolic degradation without influencing the pharmacological effects.^9^ Meanwhile, chiral C–Cl and C–Br bonds are applicable for introducing various important molecular architectures *via* simple and stereospecific transformations and are thus extensively adopted in the asymmetric synthesis of chiral complex molecules with significant biological activities.9a,b,^10^ In this context, the development of a new and generic catalytic asymmetric protonation approach to form valuable chiral tertiary α-haloketones^9^,^10^ represents a highly desirable task that would be a complementary and more versatile method than direct catalytic enantioselective halogenation. Of note, a few direct asymmetric α-chlorination and α-fluorination reactions of aliphatic ketones *via* enamine catalysis have been established (Scheme 1b);^11^ however, the catalytic systems are not compatible with aromatic variants, likely because of the slow enamine formation and the approximately equimolar enamine rotational isomers. An approach through indirect enantioselective chlorination of β-ketocarboxylic acids^12^ also failed to generate chiral α-secondary chloro-ketones in high enantioselectivities (Scheme 1c).

**Scheme 2:**
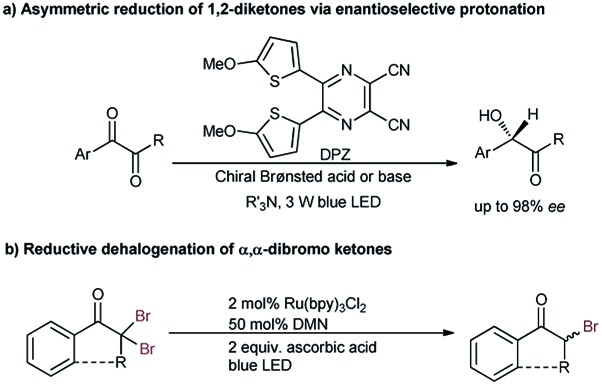
Previous studies. (a) Photoredox catalytic asymmetric reduction of 1,2-diketones *via* enantioselective protonation to build the labile α-secondary alcohols of ketones. (b) Photoredox catalytic reductive dehalogenation of α,α-dibromoketones in a racemic manner.

Due to the strong electron-withdrawing properties of ketones and the high electronegativity of halogens, the stereocenter of secondary α-haloketones should be particularly labile, especially for those aromatic variants.6a,11b As such, a mild and neutral protonation strategy to avoid racemization of the products in the reaction process is inarguably crucial. Recently, we reported the asymmetric photoreduction of 1,2-diketones to chiral α-hydroxy ketones enabled by a synergistic photosensitizer and H-bonding catalysis with weakly basic tertiary amines as the terminal reductant (Scheme 2a).^13^ The excellent enantioselectivity clearly demonstrated the applicability of such an open-shell single-electron-transfer (SET) approach^14^ for the formation of the labile tertiary carbon stereocenter of α-hydroxyl ketones *via* an asymmetric protonation procedure. Accordingly, we wondered whether the ability to combine this dual-catalysis^15^ reduction system with competent oxidative feedstocks, from which halogenated carbon anions α to ketones could be produced *via* two SET reductions, might address this challenging task.

Alkyl halides (X) are an important class of readily accessible starting substrates in organic synthesis.^16^ Since Tanaka and co-workers introduced the visible light-driven reductive dehalogenation of benzyl bromide to benzene in 1984,^17^ the groups of Kellogg,18*a* Fukuzumi,18*b* Stephenson,18c–e Zeitler,18*f* and Hisaeda18*g* and so on18h–o have accomplished the transformation of diverse alkyl halides to alkanes *via* photoredox catalysis with tertiary amines as the reductant. This conceptual strategy was developed by the Reiser group^19^ by establishing an alternative reductive system, *i.e.*, 1,5-dimethoxynaphthalene (DMN) with ascorbic acid, wherein the selective debrominative protonation of α,α-dibromoketones to the racemic monobromo ketones was described (Scheme 2b). Furthermore, Hyster and co-workers recently reported an enzyme catalytic debromination-enantioselective hydrogen atom transfer (HAT) to access enantioenriched α-tertiary esters from α-bromoesters.^20^ Encouraged by these elegant studies, we hypothesized that the enantioselective platform of α,α-dihaloketones would be an expedient and modular strategy for accessing the desired chiral organohalides. Several significant challenges should remain in this promising task, namely the weaker basicity of ketones relative to ketyls which would impair the stereocontrol of H-bonding catalysis, and both the competitive racemic background reaction and the possible racemization of the stereocenter which should diminish the enantioselectivity. More importantly, even employing a powerful enzyme catalysis and capturing a hydrogen atom from the bulky flavin still could not provide a good enantiofacial result for the formation of a C–F bond.20*a* Therefore, the direct delivery of the smallest proton to the prochiral intermediates through the distinctly weaker asymmetric H-bonding induction to provide stereocontrolling environments would be more formidable.

## Results and discussion

### Optimization of reaction conditions

We began our study with 2-chloro-2-fluoro-tetralone **1a** as the model substrate (Table 1). A range of chiral H-bonding catalysts, photoredox catalysts, amine reductants, and inorganic bases as the acid-binding agents to remove the generated HCl were examined in conjunction with diverse reaction parameters (see Tables S1 and S2 in the ESI†). As a result, the desired product **2a** was obtained in 92% yield with 96% ee when the reaction was performed in 1,2-dichloroethane at 5 °C for 36 h in the presence of dicyanopyrazine-derived chromophore (DPZ, *E*^t^(S*/S˙^–^) = +0.91 V *vs.* SCE, *E*red1/2 = –1.45 V *vs.* SCE in CH_2_Cl_2_, *E*_T_ = 46.4 kcal mol^–1^),^13^,16b,^21^ 5 mol% l-*tert*-leucine-based squaramide-tertiary amine **C1**,^22^ 0.6 equiv. **Amine-1** as the secondary amine, 1.0 equiv. H_2_O and 2.5 equiv. Na_2_CO_3_ (entry 1). The structural features of the chiral catalyst, consisting of the aryl substituent of squaramide (**C2**), the structural skeleton of the tertiary amine (**C3**) and the type of H-bonding donor (**C4** and **C5**), were modified to affect the enantiofacial selectivity (entries 2–5). When **Amine-2** was the reductant, the yield of **2a** decreased to 76% with a similar enantioselectivity (entry 6). Other plausible photoredox catalysts, including Ru(bpy)_3_Cl_2_·6H_2_O (
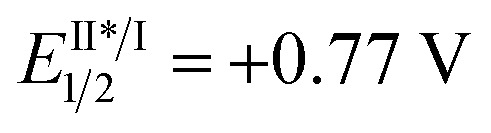
 *vs.* SCE, *E*II/I1/2 = –1.33 V *vs.* SCE in CH_3_CN, *E*_T_ = 46.5 kcal mol^–1^), Rose Bengal (*E*^t^(S*/S˙^–^) = +0.99 V *vs.* SCE, *E*red1/2 = –0.68 V *vs.* SCE in CH_3_CN, *E*_T_ = 40.9 kcal mol^–1^) and Ir(ppy)_2_(dtbbpy)PF_6_ (
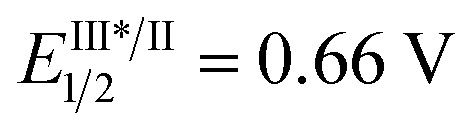
 *vs.* SCE, *E*III/II1/2 = –1.51 V *vs.* SCE in CH_3_CN, *E*_T_ = 49.2 kcal mol^–1^), were tested (entries 7–9), but better comprehensive results were not achieved. The moderate yield obtained with Rose Bengal stemmed from an unclear reaction interruption, which resulted in unsatisfactory chemical conversion. The transformation in the absence of catalyst **C1** offered *rac*-**2a** in 48% yield with >95% chemical conversion after 36 h, indicating that a racemic background reaction occurred (entry 10). The lower yield was due to deterioration of the chemical selectivity, as a few unknown side products were observed. In the absence of H_2_O, the yield of **2a** decreased to 74% with a similar enantioselectivity (entry 11), suggesting its important effect in increasing the solubility of inorganic salts. DPZ as the photoredox catalyst was found to accelerate the transformation (entry 12), although the type of EDA^23^ is involved in the process (see ESI† for details). Control experiments also confirmed that reductants, inorganic bases and visible light were essential for the reaction (entries 13–15).

**Table 1:** Optimization of reaction conditions^*a*^

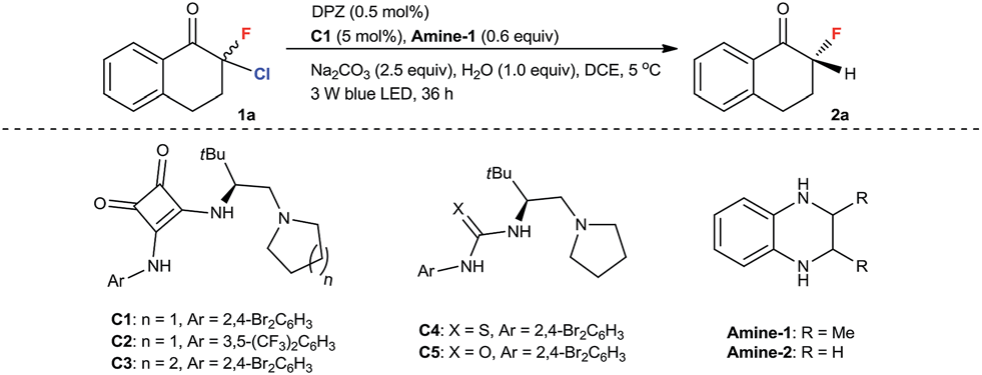			
Entry	Variation from the standard conditions	Yield^*b*^ (%)	ee^*c*^ (%)
1	None	92	96
2	**C2** instead of **C1**	87	81
3	**C3** instead of **C1**	64^*d*^	23
4	**C4** instead of **C1**	89	77
5	**C5** instead of **C1**	87	65
6	**Amine-2** instead of **Amine-1**	76	94
7	Ru(bpy)_3_Cl_2_·6H_2_O instead of DPZ	87	95
8	Rose Bengal instead of DPZ	67	96
9	Ir(ppy)_2_(dtbbpy)PF_6_ instead of DPZ	82	91
10	No **C1**	48^*d*^	N.A.
11	No H_2_O	74	95
12	No DPZ	38^*e*^	94
13	No **Amine-1**	0	N.A.
14	No Na_2_CO_3_	0	N.A.
15	No light	0	N.A.

^*a*^The reaction was performed on a 0.05 mmol scale. Entries 1–9, the chemical conversion of **1a** was >95% determined by crude ^1^H NMR.

^*b*^Yields were determined from the isolated compound following chromatographic purification.

^*c*^Enantiomeric excesses were determined by HPLC analysis on a chiral stationary phase.

^*d*^The chemical conversion of **1a** was >95%, as determined by crude ^1^H NMR.

^*e*^The chemical conversion of **1a** was 45%.

With the optimized conditions in hand, we next evaluated the substrate scope (Table 2). A range of 2-chloro-2-fluoro-tetralones with diverse electron-withdrawing and electron-donating substituents on the aryl ring were first examined. The reactions were finished within 24–36 h, and a series of chiral α-fluorinated tetralones **2a–o** were obtained in 68–96% yield and with 92 to >99% ee. The satisfactory results achieved with alkyne (**2h**), hydroxyls protected by tosyl (Ts, **2j**), allyl (**2k**), mesyl (Ms, **2m**) and *tert*-butyl-dimethylsilyl (TBS, **2n**) groups and even the naked hydroxyl (**2o**) as the substituent underscore the high functional-group tolerance of this catalytic system. The reaction to furnish **2a** was attempted on a 1.0 mmol scale, and a similar yield and enantioselectivity were observed with a slightly longer reaction time (48 h, see footnote *b*), indicating the promising synthetic utility of this method. Other significant cyclic ketones, including 4-chromanone (**2p**), thiochroman-4-one (**2q**), 1-indanones with distinct substituents on the aromatic ring (**2r–2x**) and 1-benzosuberone (**2y**), were subsequently tested, leading to a variety of corresponding enantioenriched α-fluoroketones in 55 to 90% yield and 84 to 93% ee. Linear and nonaromatic ketones were also evaluated. To achieve the best enantioselectivity, catalyst **C25** was used for the preparation of **2za** and **2zc–e** (82–90% ee), and **C26** was used for **2zb** (90% ee) (see ESI† for the structures of tertiary-squaramide-based **C25** and **C26**). The generality of this method was thus illustrated. We also carried out enantioselective reductive debromination of 2-bromo-2-fluoro-tetralone **3**; **2a** was obtained with a similar excellent enantioselectivity but in a lower yield than that achieved *via* the dechlorination of **1a**. Note that these α-fluoroketones are stable under the reaction conditions, as indicated by the ee value being maintained and no defluorinated parent ketone being observed when the reaction was performed for a longer time. Moreover, the determination of 0% ee of **2a** before the reaction completed excluded the possibility of kinetic resolution of **2a**.1
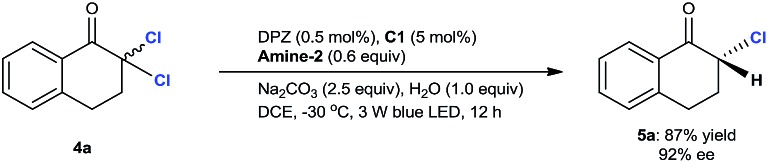



**Table 2:** Dehalogenative protonation for the synthesis of chiral secondary α-fluoroketones^*a*^

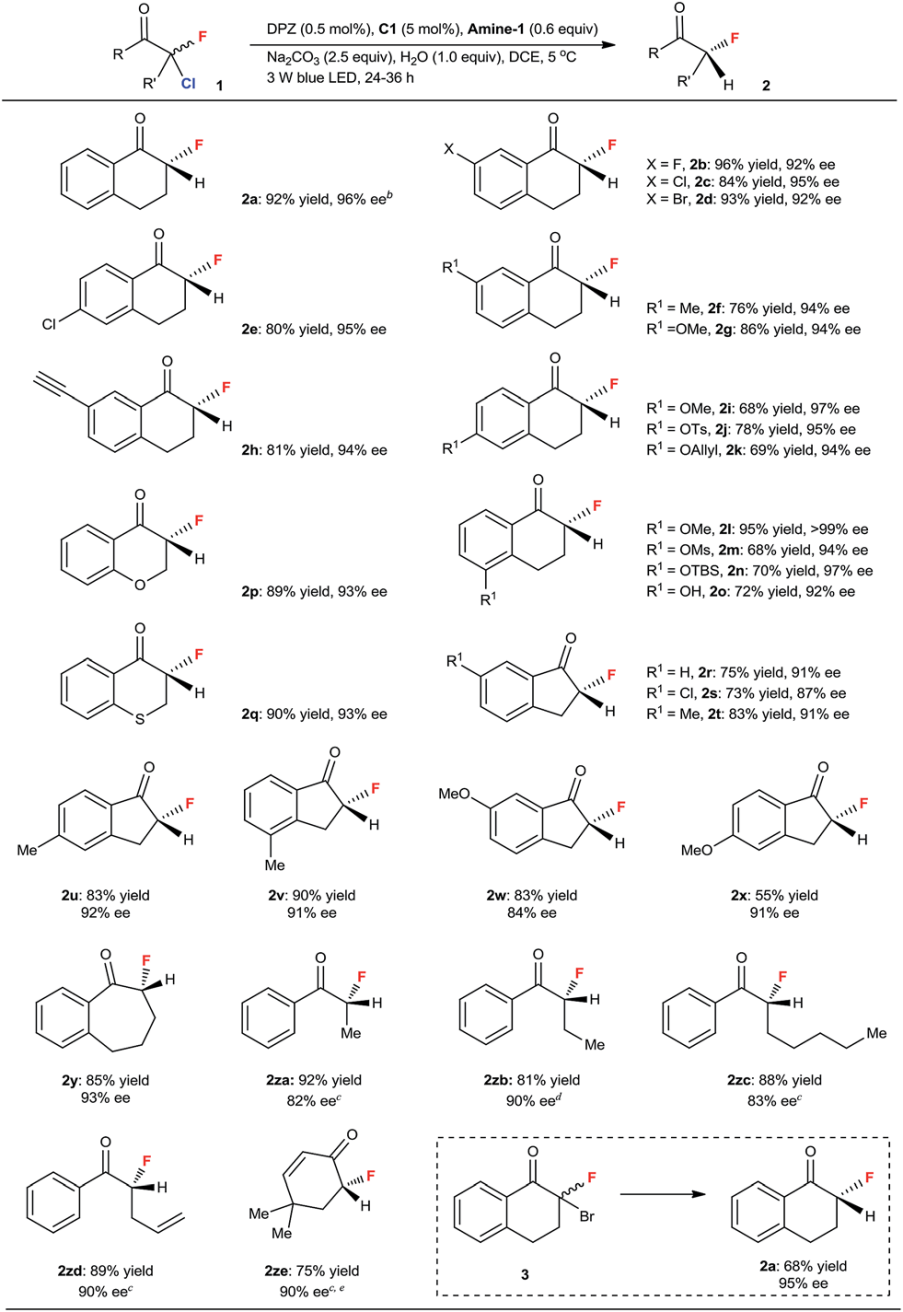

^*a*^Reaction performed on a 0.10 mmol scale. Yields were determined from the isolated material after chromatographic purification. Enantiomeric excesses were determined by HPLC analysis on a chiral stationary phase.

^*b*^When the reaction was performed on a 1.0 mmol scale, *t* = 48 h, 86% yield, 97% ee.

^*c*^Catalyst **C25** was used instead of **C1**.

^*d*^Catalyst **C26** was used instead of **C1**.

^*e*^The modified reaction parameters: **Amine-2** instead of **Amine-1**, no H_2_O in THF at 40 °C for 60 h.

The aforementioned success encouraged us to further evaluate this protocol in constructing chiral tertiary α-chloroketones from α,α-dichloroketones. The preliminary studies with 2,2-dichloro-tetralone **4a** as the starting substrate showed that the slightly modified reaction conditions, wherein the reductant was **Amine-2** and the temperature was –30 °C, afforded chiral 2-chloro-tetralone **5a** in 87% yield and with 92% ee within 12 h (eqn (1)).^25^ However, the ee value of this product was observed to deteriorate when flash column chromatography was performed, revealing that such a tertiary stereocenter is considerably labile.

Accordingly, we planned to explore a sequential strategy involving subsequent one-pot reduction of ketone to chlorohydrin, thus facilitating purification. After careful examination, we found that the desired product **6a** was obtained in 79% yield over two steps with a maintained ee of 92% and >20 : 1 dr when the reaction mixture from the first reductive dechlorination step was directly treated with 3.0 equiv. of diisobutylaluminum hydride (DIBAL-H) at –40 °C under an argon atmosphere for 3 h (Table 3). In the presence of the established tandem protocol, a series of 2,2-dichloro-tetralones with different substituents on the aryl ring were examined, leading to the important chlorohydrins^24^**6b–k** in 60–82% yield with 84–94% ee and >20 : 1 dr. With respect to 2,2-dichloro-1-indanone, the reaction conditions were further modified for achieving higher enantioselectivity (see footnote *c*). As a result, product **6l** was obtained in 65% yield, 87% ee and >20 : 1 dr. Inarguably, these results demonstrated that a secondary C–Cl bond was successfully introduced at the α-position of these cyclic ketones with high enantioselectivities, indicating the compatibility of this catalytic system even though these α-secondary C–Cl bonds of ketones are unstable. The attempt with linear ketones showed that **C25** was a suitable H-bonding catalyst, leading to a series of α-chloroketones **5m–o** in 73–83% yield with 80–82% ee. Because the products were stable during flash column chromatography, the further reduction of ketone to alcohol was unnecessary. Notably, some amount of doubly dechlorinated products was found before the reactions were finished. Hence, we tested the ee of **5a** at different reaction stages and after prolonging the reaction beyond completion and found that the amount of tetralone continued to increase. The results demonstrated that all the ee values were consistent, indicating that kinetic resolution is not possible in the dechlorination of monochlorinated ketones.2
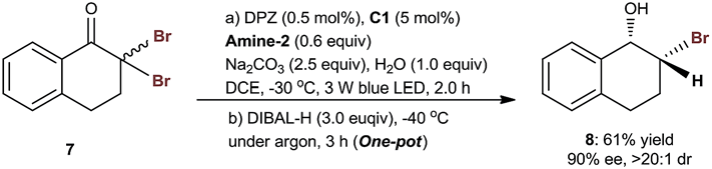



**Table 3:** Dechlorinative protonation for the synthesis of chiral secondary α-chlorohydrins or α-chloroketones^*a*^

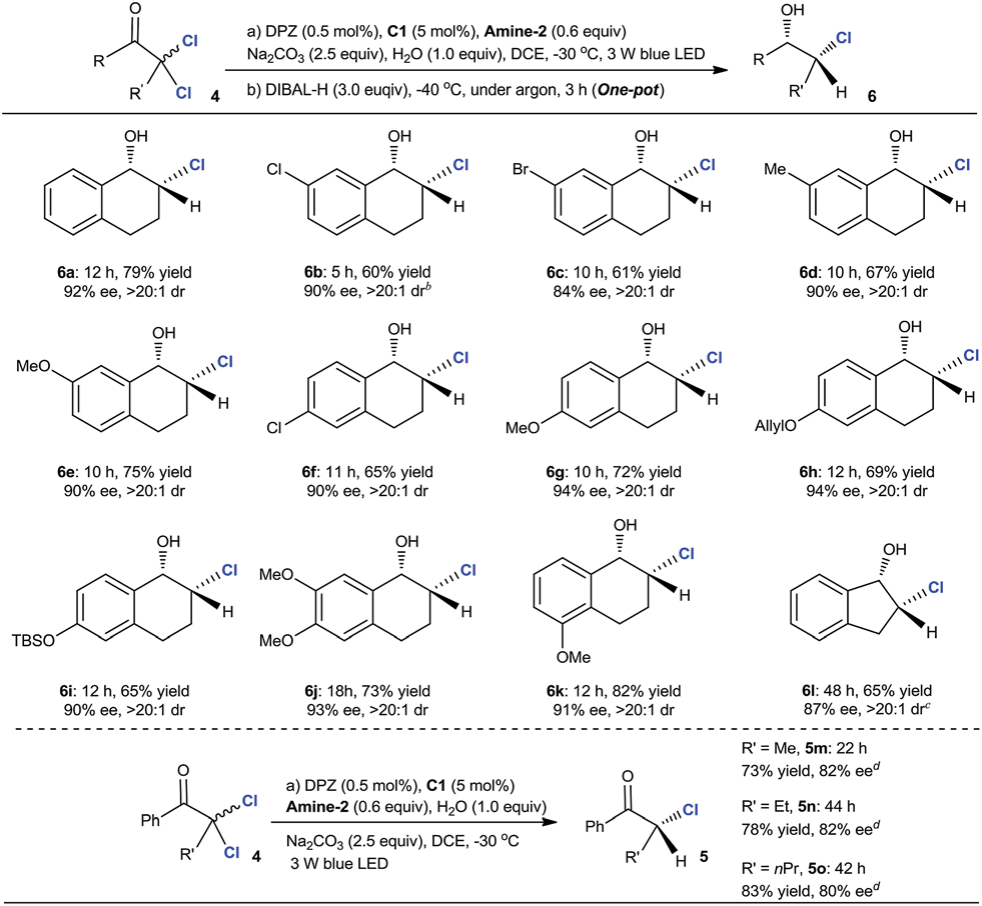

^*a*^Reaction performed on a 0.10 mmol scale. The reaction time stated below the product structure refers to the reductive dechlorination process. Yields were determined from the isolated material after chromatographic purification. Enantiomeric excesses were determined by HPLC analysis on a chiral stationary phase. The dr was determined by the crude ^1^H NMR analysis.

^*b*^2.0 mL DCE was used.

^*c*^The modified reaction parameters: 20 mol% **C1**, 1.5 equiv. Na_2_CO_3_ in DCM at –60 °C.

^*d*^Catalyst **C25** was used instead of **C1**.

Given the almost identical utility of chiral C–Cl and C–Br bonds in organic synthesis, 2,2-dibromo-tetralone **7** was selected as a representative to evaluate the viability of enantioselective photoredox debrominative protonation. The investigation revealed that the chiral α-bromo-tetralone stereocenter exhibited lability similar to that of the chloro variant. Accordingly, the cascade reaction was performed, and chiral bromohydrin **8** was successfully achieved in 61% yield, 90% ee and >20 : 1 dr (eqn (2)).

### Mechanism studies

**Scheme 3:**
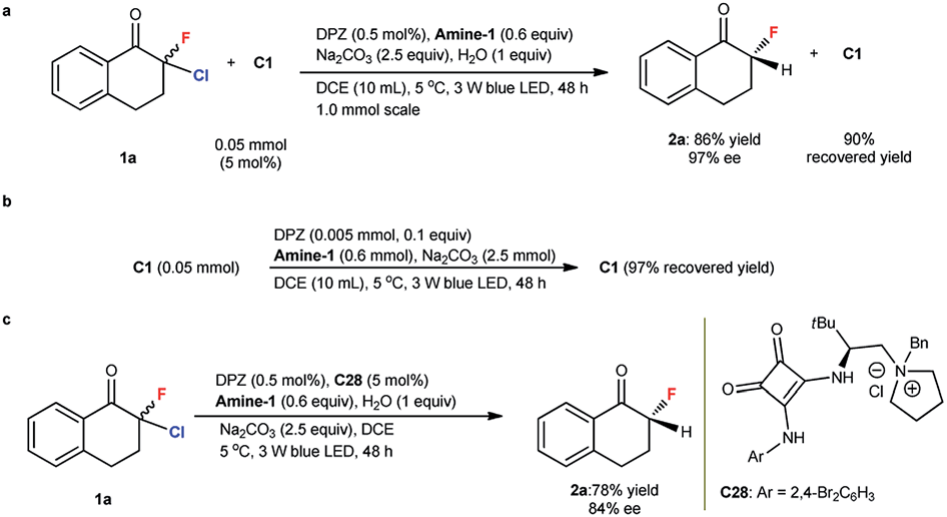
^a^Determining the structure of **C1** after the reaction completed. ^b^Determining the structure of **C1** when without the starting substrate **1a**. ^c^The reaction of **1a** to **2a** was performed under standard reaction conditions but with **C28** instead of **C1** as the chiral catalyst.

The mechanism of these reactions *via* a photoredox dehalogenation–protonation process is established.^18^,^19^ Our Stern–Volmer experiments demonstrated the occurrence of the same catalytic cycle in which the transformation was triggered by reductive quenching of the photoredox catalyst (see the ESI†). Notably, all chiral catalysts (**C1**, **C25** and **C26**) contain a squaramide group, and this diketone moiety (*e.g.*, *E*red1/2 of **C1** = –0.22, –0.79 V *vs.* SCE in CH_3_CN) should be reduced by DPZ˙^–^ more readily than α,α-dihaloketones (*e.g.*, *E*red1/2 of **1a** = –0.80, –1.23 V *vs.* SCE in CH_3_CN). In this context, the structure of **C1** after the transformation of **1a** to **2a** was analyzed. As shown in Scheme 3a, **C1** was recovered in 90% yield and no reduced product was observed. In the absence of **1a**, a high recovery yield of **C1** was still obtained (Scheme 3b). These results suggest a perfect tolerance of **C1** under the reaction conditions, likely because of its evidently lower concentration than the substrates in the reaction system. For the formation of the α-secondary stereocenter, the use of structurally different **Amine-1** and **Amine-2** as the reductants offering similar enantioselectivities (entries 1 and 6, Table 1) could roughly prove a protonation process rather than HAT. The deuterium labelling studies could further demonstrate this protonation process (see ESI†).

To probe the role of **C1**'s bifunctional groups, *i.e.*, tertiary amine and squaramide, the transformation of **1a** was performed under the standard reaction conditions, as shown in Table 1, but by using **C1**-derived quaternary ammonium salt **C28** as the catalyst. Product **2a** was obtained in 78% yield with 84% ee (Scheme 3c). The negligible change in enantioselectivity and the maintained absolute configuration suggest that the tertiary amine moiety of **C1** might not participate in proton transfer but instead provides a steric factor only for establishing stereocontrol. From the similar enantioselective results in the construction of different C–X bonds with the same chiral catalyst **C1** (see the results for **2a**, **5a** and **8** in Table 2, eqn (1) and (2), respectively), the hydrogen-bonding interaction between the squaramide of **C1** and halogen of ketones might be excluded. Meanwhile, the remarkably different enantioselectivity with **C1** and **C3** catalysts (entries 1 and 3, Table 1) indicates a slight possibility of the strongly acidic squaramide participating in proton transfer interchange to present H^+^ to carbanion intermediates in the protonation step. Accordingly, a plausible transition state involving H-bonding interactions between the squaramide of **C1** and ketone for facilitating stereocontrol and improving the chemoselectivity was proposed and is shown in the ESI.†

## Conclusions

In summary, we have developed a novel asymmetric protonation strategy that is the visible-light-driven photoredox-catalyzed dehalogenation–enantioselective protonation. Through the establishment of a dual organocatalytic system with a secondary amine reductant, a range of important cyclic and acyclic ketones with labile chiral secondary C–F, C–Cl and C–Br bonds at the α-position were collectively obtained in high yields and with high ee values. Although the stereocenters of these products are liable to proceed racemization in the reaction process and a competitive racemic background reaction exists in the reaction system, the satisfactory results suggest the amazing feasibility and compatibility of this strategy. Given the convenient preparation of alkyl halides, we expect this method to be extensively utilized in the construction of diverse significant and, especially, complex chiral α-tertiary carbonyls and their variants, thus leveraging the broad applicability of enantioselective protonation in organic synthesis.

## Conflicts of interest

There are no conflicts to declare.

## Supplementary Material

Supplementary information

Crystal structure data
