# Supplementary material for: Enantioselective photoredox dehalogenative protonation
Source: Chem Sci. 2019 Jun 7;10(27):6629–34. doi: 10.1039/c9sc02000d (PMC6625487; doi:10.1039/c9sc02000d)

Electronic Supplementary Material (ESI) for Chemical Science.
This journal is © The Royal Society of Chemistry 2019

Supporting Information

Enantioselective Photoredox Dehalogenative Protonation

Meimei Hou,[a] Lu Lin,[a] Xiangpei Chai,[a] Xiaowei Zhao,[a] Baokun Qiao,*,[a] and Zhiyong Jiang*,[a,b]

[a]*Henan University, Jinming Campus, Kaifeng, Henan, 475004, P. R. China*

[b]*Henan Key Laboratory of Organic Functional Molecules and Drug Innovation, Key Laboratory of Green Chemical Media and Reactions, Ministry of Education, Collaborative Innovation Center of Henan Province for Green Manufacturing of Fine Chemicals, School of Chemistry and Chemical Engineering, Henan Normal University, Xinxiang, Henan 453007, China*

Table of Contents

1. **General information** S3

2. **Optimization of reaction conditions** S5

3. **General experimental procedures** S9

4. **Mechanism studies** S13

5. **Proposed mechanism.** S32

6. **Determination of the absolute configurations** S33

7. **References** S43

8. **Characterization of products** S44

9. **Crude ^{1}H NMR spectra to determine dr** S90

10. **Copies of NMR spectra** S97

1. General information

General procedures and methods

Experiments involving moisture and/or air sensitive components were performed under a positive pressure of argon in oven-dried glassware equipped with a rubber septum inlet. Dried solvents and liquid reagents were transferred by oven-dried syringes or hypodermic syringe cooled to ambient temperature in a desiccator. Reactions mixtures were stirred in 10 mL sample vial with Teflon-coated magnetic stirring bars unless otherwise stated. Moisture in non-volatile reagents/compounds was removed in high *vacuo* by means of an oil pump and subsequent purging with nitrogen. Solvents were removed *in vacuo* under ~30 mmHg and heated with a water bath at 30–35 °C using rotary evaporator with aspirator. The condenser was cooled with running water at 0 °C.

All experiments were monitored by analytical thin layer chromatography (TLC). TLC was performed on pre-coated plates, 60 F_{254}. After elution, plate was visualized under UV illumination at 254 nm and 365 nm for UV active material. Further visualization was achieved by staining phosphomolybdic acid and anisaldehyde solution. For those using the aqueous stains, the TLC plates were heated on a hot plate.

Columns for flash chromatography (FC) contained *silica gel* 200–300 mesh. Columns were packed as slurry of *silica gel* in petroleum ether and equilibrated solution using the appropriate solvent system. The elution was assisted by applying pressure of about 2 atm with an air pump.

Instrumentations

Proton nuclear magnetic resonance (^{1}H NMR), carbon NMR (^{13}C NMR) and fluorous (^{19}F NMR) spectra were recorded in $CDCl_3$ otherwise stated. Chemical shifts are reported in parts per million (ppm), using the residual solvent signal as an internal standard: $CDCl_3$ (^{1}H NMR: δ 7.26, singlet; ^{13}C NMR: δ 77.0, triplet). Coupling constants (J) were recorded in Hertz (Hz). The number of proton atoms (n) for a given resonance was indicated by nH. The number of carbon atoms (n) for a given resonance was indicated by nC. HRMS (Analyzer: TOF) was reported in units of mass of charge ratio (m/z). Mass samples were dissolved in CH_3CN (HPLC Grade) unless otherwise stated. Optical rotations were recorded on a polarimeter with

a sodium lamp of wavelength 589 nm and reported as follows; $[\alpha]_{\lambda}^{T °C}$ (c = g/100 mL, solvent). Melting points were determined on a melting point apparatus.

Enantiomeric excesses were determined by chiral High Performance Liquid Chromatography (HPLC) analysis. UV detection was monitored at 254 nm, 230 nm and 210 nm at the same time. HPLC samples were dissolved in HPLC grade isopropanol (IPA) unless otherwise stated.

Materials

All commercial reagents were purchased with the highest purity grade. They were used without further purification unless specified. All solvents used, mainly petroleum ether (PE) and ethyl acetate (EtOAc) were distilled. Anhydrous dichloromethane (DCM), 1,2-dichloroethane (DCE) and chloroform ($CHCl_3$) were freshly distilled from CaH_2 and stored under N_2 atmosphere. THF, Et_2O and toluene were freshly distilled from sodium/benzophenone before use. Other solvent were purchased with the highest purity grade and without further treatment. Substrates **1**, **3**, **4** and **7** were prepared according to the relevant literatures. [1-4] **D-Amine-1** was prepared according to the relevant literature.[5] All compounds synthesized were stored in a −20 °C freezer and light-sensitive compounds were protected with aluminium foil.

2. Optimization of reaction conditions

Table S1. Optimization of Reaction Conditions for Enantioselectivity of Approaching Chiral Secondary α-Fluoroketones[a]

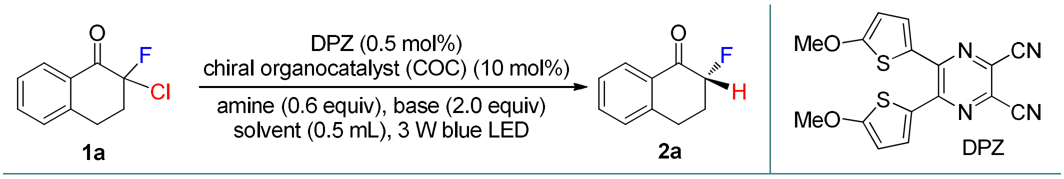

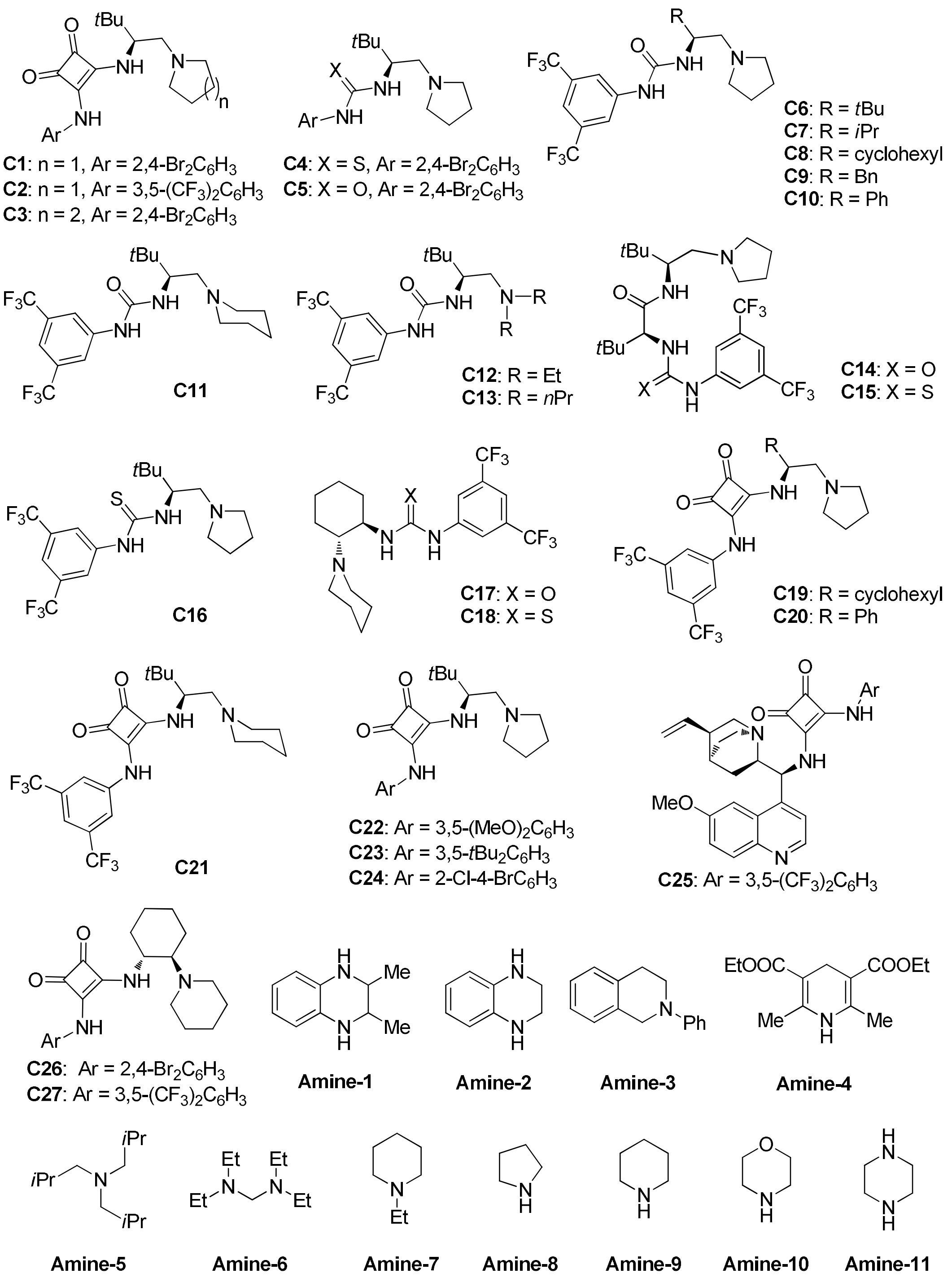

entry	COC	amine	base	solvent	T (°C)	ee (%)[b]
1	**C6**	Et_3N	Na_2CO_3	PhBr	25	12
2	**C6**	DIPEA	Na_2CO_3	PhBr	25	49
3	**C6**	**Amine-3**	Na_2CO_3	PhBr	25	0
4	**C6**	**Amine-4**	Na_2CO_3	PhBr	25	26
5	**C6**	**Amine-5**	Na_2CO_3	PhBr	25	20
6	**C6**	**Amine-6**	Na_2CO_3	PhBr	25	−4
7	**C6**	**Amine-7**	Na_2CO_3	PhBr	25	27
8	**C6**	**Amine-8**	Na_2CO_3	PhBr	25	0
9	**C6**	**Amine-9**	Na_2CO_3	PhBr	25	2
10	**C6**	**Amine-10**	Na_2CO_3	PhBr	25	31
11	**C6**	**Amine-11**	Na_2CO_3	PhBr	25	45
12	**C6**	**Amine-2**	Na_2CO_3	PhBr	25	73
13	**C6**	**Amine-2**	NaF	PhBr	25	18
14	**C6**	**Amine-2**	Na_2SO_3	PhBr	25	22
15	**C6**	**Amine-2**	PhCOONa	PhBr	25	27
16	**C6**	**Amine-2**	$NaHCO_3$	PhBr	25	22
17	**C6**	**Amine-2**	NaOAc	PhBr	25	38
18	**C6**	**Amine-2**	EtONa	PhBr	25	61
19	**C6**	**Amine-2**	Na_2HPO_4	PhBr	25	43
20	**C6**	**Amine-2**	Na_3PO_4	PhBr	25	68
21	**C6**	**Amine-2**	$PhSO_2Na$	PhBr	25	68
22	**C6**	**Amine-2**	KF	PhBr	25	69
23	**C6**	**Amine-2**	KOAc	PhBr	25	49
24	**C6**	**Amine-2**	K_2HPO_4	PhBr	25	67
25	**C6**	**Amine-2**	K_2CO_3	PhBr	25	62
26	**C6**	**Amine-2**	K_3PO_4	PhBr	25	30
27	**C6**	**Amine-2**	Cs_2CO_3	PhBr	25	15
28	**C6**	**Amine-2**	Li_2CO_3	PhBr	25	32
29	**C6**	**Amine-2**	Li_3PO_4	PhBr	25	13
30	**C7**	**Amine-2**	Na_2CO_3	PhBr	25	60
31	**C8**	**Amine-2**	Na_2CO_3	PhBr	25	51
32	**C9**	**Amine-2**	Na_2CO_3	PhBr	25	35
33	**C10**	**Amine-2**	Na_2CO_3	PhBr	25	34
34	**C11**	**Amine-2**	Na_2CO_3	PhBr	25	62
35	**C12**	**Amine-2**	Na_2CO_3	PhBr	25	11
36	**C13**	**Amine-2**	Na_2CO_3	PhBr	25	43
37	**C14**	**Amine-2**	Na_2CO_3	PhBr	25	52
38	**C15**	**Amine-2**	Na_2CO_3	PhBr	25	60
39	**C16**	**Amine-2**	Na_2CO_3	PhBr	25	78
40	**C17**	**Amine-2**	Na_2CO_3	PhBr	25	−32
41	**C18**	**Amine-2**	Na_2CO_3	PhBr	25	−54
42	**C2**	**Amine-2**	Na_2CO_3	PhBr	25	81
43	**C19**	**Amine-2**	Na_2CO_3	PhBr	25	76

44	**C20**	**Amine-2**	Na_2CO_3	PhBr	25	50
45	**C21**	**Amine-2**	Na_2CO_3	PhBr	25	8
46	**C22**	**Amine-2**	Na_2CO_3	PhBr	25	16
47	**C23**	**Amine-2**	Na_2CO_3	PhBr	25	75
48	**C24**	**Amine-2**	Na_2CO_3	PhBr	25	82
49	**C25**	**Amine-2**	Na_2CO_3	PhBr	25	46
50	**C26**	**Amine-2**	Na_2CO_3	PhBr	25	48
51	**C27**	**Amine-2**	Na_2CO_3	PhBr	25	44
52	**C1**	**Amine-2**	Na_2CO_3	PhBr	25	84
53	**C1**	**Amine-1**	Na_2CO_3	PhBr	25	85
54	**C1**	**Amine-1**	Na_2CO_3	toluene	25	73
55	**C1**	**Amine-1**	Na_2CO_3	PhCl	25	82
56	**C1**	**Amine-1**	Na_2CO_3	$PhCF_3$	25	82
57	**C1**	**Amine-1**	Na_2CO_3	C_6F_5H	25	81
58	**C1**	**Amine-1**	Na_2CO_3	*t*BuPh	25	68
59	**C1**	**Amine-1**	Na_2CO_3	anisole	25	80
60	**C1**	**Amine-1**	Na_2CO_3	2-bromoanisole	25	83
61	**C1**	**Amine-1**	Na_2CO_3	3-bromoanisole	25	84
62	**C1**	**Amine-1**	Na_2CO_3	4-bromoanisole	25	85
63	**C1**	**Amine-1**	Na_2CO_3	2-fluoroanisole	25	84
64	**C1**	**Amine-1**	Na_2CO_3	3-fluoroanisole	25	84
65	**C1**	**Amine-1**	Na_2CO_3	4-fluoroanisole	25	85
66	**C1**	**Amine-1**	Na_2CO_3	DCM	25	73
67	**C1**	**Amine-1**	Na_2CO_3	DCE	25	90
68	**C1**	**Amine-1**	Na_2CO_3	$CHCl_3$	25	80
69	**C1**	**Amine-1**	Na_2CO_3	THF	25	55
70	**C1**	**Amine-1**	Na_2CO_3	Et_2O	25	64
71	**C1**	**Amine-1**	Na_2CO_3	DCE	15	91
72	**C1**	**Amine-1**	Na_2CO_3	DCE	10	93
73	**C1**	**Amine-1**	Na_2CO_3	DCE	5	94[c]
74	**C1**	**Amine-1**	Na_2CO_3	DCE	0	94[d]
75	**C1**	**Amine-1**	Na_2CO_3	DCE	−5	93
76	**C1**	**Amine-1**	Na_2CO_3	DCE	−10	93

[a]0.05 mmol scale. For catalyst **C3-C5**, we attempted their viability under the standard reaction conditions. The results could be checked in Table 1 of the manuscript. [b]Determined by HPLC analysis on a chiral stationary phase. 36 h, [c]Yield = 72%. 36 h, [d]Yield = 52%.

Table S2. Optimization of Reaction Conditions for Isolated Yield of Approaching Chiral Secondary α-Fluoroketones[a]

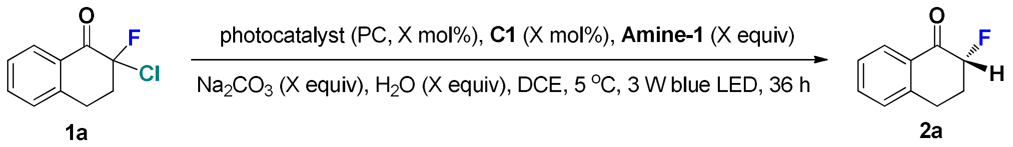

entry	PC (X mol%)	mol% of **C1**	equiv of **Amine-1**	equiv of Na_2CO_3	equiv of H_2O	yield (%)[b]	ee (%)[c]
1	DPZ (0.2)	10	0.6	2.0	--	68	94
2	DPZ (1.0)	10	0.6	2.0	--	73	92
3	DPZ (2.0)	10	0.6	2.0	--	72	92
4	DPZ (0.5)	10	0.6	1.5	--	71	91
5	DPZ (0.5)	10	0.6	2.5	--	74	94
6	DPZ (0.5)	10	0.6	3.0	--	72	91
7	DPZ (0.5)	10	0.4	2.5	--	67	93
8	DPZ (0.5)	10	1.0	2.5	--	74	95
9	DPZ (0.5)	10	2.0	2.5	--	74	93
10	DPZ (0.5)	15	0.6	2.5	--	73	92
11	DPZ (0.5)	5	0.6	2.5	--	74	95
12	DPZ (0.5)	5	0.6	2.5	1.0	92	96
13	DPZ (0.5)	5	0.6	2.5	2.0	91	96
14	DPZ (0.5)	5	0.6	2.5	5.0	89	96
15	DPZ (0.5)	5	0.6	2.5	10.0	72	96
16	Rose Bangel (0.5)	5	0.6	2.5	1.0	54	95
17	Eosin Y (0.5)	5	0.6	2.5	1.0	47	93
18	$Ru(bpy)_3Cl_2\cdot6H_2O$ (0.5)	5	0.6	2.5	1.0	87	95
19	$Ir(ppy)_2(dtbbpy)PF_6$ (0.5)	5	0.6	2.5	1.0	75	90
21	$[Acr-Mes]ClO_4$ (0.5)	5	0.6	2.5	1.0	44	84

[a]0.05 mmol scale. [b]Yield of isolated product. [c]Determined by HPLC analysis on a chiral stationary phase.

Table S3. Optimization of Reaction Conditions for Chiral α-Chlorohydrin[a]

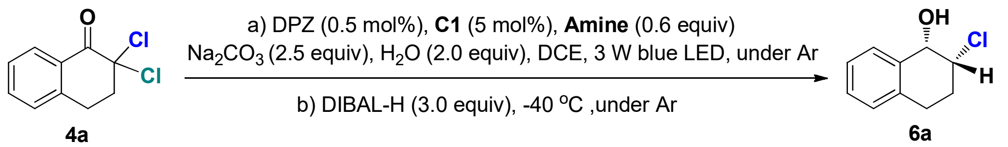

entry	amine	temperature	dr	yield (%)[b]	ee (%)[c]
1	Amine-1	0 °C	>20:1	62%	92%
2	Amine-2	0 °C	>20:1	73%	89%
3	Amine-2	−30 °C	>20:1	79%	92%

[a]0.1 mmol scale. [b]Yield of isolated product. [c]Determined by HPLC analysis on a chiral stationary phase.

3. General experimental procedures

(1) Reaction setup

10 mL Schlenk tube is placed at the center of a stir plate. A 3 W blue LED lamp (HW-450-455LED-3W) is placed to one sidewall of reaction tube (at approximately 2 cm away from the light source). The transformations were conducted in a cryostat which allows a stable and certain temperature.

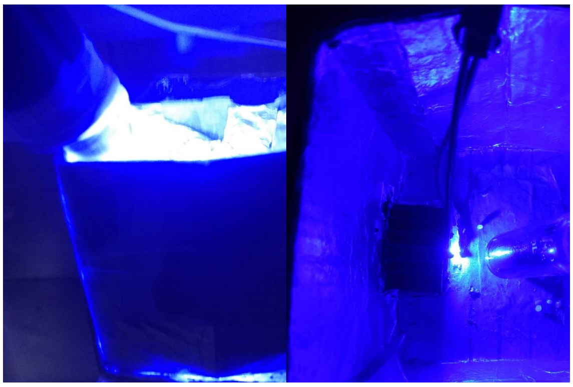

Side view Top View

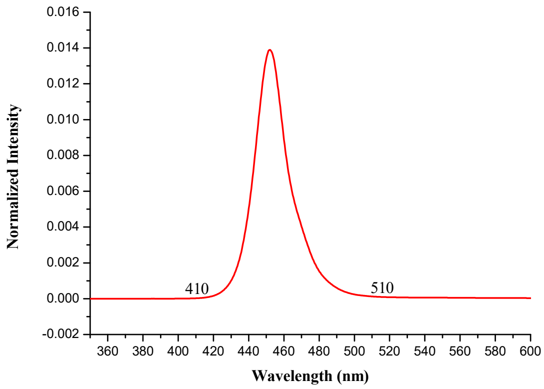

Emission spectrum of the 3 W LED light.

(2) General procedure for the synthesis of chiral secondary α-fluoketones

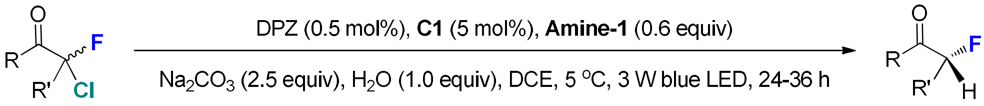

35 μL (0.0005 mmol, 0.005 equiv) of DPZ solution (1.0 mg of DPZ in 200 μL of anhydrous toluene) was added into a 10 mL Schlenk tube, and then solvent was removed in *vacuo*. Subsequently, **C1/C25/C26** (0.005 mmol, 0.05 equiv), Na_2CO_3 (0.25 mmol, 2.5 equiv),

Amine-1 (0.06 mmol, 0.6 equiv), **1** (0.1 mmol, 1.0 equiv), distilled H_2O (0.1 mmol, 1.0 equiv), stir bar and DCE (1.0 mL) were added sequentially and then degassed for three times by freeze-pump-thaw method. The reaction mixture was stirred under an argon atmosphere at 5 ºC for 30 min without light, then irradiated by a 3 W blue LED (λ = 450–455 nm) from a 2.0 cm distance for another 24–36 hours. The reaction mixture was directly loaded onto a short *silica gel* column, followed by gradient elution with petroleum ether/dichloromethane (5/1–2/1 ratio). Removing the solvent in *vacuo*, afforded products **2a-zd**.

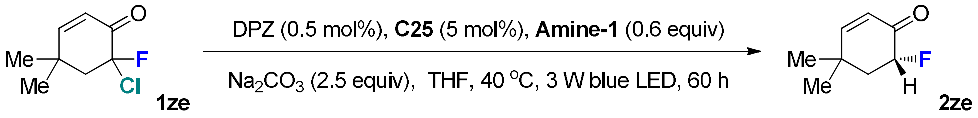

35 μL (0.0005 mmol, 0.005 equiv) of DPZ solution (1.0 mg of DPZ in 200 μL of anhydrous toluene) was added into a 10 mL Schlenk tube, and then solvent was removed in *vacuo*. Subsequently, **C25** (0.005 mmol, 0.05 equiv), Na_2CO_3 (0.25 mmol, 2.5 equiv), **Amine-2** (0.06 mmol, 0.6 equiv), **1ze** (0.1 mmol, 1.0 equiv), stir bar and THF (1.0 mL) were added sequentially and then degassed for three times by freeze-pump-thaw method. The reaction mixture was stirred under an argon atmosphere at 40 ºC for 30 min without light, then irradiated by a 3 W blue LED (λ = 450–455 nm) from a 2.0 cm distance for another 60 hours. The reaction mixture was directly loaded onto a short *silica gel* column, followed by gradient elution with petroleum ether/ ethyl acetate (40/1–10/1 ratio). Removing the solvent in *vacuo*, afforded products **2ze**.

(3) General procedure for the synthesis of chiral secondary α-chloroketones

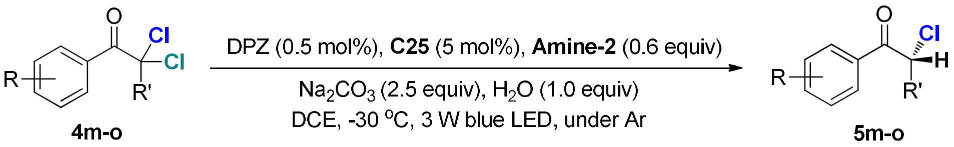

35 μL (0.0005 mmol, 0.005 equiv) of DPZ solution (1.0 mg of DPZ in 200 μL of anhydrous toluene) was added into a 10 mL Schlenk tube, and then solvent was removed in *vacuo*. Subsequently, **C25** (0.005 mmol, 0.05 equiv), Na_2CO_3 (0.25 mmol, 2.5 equiv), **Amine-2** (0.06 mmol, 0.6 equiv), **4** (0.1 mmol, 1.0 equiv), distilled H_2O (0.1 mmol, 1.0 equiv), stir bar and DCE (1.0 mL) were added sequentially and then degassed for three times by freeze-pump-thaw method. The reaction mixture was stirred under an argon atmosphere at

−30 ºC for 30 min without light, then irradiated by a 3 W blue LED (*λ* = 450−455 nm) from a 2.0 cm distance for another 22–42 hours until **4** exhausted monitored by TLC. The reaction mixture was directly loaded onto a short *silica gel* column, followed by gradient elution with petroleum ether/dichloromethane (5/1–2/1 ratio). Removing the solvent in *vacuo*, afforded products **5m-o**.

(4) General procedure for the synthesis of chiral α-chlorohydrins

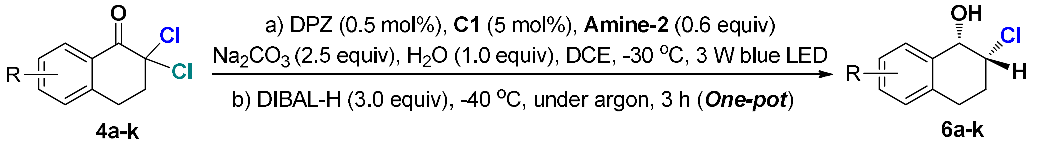

35 *μ*L (0.0005 mmol, 0.005 equiv) of DPZ solution (1.0 mg of DPZ in 200 *μ*L of anhydrous toluene) was added into a 10 mL Schlenk tube, and then solvent was removed in *vacuo*. Subsequently, **C1** (0.005 mmol, 0.05 equiv), Na_2CO_3 (0.25 mmol, 2.5 equiv), **Amine-2** (0.06 mmol, 0.6 equiv), **4a-k** (0.1 mmol, 1.0 equiv), distilled H_2O (0.1 mmol, 1.0 equiv), stir bar and DCE (1.0 mL, **4b** for 2 mL) were added sequentially and then degassed for three times by freeze-pump-thaw method. The reaction mixture was stirred under an argon atmosphere at −30 ºC for 30 min without light, then irradiated by a 3 W blue LED (*λ* = 450−455 nm) from a 2.0 cm distance for another 5–18 hours until **4a-k** exhausted monitored by TLC. Then 3.0 equiv DIBAL-H (1.0 M in toluene) was added slowly at −40 ºC under an argon atmosphere. After stirring for 3 hours, added two drops of H_2O and warmed to room temperature. The reaction mixture was filtered and washed with DCM (5.0 mL), concentrated in *vacuo*, followed by gradient elution with petroleum ether/dichloromethane (5/1–2/1 ratio). Removing the solvent in *vacuo*, afforded products **6a−6k**.

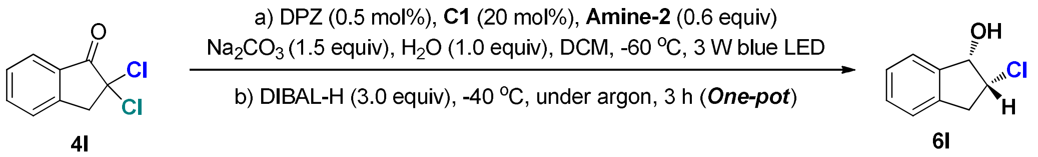

35 *μ*L (0.0005 mmol, 0.005 equiv) of DPZ solution (1.0 mg of DPZ in 200 *μ*L of anhydrous toluene) was added into a 10 mL Schlenk tube, and then solvent was removed in *vacuo*. Subsequently, **C1** (0.02 mmol, 0.2 equiv), Na_2CO_3 (0.15 mmol, 1.5 equiv), **Amine-2** (0.06 mmol, 0.6 equiv), **4l** (0.1 mmol, 1.0 equiv), distilled H_2O (0.1 mmol, 1.0 equiv), stir bar and DCM (1.0 mL) were added sequentially and then degassed for three times by

freeze-pump-thaw method. The reaction mixture was stirred under an argon atmosphere at −60 ºC for 30 min without light, then irradiated by a 3 W blue LED (λ = 450−455 nm) from a 2.0 cm distance for another 48 hours until **4l** exhausted monitored by TLC under Ar atmosphere. Then 3.0 equiv DIBAL-H (1.0 M in toluene) was added slowly at −40 ºC under an argon atmosphere. After stirring for 3 hours, added two drops of H_2O and warmed to room temperature. The reaction mixture was filtered and washed with DCM (5 mL), concentrated in *vacuo*, followed by gradient elution with petroleum ether/dichloromethane (5/1–2/1 ratio). Removing the solvent in *vacuo*, afforded products **6l**.

(5) Procedure for the synthesis of chiral secondary α-bromohydrin 8

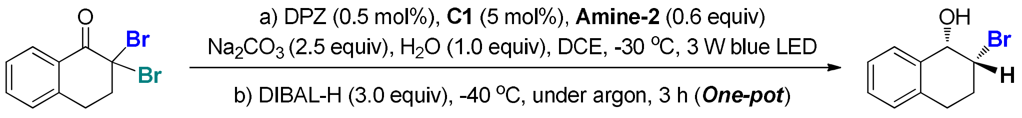

7 **8**

35 μL (0.0005 mmol, 0.005 equiv) of DPZ solution (1.0 mg of DPZ in 200 μL of anhydrous toluene) was added into a 10 mL Schlenk tube, and then solvent was removed in *vacuo*. Subsequently, **C1** (0.005 mmol, 0.05 equiv), Na_2CO_3 (0.25 mmol, 2.5 equiv), **Amine-2** (0.06 mmol, 0.6 equiv), **7** (0.1 mmol, 1.0 equiv), distilled H_2O (0.1 mmol, 1.0 equiv), stir bar and DCE (1.0 mL) were added sequentially and then degassed for three times by freeze-pump-thaw method. The reaction mixture was stirred under an argon atmosphere at −30 ºC for 30 min without light, then irradiated by a 3 W blue LED (λ = 450−455 nm) from a 2.0 cm distance for another 2 hours until **7** exhausted monitored by TLC. Then 3.0 equiv DIBAL-H (1.0 M in toluene) was added slowly at −40 ºC under an argon atmosphere. After stirring for 3 hours, added two drops of H_2O and warmed to room temperature. The reaction mixture was filtered and washed with DCM (5.0 mL), concentrated in *vacuo*. followed by gradient elution with petroleum ether/dichloromethane (5/1–2/1 ratio). Removing the solvent in *vacuo*, afforded products **8**.

Note: All racemic samples for determining HPLC conditions and ee values of chiral compounds were prepared through: 0.5 mol% DPZ, 0.6 equiv. of **Amine-1**, 2.5 equiv. of Na_2CO_3 in DCE at 25 ºC (for racemic products **2** and **5m-o**); 0.5 mol% DPZ, 0.6 equiv. of **Amine-1**, 2.5 equiv. of Na_2CO_3 in DCE at 25 ºC. After 6 h, 3.0 equiv. of DIBAL-H was added and the transformations were conducted at −40 ºC (for racemic products **6** and **8**)

4. Mechanism studies

Emission quenching experiments

Emission intensities were recorded on a spectrofluorometer. DPZ solution was excited at 448 nm and the emission intensity at 544 nm was observed. A solution of DPZ (2.5×10^{-4} M) in MeCN was added to the appropriate amount of quencher in 5.0 mL volumetric flask under N_2. The solution was transferred to a 1.5 mL quartz cell and the emission spectrum of the sample was collected.

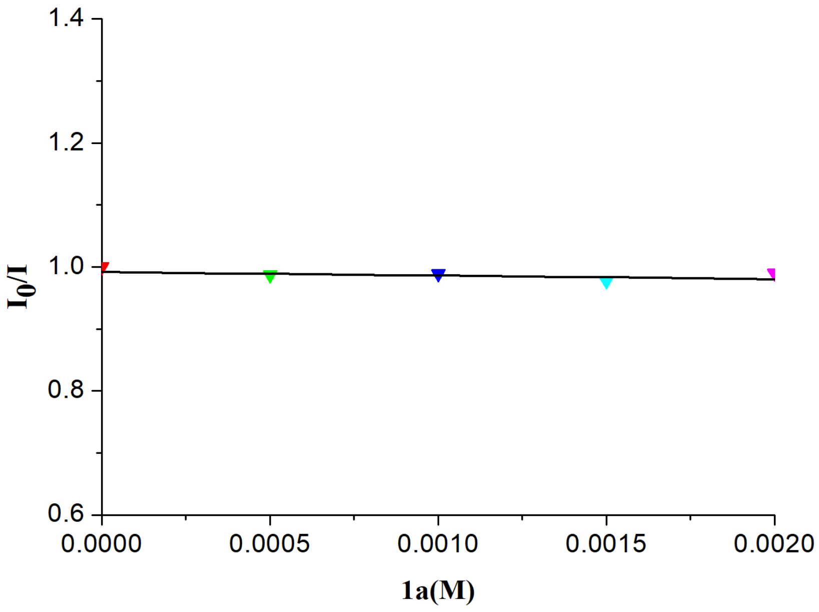

Fig. S1 Stern–Volmer quenching experiment of DPZ and **1a**.

(Result: no quenching observed)

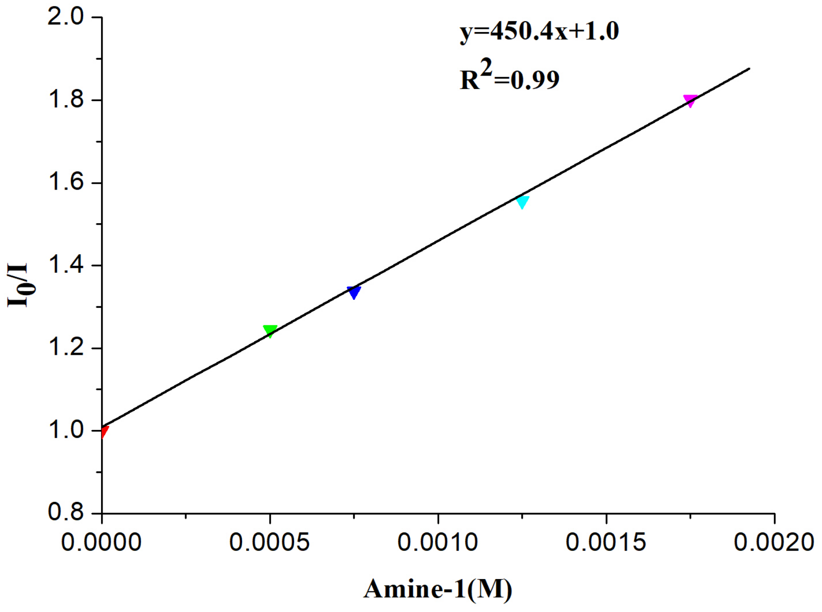

Fig. S2 Stern–Volmer quenching experiment of DPZ and **Amine-1**.

(Result: quenching observed)

Cyclic voltammetry measurement

Cyclic voltammetry experiments were performed on a CHI600E Workstation. Measurements were performed for anhydrous acetonitrile solutions ([sample] = 1.0 mM, $[(NBu_4)PF_6]$ = 0.10 M) with a radium glassy carbon (working electrode) and platinum wire (counter electrode), and a Ag/AgCl($AgNO_3$) reference electrode under N_2 at room temperature. The scan rate was 50 mV/s. Ferrocene (Cp_2Fe) was used as a reference. The obtained value was referenced to Ag/AgCl and converted to SCE by adding 0.03 V. The obtained value was referenced to Ag/$AgNO_3$ and converted to SCE by adding 0.337 V.

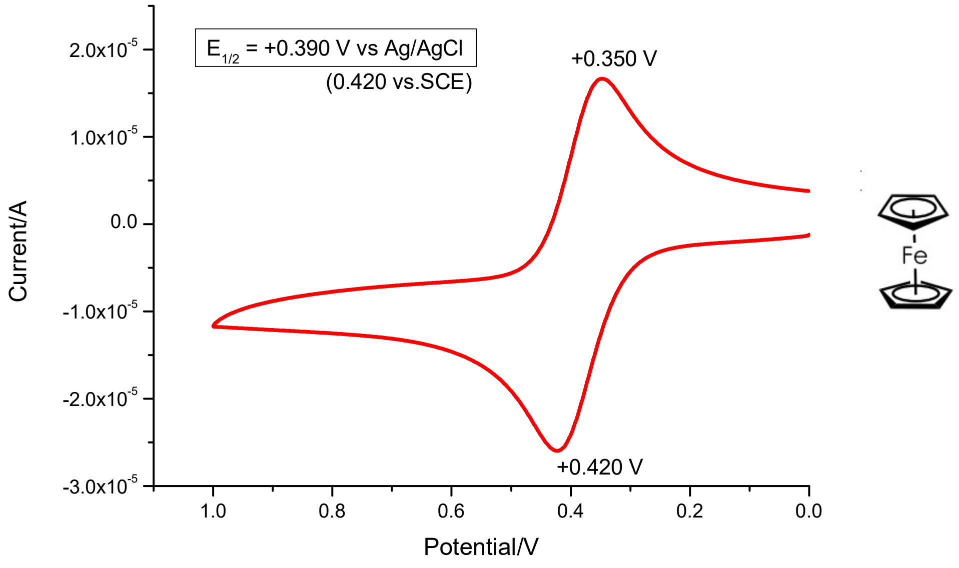

Fig. S3. Cyclic voltammogram of Ferrocene with a Ag/AgCl reference electrode.

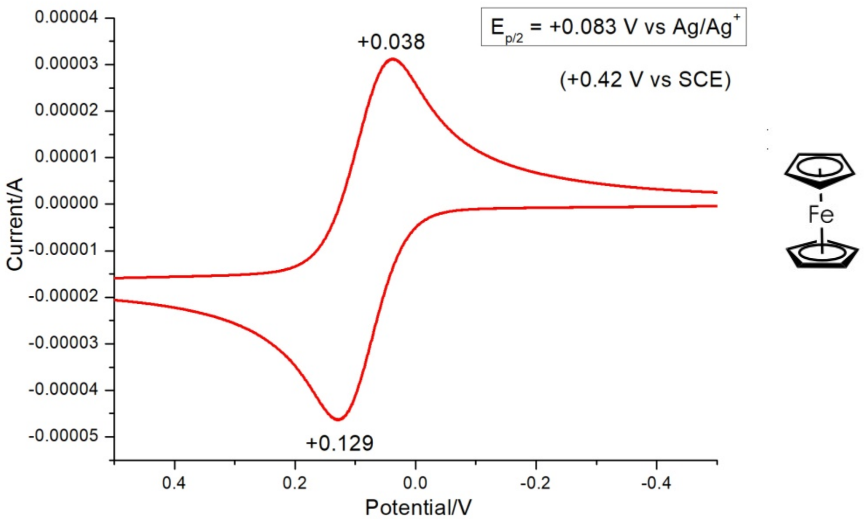

Fig. S4. Cyclic voltammogram of Ferrocene in MeCN with a Ag/$AgNO_3$ reference electrode.

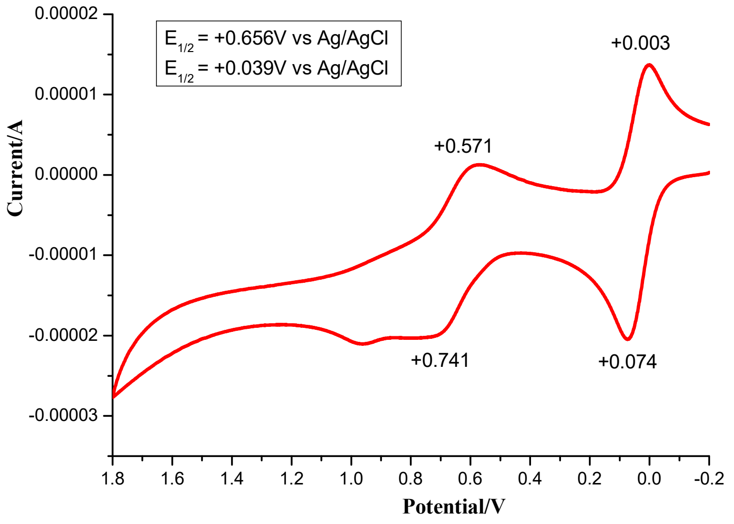

Fig. S5. Cyclic voltammogram of **Amine-1** in MeCN. $E_{1/2}$ = +0.686 V versus SCE in CH_3CN, $E_{1/2}$ = +0.069 V versus SCE in CH_3CN.

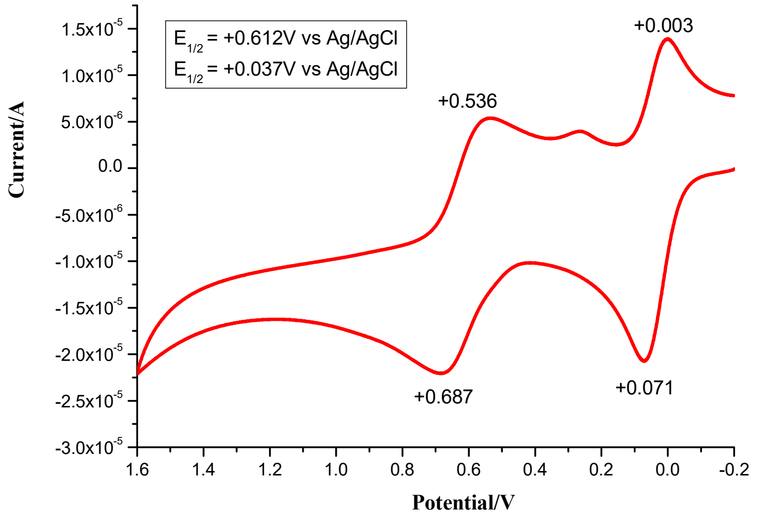

Fig. S6 Cyclic voltammogram of **Amine-2** in MeCN. $E_{1/2}$ = +0.642 V versus SCE in CH_3CN, $E_{1/2}$ = +0.067 V versus SCE in CH_3CN.

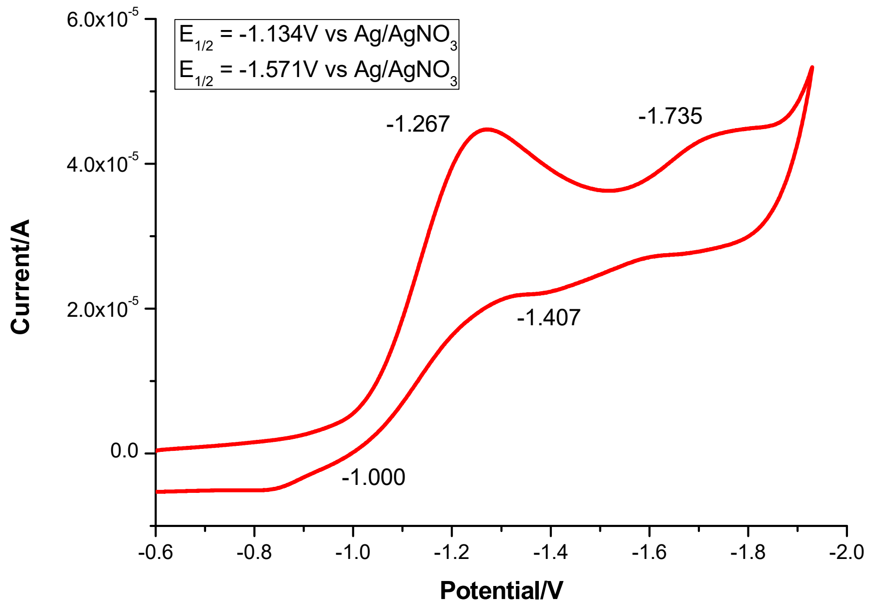

Fig. S7 Cyclic voltammogram of **1a** in MeCN. $E_{1/2}$ = –0.797 V versus SCE in CH_3CN, $E_{1/2}$ = –1.234 V versus SCE in CH_3CN.

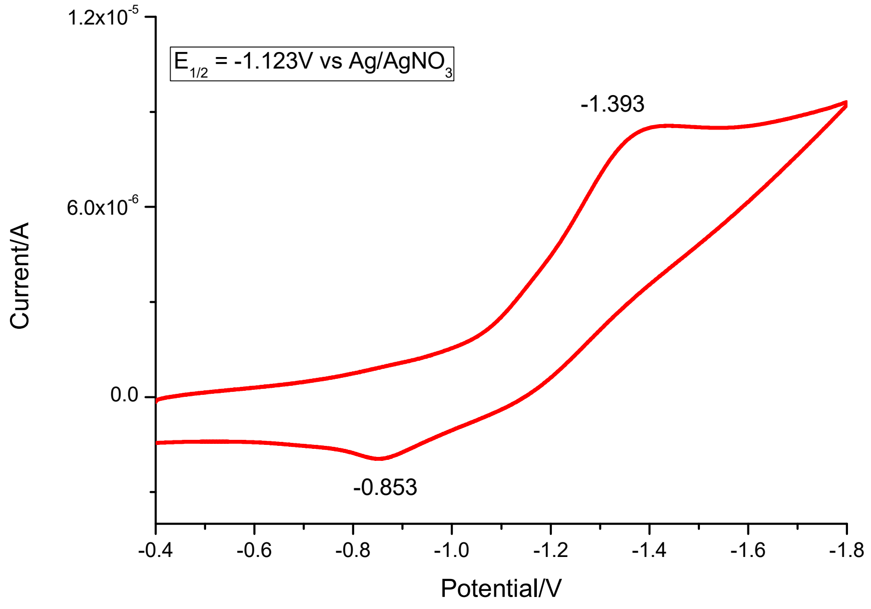

Fig. S8 Cyclic voltammogram of **2a** in MeCN. $E_{1/2}$ = –0.786 V versus SCE in CH_3CN.

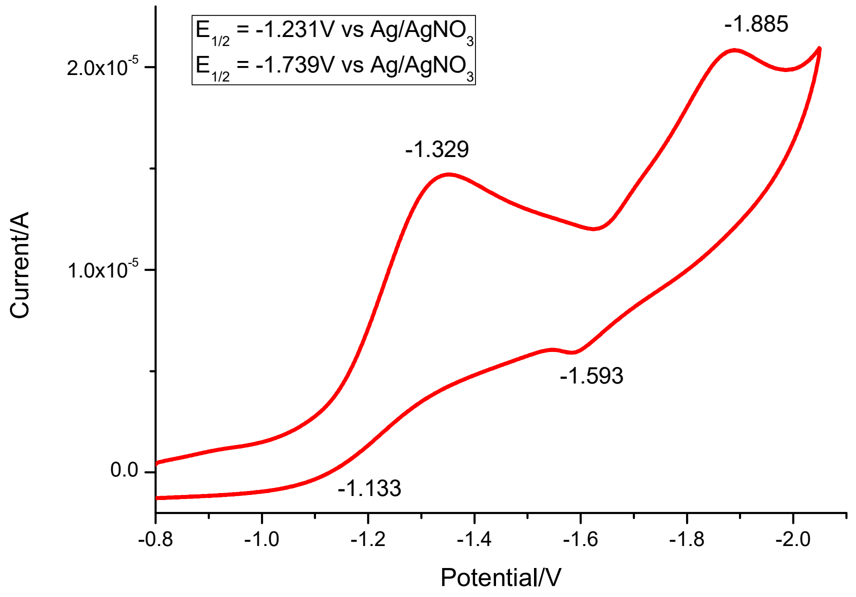

Fig. S9 Cyclic voltammogram of **1b** in MeCN. $E_{1/2}$ = –0.894 V versus SCE in CH_3CN, $E_{1/2}$ = –1.402 V versus SCE in CH_3CN.

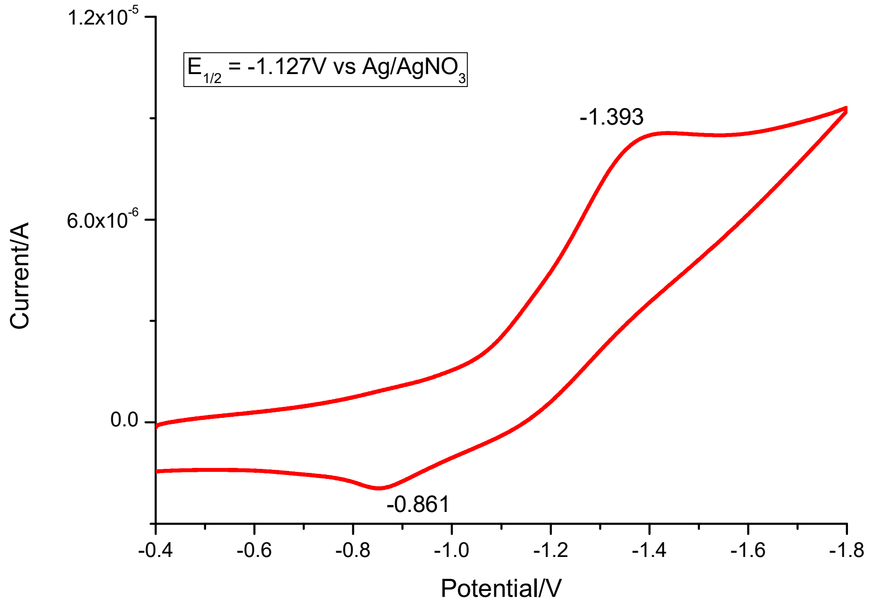

Fig. S10 Cyclic voltammogram of **2zb** in MeCN. $E_{1/2}$ = –0.790 V versus SCE in CH_3CN.

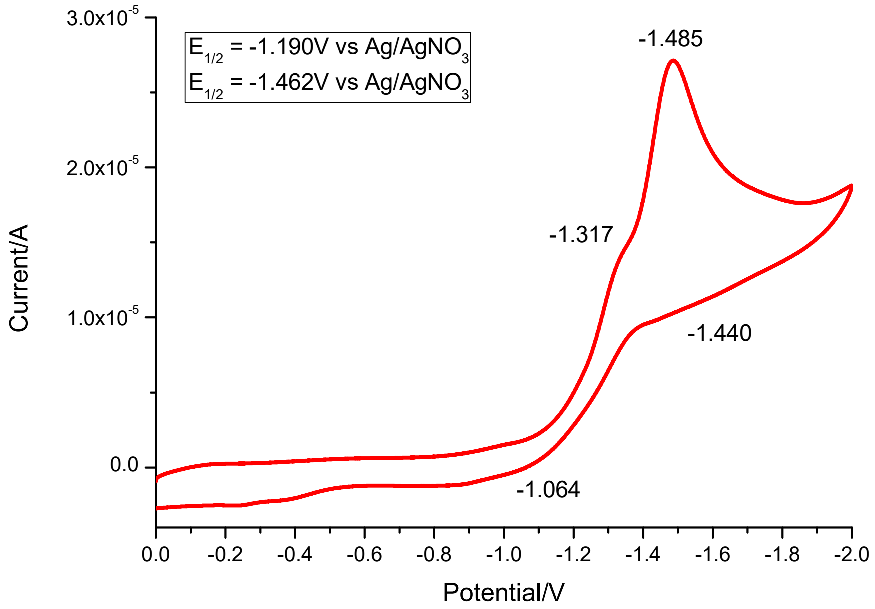

Fig. S11 Cyclic voltammogram of **4a** in MeCN. $E_{1/2}$ = –0.853 V versus SCE in CH_3CN, $E_{1/2}$ = –1.125 V versus SCE in CH_3CN.

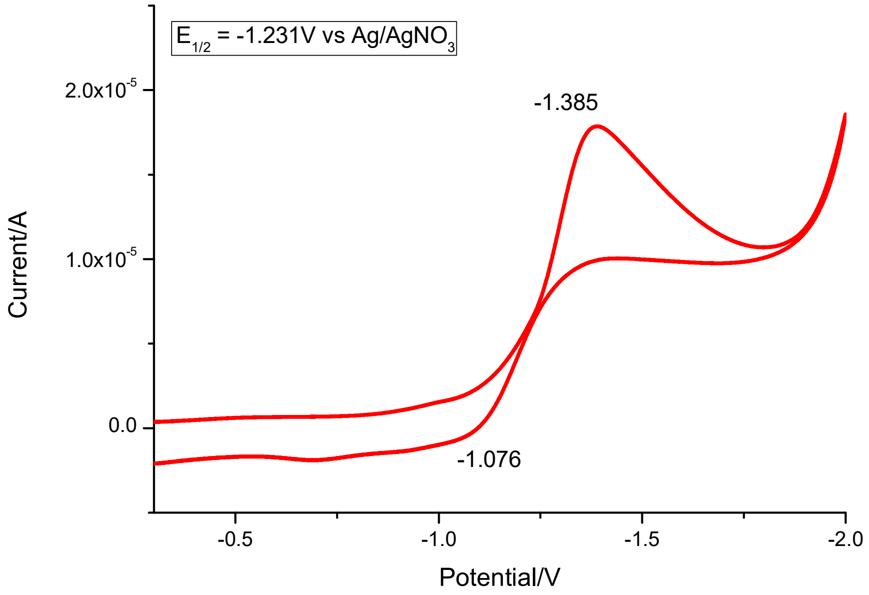

Fig. S12 Cyclic voltammogram of **5a** in MeCN. $E_{1/2}$ = –0.894 V versus SCE in CH_3CN.

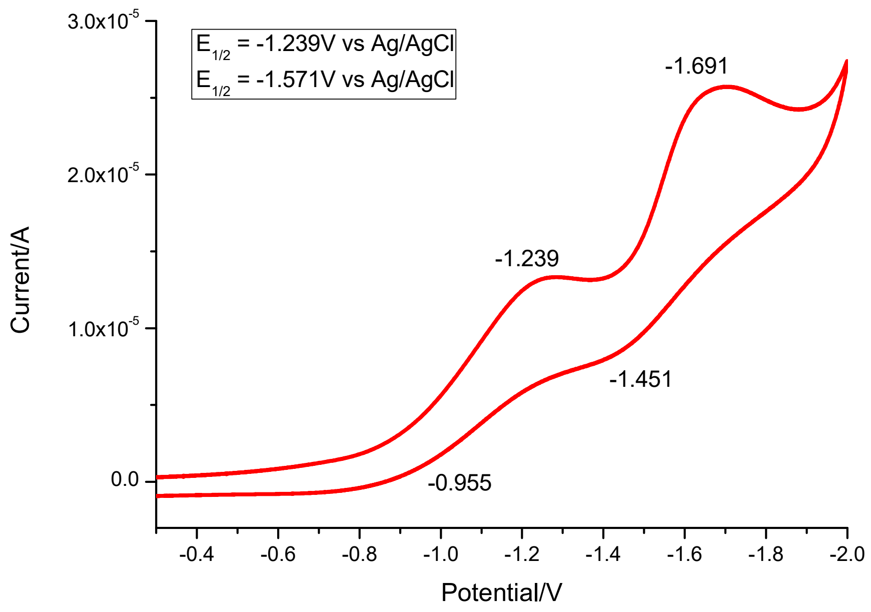

Fig. S13 Cyclic voltammogram of **4n** in MeCN. $E_{1/2}$ = –1.209 V versus SCE in CH_3CN, $E_{1/2}$ = –1.541 V versus SCE in CH_3CN.

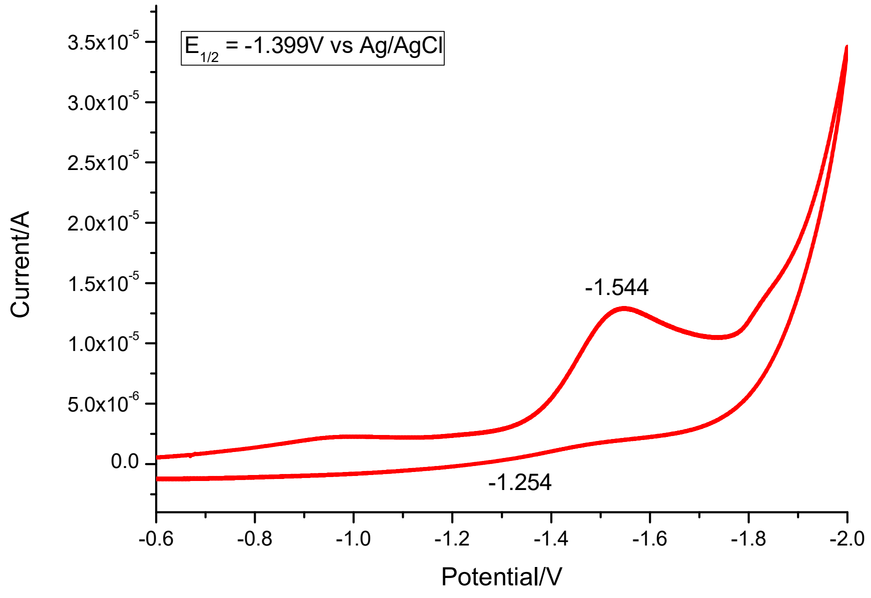

Fig. S14 Cyclic voltammogram of **5n** in MeCN. $E_{1/2}$ = –1.369 V versus SCE in CH_3CN.

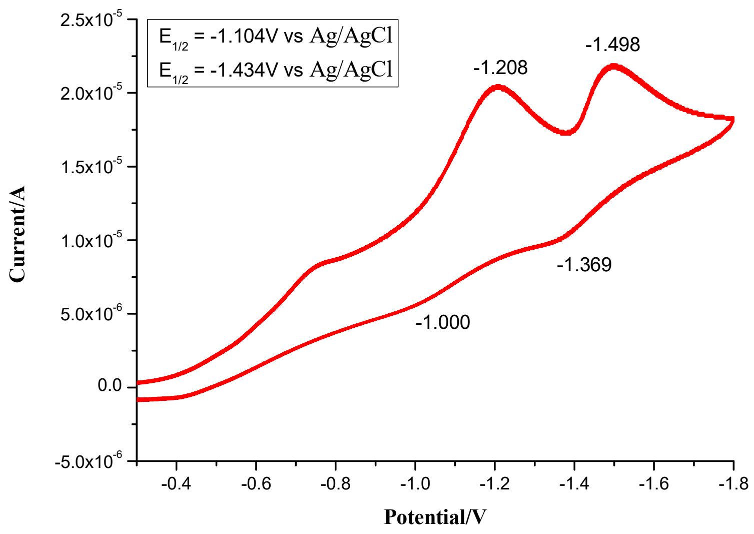

Fig. S15 Cyclic voltammogram of **7** in MeCN. $E_{1/2}$ = –1.074 V versus SCE in CH_3CN, $E_{1/2}$ = –1.404 V versus SCE in CH_3CN.

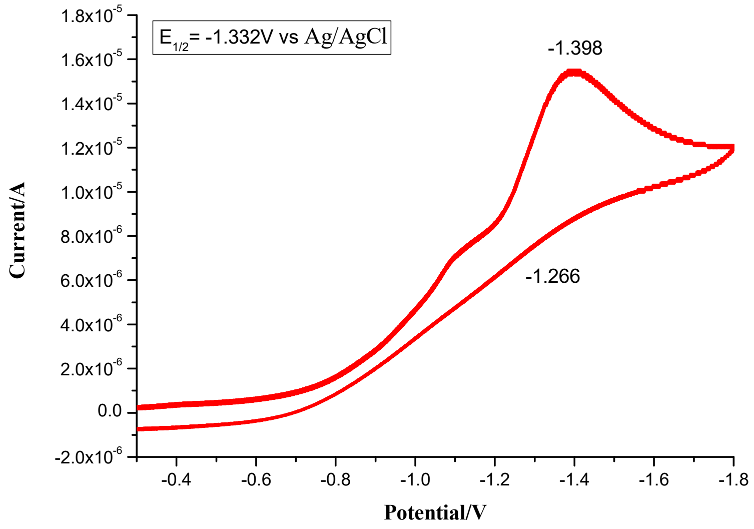

Fig. S16 Cyclic voltammogram of **8** in MeCN. $E_{1/2}$ = –1.302 V versus SCE in CH_3CN.

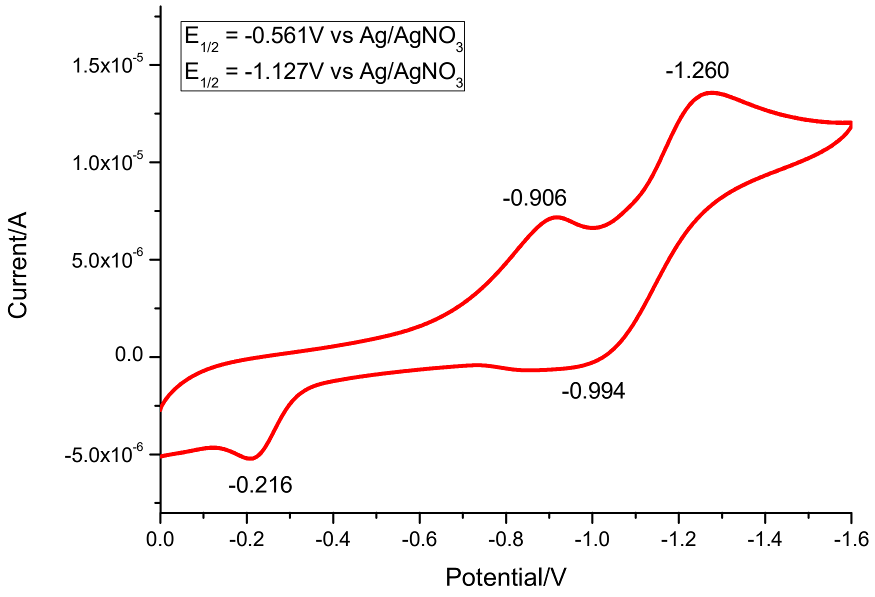

Fig. S17 Cyclic voltammogram of **C1** in MeCN. $E_{1/2}$ = –0.224 V versus SCE in CH_3CN, $E_{1/2}$ = –0.790 V versus SCE in CH_3CN.

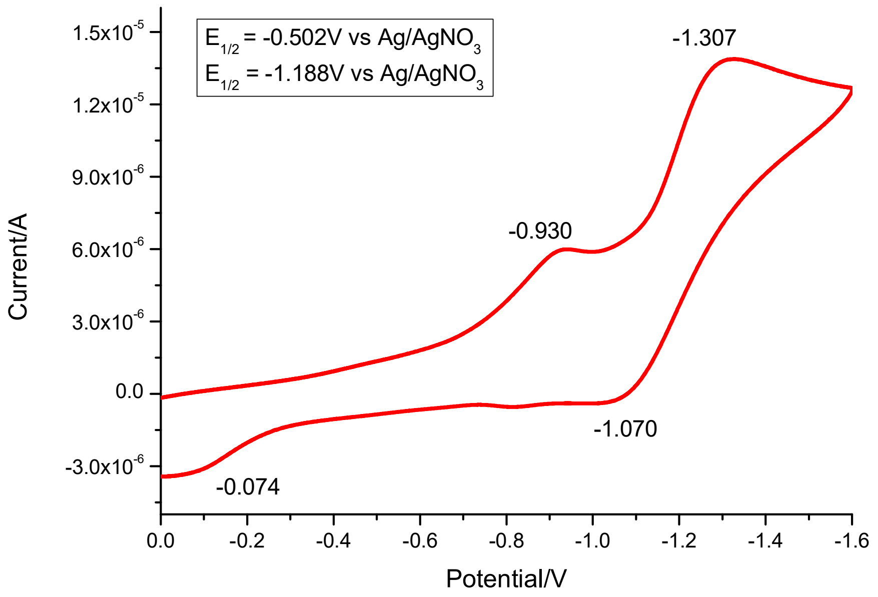

Fig. S18 Cyclic voltammogram of **C25** in MeCN. $E_{1/2}$ = –0.165 V versus SCE in CH_3CN, $E_{1/2}$ = –0.851 V versus SCE in CH_3CN.

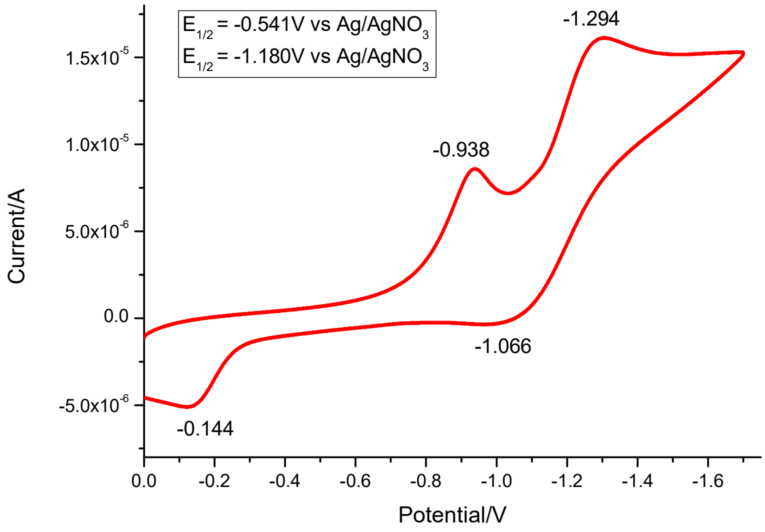

Fig. S19 Cyclic voltammogram of **C26** in MeCN. $E_{1/2}$ = –0.204 V versus SCE in CH_3CN, $E_{1/2}$ = –0.843 V versus SCE in CH_3CN.

UV-vis experiments.

Solutions of different complexes were introduced to a 3 cm path length quartz cuvette equipped with a Teflon® septum and analyzed using a TU-1901 spectrophotometer.

For the solutions of **1a** in DCE: **1a** (7.92 mg, 0.04 mmol) was dissolved in DCE (40 mL), then transformed to 3 cm path length quartz cuvettes, and sealed with a Teflon® septa.

For the solutions of **Amine-1** in DCE: **Amine-1** (3.89 mg, 0.024 mmol) was dissolved in DCE (40 mL), then transformed to 3 cm path length quartz cuvettes, and sealed with a Teflon® septa.

For the solutions of **1a** and Na_2CO_3 in DCE: **1a** (7.92 mg, 0.04 mmol) and Na_2CO_3 (10.6 mg, 0.1 mmol) were dissolved in DCE (40 mL), then transformed to 3 cm path length quartz cuvettes, and sealed with a Teflon® septa.

For the solutions of **1a** and **Amine-1** in DCE: **1a** (7.92 mg, 0.04 mmol) and **Amine-1** (3.89 mg, 0.024 mmol) were dissolved in DCE (40 mL), then transformed to 3 cm path length

quartz cuvettes, and sealed with a Teflon® septa.

For the solutions of **1a**, **Amine-1**, and Na_2CO_3 in DCE: **1a** (7.92 mg, 0.04 mmol), **Amine-1** (3.89 mg, 0.024 mmol), and Na_2CO_3 (10.6 mg, 0.1 mmol) were dissolved in DCE (40 mL), then transformed to 3 cm path length quartz cuvettes, and sealed with a Teflon® septa.

For the solutions of **1a**, **Amine-1**, Na_2CO_3, and **C1** in DCE: **1a** (7.92 mg, 0.04 mmol), **Amine-1** (3.89 mg, 0.024 mmol), Na_2CO_3 (10.6 mg, 0.1 mmol) and **C1** (0.99 mg, 0.002 mmol) were dissolved in DCE (40 mL), then transformed to 3 cm path length quartz cuvettes, and sealed with a Teflon® septa.

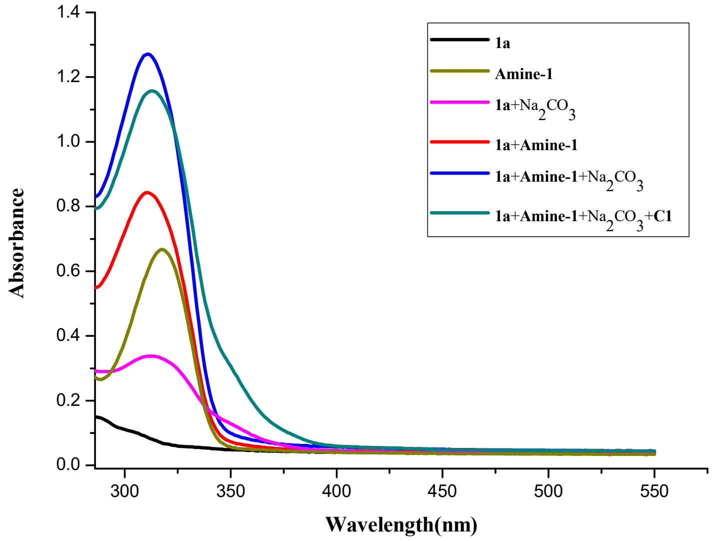

Fig. S20 Absorption spectra of various complexes.

For the solutions of **2a** in DCE: **2a** (6.56 mg, 0.04 mmol) was dissolved in DCE (40 mL), then transformed to 3 cm path length quartz cuvettes, and sealed with a Teflon® septa.

For the solutions of **Amine-1** in DCE: **Amine-1** (3.89 mg, 0.024 mmol) was dissolved in DCE (40 mL), then transformed to 3 cm path length quartz cuvettes, and sealed with a Teflon® septa.

For the solutions of **2a** and Na_2CO_3 in DCE: **2a** (6.56 mg, 0.04 mmol) and Na_2CO_3 (10.6 mg, 0.1 mmol) were dissolved in DCE (40 mL), then transformed to 3 cm path length quartz

cuvettes, and sealed with a Teflon® septa.

For the solutions of **2a** and **Amine-1** in DCE: **2a** (6.56 mg, 0.04 mmol) and **Amine-1** (3.89 mg, 0.024 mmol) were dissolved in DCE (40 mL), then transformed to 3 cm path length quartz cuvettes, and sealed with a Teflon® septa.

For the solutions of **2a**, **Amine-1**, and Na_2CO_3 in DCE: **2a** (6.56 mg, 0.04 mmol), **Amine-1** (3.89 mg, 0.024 mmol), and Na_2CO_3 (10.6 mg, 0.1 mmol) were dissolved in DCE (40 mL), then transformed to 3 cm path length quartz cuvettes, and sealed with a Teflon® septa.

For the solutions of **2a**, **Amine-1**, Na_2CO_3, and **C1** in DCE: **2a** (6.56 mg, 0.04 mmol), **Amine-1** (3.89 mg, 0.024 mmol), Na_2CO_3 (10.6 mg, 0.1 mmol) and **C1** (0.99 mg, 0.002 mmol) were dissolved in DCE (40 mL), then transformed to 3 cm path length quartz cuvettes, and sealed with a Teflon® septa.

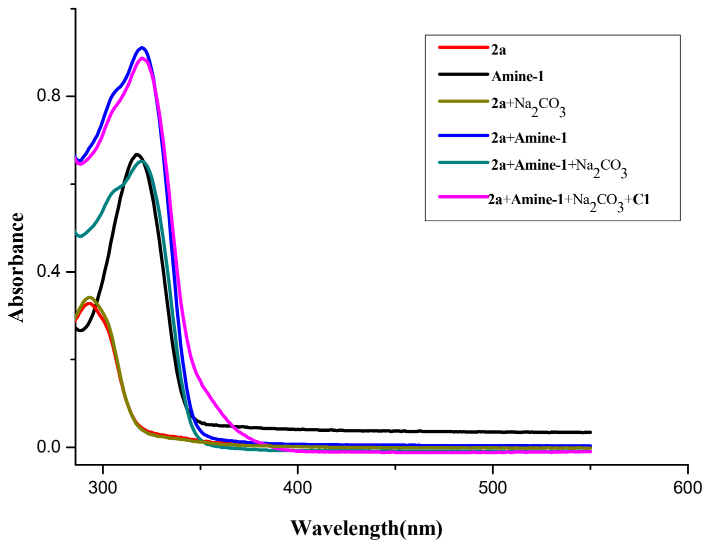

Fig. S21 Absorption spectra of various complexes.

The study of the proton source

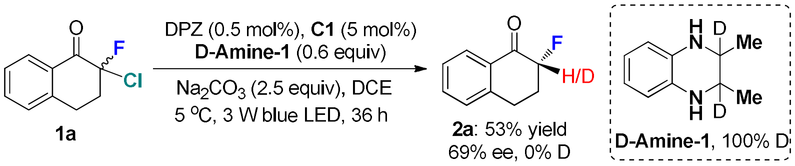

35 μL (0.0005 mmol, 0.005 equiv) of DPZ solution (1.0 mg of DPZ in 200 μL of anhydrous toluene) was added into a 10 mL Schlenk tube, and then solvent was removed in *vacuo*. Subsequently, **C1** (2.48 mg, 0.005 mmol, 0.05 equiv), Na_2CO_3 (26.5 mg, 0.25 mmol, 2.5 equiv), **D-Amine-1** (9.84 mg, 0.06 mmol, 0.6 equiv), **1a** (19.8 mg, 0.1 mmol, 1.0 equiv), and stir bar and DCE (1.0 mL) were added sequentially and then degassed for three times by freeze-pump-thaw method. The reaction mixture was stirred under an argon atmosphere at 5 °C for 30 min without light, then irradiated by a 3 W blue LED (λ = 450–455 nm) from a 2.0 cm distance for another 36 hours. The reaction mixture was directly loaded onto a short *silica gel* column, followed by gradient elution with petroleum ether/dichloromethane (5/1–2/1 ratio). Removing the solvent in *vacuo*, afforded products **2a** with 0% D incorporation.

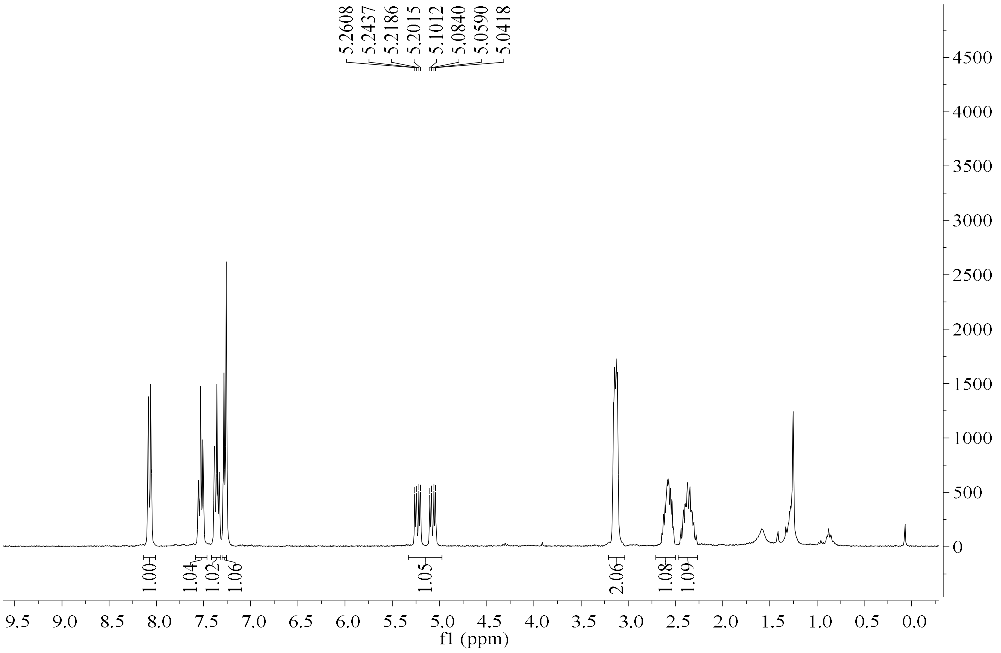

^{1}H NMR of 2a (D-Amine-1 as the sacrificial reductant)

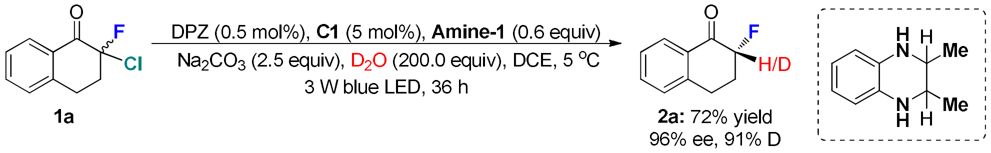

35 μL (0.0005 mmol, 0.005 equiv) of DPZ solution (1.0 mg of DPZ in 200 μL of anhydrous toluene) was added into a 10 mL Schlenk tube, and then solvent was removed in *vacuo*. Subsequently, **C1** (2.48 mg, 0.005 mmol, 0.05 equiv), Na_2CO_3 (26.5 mg, 0.25 mmol, 2.5 equiv), **Amine-1** (9.72 mg, 0.06 mmol, 0.6 equiv), **1a** (19.8 mg, 0.1 mmol, 1.0 equiv), D_2O (0.4 mL, 20 mmol, 200 equiv), stir bar and DCE (1.0 mL) were added sequentially and then degassed for three times by freeze-pump-thaw method. The reaction mixture was stirred under an argon atmosphere at 5 °C for 30 min without light, then irradiated by a 3 W blue LED (λ = 450−455 nm) from a 2.0 cm distance for another 36 hours. The reaction mixture was directly loaded onto a short *silica gel* column, followed by gradient elution with petroleum ether/dichloromethane (5/1–2/1 ratio). Removing the solvent in *vacuo*, afforded products **2a** in 72% yield with 96% ee and 91% D incorporation.

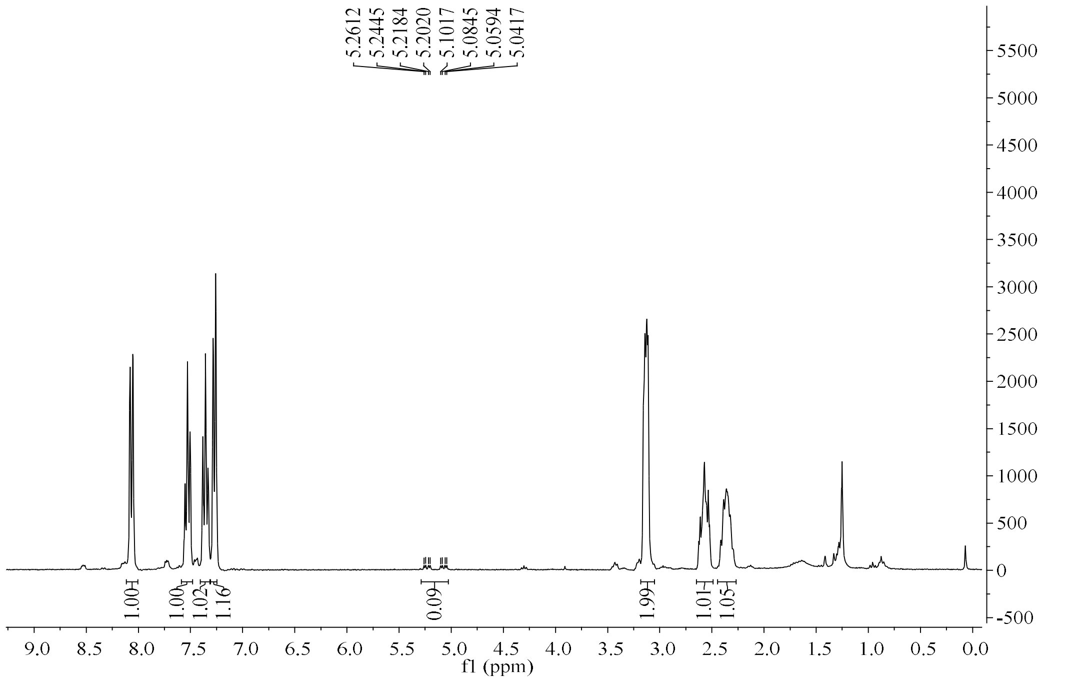

^{1}H NMR of 2a (D_2O as the proton source)

These reasults indicated that the proton might be from free H^+ of the reaction system.

Control experiments

(a)

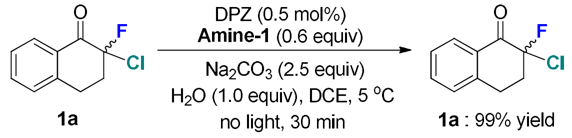

35 μL (0.0005 mmol, 0.005 equiv) of DPZ solution (1.0 mg of DPZ in 200 μL of anhydrous toluene) was added into a 10 mL Schlenk tube, and then solvent was removed in *vacuo*. Subsequently, **C1** (2.48 mg, 0.005 mmol, 0.05 equiv), Na_2CO_3 (26.5 mg, 0.25 mmol, 2.5 equiv), **Amine-1** (9.72 mg, 0.06 mmol, 0.6 equiv), **1a** (19.8 mg, 0.1 mmol, 1.0 equiv), H_2O (1.8 µL, 0.1 mmol, 1.0 equiv), stir bar and DCE (1.0 mL) were added sequentially and then degassed for three times by freeze-pump-thaw method. The reaction mixture was stirred under an argon atmosphere at 5 °C for 30 min without light. The reaction mixture was directly loaded onto a short *silica gel* column, followed by gradient elution with petroleum ether/dichloromethane (5/1–3/1 ratio). Removing the solvent in *vacuo*, recovered **1a** in 99% yield.

(b)

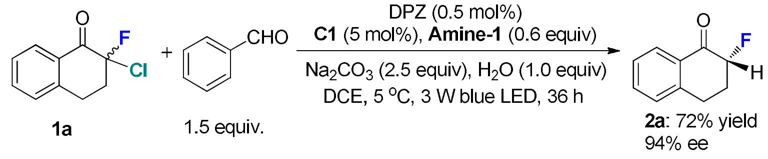

35 μL (0.0005 mmol, 0.005 equiv) of DPZ solution (1.0 mg of DPZ in 200 μL of anhydrous toluene) was added into a 10 mL Schlenk tube, and then solvent was removed in *vacuo*. Subsequently, **C1** (2.48 mg, 0.005 mmol, 0.05 equiv), Na_2CO_3 (26.5 mg, 0.25 mmol, 2.5 equiv), **Amine-1** (9.72 mg, 0.06 mmol, 0.6 equiv), **1a** (19.8 mg, 0.1 mmol, 1.0 equiv), benzaldehyde(15.3 µL, 0.15 mmol, 1.5 equiv), H_2O (1.8 µL, 0.1 mmol, 1.0 equiv), stir bar and DCE (1.0 mL) were added sequentially and then degassed for three times by freeze-pump-thaw method. The reaction mixture was stirred under an argon atmosphere at 5 °C for 30 min without light, then irradiated by a 3 W blue LED (λ = 450–455 nm) from a 2.0 cm distance for another 10 hours. The reaction mixture was directly loaded onto a short *silica gel* column, followed by gradient elution with petroleum ether/dichloromethane (5/1–2/1 ratio). Removing the solvent in *vacuo*, afforded products **2a** in 72% yield with 94% ee.

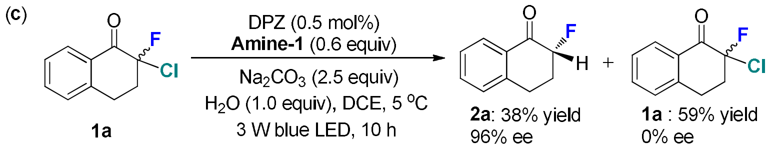

35 μL (0.0005 mmol, 0.005 equiv) of DPZ solution (1.0 mg of DPZ in 200 μL of anhydrous toluene) was added into a 10 mL Schlenk tube, and then solvent was removed in *vacuo*. Subsequently, **C1** (2.48 mg, 0.005 mmol, 0.05 equiv), Na_2CO_3 (26.5 mg, 0.25 mmol, 2.5 equiv), **Amine-1** (9.72 mg, 0.06 mmol, 0.6 equiv), **1a** (19.8 mg, 0.1 mmol, 1.0 equiv), H_2O (1.8 µL, 0.1 mmol, 1.0 equiv), stir bar and DCE (1.0 mL) were added sequentially and then degassed for three times by freeze-pump-thaw method. The reaction mixture was stirred under an argon atmosphere at 5 °C for 30 min without light, then irradiated by a 3 W blue LED (λ = 450−455 nm) from a 2.0 cm distance for another 10 hours. The reaction mixture was directly loaded onto a short *silica gel* column, followed by gradient elution with petroleum ether/dichloromethane (5/1–2/1 ratio). Removing the solvent in *vacuo*, afforded products **2a** in 38% yield with 96% ee, and recovered **1a** in 59% yield with 0 ee.

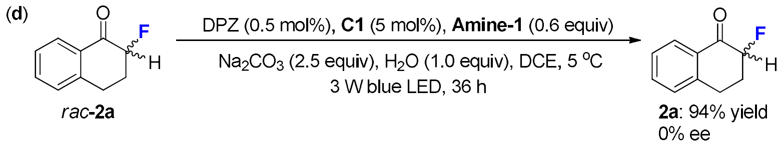

35 μL (0.0005 mmol, 0.005 equiv) of DPZ solution (1.0 mg of DPZ in 200 μL of anhydrous toluene) was added into a 10 mL Schlenk tube, and then solvent was removed in *vacuo*. Subsequently, **C1** (2.48 mg, 0.005 mmol, 0.05 equiv), Na_2CO_3 (26.5 mg, 0.25 mmol, 2.5 equiv), **Amine-1** (9.72 mg, 0.06 mmol, 0.6 equiv), *rac*-**2a** (16.4 mg, 0.1 mmol, 1.0 equiv), H_2O (1.8 µL, 0.1 mmol, 1.0 equiv), stir bar and DCE (1.0 mL) were added sequentially and then degassed for three times by freeze-pump-thaw method. The reaction mixture was stirred under an argon atmosphere at 5 °C for 30 min without light, then irradiated by a 3 W blue LED (λ = 450−455 nm) from a 2.0 cm distance for another 10 hours. The reaction mixture was directly loaded onto a short *silica gel* column, followed by gradient elution with petroleum ether/dichloromethane (5/1–2/1 ratio). Removing the solvent in *vacuo*, afforded products **2a** in 94% yield with 0 ee.

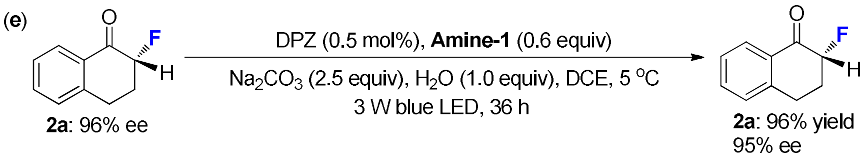

35 μL (0.0005 mmol, 0.005 equiv) of DPZ solution (1.0 mg of DPZ in 200 μL of anhydrous toluene) was added into a 10 mL Schlenk tube, and then solvent was removed in *vacuo*. Subsequently, Na_2CO_3 (26.5 mg, 0.25 mmol, 2.5 equiv), **Amine-1** (9.72 mg, 0.06 mmol, 0.6 equiv), **2a** (96% ee, 16.4 mg, 0.1 mmol, 1.0 equiv), H_2O (1.8 μL, 0.1 mmol, 1.0 equiv), stir bar and DCE (1.0 mL) were added sequentially and then degassed for three times by freeze-pump-thaw method. The reaction mixture was stirred under an argon atmosphere at 5 °C for 30 min without light, then irradiated by a 3 W blue LED (λ = 450–455 nm) from a 2.0 cm distance for another 10 hours. The reaction mixture was directly loaded onto a short *silica gel* column, followed by gradient elution with petroleum ether/dichloromethane (5/1–2/1 ratio). Removing the solvent in *vacuo*, afforded products **2a** in 96% yield with 95% ee.

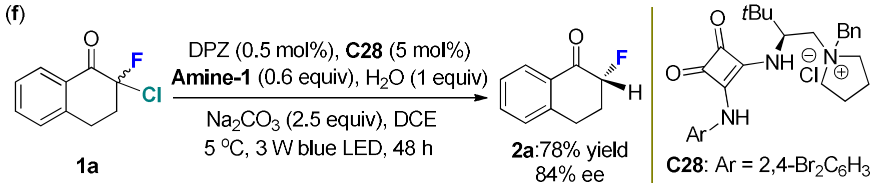

35 μL (0.0005 mmol, 0.005 equiv) of DPZ solution (1.0 mg of DPZ in 200 μL of anhydrous toluene) was added into a 10 mL Schlenk tube, and then solvent was removed in *vacuo*. Subsequently, **C28** (3.1 mg, 0.005 mmol, 0.05 equiv), Na_2CO_3 (26.5 mg, 0.25 mmol, 2.5 equiv), **Amine-1** (9.72 mg, 0.06 mmol, 0.6 equiv), **1a** (19.8 mg, 0.1 mmol, 1.0 equiv), H_2O (1.8 μL, 0.1 mmol, 1.0 equiv), stir bar and DCE (1.0 mL) were added sequentially and then degassed for three times by freeze-pump-thaw method. The reaction mixture was stirred under an argon atmosphere at 5 °C for 30 min without light, then irradiated by a 3 W blue LED (λ = 450–455 nm) from a 2.0 cm distance for another 10 hours. The reaction mixture was directly loaded onto a short *silica gel* column, followed by gradient elution with petroleum ether/dichloromethane (5/1–2/1 ratio). Removing the solvent in *vacuo*, afforded products **2a** in 78% yield with 84% ee.

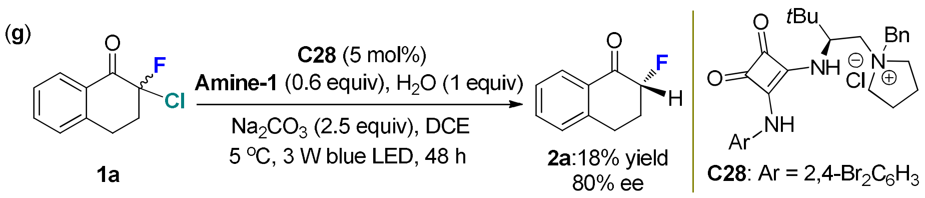

Na_2CO_3 (26.5 mg, 0.25 mmol, 2.5 equiv), **Amine-1** (9.72 mg, 0.06 mmol, 0.6 equiv), **2a** (96% ee, 16.4 mg, 0.1 mmol, 1.0 equiv), H_2O (1.8 μL, 0.1 mmol, 1.0 equiv), stir bar and DCE (1.0 mL) were added to a 10 mL Schlenk tube sequentially and then degassed for three times by freeze-pump-thaw method. The reaction mixture was stirred under an argon atmosphere at 5 °C for 30 min without light, then irradiated by a 3 W blue LED (λ = 450−455 nm) from a 2.0 cm distance for another 10 hours. The reaction mixture was directly loaded onto a short *silica gel* column, followed by gradient elution with petroleum ether/dichloromethane (5/1–2/1 ratio). Removing the solvent in *vacuo*, afforded products **2a** in 18% yield with 80% ee.

5. Proposed mechanism

Proposed reaction process:

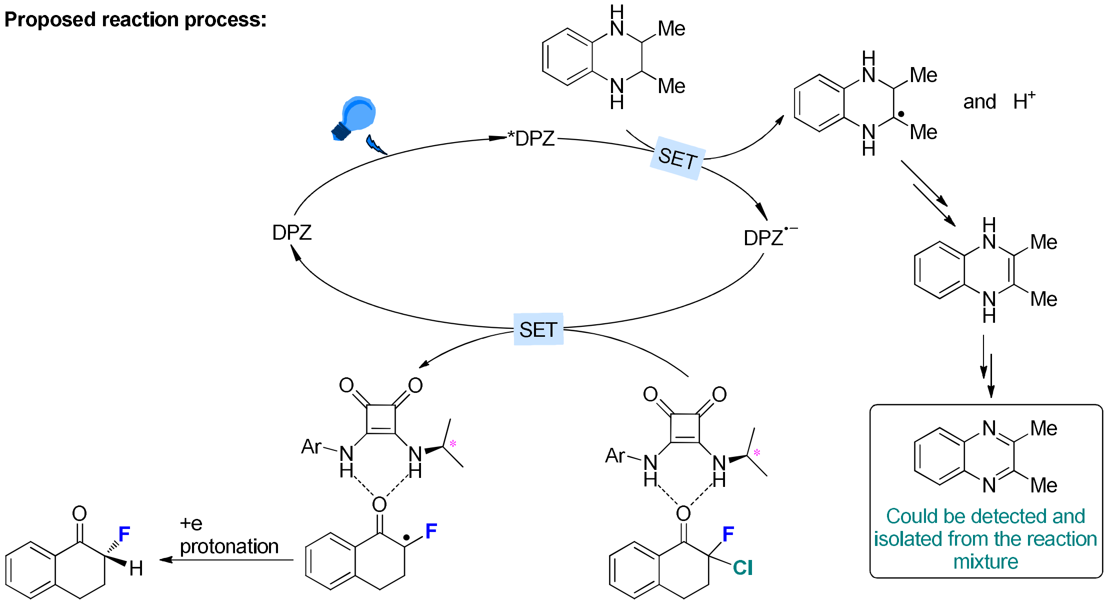

Disfavoured and favoured transition states:

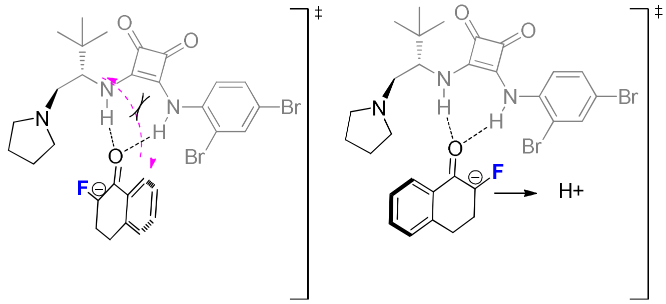

Fig. S22 Plausible reaction process and transition states.

6. Determination of the absolute configurations

Absolute configurations of **2** are determined by *X*-ray structure analysis of the product **2d.**

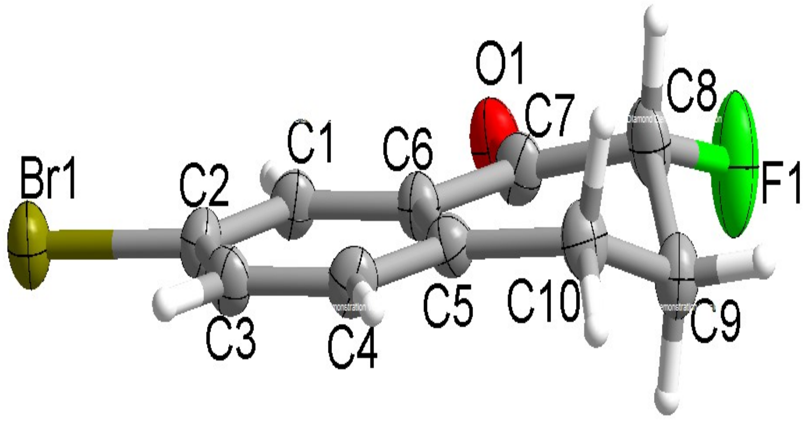

Fig. S21 Absolute configuration of **2d** (CCDC 1590512).

Displacement ellipsoids are drawn at the 30% probability level. (Solvent: dichloromethane)

Table S4 Crystal data and structure refinement.

Identification code	HMT70074
Empirical formula	$C_{10}H_8BrFO$
Formula weight	243.07
Temperature/K	293(2)
Crystal system	monoclinic
Space group	$P2_1$
a/Å	7.8209(6)
b/Å	6.9066(8)
c/Å	8.6288(6)
α/°	90
β/°	100.372(7)
γ/°	90
Volume/Å^3	458.47(7)
Z	2
ρ_{calc}g/cm^3	1.761
μ/mm^{-1}	5.892
F(000)	240.0
Crystal size/mm^3	0.23 × 0.13 × 0.08
Radiation	CuKα (λ = 1.54184)
2Θ range for data collection/°	10.422 to 134.124
Index ranges	$-9 \leq h \leq 8, -7 \leq k \leq 8, -10 \leq l \leq 10$
Reflections collected	3410
Independent reflections	1530 [R_{int} = 0.0269, R_{sigma} = 0.0345]

Data/restraints/parameters	1530/1/118
Goodness-of-fit on F^2	1.064
Final R indexes [I>=2σ (I)]	R_1 = 0.0342, wR_2 = 0.0855
Final R indexes [all data]	R_1 = 0.0410, wR_2 = 0.0922
Largest diff. peak/hole / e Å^{-3}	0.43/-0.35
Flack parameter	-0.04(6)

Table S5 Fractional Atomic Coordinates (×10^4) and Equivalent Isotropic Displacement Parameters (Å^2×10^3). U_{eq} is defined as 1/3 of of the trace of the orthogonalised U_{IJ} tensor.

Atom	*x*	*y*	*z*	U(eq)
Br1	8986.6(7)	4963.8(2)	6006.1(7)	66.0(3)
C1	6489(7)	4910(30)	3177(6)	51.0(16)
C2	6706(6)	4950(30)	4785(6)	47.7(11)
C3	5288(7)	4980(30)	5553(6)	52.8(13)
C4	3654(7)	5010(30)	4661(6)	51.8(13)
C5	3375(6)	4990(30)	3020(6)	47.7(11)
C6	4830(6)	4940(30)	2284(5)	45.2(11)
C7	4619(7)	4950(30)	530(6)	55.1(15)
C8	2784(9)	4640(30)	-327(7)	67(5)
C9	1487(9)	5749(13)	420(8)	63(2)
C10	1564(6)	4940(30)	2080(6)	57.8(14)
F1	2697(6)	5260(30)	-1862(4)	121(4)
O1	5835(5)	5010(30)	-146(4)	68.9(13)

Table S6 Anisotropic Displacement Parameters (Å^2×10^3). The Anisotropic displacement factor exponent takes the form: $-2\pi^2[h^2a^{*2}U_{11}+2hka^*b^*U_{12}+\ldots]$.

Atom	U_{11}	U_{22}	U_{33}	U_{23}	U_{13}	U_{12}
Br1	49.8(3)	93.0(5)	51.7(3)	-1.9(9)	-0.6(2)	1.3(10)
C1	49(3)	65(4)	42(2)	-7(7)	14(2)	14(8)
C2	43(2)	58(3)	42(2)	6(8)	5(2)	4(9)
C3	56(3)	65(4)	37(2)	-8(8)	10(2)	6(10)
C4	51(3)	66(4)	44(2)	-1(8)	21(2)	9(8)
C5	47(2)	53(3)	45(2)	-6(8)	13(2)	1(9)
C6	46(2)	55(3)	36(2)	1(8)	11.0(19)	0(9)
C7	60(3)	71(4)	37(2)	-5(9)	16(2)	13(9)
C8	62(4)	100(14)	37(3)	-8(5)	4(3)	-2(5)
C9	51(4)	85(6)	50(4)	6(3)	2(3)	2(3)
C10	44(3)	77(4)	54(3)	0(11)	12(2)	-8(10)
F1	77(3)	242(11)	41.6(19)	30(6)	5.9(19)	0(7)
O1	63(2)	107(4)	41.0(18)	-10(8)	20.2(18)	-5(8)

Table S7 Bond Lengths.

Atom	Atom	Length/Å	Atom	Atom	Length/Å
Br1	C2	1.901(5)	C5	C10	1.501(7)
C1	C2	1.367(7)	C6	C7	1.492(6)
C1	C6	1.385(7)	C7	C8	1.506(11)
C2	C3	1.390(7)	C7	O1	1.203(7)
C3	C4	1.368(7)	C8	C9	1.504(15)
C4	C5	1.393(7)	C8	F1	1.380(12)
C5	C6	1.400(7)	C9	C10	1.530(12)

Table S8 Bond Angles.

Atom	Atom	Atom	Angle/°	Atom	Atom	Atom	Angle/°
C2	C1	C6	119.9(4)	C1	C6	C7	119.1(4)
C1	C2	Br1	119.7(4)	C5	C6	C7	120.6(5)
C1	C2	C3	121.3(4)	C6	C7	C8	114.8(6)
C3	C2	Br1	119.0(4)	O1	C7	C6	122.6(5)
C4	C3	C2	118.5(4)	O1	C7	C8	122.3(5)
C3	C4	C5	122.1(4)	C9	C8	C7	112.1(12)
C4	C5	C6	118.0(5)	F1	C8	C7	107.9(9)
C4	C5	C10	120.6(4)	F1	C8	C9	109.6(14)
C6	C5	C10	121.3(4)	C8	C9	C10	107.4(9)
C1	C6	C5	120.3(4)	C5	C10	C9	111.8(7)

Table S9 Torsion Angles.

A	B	C	D	Angle/°	A	B	C	D	Angle/°
Br1	C2	C3	C4	178.8(15)	C5	C6	C7	O1	176(2)
C1	C2	C3	C4	-1(3)	C6	C1	C2	Br1	-178.7(18)
C1	C6	C7	C8	170(2)	C6	C1	C2	C3	1(3)
C1	C6	C7	O1	-3(4)	C6	C5	C10	C9	-26(3)
C2	C1	C6	C5	-1(3)	C6	C7	C8	C9	41(3)
C2	C1	C6	C7	179(2)	C6	C7	C8	F1	162.1(19)
C2	C3	C4	C5	1(4)	C7	C8	C9	C10	-63.5(17)
C3	C4	C5	C6	0(3)	C8	C9	C10	C5	54.8(18)
C3	C4	C5	C10	177(2)	C10	C5	C6	C1	-177(2)
C4	C5	C6	C1	0(3)	C10	C5	C6	C7	3(3)
C4	C5	C6	C7	-179(3)	F1	C8	C9	C10	176.8(14)
C4	C5	C10	C9	156.1(19)	O1	C7	C8	C9	-144.9(18)
C5	C6	C7	C8	-10(3)	O1	C7	C8	F1	-24(3)

Table S10 Hydrogen Atom Coordinates (Å×10^4) and Isotropic Displacement Parameters

(Å²×10³).

**Atom**	***x***	***y***	***z***	**U(eq)**
H1	7452	4868	2685	61
H3	5447	4983	6647	63
H4	2700	5048	5166	62
H8	2505	3261	-326	80
H9A	328	5594	-194	76
H9B	1772	7117	470	76
H10A	801	5684	2620	69
H10B	1150	3609	2007	69

Experimental

The crystal was kept at 293(2) K during data collection. Using Olex2, the structure was solved with the ShelXS structure solution program using Patterson Method and refined with the ShelXL [3] refinement package using Least Squares minimisation.

Crystal structure determination

Crystal Data for $C_{10}H_8BrFO$ (M =243.07 g/mol): monoclinic, space group $P2_1$ (no. 4), a = 7.8209(6) Å, b = 6.9066(8) Å, c = 8.6288(6) Å, β = 100.372(7)°, V = 458.47(7) Å^3, Z = 2, T = 293(2) K, μ(CuKα) = 5.892 mm^{-1}, $Dcalc$ = 1.761 g/cm^3, 3410 reflections measured (10.422° ≤ 2Θ ≤ 134.124°), 1530 unique (R_{int} = 0.0269, R_{sigma} = 0.0345) which were used in all calculations. The final R_1 was 0.0342 (I > 2σ(I)) and wR_2 was 0.0922 (all data).

Refinement model description

Number of restraints - 1, number of constraints - unknown.
Details:
1. Fixed Uiso
At 1.2 times of:
All C(H) groups, All C(H,H) groups
2.a Ternary CH refined with riding coordinates:
C8(H8)
2.b Secondary CH2 refined with riding coordinates:
C9(H9A,H9B), C10(H10A,H10B)
2.c Aromatic/amide H refined with riding coordinates:
C1(H1), C3(H3), C4(H4)

2) Absolute configurations of **6** and **8** are determined by *X*-ray structure analysis of the product **6f**

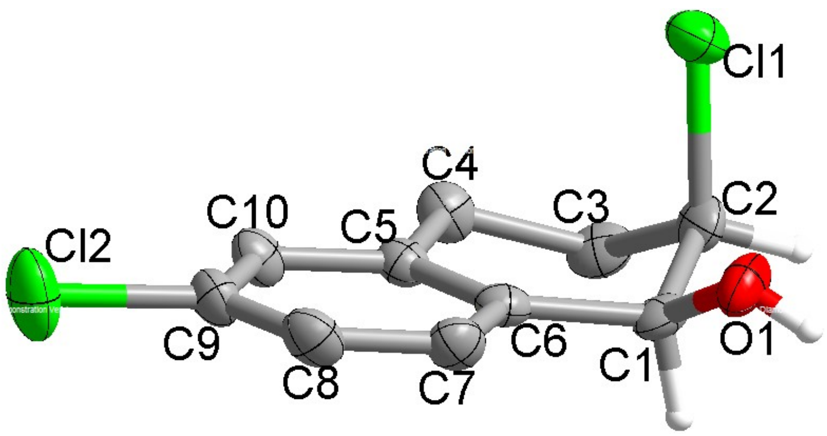

Fig. S22 Absolute configuration of **6f** (CCDC 1840318).

Table S11 Crystal data and structure refinement.

Identification code	HM23026
Empirical formula	$C_{10}H_{10}Cl_2O$
Formula weight	217.08
Temperature/K	293(2)
Crystal system	orthorhombic
Space group	$P2_12_12_1$
a/Å	5.04728(18)
b/Å	19.5159(6)
c/Å	20.2528(8)
α/°	90
β/°	90
γ/°	90
Volume/Å^3	1994.94(12)
Z	8
ρ_{calc}g/cm^3	1.446
μ/mm^{-1}	5.490
F(000)	896.0
Crystal size/mm^3	0.16 × 0.11 × 0.1
Radiation	CuKα (λ = 1.54184)
2Θ range for data collection/°	8.732 to 134.138
Index ranges	$-6 \leq h \leq 4$, $-23 \leq k \leq 22$, $-24 \leq l \leq 23$
Reflections collected	8713
Independent reflections	3534 [R_{int} = 0.0378, R_{sigma} = 0.0418]
Data/restraints/parameters	3534/0/243
Goodness-of-fit on F^2	1.032
Final R indexes [I>=2σ (I)]	R_1 = 0.0464, wR_2 = 0.1189
Final R indexes [all data]	R_1 = 0.0553, wR_2 = 0.1265
Largest diff. peak/hole / e Å^{-3}	0.38/-0.17

Flack parameter -0.014(19)

Table S12 Fractional Atomic Coordinates (×10[4]) and Equivalent Isotropic Displacement Parameters (Å[2]×10[3]). U_{eq} is defined as 1/3 of of the trace of the orthogonalised U_{IJ} tensor.

Atom	*x*	*y*	*z*	U(eq)
C1	4524(9)	6731(2)	7674(2)	47.7(10)
C2	4476(10)	6342(2)	7032(3)	53.2(11)
C3	4213(10)	6830(3)	6451(3)	55.9(12)
C4	6554(11)	7322(2)	6413(2)	51.8(12)
C5	7370(10)	7602.9(19)	7081(2)	42.4(9)
C6	6518(9)	7310(2)	7673(2)	43.0(10)
C7	7456(12)	7577(2)	8270(2)	52.5(11)
C8	9208(12)	8115(2)	8285(3)	58.1(13)
C9	9983(11)	8402(2)	7697(3)	54.0(12)
C10	9145(11)	8152(2)	7100(2)	50.7(12)
Cl1	7467(3)	5836.2(6)	6946.7(7)	65.1(3)
Cl2	12146(4)	9103.6(7)	7711.0(8)	88.9(6)
O1	5028(8)	6297(2)	8222(2)	59.8(10)
C1'	5328(10)	4323(3)	4308(3)	51.7(11)
C2'	5348(10)	3782(3)	4849(3)	57.0(13)
C3'	5594(11)	4117(3)	5515(3)	69.2(16)
C4'	3414(12)	4631(3)	5660(3)	65.0(15)
C5'	2453(12)	5030(2)	5072(2)	50.1(11)
C6'	3274(9)	4873(2)	4428(2)	47.4(11)
C7'	2238(12)	5243(2)	3900(2)	52.7(11)
C8'	414(12)	5757(2)	3999(2)	57.5(13)
C9'	-387(11)	5906(2)	4629(3)	56.0(12)
C10'	620(11)	5550(2)	5166(2)	54.8(13)
Cl1'	2346(3)	3271.8(6)	4820.3(7)	62.6(3)
Cl2'	-2718(4)	6557.0(7)	4756.7(8)	80.5(5)
O1'	4947(8)	4041(2)	3666.1(19)	58.4(9)

Table S13 Anisotropic Displacement Parameters (Å[2]×10[3]). The Anisotropic displacement factor exponent takes the form: $-2\pi^2[h^2a^{*2}U_{11}+2hka^*b^*U_{12}+\ldots]$.

Atom	U_{11}	U_{22}	U_{33}	U_{23}	U_{13}	U_{12}
C1	28(2)	61(2)	54(3)	5(2)	4(2)	5(2)
C2	32(2)	56(2)	71(3)	-3(2)	-4(2)	-6(2)
C3	38(3)	76(3)	54(3)	-5(2)	-12(2)	-2(2)
C4	60(3)	55(2)	40(2)	3.2(19)	-8(2)	-1(2)
C5	43(2)	41.4(19)	42.8(19)	-1.0(15)	-6(2)	5(2)

C6	39(2)	43(2)	47(2)	1.1(18)	0.2(19)	10.2(18)
C7	58(3)	57(2)	43(2)	-2.3(18)	9(3)	5(3)
C8	74(4)	53(3)	48(3)	-11(2)	-3(3)	2(3)
C9	60(3)	40(2)	62(3)	-4(2)	-4(3)	-6(2)
C10	65(3)	41(2)	46(2)	4.6(18)	-2(2)	0(2)
Cl1	63.0(8)	51.6(5)	80.7(8)	-7.7(5)	3.8(8)	10.5(7)
Cl2	120.9(15)	65.4(8)	80.3(9)	-5.5(6)	-9.9(11)	-41.3(10)
O1	40(2)	73(2)	67(2)	24.1(19)	6.0(18)	-4.7(18)
C1'	29(2)	71(3)	55(3)	-10(2)	0(2)	-14(2)
C2'	29(2)	71(3)	70(3)	2(3)	-7(2)	-2(2)
C3'	46(3)	99(4)	63(3)	11(3)	-16(3)	-14(3)
C4'	68(4)	82(3)	45(3)	1(2)	-7(3)	-14(3)
C5'	51(3)	56(2)	43(2)	-5.3(17)	-4(3)	-17(3)
C6'	41(3)	53(2)	48(2)	-4.4(19)	3(2)	-17.8(19)
C7'	59(3)	57(2)	42(2)	-4.3(18)	9(3)	-12(3)
C8'	65(3)	54(3)	54(3)	6(2)	4(3)	-6(3)
C9'	55(3)	53(2)	59(3)	-4(2)	9(2)	-9(2)
C10'	60(3)	61(3)	43(2)	-10(2)	10(2)	-18(3)
Cl1'	48.7(7)	63.0(6)	75.9(8)	6.5(5)	-5.8(7)	-11.1(6)
Cl2'	86.7(11)	70.6(8)	84.1(9)	-3.7(6)	22.0(10)	10.7(8)
O1'	40.4(19)	78(2)	57(2)	-20.1(18)	5.6(17)	-5.8(18)

Table S14 Bond Lengths.

Atom	Atom	Length/Å	Atom	Atom	Length/Å
C1	C2	1.506(7)	C1'	C2'	1.521(7)
C1	C6	1.513(6)	C1'	C6'	1.512(7)
C1	O1	1.420(6)	C1'	O1'	1.425(6)
C2	C3	1.519(7)	C2'	C3'	1.505(8)
C2	Cl1	1.812(5)	C2'	Cl1'	1.814(5)
C3	C4	1.524(7)	C3'	C4'	1.517(9)
C4	C5	1.517(6)	C4'	C5'	1.505(7)
C5	C6	1.395(6)	C5'	C6'	1.402(7)
C5	C10	1.397(7)	C5'	C10'	1.386(8)
C6	C7	1.400(6)	C6'	C7'	1.392(7)
C7	C8	1.373(7)	C7'	C8'	1.375(8)
C8	C9	1.372(7)	C8'	C9'	1.371(7)
C9	C10	1.371(7)	C9'	C10'	1.387(8)
C9	Cl2	1.751(5)	C9'	Cl2'	1.750(6)

Table S15 Bond Angles.

Atom	Atom	Atom	Angle/°	Atom	Atom	Atom	Angle/°
C2	C1	C6	112.7(4)	C6'	C1'	C2'	112.5(4)
O1	C1	C2	112.2(4)	O1'	C1'	C2'	112.9(4)
O1	C1	C6	109.2(4)	O1'	C1'	C6'	109.2(4)
C1	C2	C3	110.7(4)	C1'	C2'	Cl1'	110.6(3)
C1	C2	Cl1	110.1(4)	C3'	C2'	C1'	110.2(5)
C3	C2	Cl1	109.9(4)	C3'	C2'	Cl1'	109.6(4)
C2	C3	C4	111.5(4)	C2'	C3'	C4'	113.6(5)
C5	C4	C3	113.1(4)	C5'	C4'	C3'	115.0(5)
C6	C5	C4	122.3(4)	C6'	C5'	C4'	121.9(5)
C6	C5	C10	119.2(4)	C10'	C5'	C4'	119.0(4)
C10	C5	C4	118.4(4)	C10'	C5'	C6'	119.0(4)
C5	C6	C1	120.8(4)	C5'	C6'	C1'	120.5(5)
C5	C6	C7	119.1(4)	C7'	C6'	C1'	120.2(4)
C7	C6	C1	120.1(4)	C7'	C6'	C5'	119.3(5)
C8	C7	C6	121.4(4)	C8'	C7'	C6'	121.2(4)
C9	C8	C7	118.5(5)	C9'	C8'	C7'	119.2(5)
C8	C9	Cl2	118.9(4)	C8'	C9'	C10'	121.0(5)
C10	C9	C8	122.1(5)	C8'	C9'	Cl2'	119.4(4)
C10	C9	Cl2	119.0(4)	C10'	C9'	Cl2'	119.7(4)
C9	C10	C5	119.7(4)	C5'	C10'	C9'	120.3(4)

Table S16 Hydrogen Bonds.

D	H	A	d(D-H)/Å	d(H-A)/Å	d(D-A)/Å	D-H-A/°
O1	H1A	O1'[1]	0.71(7)	2.05(7)	2.747(6)	165(7)
O1'	H1'A	O1[2]	0.76(9)	2.03(9)	2.771(6)	163(9)

[1]1/2-X,1-Y,1/2+Z; [2]3/2-X,1-Y,-1/2+Z

Table S17 Torsion Angles.

A	B	C	D	Angle/°	A	B	C	D	Angle/°
C1	C2	C3	C4	61.4(6)	C1'	C2'	C3'	C4'	57.5(6)
C1	C6	C7	C8	-178.1(4)	C1'	C6'	C7'	C8'	-178.5(4)
C2	C1	C6	C5	23.3(6)	C2'	C1'	C6'	C5'	27.0(6)
C2	C1	C6	C7	-159.2(4)	C2'	C1'	C6'	C7'	-154.9(4)
C2	C3	C4	C5	-42.4(6)	C2'	C3'	C4'	C5'	-34.8(7)
C3	C4	C5	C6	15.2(7)	C3'	C4'	C5'	C6'	7.9(7)
C3	C4	C5	C10	-168.2(4)	C3'	C4'	C5'	C10'	-174.7(5)
C4	C5	C6	C1	-5.5(7)	C4'	C5'	C6'	C1'	-4.4(7)
C4	C5	C6	C7	176.9(4)	C4'	C5'	C6'	C7'	177.5(5)
C4	C5	C10	C9	-178.0(5)	C4'	C5'	C10'	C9'	-177.2(5)

C5	C6	C7	C8	-0.6(7)	C5'	C6'	C7'	C8'	-0.3(8)
C6	C1	C2	C3	-50.6(5)	C6'	C1'	C2'	C3'	-52.6(6)
C6	C1	C2	Cl1	71.2(4)	C6'	C1'	C2'	Cl1'	68.8(5)
C6	C5	C10	C9	-1.2(7)	C6'	C5'	C10'	C9'	0.2(8)
C6	C7	C8	C9	1.6(8)	C6'	C7'	C8'	C9'	0.2(8)
C7	C8	C9	C10	-2.6(8)	C7'	C8'	C9'	C10'	0.2(8)
C7	C8	C9	Cl2	178.3(4)	C7'	C8'	C9'	Cl2'	-179.7(4)
C8	C9	C10	C5	2.4(8)	C8'	C9'	C10'	C5'	-0.4(8)
C10	C5	C6	C1	177.9(4)	C10'	C5'	C6'	C1'	178.2(4)
C10	C5	C6	C7	0.3(7)	C10'	C5'	C6'	C7'	0.1(7)
Cl1	C2	C3	C4	-60.4(5)	Cl1'	C2'	C3'	C4'	-64.5(6)
Cl2	C9	C10	C5	-178.5(4)	Cl2'	C9'	C10'	C5'	179.5(4)
O1	C1	C2	C3	-174.2(4)	O1'	C1'	C2'	C3'	-176.6(4)
O1	C1	C2	Cl1	-52.5(5)	O1'	C1'	C2'	Cl1'	-55.2(5)
O1	C1	C6	C5	148.6(4)	O1'	C1'	C6'	C5'	153.2(4)
O1	C1	C6	C7	-33.8(6)	O1'	C1'	C6'	C7'	-28.7(6)

Table S18 Hydrogen Atom Coordinates (Å×10^4) and Isotropic Displacement Parameters (Å^2×10^3).

Atom	*x*	*y*	*z*	U(eq)
H1	2765	6934	7737	57
H2	2947	6033	7033	64
H3A	2586	7091	6495	67
H3B	4109	6569	6045	67
H4A	8051	7086	6217	62
H4B	6091	7702	6126	62
H7	6881	7386	8665	63
H8	9855	8281	8684	70
H10	9755	8346	6709	61
H1A	3870(130)	6190(30)	8400(30)	60(19)
H1'	7068	4546	4311	62
H2'	6875	3480	4781	68
H3'A	7292	4349	5541	83
H3'B	5570	3765	5853	83
H4'A	1921	4388	5851	78
H4'B	4053	4951	5990	78
H7'	2788	5141	3473	63
H8'	-268	5999	3642	69
H10'	64	5660	5590	66
H1'A	6180(180)	3880(40)	3510(40)	120(30)

Experimental

The crystal was kept at 293(2) K during data collection. Using Olex2, the structure was solved with the ShelXS structure solution program using Direct Methods and refined with the ShelXL refinement package using Least Squares minimisation.

Crystal structure determination

Crystal Data for $C_{10}H_{10}Cl_2O$ (M =217.08 g/mol): orthorhombic, space group $P2_12_12_1$ (no. 19), a = 5.04728(18) Å, b = 19.5159(6) Å, c = 20.2528(8) Å, V = 1994.94(12) Å^3, Z = 8, T = 293(2) K, μ(CuKα) = 5.490 mm^{-1}, $Dcalc$ = 1.446 g/cm^3, 8713 reflections measured (8.732° ≤ 2Θ ≤ 134.138°), 3534 unique (R_{int} = 0.0378, R_{sigma} = 0.0418) which were used in all calculations. The final R_1 was 0.0464 (I > 2σ(I)) and wR_2 was 0.1265 (all data).

Refinement model description

Number of restraints - 0, number of constraints - unknown.

Details:

1. Fixed Uiso

At 1.2 times of:

All C(H) groups, All C(H,H) groups

2.a Ternary CH refined with riding coordinates:

C1(H1), C2(H2), C1'(H1'), C2'(H2')

2.b Secondary CH2 refined with riding coordinates:

C3(H3A,H3B), C4(H4A,H4B), C3'(H3'A,H3'B), C4'(H4'A,H4'B)

2.c Aromatic/amide H refined with riding coordinates:

C7(H7), C8(H8), C10(H10), C7'(H7'), C8'(H8'), C10'(H10')

7. References

1. K. Shibatomi, H. Yamamoto, *Angew. Chem. Int. Ed.,* 2008, **47**, 5796.

2. J. D. Hamel, M. Cloutier, J. F. Paquin, *Org. Lett.,* 2016, **18**, 1852.

3. B. Šket, N. Zupančič, M. Zupan, *J. Fluorine Chem.,* 1989, **45**, 313.

4. T. Maji, A. Karmakar, O. Reiser, *J. Org. Chem.,* 2010, **76**, 736.

5. S. Liu, Y. Zhou, Y. Sui, H. Liu, H. Zhou, *Org. Chem. Front.,* 2017, **4**, 2175.

8. Characterization of products

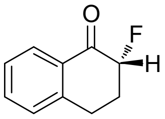

2a: white solid; Mp 44.6–46.0 °C; 15.1 mg, 92% yield; 96% ee (On a 1.0 mmol scale: 141.0 mg, 86% yield; 97% ee); $[\alpha]_D^{22}$ +157.1 (*c* 1.0, $CHCl_3$); ^{1}H NMR (300 MHz, $CDCl_3$) δ 7.99 (d, *J* = 7.8 Hz, 1H), 7.46 (t, *J* = 7.5 Hz, 1H), 7.30 (d, *J* = 7.6 Hz, 1H), 7.21 (d, *J* = 5.8 Hz, 1H), 5.08 (ddd, *J* = 47.9, 12.7, 5.2 Hz, 1H), 3.07 (dd, *J* = 9.1, 3.8 Hz, 2H), 2.57–2.45 (m, 1H), 2.36–2.20 (m, 1H); ^{13}C NMR (75 MHz, $CDCl_3$) δ 193.3 (d, *J* = 14.7 Hz), 143.0 (d, *J* = 1.4 Hz), 134.1, 131.2, 128.6, 127.8 (d, *J* = 2.2 Hz), 127.1, 91.2 (d, *J* = 188.0 Hz), 30.1 (d, *J* = 19.1 Hz), 27.0 (d, *J* = 11.5 Hz); ^{19}F NMR (376 MHz, $CDCl_3$) δ −190.3; HRMS (ESI) m/z 187.0541 $(M+Na)^+$, calc. for $C_{10}H_9OFNa^+$ 187.0535.

The ee was determined by HPLC analysis: LUX CELLULOSE-3 (4.6 mm i.d. x 250 mm); Hexane/2-propanol = 95/5; flow rate 1.0 mL/min; 25 °C; 254 nm; retention time: 12.6 min (minor) and 14.5 min (major).

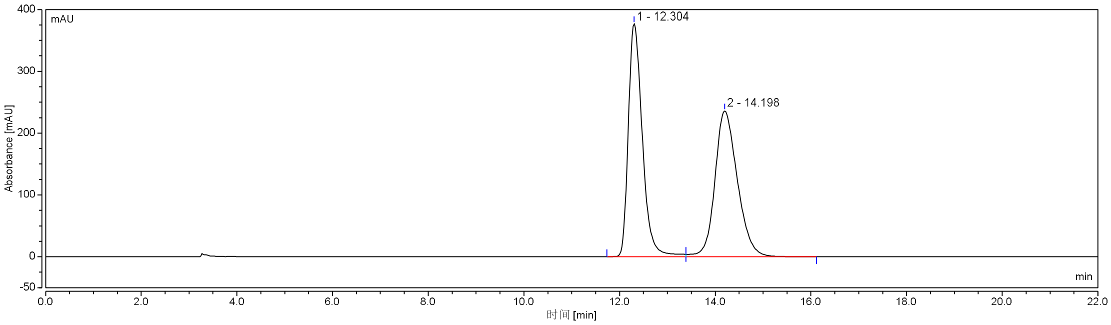

Entry	Retention Time	Area	Height	%Area
1	12.304	127.9981	376.82	49.98
2	14.198	128.1223	235.75	50.02

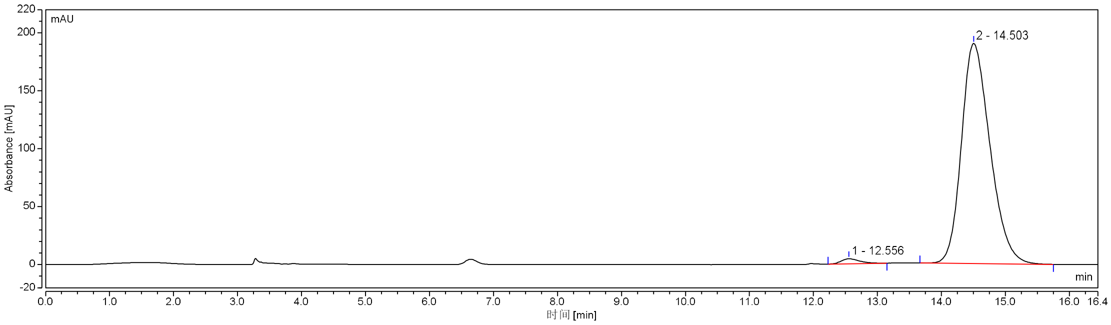

Entry	Retention Time	Area	Height	%Area
1	12.556	1.4671	4.39	1.47
2	14.503	98.3747	189.86	98.53

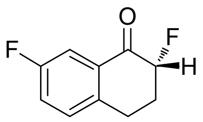

2b: yellow solid; Mp 119.8−121.0 ºC; 17.5 mg, 96% yield; 92% ee; $[\alpha]_D^{22}$ +70.0 (*c* 1.0, $CHCl_3$); ^1H NMR (300 MHz, $CDCl_3$) δ 7.70 (dd, *J* = 8.8, 2.0 Hz, 1H), 7.29–7.19 (m, 2H), 5.14 (ddd, *J* = 47.7, 12.6, 5.1 Hz, 1H), 3.10 (dd, *J* = 8.1, 3.6 Hz, 2H), 2.57 (qd, *J* = 9.3, 4.3 Hz, 1H), 2.43–2.27 (m, 1H); ^{13}C NMR (75 MHz, $CDCl_3$) δ 192.3 (dd, *J* = 15.0 Hz, 1.8 Hz), 161.7 (d, *J* = 247.2 Hz), 138.8 (dd, *J* = 3.0 Hz, 1.4 Hz), 132.8 (dd, *J* = 6.6 Hz, 1.2 Hz), 130.6 (d, *J* = 7.3 Hz), 121.6 (d, *J* = 22.4 Hz), 113.5 (dd, *J* = 22.2 Hz, 2.3 Hz), 90.9 (dd, *J* = 188.3 Hz, 1.1 Hz), 30.0 (d, *J* = 19.2 Hz), 26.3 (d, *J* = 11.5 Hz); ^{19}F NMR (376 MHz, $CDCl_3$) δ −114.1, −191.0; HRMS (ESI) m/z 205.0438 $(M+Na)^+$, calc. for $C_{10}H_8OF_2Na^+$ 205.0441.

The ee was determined by HPLC analysis: CHIRALPAK AS-H (4.6 mm i.d. x 250 mm); Hexane/2-propanol = 90/10; flow rate 1.0 mL/min; 25 ºC; 254 nm; retention time: 10.2 min (minor) and 14.1min (major).

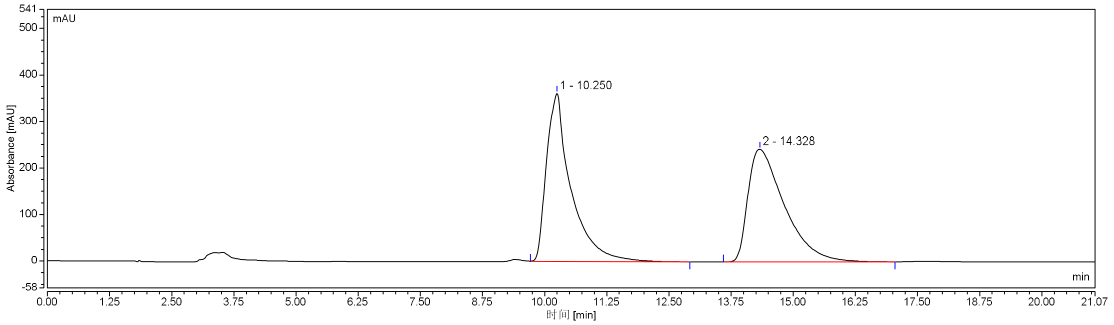

Entry	Retention Time	Area	Height	%Area
1	10.250	205.5293	361.14	49.91
2	14.328	206.2378	242.43	50.09

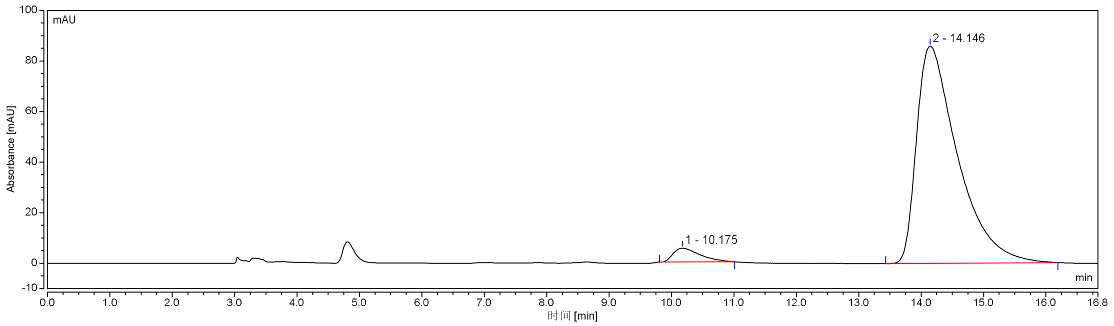

Entry	Retention Time	Area	Height	%Area
1	10.175	2.7524	5.49	4.04
2	14.146	65.2943	85.89	95.96

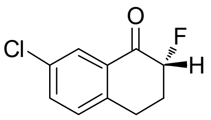

2c: white solid; Mp 119.8−121.5 ºC; 16.7 mg, 84% yield; 95% ee; $[\alpha]_D^{22}$ +27.1 (*c* 1.0, $CHCl_3$); ^1H NMR (300 MHz, $CDCl_3$) δ 8.07 (d, *J* = 2.1 Hz, 1H), 7.53 (dd, *J* = 8.2, 2.3 Hz, 1H), 7.30 (d, *J* = 5.7 Hz, 1H), 5.19 (ddd, *J* = 47.7, 12.6, 5.1 Hz, 1H), 3.15 (dd, *J* = 8.8, 4.2 Hz, 2H), 2.62 (qd, *J* = 9.1, 4.3 Hz, 1H), 2.48–2.32 (m, 1H); ^{13}C NMR (75 MHz, $CDCl_3$) δ 192.1 (d, *J* = 15.0 Hz), 141.2 (d, *J* = 1.3 Hz), 134.1, 133.5, 132.5 (d, *J* = 1.2 Hz), 130.2, 127.4 (d, *J* = 2.2 Hz), 90.8 (d, *J* = 188.7 Hz), 29.8 (d, *J* = 19.3 Hz,), 26.4 (d, *J* = 11.4 Hz); ^{19}F NMR (376 MHz, $CDCl_3$) δ −190.9; HRMS (ESI) m/z 221.0145 $(M+Na)^+$, calc. for $C_{10}H_8OFNaCl^+$ 221.0145.

The ee was determined by HPLC analysis: CHIRALPAK IC (4.6 mm i.d. x 250 mm); Hexane/2-propanol = 95/5; flow rate 1.0 mL/min; 25 ºC; 254 nm; retention time: 34.0min (minor) and 31.8 min (major).

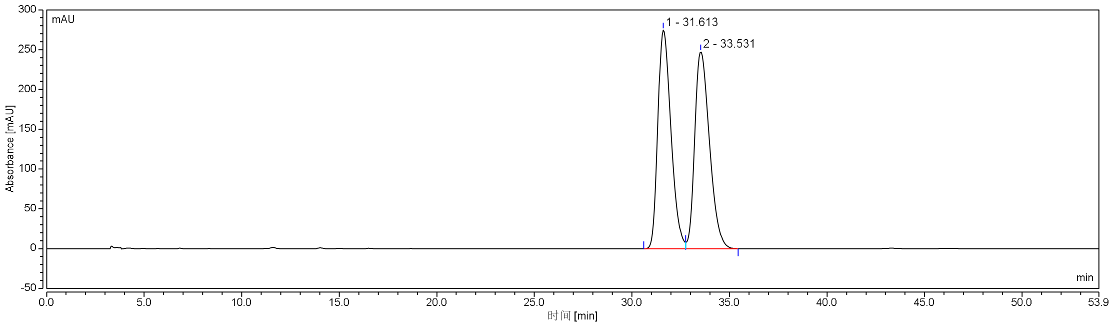

Entry	Retention Time	Area	Height	%Area
1	31.613	214.5172	274.63	49.79
2	33.531	216.3284	247.32	50.21

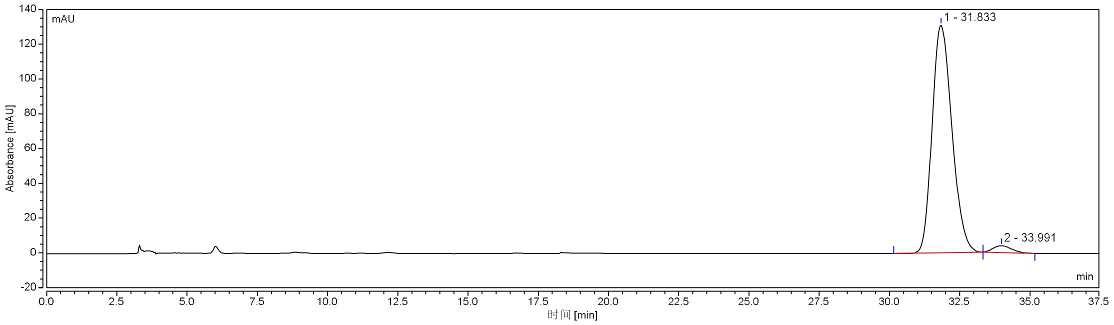

Entry	Retention Time	Area	Height	%Area
1	31.833	107.0252	130.86	97.28
2	33.991	2.9914	3.91	2.72

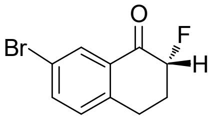

2d: white solid; Mp 122.1−123.8 ºC; 22.6 mg, 93% yield; 92% ee; $[\alpha]_D^{22}$ +234.1 (*c* 1.0, $CHCl_3$); ^1H NMR (300 MHz, $CDCl_3$) δ 8.16 (d, *J* = 1.7 Hz,

1H), 7.62 (dd, J = 8.2, 1.9 Hz, 1H), 7.16 (d, J = 8.2 Hz, 1H), 5.14 (ddd, J = 47.7, 12.7, 5.1 Hz, 1H), 3.07 (dd, J = 8.7, 4.0 Hz, 2H), 2.57 (qd, J = 9.3, 4.3 Hz, 1H), 2.41–2.25 (m, 1H); ^{13}C NMR (75 MHz, $CDCl_3$) δ 192.1 (d, J = 14.9 Hz), 141.6 (d, J = 1.4 Hz), 136.9, 132.7 (d, J = 1.1 Hz), 130.5, 121.2, 90.8 (d, J = 188.9 Hz), 29.8 (d, J = 19.3 Hz), 26.5 (d, J = 11.5 Hz); ^{19}F NMR (376 MHz, $CDCl_3$) δ −190.8; HRMS (ESI) m/z 264.9637 $(M+Na)^+$, calc. for $C_{10}H_8OFNaBr^+$ 264.9640.

The ee was determined by HPLC analysis: CHIRALPAK ID (4.6 mm i.d. x 250 mm); Hexane/2-propanol = 90/10; flow rate 1.0 mL/min; 25 °C; 254 nm; retention time: 21.5 min (minor) and 18.3 min (major).

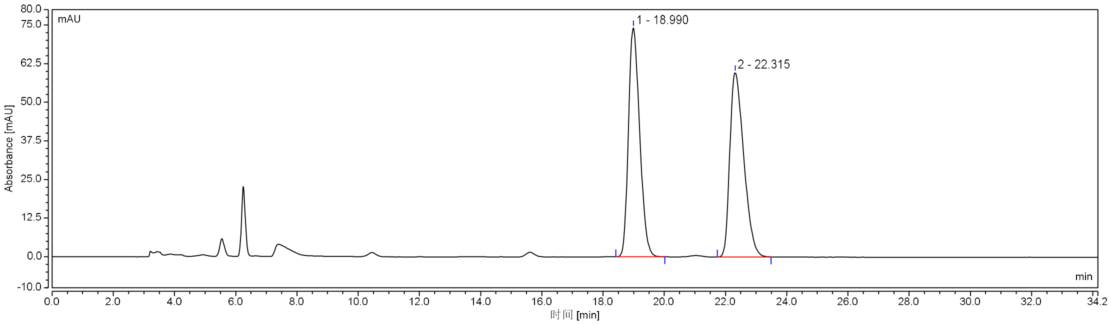

Entry	Retention Time	Area	Height	%Area
1	18.990	31.5624	74.07	50.07
2	22.315	31.4733	59.70	49.93

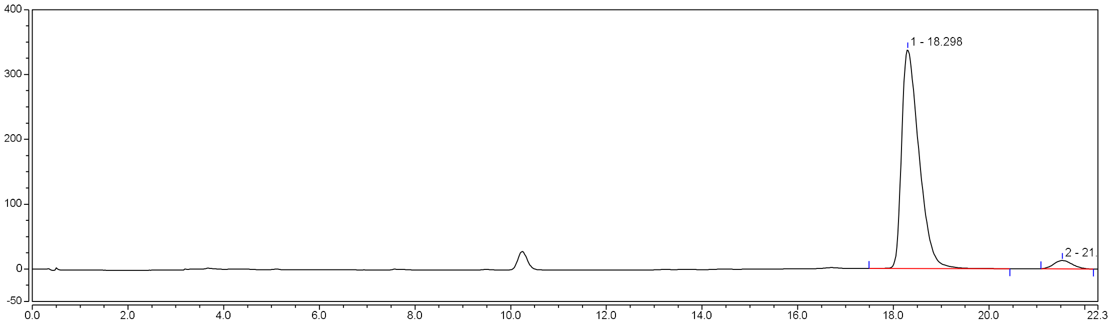

Entry	Retention Time	Area	Height	%Area
1	18.298	142.0097	337.21	96.09
2	21.531	5.7725	12.86	3.91

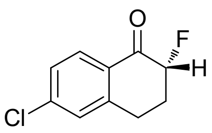

2e: white solid; Mp 106.9−108.8 °C; 15.9 mg, 80% yield; 95% ee; $[\alpha]_D^{22}$ +53.7 (c 1.0, $CHCl_3$); ^{1}H NMR (300 MHz, $CDCl_3$) δ 7.99 (d, J = 8.4 Hz, 1H), 7.32 (d, J = 8.4 Hz, 1H), 7.28 (s, 1H), 5.13 (ddd, J = 47.7, 12.6, 5.1 Hz, 1H), 3.10 (dd, J = 9.0, 4.0 Hz, 2H), 2.63–2.51 (m, 1H), 2.43–2.27 (m, 1H); ^{13}C NMR (75 MHz, $CDCl_3$) δ 192.2 (d, J = 14.9 Hz), 144.5 (d, J = 1.4 Hz), 140.6, 129.6 (d, J = 0.9 Hz),

129.4 (d, J = 2.2 Hz), 128.5, 127.7, 90.8 (d, J = 188.3 Hz), 29.8 (d, J = 19.4 Hz), 26.7 (d, J = 11.5 Hz); ^{19}F NMR (376 MHz, $CDCl_3$) δ −190.7; HRMS (ESI) m/z 199.0334 $(M+H)^+$, calc. for $C_{10}H_9OClF^+$ 199.0326.

The ee was determined by HPLC analysis: CHIRALPAK ID (4.6 mm i.d. x 250 mm); Hexane/2-propanol = 95/5; flow rate 1.0 mL/min; 25 °C; 254 nm; retention time: 17.1 min (minor) and 18.5 min (major).

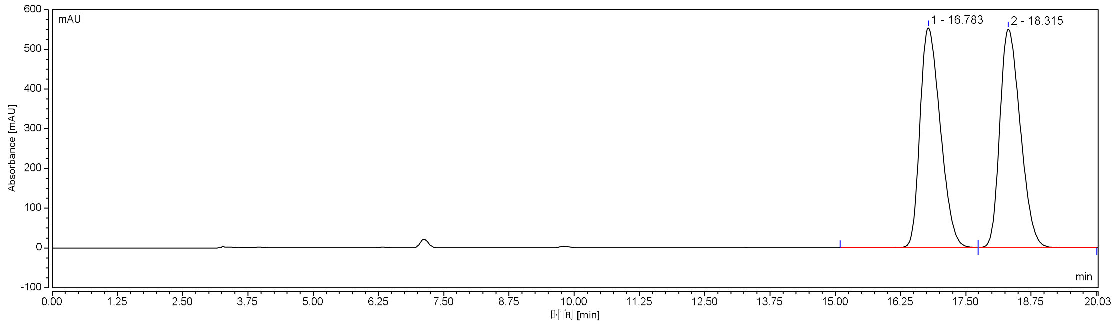

Entry	Retention Time	Area	Height	%Area
1	16.783	251.0569	553.69	49.95
2	18.315	251.5918	550.62	50.05

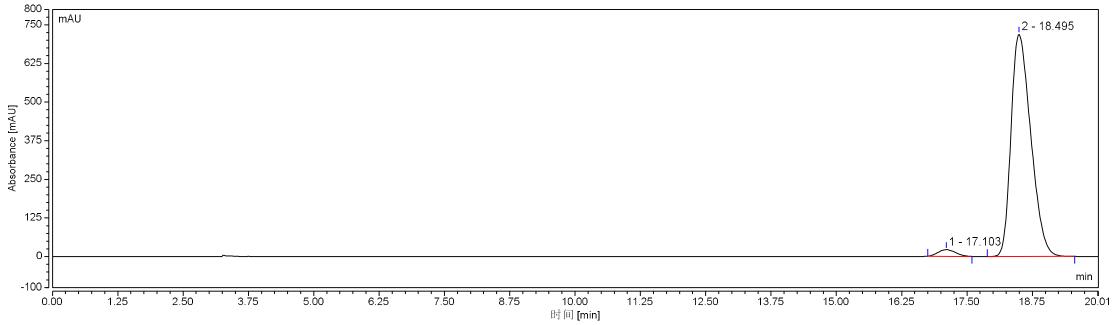

Entry	Retention Time	Area	Height	%Area
1	17.103	8.2298	21.37	2.54
2	18.495	315.2299	719.29	97.46

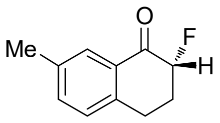

2f: white solid; Mp 51.7−53.4 °C; 13.5 mg, 76% yield; 94% ee; $[\alpha]_D^{22}$ +74.6 (c 1.0, $CHCl_3$); ^{1}H NMR (300 MHz, $CDCl_3$) δ 7.85(s, 1H), 7.33 (d, J = 7.8 Hz, 1H), 7.15 (d, J = 7.8 Hz, 1H), 5.13 (ddd, J = 47.9, 12.8, 5.2 Hz, 1H), 3.08 (d, J = 5.7 Hz, 2H), 2.61–2.50 (m, 1H), 2.36 (s, 3H), 2.35–2.23 (m, 1H); ^{13}C NMR (75 MHz, $CDCl_3$) δ 193.6 (d, J = 14.5 Hz), 140.1 (d, J = 1.4 Hz), 136.9, 135.2, 130.9 (d, J = 0.7 Hz), 128.5, 127.7 (d, J = 2.2 Hz), 91.3 (d, J = 187.9 Hz), 30.2 (d, J = 18.9 Hz), 26.6 (d, J = 11.7 Hz), 20.8; ^{19}F NMR (376 MHz, $CDCl_3$) δ −190.3; HRMS (ESI) m/z 201.0695 $(M+Na)^+$, calc. for $C_{11}H_{11}OFNa^+$ 201.0692.

The ee was determined by HPLC analysis: CHIRALPAK IC (4.6 mm i.d. x 250 mm); Hexane/2-propanol = 90/10; flow rate 1.0 mL/min; 25 °C; 254 nm; retention time: 32.3 min (minor) and 30.3min (major).

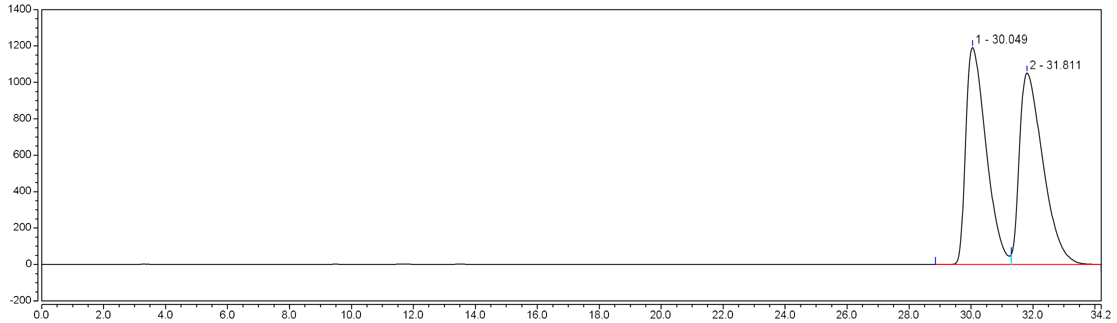

Entry	Retention Time	Area	Height	%Area
1	30.049	909.1999	1191.13	49.56
2	31.811	925.2250	1051.03	50.44

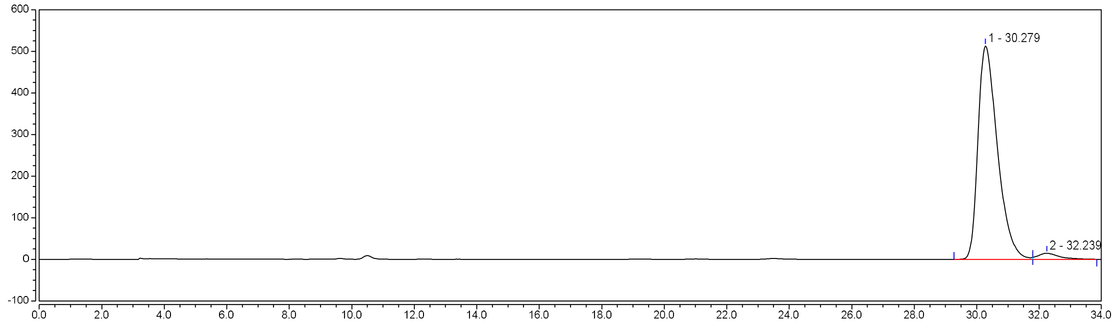

Entry	Retention Time	Area	Height	%Area
1	30.279	369.5150	512.59	97.05
2	32.239	11.2422	14.38	2.95

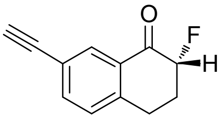

2g: yellow solid; Mp 162.8−163.4 °C; 15.2 mg, 81% yield; 94% ee; $[\alpha]_D^{22}$ +48.3 (*c* 1.0, $CHCl_3$); ^{1}H NMR (300 MHz, $CDCl_3$) δ 8.21 (s, 1H), 7.64 (d, *J* = 7.7 Hz, 1H), 7.29 (s, 1H), 5.18 (ddd, *J* = 47.7, 12.6, 5.0 Hz, 1H), 3.16 (d, *J* = 5.2 Hz, 2H), 3.14 (s, 1H), 2.66–2.57 (m, 1H), 2.47–2.31 (m, 1H); ^{13}C NMR (75 MHz, $CDCl_3$) δ 192.4 (d, *J* = 14.9 Hz), 143.3 (d, *J* = 1.2 Hz), 137.0, 131.4 (d, *J* = 2.2 Hz), 131.2, 128.8, 121.3, 90.9 (d, *J* = 188.6 Hz), 82.0, 78.3, 29.8 (d, *J* = 19.2 Hz), 26.9 (d, *J* = 11.5 Hz); ^{19}F NMR (376 MHz, $CDCl_3$) δ −190.6; HRMS (ESI) m/z 211.0539 $(M+Na)^+$, calc. for $C_{12}H_{11}OFNa^+$ 211.0535.

The ee was determined by HPLC analysis: CHIRALCEL OD-H (4.6 mm i.d. x 250 mm); Hexane/2-propanol = 90/10; flow rate 1.0 mL/min; 25 °C; 254 nm; retention time: 10.9 min (minor) and 12.4 min (major).

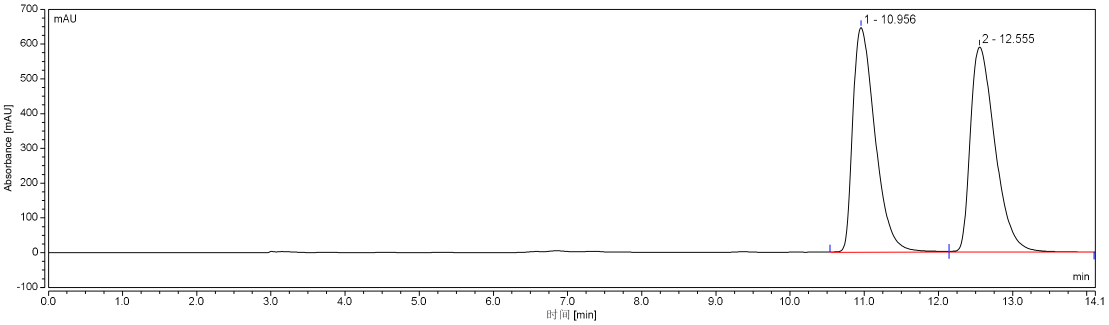

Entry	Retention Time	Area	Height	%Area
1	10.956	221.9637	644.15	49.83
2	12.555	223.4855	587.31	50.17

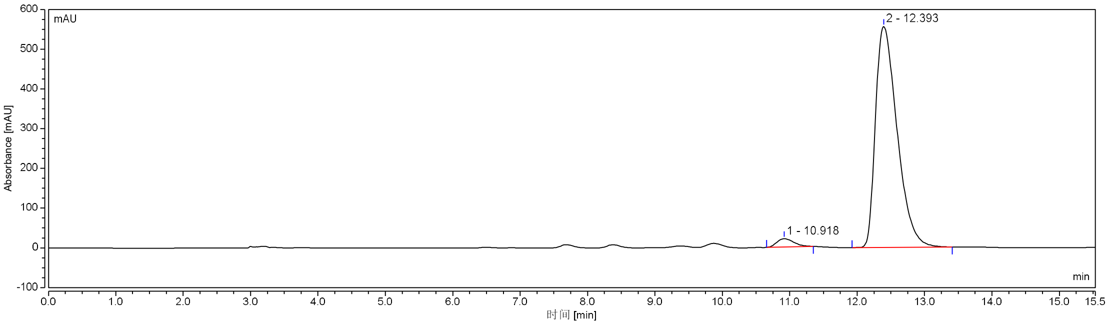

Entry	Retention Time	Area	Height	%Area
1	10.918	6.7516	21.82	3.17
2	12.393	206.3097	556.03	96.83

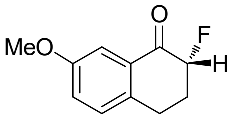

2h: white solid; Mp 87.2–88.7 °C; 15.2 mg, 86% yield; 94% ee; $[\alpha]_D^{22}$ +82.7 (*c* 1.0, $CHCl_3$); ^{1}H NMR (300 MHz, $CDCl_3$) δ 7.50 (d, *J* = 2.6 Hz, 1H), 7.17 (d, *J* = 8.5 Hz, 1H), 7.09 (dd, *J* = 8.5, 2.7 Hz, 1H), 5.13 (ddd, *J* = 47.9, 12.8, 5.2 Hz, 1H), 3.84 (s, 3H), 3.06 (dd, *J* = 9.3, 3.9 Hz, 2H), 2.61–2.50 (m, 1H), 2.40–2.24 (m, 1H); ^{13}C NMR (75 MHz, $CDCl_3$) δ 193.3 (d, *J* = 14.7 Hz), 158.6, 135.6 (d, *J* = 1.4 Hz), 132.0 (d, *J* = 1.0 Hz), 129.9, 122.7, 109.3 (d, *J* = 2.3 Hz), 91.3 (d, *J* = 187.8 Hz), 55.5, 30.3 (d, *J* = 19.0 Hz), 26.2 (d, *J* = 11.7 Hz); ^{19}F NMR (376 MHz, $CDCl_3$) δ −190.4; HRMS (ESI) m/z 217.0647 $(M+Na)^+$, calc. for $C_{11}H_{11}O_2FNa^+$ 217.0641.

The ee was determined by HPLC analysis: CHIRALPAK ID (4.6 mm i.d. x 250 mm); Hexane/2-propanol = 90/10; flow rate 1.0 mL/min; 25 °C; 254 nm; retention time: 16.6 min (minor) and 17.7 min (major).

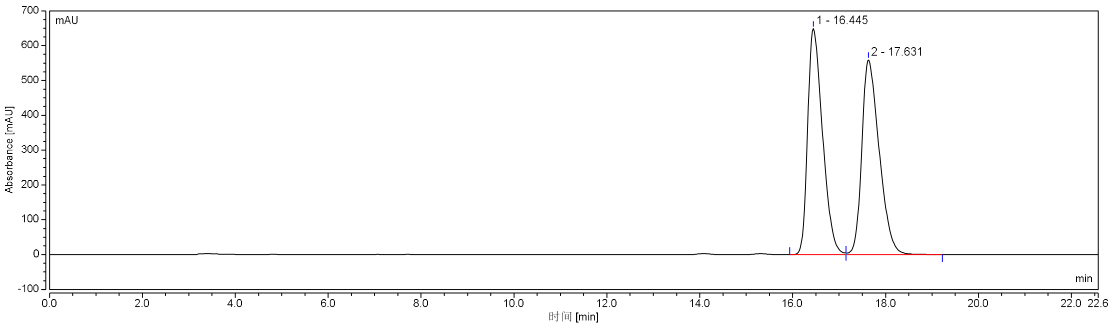

Entry	Retention Time	Area	Height	%Area
1	16.445	240.4402	649.34	49.77
2	17.631	242.6733	559.53	50.23

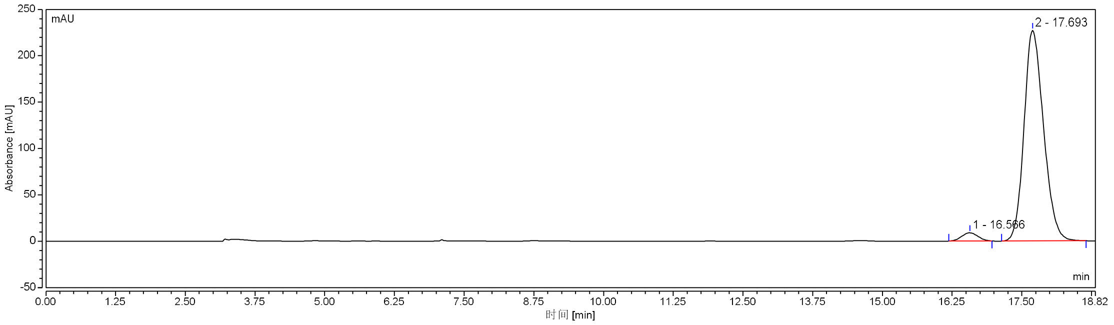

Entry	Retention Time	Area	Height	%Area
1	16.566	3.0162	8.94	3.17
2	17.693	92.2742	227.09	96.83

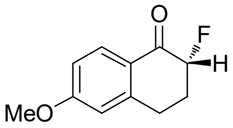

2i: white solid; Mp 113.4−115.0 °C; 13.2 mg, 68% yield; 97% ee; $[\alpha]_D^{22}$ +51.4 (*c* 1.0, $CHCl_3$); ^{1}H NMR (300 MHz, $CDCl_3$) δ 8.03 (d, *J* = 8.8 Hz, 1H), 6.86 (dd, *J* = 8.8, 2.4 Hz, 1H), 6.69 (d, *J* = 2.1 Hz, 1H), 5.09 (ddd, *J* = 47.9, 12.5, 5.1 Hz, 1H), 3.86 (s, 3H), 3.07 (dd, *J* = 9.1, 4.0 Hz, 2H), 2.59–2.47 (m, 1H), 2.40–2.24 (m, 1H); ^{13}C NMR (75 MHz, $CDCl_3$) δ 192.0 (d, *J* = 14.7 Hz), 164.2, 145.5 (d, *J* = 1.4 Hz), 130.3 (d, *J* = 2.3 Hz), 124.6, 113.8, 112.4, 90.9 (d, *J* = 185.6 Hz), 55.5, 30.1 (d, *J* = 19.1 Hz), 27.2 (d, *J* = 11.5 Hz); ^{19}F NMR (376 MHz, $CDCl_3$) δ −190.4; HRMS (ESI) m/z 217.0646 $(M+Na)^+$, calc. for $C_{11}H_{11}O_2FNa^+$ 217.0641.

The ee was determined by HPLC analysis: CHIRALPAK ID (4.6 mm i.d. x 250 mm); Hexane/2-propanol = 90/10; flow rate 1.0 mL/min; 25 °C; 254 nm; retention time: 29.0 min (minor) and 30.4 min (major).

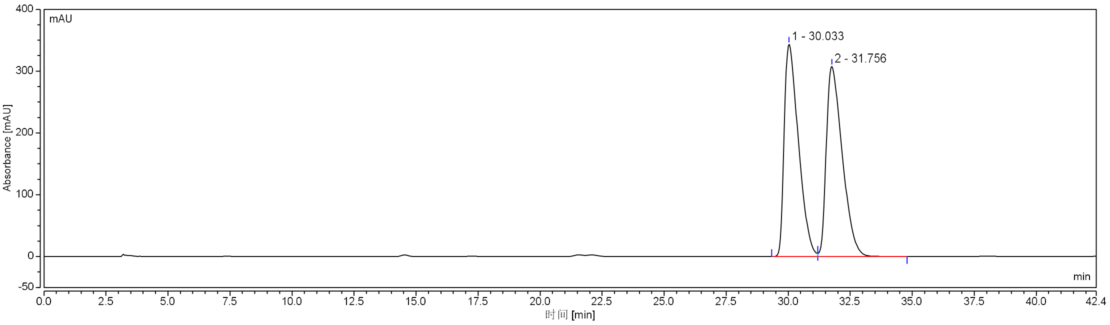

Entry	Retention Time	Area	Height	%Area
1	30.033	228.3652	343.65	49.83
2	31.756	229.8979	307.77	50.17

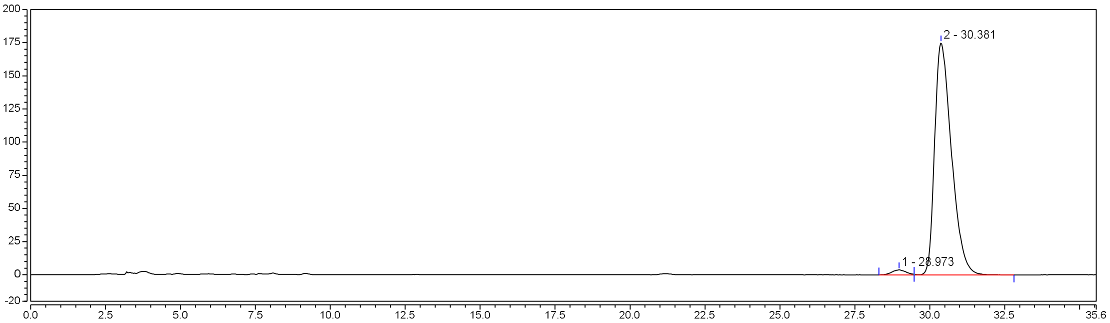

Entry	Retention Time	Area	Height	%Area
1	28.973	2.0324	3.70	1.45
2	30.381	114.0388	174.82	98.55

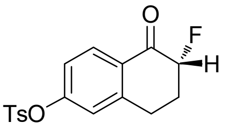

2j: yellow solid; Mp 87.2−88.7 °C; 26.1mg, 78% yield; 95% ee; $[\alpha]_D^{22}$ +29.3 (*c* 1.0, $CHCl_3$); ^{1}H NMR (300 MHz, $CDCl_3$) δ 7.94 (d, *J* = 8.6 Hz, 1H), 7.70 (d, *J* = 8.2 Hz, 2H), 7.33 (d, *J* = 8.0 Hz, 2H), 7.04 (s, 1H), 6.82 (d, *J* = 8.5 Hz, 1H), 5.11 (ddd, *J* = 47.7, 12.6, 5.1 Hz, 1H), 3.08 (dd, *J* = 8.7, 3.7 Hz, 2H), 2.68–2.49 (m, 1H), 2.45 (s, 3H), 2.40–2.23 (m, 1H); ^{13}C NMR (75 MHz, $CDCl_3$) δ 192.0 (d, *J* = 14.9 Hz), 153.4, 145.9, 145.1 (d, *J* = 1.4 Hz), 131.9, 129.9, 129.8, 129.8, 128.3, 122.3, 121.0, 90.7 (d, *J* = 187.3 Hz), 29.7 (d, *J* = 19.3 Hz), 26.8 (d, *J* = 11.5 Hz), 21.7; ^{19}F NMR (376 MHz, $CDCl_3$) δ −190.8; HRMS (ESI) m/z 357.0578 $(M+Na)^+$, calc. for $C_{17}H_{15}O_4FNaS^+$ 357.0573.

The ee was determined by HPLC analysis: CHIRALPAK IE (4.6 mm i.d. x 250 mm); Hexane/2-propanol = 80/20; flow rate 1.0 mL/min; 25 °C; 254 nm; retention time: 46.4 min (minor) and 43.3 min (major).

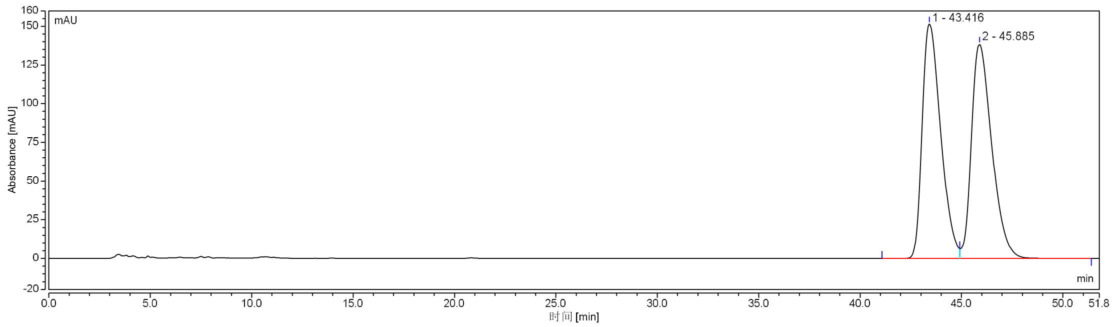

Entry	Retention Time	Area	Height	%Area
1	43.416	160.3057	151.45	49.95
2	45.885	160.6545	138.34	50.05

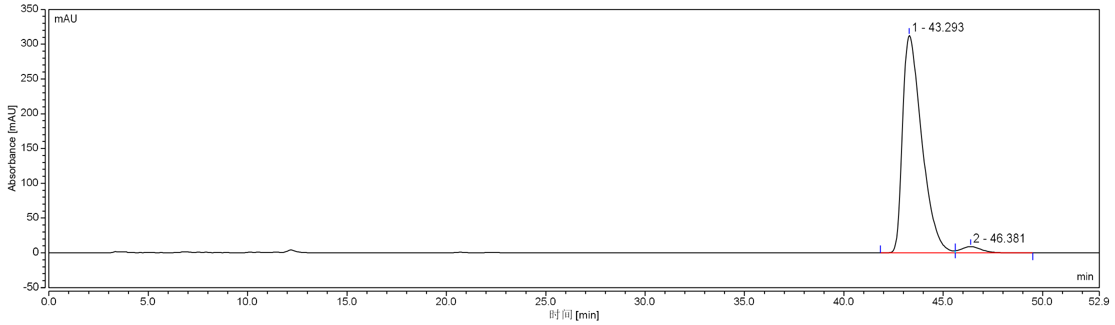

Entry	Retention Time	Area	Height	%Area
1	43.293	350.0930	312.40	97.34
2	46.381	10.3014	8.95	2.66

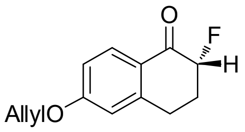

2k: white solid; Mp 100.9−102.2 °C; 15.2 mg, 69% yield; 94% ee; $[\alpha]_D^{22}$ +70.6 (*c* 1.0, $CHCl_3$); ^{1}H NMR (300 MHz, $CDCl_3$) δ 8.03 (d, *J* = 8.8 Hz, 1H), 6.87 (dd, *J* = 8.7, 2.1 Hz, 1H), 6.71 (s, 1H), 6.03 (ddd, *J* = 22.4, 10.5, 5.2 Hz, 1H), 5.37 (ddd, *J* = 13.9, 11.5, 1.1 Hz, 2H), 5.09 (ddd, *J* = 47.9, 12.4, 5.1 Hz, 1H), 4.60 (d, *J* = 5.2 Hz, 2H), 3.07 (dd, *J* = 8.8, 3.7 Hz, 2H), 2.54 (qd, *J* = 9.2, 4.2 Hz, 1H), 2.40–2.24 (m, 1H); ^{13}C NMR (75 MHz, $CDCl_3$) δ 191.9 (d, *J* = 14.6 Hz), 163.2, 145.5 (d, *J* = 1.3 Hz), 132.2, 130.3 (d, *J* = 2.3 Hz), 124.7, 118.3, 114.3, 113.3, 90.9 (d, *J* = 185.6 Hz), 68.9, 30.1 (d, *J* = 19.2 Hz), 27.2 (d, *J* = 11.5 Hz); ^{19}F NMR (376 MHz, $CDCl_3$) δ −190.4; HRMS (ESI) m/z 221.0979 $(M+H)^+$, calc. for $C_{13}H_{14}O_2F^+$ 221.0978.

The ee was determined by HPLC analysis: CHIRALPAK IE (4.6 mm i.d. x 250 mm); Hexane/2-propanol = 80/20; flow rate 1.0 mL/min; 25 °C; 254 nm; retention time: 19.9 min (minor) and 18.8 min (major).

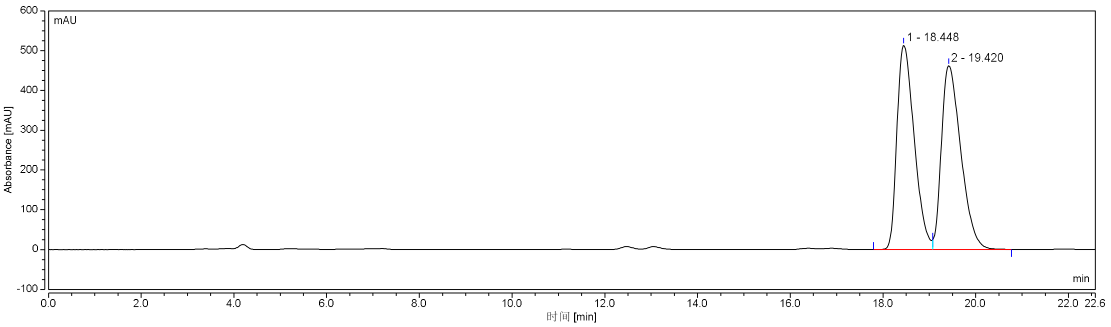

Entry	Retention Time	Area	Height	%Area
1	18.448	221.1624	513.09	49.73
2	19.420	223.5872	461.37	50.27

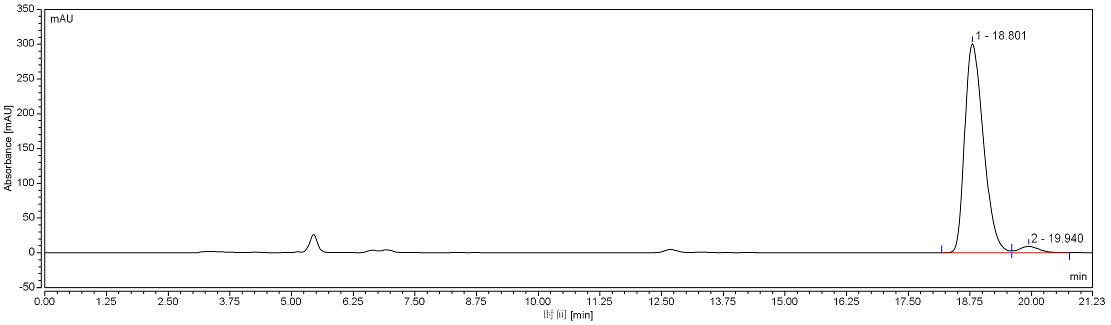

Entry	Retention Time	Area	Height	%Area
1	18.801	131.9842	300.36	96.96
2	19.940	4.1422	8.98	3.04

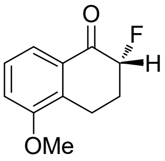

2l: white solid; Mp 122.4–123.8 °C; 18.4 mg, 95% yield; >99% ee; $[\alpha]_D^{22}$ +26.9 (*c* 1.0, $CHCl_3$); ^{1}H NMR (300 MHz, $CDCl_3$) δ 7.50 (d, *J* = 2.3 Hz, 1H), 7.17 (d, *J* = 8.5 Hz, 1H), 7.09 (dd, *J* = 8.5, 2.5 Hz, 1H), 5.12 (ddd, *J* = 47.9, 12.7, 5.1 Hz, 1H), 3.83 (s, 3H), 3.05 (dd, *J* = 8.9, 3.6 Hz, 2H), 2.61–2.49 (m, 1H), 2.40–2.24 (m, 1H); ^{13}C NMR (75 MHz, $CDCl_3$) δ 193.3 (d, *J* = 14.6 Hz), 158.6, 135.6 (d, *J* = 1.3 Hz), 132.0 (d, *J* = 0.7 Hz), 129.9, 122.7, 109.3 (d, *J* = 2.3 Hz), 91.3 (d, *J* = 186.6 Hz), 55.5, 30.3 (d, *J* =18.8 Hz), 26.2 (d, *J* = 11.6 Hz); ^{19}F NMR (376 MHz, $CDCl_3$) δ −190.4; HRMS (ESI) m/z 217.0647 $(M+Na)^+$, calc. for $C_{11}H_{11}O_2FNa^+$ 217.0641.

The ee was determined by HPLC analysis: CHIRALPAK IC (4.6 mm i.d. x 250 mm); Hexane/2-propanol = 80/20; flow rate 1.0 mL/min; 25 °C; 254 nm; retention time: 23.1 min (minor) and 20.5 min (major).

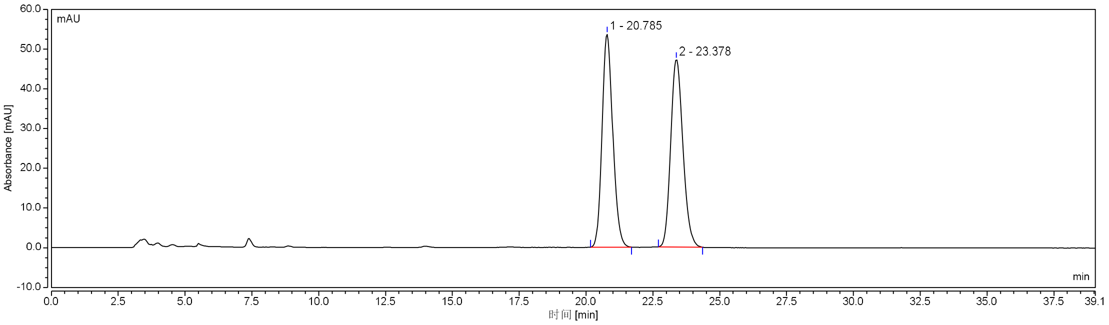

Entry	Retention Time	Area	Height	%Area
1	20.668	22.3796	47.30	50.21
2	23.215	22.1894	41.68	49.79

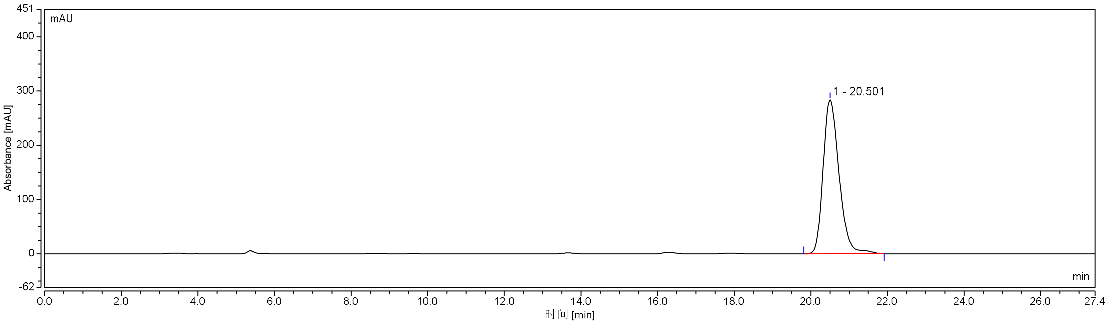

Entry	Retention Time	Area	Height	%Area
1	20.501	137.3159	283.76	100.00
2	23.1	0	0	0

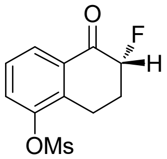

2m: white solid; Mp 104.3−105.9 °C; 17.6 mg, 68% yield; 94% ee; $[\alpha]_D^{22}$ +64.0 (*c* 1.0, $CHCl_3$); ^1H NMR (300 MHz, $CDCl_3$) δ 8.06 (d, *J* = 7.8 Hz, 1H), 7.54 (d, *J* = 7.9 Hz, 1H), 7.43 (t, *J* = 7.9 Hz, 1H), 5.18 (ddd, J = 47.9, 12.8, 5.1 Hz, 1H), 3.40–3.32 (m, 1H), 3.12–3.01 (m, 1H), 2.66–2.57 (m, 1H), 2.45–2.22 (m, 1H); ^{13}C NMR (75 MHz, $CDCl_3$) δ 192.1 (d, *J* = 14.9 Hz), 146.4, 136.5 (d, *J* = 1.4 Hz), 133.4 (d, *J* = 0.7 Hz), 128.2, 127.7, 126.8 (d, *J* = 2.1 Hz), 90.5 (d, *J* = 188.9 Hz), 38.5, 29.0 (d, *J* = 19.5 Hz), 21.7 (d, *J* = 11.7 Hz); ^{19}F NMR (376 MHz, $CDCl_3$) δ −191.1; HRMS (ESI) m/z 281.0259 $(M+Na)^+$, calc. for $C_{11}H_{11}O_4FNaS^+$ 281.0260.

The ee was determined by HPLC analysis: CHIRALPAK ID (4.6 mm i.d. x 250 mm); Hexane/2-propanol = 90/10; flow rate 2.0 mL/min; 25 °C; 254 nm; retention time: 35.3 min (minor) and 31.8 min (major).

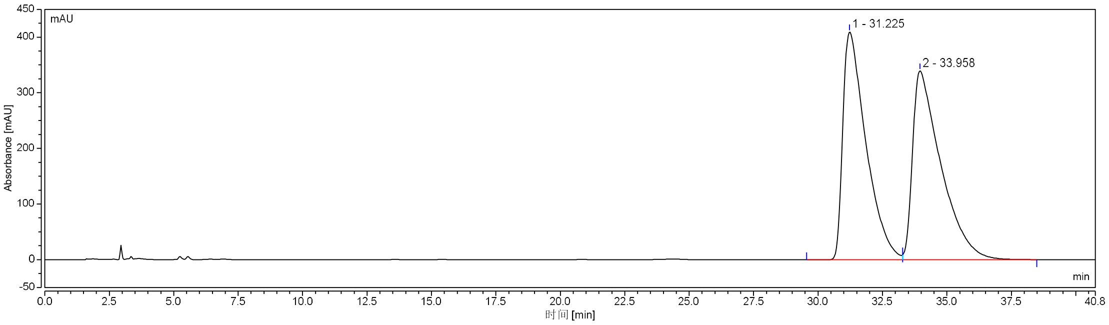

Entry	Retention Time	Area	Height	%Area
1	31.225	425.4863	409.32	49.70
2	33.958	430.6803	339.17	50.30

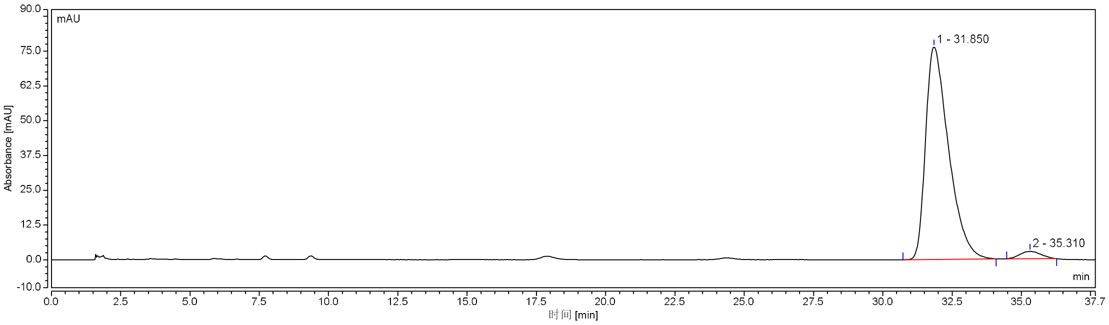

Entry	Retention Time	Area	Height	%Area
1	31.850	73.6254	76.38	96.92
2	35.310	2.3414	2.67	3.08

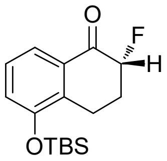

2n: yellow oil; 20.6 mg, 70% yield; 97% ee; $[\alpha]_D^{22}$ +50.5 (*c* 1.0, $CHCl_3$); ^1H NMR (300 MHz, $CDCl_3$) δ 7.99 (d, *J* = 8.6 Hz, 1H), 6.79 (dd, *J* = 8.6, 2.1 Hz, 1H), 6.66 (s, 1H), 5.10 (ddd, *J* = 47.9, 12.5, 5.1 Hz, 1H), 3.05 (dd, *J* = 8.8, 4.0 Hz, 2H), 2.54 (qd, *J* = 9.2, 4.3 Hz, 1H), 2.40–2.25 (m, 1H), 0.98 (s, 9H), 0.24 (s, 6H); ^{13}C NMR (75 MHz, $CDCl_3$) δ 192.2 (d, *J* = 15.8 Hz), 161.1, 145.4 (d, *J* = 1.3 Hz), 130.3 (d, *J* = 2.3 Hz), 125.1, 119.4, 119.0, 91.0 (d, *J* = 185.7 Hz), 30.1 (d, *J* = 19.1 Hz), 27.0 (d, *J* = 11.5 Hz), 25.5, 18.2, −4.3, −4.3; ^{19}F NMR (376 MHz, $CDCl_3$) δ −190.9; HRMS (ESI) m/z 295.1529 $(M+H)^+$, calc. for $C_{16}H_{24}O_2FNSi^+$ 295.1530.

The ee was determined by HPLC analysis: CHIRALCEL OD-H (4.6 mm i.d. x 250 mm); Hexane/2-propanol = 90/10; flow rate 1.0 mL/min; 25 ºC; 254 nm; retention time: 5.0 min (minor) and 6.2 min (major).

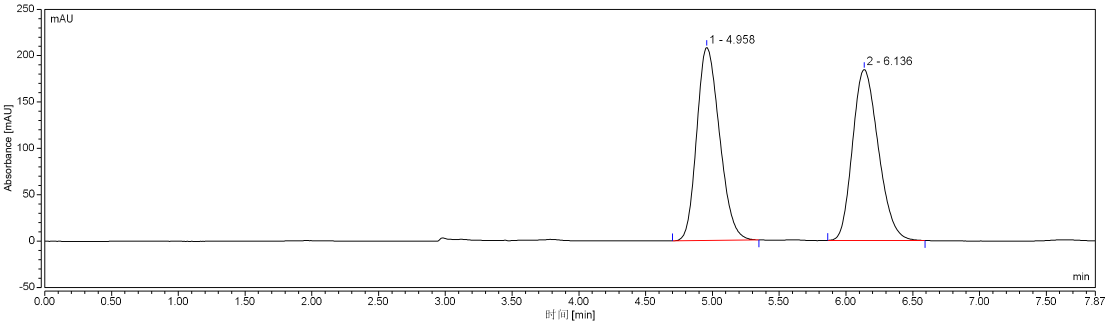

Entry	Retention Time	Area	Height	%Area
1	4.958	41.2355	208.11	49.99
2	6.136	41.2497	184.39	50.01

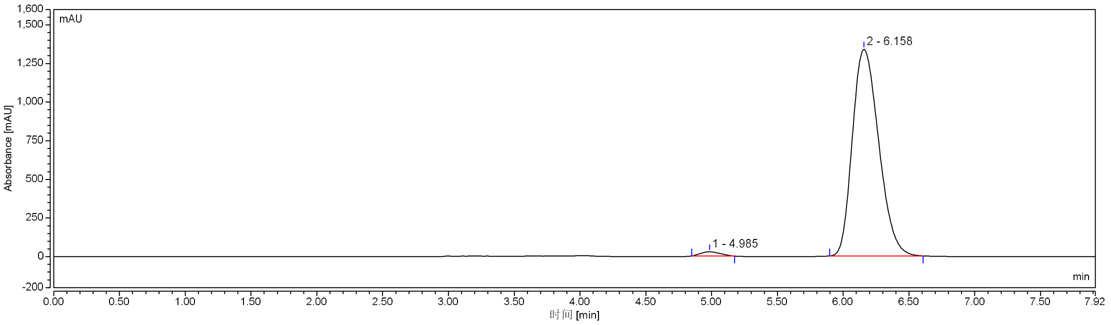

Entry	Retention Time	Area	Height	%Area
1	4.985	4.5785	26.63	1.45
2	6.158	310.1722	1336.86	98.55

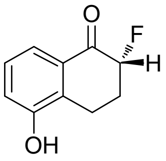

2o: white solid; Mp 174.9−196.4 ºC; 20.6 mg, 72% yield; 92% ee; $[\alpha]^{22}_{D}$ +46.8 (*c* 1.0, $CHCl_3$); ^{1}H NMR (300 MHz, CD_3OD) δ 7.46 (d, *J* = 7.7 Hz, 1H), 7.19 (t, *J* = 7.9 Hz, 1H), 7.02 (d, *J* = 7.9 Hz, 1H), 5.24 (ddd, *J* = 48.1, 13.1, 5.2 Hz, 1H), 3.23 (ddd, *J* = 17.9, 8.1, 4.7 Hz, 1H), 2.96–2.73 (m, 1H), 2.55 (tt, *J* = 14.6, 5.0 Hz, 1H), 2.41–2.08 (m, 1H); ^{13}C NMR (75 MHz, CD_3OD) δ 196.3 (d, *J* = 14.2 Hz), 156.2, 133.3, 131.9 (d, *J* = 1.5 Hz), 128.4, 120.7, 118.8 (d, *J* = 2.2 Hz), 92.3 (d, *J* = 185.2 Hz), 30.4 (d, *J* = 18.7 Hz), 21.8 (d, *J* = 12.0 Hz); ^{19}F NMR (376 MHz, CD_3OD) δ −192.6; HRMS (ESI) m/z 181.0671 $(M+H)^+$, calc. for $C_{10}H_{10}O_2F^+$ 181.0665.

The ee was determined by HPLC analysis: CHIRALPAK AS-H (4.6 mm i.d. x 250 mm); Hexane/2-propanol = 80/20; flow rate 1.0 mL/min; 25 ºC; 254 nm; retention time: 9.9 min (minor) and 13.8 min (major).

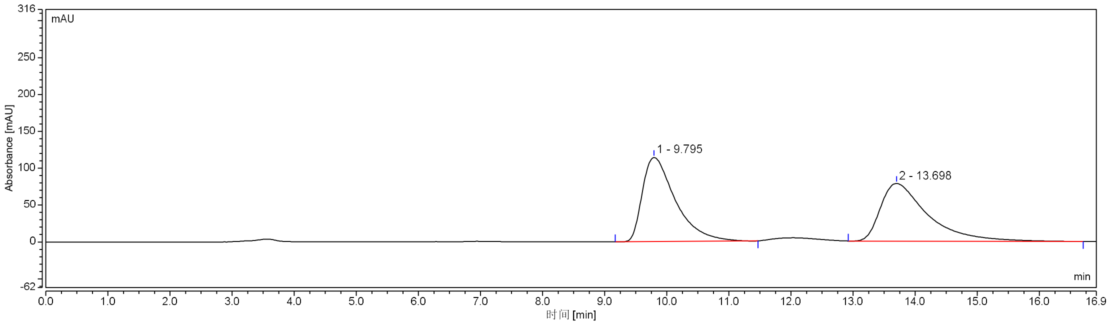

Entry	Retention Time	Area	Height	%Area
1	9.795	70.2708	113.95	50.47
2	13.698	68.9706	78.14	49.53

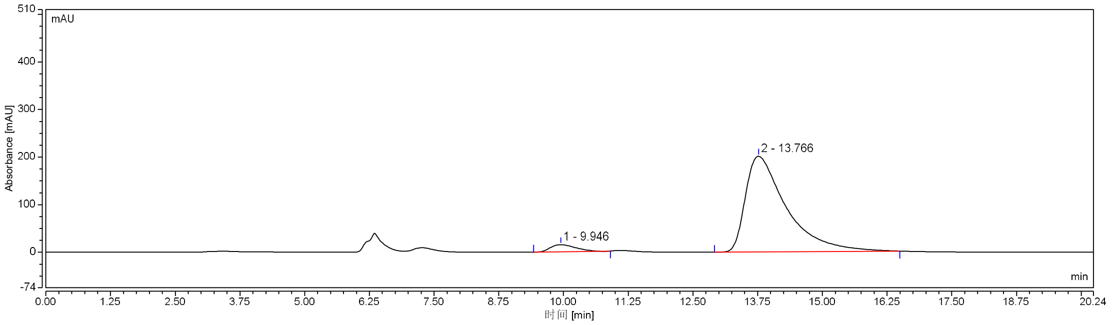

Entry	Retention Time	Area	Height	%Area
1	9.946	8.2645	14.92	4.18
2	13.766	184.7350	201.24	95.82

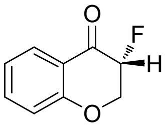

2p: white solid; Mp 71.8–73.2 °C; 14.8 mg, 89% yield; 93% ee; $[\alpha]_D^{22}$ +166.8 (*c* 1.0, $CHCl_3$); ^{1}H NMR (300 MHz, $CDCl_3$) δ 7.92 (d, *J* = 7.9 Hz, 1H), 7.53 (dd, *J* = 11.3, 4.2 Hz, 1H), 7.09 (t, *J* = 7.5 Hz, 1H), 7.01 (d, *J* = 8.4 Hz, 1H), 5.17 (ddd, *J* = 47.0, 9.2, 4.8 Hz, 1H), 4.69–4.49 (m, 2H); ^{13}C NMR (75 MHz, $CDCl_3$) δ 187.1 (d, *J* = 15.7 Hz), 161.2, 136.8, 127.6 (d, *J* = 1.3 Hz), 122.3, 119.4, 117.9, 85.5 (d, *J* = 187.0 Hz), 68.6 (d, *J* = 25.8 Hz); ^{19}F NMR (376 MHz, $CDCl_3$) δ −204.0; HRMS (ESI) m/z 189.0330 $(M+Na)^+$, calc. for $C_9H_7O_2FNa^+$ 189.0328.

The ee was determined by HPLC analysis: CHIRALPAK IC (4.6 mm i.d. x 250 mm); Hexane/2-propanol = 80/20; flow rate 1.0 mL/min; 25 °C; 254 nm; retention time: 8.6 min (minor) and 10.4 min (major).

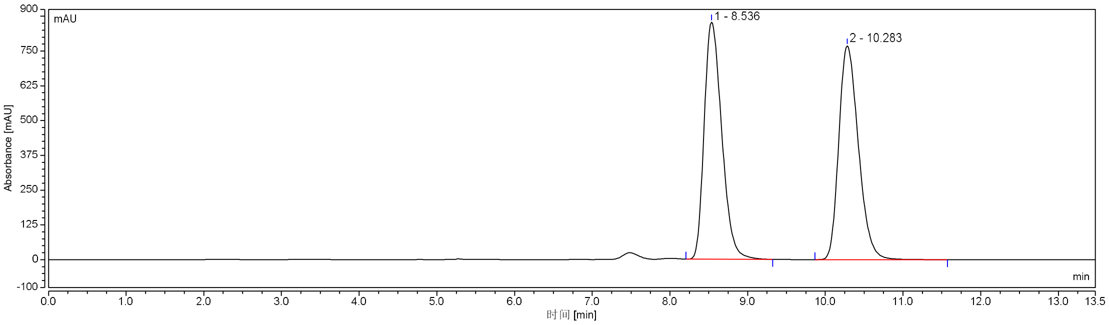

Entry	Retention Time	Area	Height	%Area
1	8.536	222.9058	852.26	49.86
2	10.283	224.1198	768.28	50.14

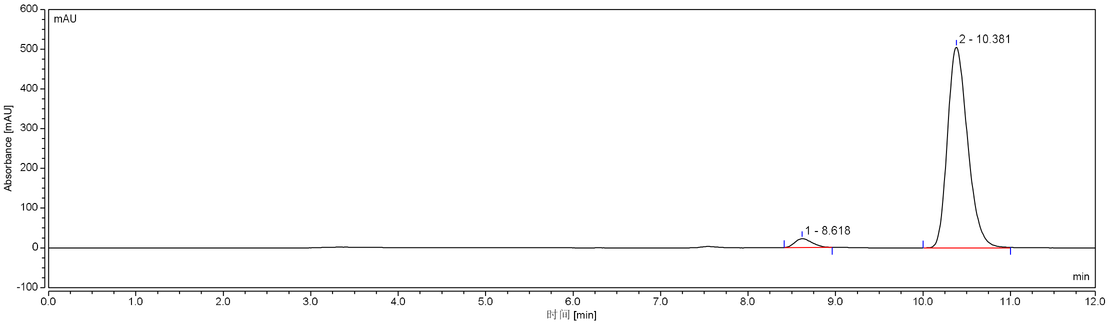

Entry	Retention Time	Area	Height	%Area
1	8.618	5.0461	22.03	3.60
2	10.381	135.1093	505.15	96.40

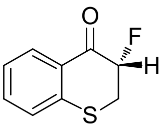

2q: white solid; Mp 87.3−88.9 ºC; 16.4 mg, 90% yield; 93% ee; $[\alpha]_D^{22}$ +4.5 (*c* 1.0, $CHCl_3$); ^1H NMR (300 MHz, $CDCl_3$) δ 8.11 (*J* = 8.0 Hz, 1H), 7.46–7.41 (m, 1H), 7.28 (s, 1H), 7.26–7.21 (m, 1H), 5.43 (ddd, J = 47.5, 13.4, 4.9 Hz, 1H), 3.61 (td, *J* = 13.0, 2.9 Hz, 1H), 3.33–3.24 (m, 1H); ^{13}C NMR (75 MHz, $CDCl_3$) δ 189.8 (d, *J* = 15.4 Hz), 140.4, 134.0, 130.2, 129.9 (d, *J* = 2.1 Hz), 127.1, 125.5, 89.3 (d, *J* = 195.0 Hz), 30.8 (d, *J* = 23.3 Hz); ^{19}F NMR (376 MHz, $CDCl_3$) δ −184.0; HRMS (ESI) m/z 205.0099 $(M+Na)^+$, calc. for $C_9H_7OFSNa^+$ 205.0099.

The ee was determined by HPLC analysis: CHIRALPAK IC (4.6 mm i.d. x 250 mm); Hexane/2-propanol = 80/20; flow rate 1.0 mL/min; 25 ºC; 254 nm; retention time: 14.4 min (minor) and 12.9 min (major).

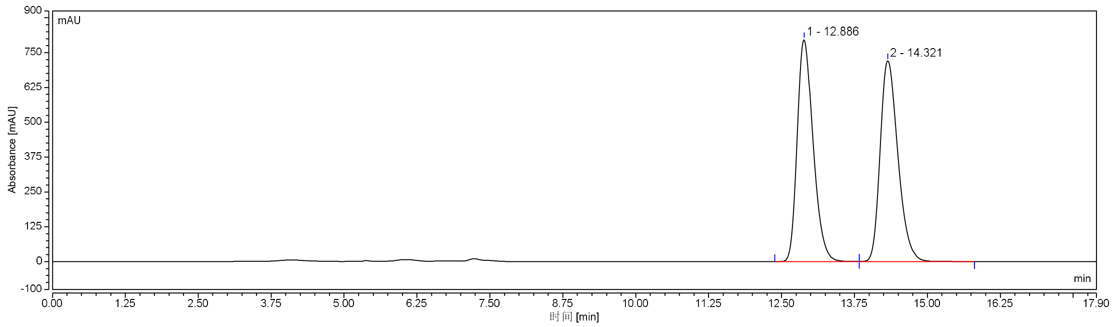

Entry	Retention Time	Area	Height	%Area
1	12.886	252.6142	796.43	49.94
2	14.321	253.1922	720.53	50.06

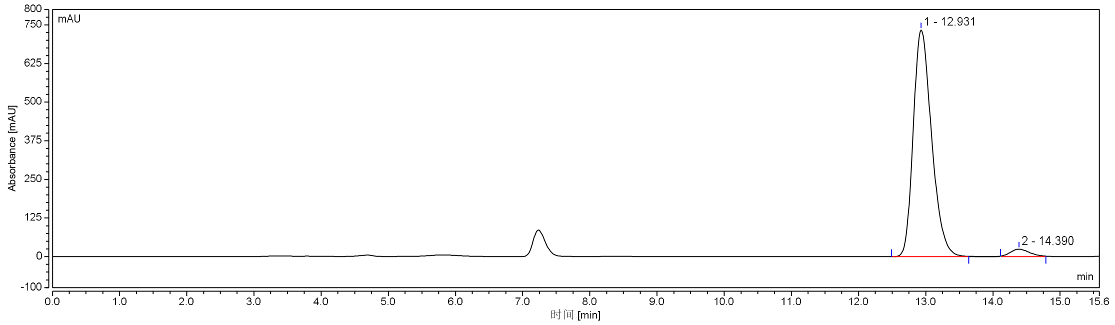

Entry	Retention Time	Area	Height	%Area
1	12.931	232.5183	733.59	96.61
2	14.390	8.1545	24.30	3.39

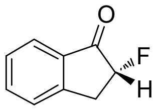

2r: white solid; Mp 54.6–56.0 °C; 11.3 mg, 75% yield; 91% ee; $[\alpha]_D^{22}$ +7.0 (*c* 1.0, $CHCl_3$); ^{1}H NMR (300 MHz, $CDCl_3$) δ 7.78 (d, *J* = 7.7 Hz, 1H), 7.67 (t, *J* = 7.5 Hz, 1H), 7.44 (dd, *J* = 14.1, 7.3 Hz, 2H), 5.27 (ddd, *J* = 51.0, 7.8, 4.4 Hz, 1H), 3.68–3.57 (m, 1H), 3.39–3.15 (m, 1H); ^{13}C NMR (75 MHz, $CDCl_3$) δ 200.0 (d, *J* = 14.7 Hz), 149.6 (d, *J* = 5.7 Hz), 133.8 (d, *J* = 1.1 Hz), 133.8, 128.4, 126.8 (d, *J* = 1.6 Hz), 124.7 (d, *J* = 1.3 Hz), 90.5 (d, *J* = 189.2 Hz), 33.4 (d, *J* = 21.3 Hz); ^{19}F NMR (376 MHz, $CDCl_3$) δ −194.0; HRMS (ESI) m/z 173.0376 $(M+Na)^+$, calc. for $C_9H_7OFNa^+$ 173.0379.

The ee was determined by HPLC analysis: CHIRALPAK ID (4.6 mm i.d. x 250 mm); Hexane/2-propanol = 90/10; flow rate 1.0 mL/min; 25 °C; 254 nm; retention time: 10.3 min (minor) and 11.3 min (major).

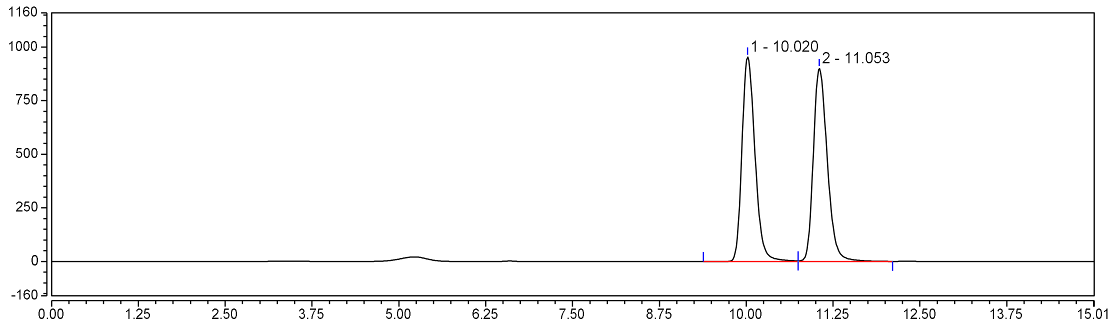

Entry	Retention Time	Area	Height	%Area
1	10.020	208.8269	953.60	49.71
2	11.053	211.2550	900.59	50.29

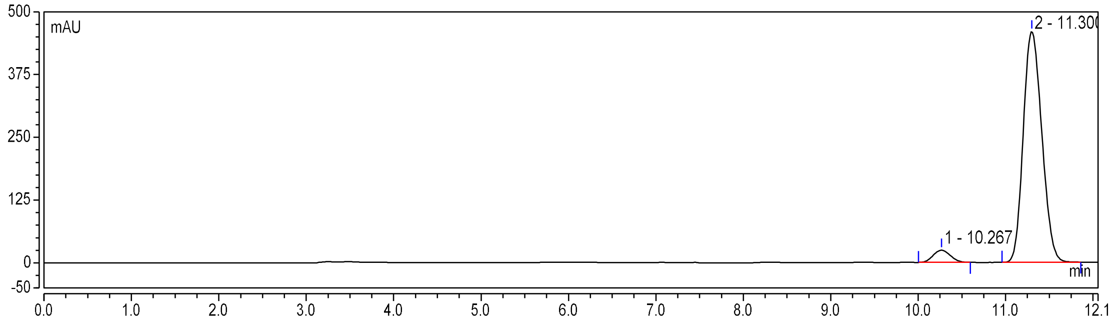

Entry	Retention Time	Area	Height	%Area
1	10.267	5.5552	24.36	4.68
2	11.300	113.1396	459.35	95.32

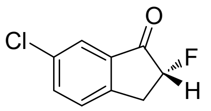

2s: white solid; Mp 89.1–91.3 °C; 13.5 mg, 73% yield; 87% ee; $[\alpha]_D^{22}$ −15.6 (*c* 1.0, $CHCl_3$); ^1H NMR (300 MHz, $CDCl_3$) δ 7.72 (d, *J* = 8.2 Hz, 1H), 7.46–7.39 (m, 2H), 5.26 (ddd, *J* = 50.9, 7.7, 4.3 Hz, 1H), 3.66–3.55 (m, 1H), 3.28–3.13 (m, 1H); ^{13}C NMR (75 MHz, $CDCl_3$) δ 198.3 (d, *J* = 14.9 Hz), 151.1 (d, *J* = 5.5 Hz), 142.9, 132.3 (d, *J* = 1.2 Hz), 129.3, 127.0 (d, *J* = 1.6 Hz), 125.9 (d, *J* = 1.3 Hz), 90.1 (d, *J* = 189.7 Hz), 33.2 (d, *J* = 22.0 Hz); ^{19}F NMR (376 MHz, $CDCl_3$) δ −193.3; HRMS (ESI) m/z 206.9991 $(M+Na)^+$, calc. for $C_9H_6OFNaCl^+$ 206.9989.

The ee was determined by HPLC analysis: CHIRALPAK ID (4.6 mm i.d. x 250 mm); Hexane/2-propanol = 95/5; flow rate 1.0 mL/min; 25 °C; 254 nm; retention time: 15.8 min (minor) and 17.5 min (major).

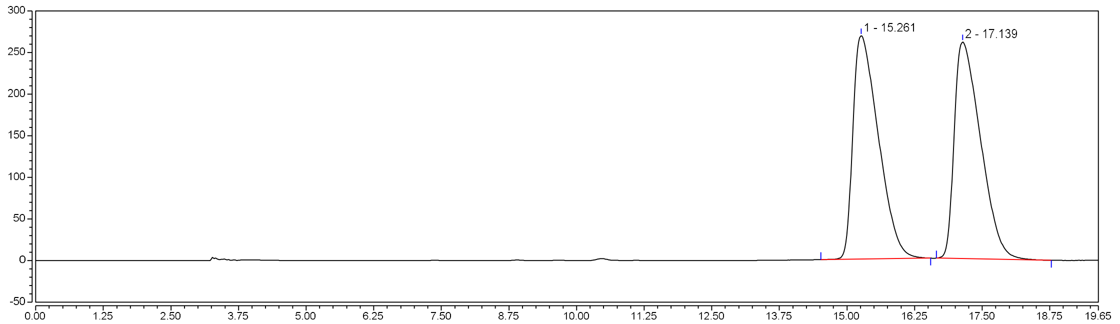

Entry	Retention Time	Area	Height	%Area
1	15.261	146.4709	268.32	49.95
2	17.139	146.7617	260.18	50.05

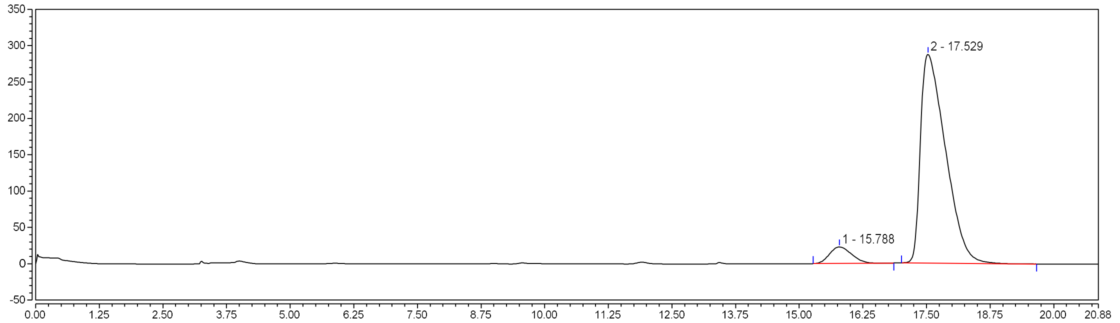

Entry	Retention Time	Area	Height	%Area
1	15.788	11.2989	22.61	6.48
2	17.529	163.0464	286.77	93.52

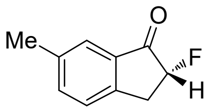

2t: white solid; Mp 67.9–69.4 °C; 13.6 mg, 83% yield; 91% ee; $[\alpha]_D^{22}$ −14.7 (*c* 1.0, $CHCl_3$); ^1H NMR (300 MHz, $CDCl_3$) δ 7.58 (s, 1H), 7.48 (d, *J* = 7.9 Hz, 1H), 7.34 (d, *J* = 7.8 Hz, 1H), 5.25 (ddd, *J* = 51.0, 7.7, 4.3 Hz, 1H), 3.62–3.52 (m, 1H), 3.23–3.09 (m, 1H), 2.40 (s, 3H); ^{13}C NMR (75 MHz, $CDCl_3$) δ 200.0 (d, *J* = 14.8 Hz), 147.0 (d, *J* = 5.8 Hz), 138.5, 137.6, 134.0 (d, *J* = 1.3 Hz), 126.4 (d, *J* = 1.6 Hz), 124.6 (d, *J* = 1.2 Hz), 90.8 (d, *J* = 189.1 Hz), 33.1 (d, *J* = 21.3 Hz), 21.1; ^{19}F NMR (376 MHz, $CDCl_3$) δ −193.6; HRMS (ESI) m/z 187.0536 $(M+Na)^+$, calc. for $C_{10}H_9OFNa^+$ 187.0535.

The ee was determined by HPLC analysis: CHIRALPAK IC (4.6 mm i.d. x 250 mm); Hexane/2-propanol = 70/30; flow rate 1.0 mL/min; 25 °C; 254 nm; retention time: 11.8 min (minor) and 16.0 min (major).

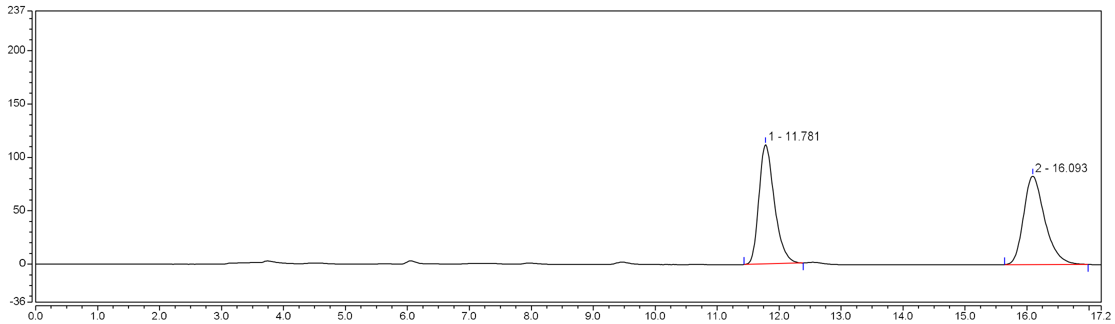

Entry	Retention Time	Area	Height	%Area
1	11.781	32.3695	111.76	49.60
2	16.093	32.8877	82.83	50.40

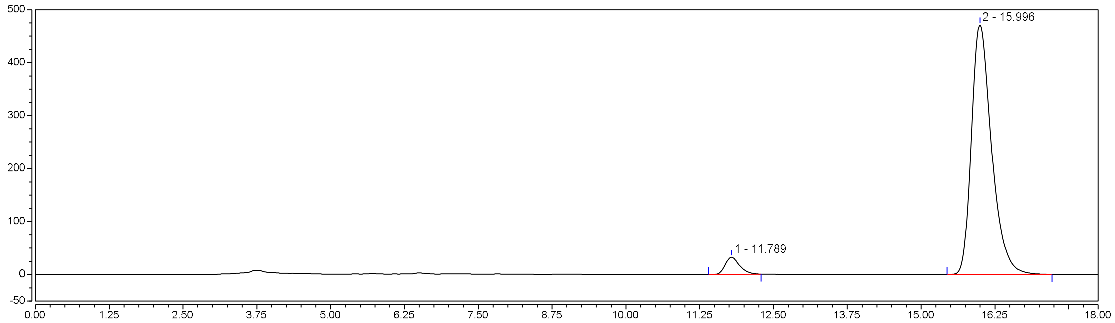

Entry	Retention Time	Area	Height	%Area
1	11.789	9.1073	32.51	4.65
2	15.996	186.7234	470.98	95.35

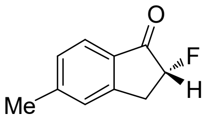

2u: white solid; Mp 81.1−82.5 ºC; 13.6 mg, 83% yield; 92% ee; $[\alpha]_D^{22}$ −8.7 (*c* 1.0, $CHCl_3$); ^1H NMR (300 MHz, $CDCl_3$) δ 7.69 (d, *J* = 7.8 Hz, 1H), 7.25 (t, *J* = 5.5 Hz, 2H), 5.25 (ddd, *J* = 51.1, 7.7, 4.3 Hz, 1H), 3.63–3.52 (m, 1H), 3.26–3.10 (m, 1H), 2.46 (s, 3H); ^{13}C NMR (75 MHz, $CDCl_3$) δ 199.4 (d, *J* = 14.8 Hz), 150.1 (d, *J* = 5.6 Hz), 147.9, 131.6 (d, *J* = 1.4 Hz), 129.7, 127.1 (d, *J* = 1.5 Hz), 124.6 (d, *J* = 1.4 Hz), 90.6 (d, *J* = 188.8 Hz), 33.3 (d, *J* = 21.4 Hz), 22.3; ^{19}F NMR (376 MHz, $CDCl_3$) δ −193.4; HRMS (ESI) m/z 187.0540 $(M+Na)^+$, calc. for $C_{10}H_9OFNa^+$ 187.0535.

The ee was determined by HPLC analysis: CHIRALPAK ID (4.6 mm i.d. x 250 mm); Hexane/2-propanol = 90/10; flow rate 1.0 mL/min; 25 ºC; 254 nm; retention time: 14.3 min (minor) and 16.9 min (major).

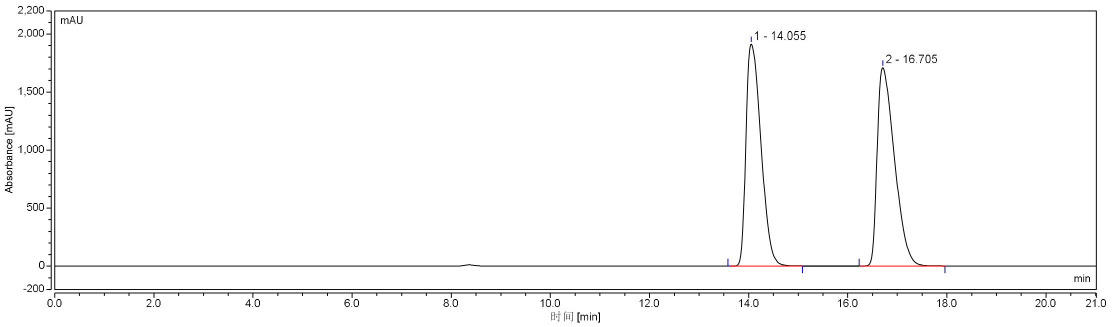

Entry	Retention Time	Area	Height	%Area
1	14.055	670.0501	1914.53	49.21
2	16.705	691.5219	1711.24	50.79

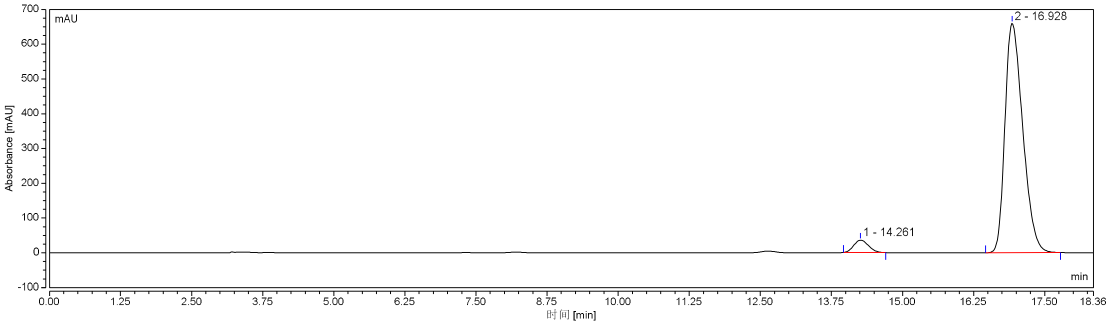

Entry	Retention Time	Area	Height	%Area
1	14.261	10.6185	35.28	4.25
2	16.928	239.3537	660.20	95.75

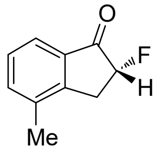

2v: white solid; Mp 64.8−66.3 °C; 14.8 mg, 90% yield; 91% ee; $[\alpha]_D^{22}$ −9.0 (*c* 1.0, $CHCl_3$); ^{1}H NMR (300 MHz, $CDCl_3$) δ 7.62 (d, *J* = 7.6 Hz, 1H), 7.47 (d, *J* = 7.4 Hz, 1H), 7.34 (t, *J* = 7.5 Hz, 1H), 5.26 (ddd, *J* = 50.9, 7.7, 4.1 Hz, 1H), 3.59–3.49 (m, 1H), 3.14–2.99 (m, 1H), 2.35 (s, 3H); ^{13}C NMR (75 MHz, $CDCl_3$) δ 200.3 (d, *J* = 14.6 Hz), 148.7 (d, *J* = 5.1 Hz), 136.8, 136.1 (d, *J* = 1.4 Hz), 133.7 (d, *J* = 1.2 Hz), 128.5, 122.0 (d, *J* = 1.2 Hz), 90.5 (d, *J* = 188.7Hz), 32.1 (d, *J* = 21.3 Hz), 17.7; ^{19}F NMR (376 MHz, $CDCl_3$) δ −193.9; HRMS (ESI) m/z 187.0537 (M+Na)$^+$, calc. for $C_{10}H_9OFNa^+$ 187.0535.

The ee was determined by HPLC analysis: CHIRALPAK ID (4.6 mm i.d. x 250 mm); Hexane/2-propanol = 90/10; flow rate 1.0 mL/min; 25 °C; 254 nm; retention time: 12.0 min (minor) and 13.2 min (major).

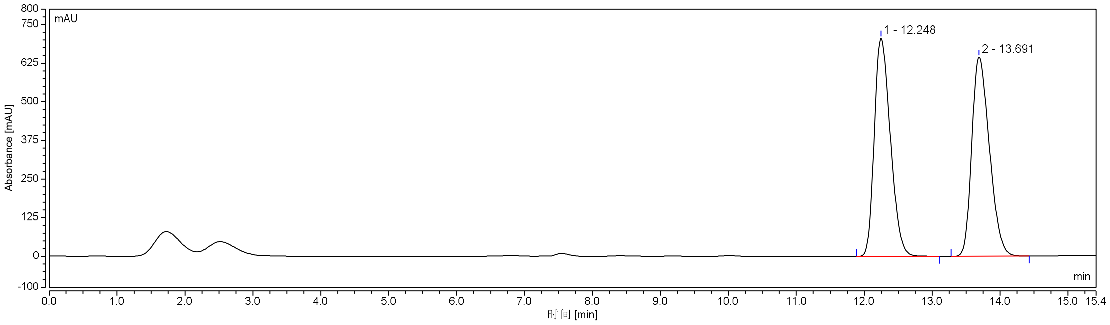

Entry	Retention Time	Area	Height	%Area
1	12.248	194.1255	706.12	50.00
2	13.691	194.1314	644.35	50.00

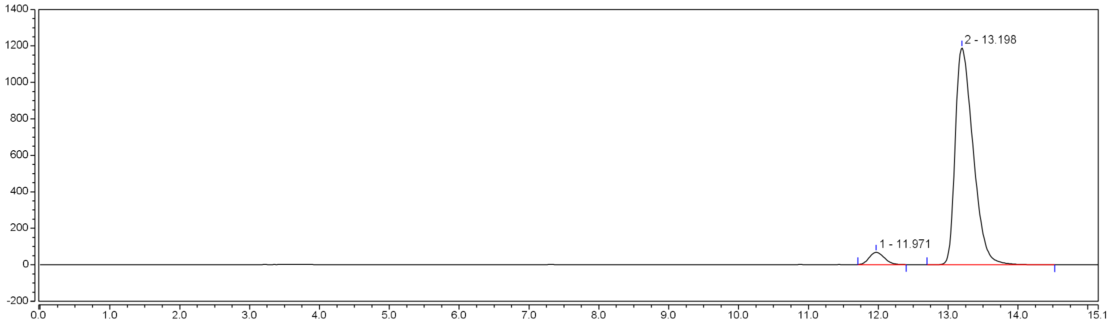

Entry	Retention Time	Area	Height	%Area
1	11.971	16.8186	67.60	4.56
2	13.198	352.1670	1188.46	95.44

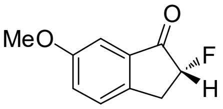

2w: white solid; Mp 67.8−68.8 °C; 14.9 mg, 83% yield; 84% ee; $[\alpha]_D^{22}$ −18.2 (*c* 1.0, $CHCl_3$); ^1H NMR (300 MHz, $CDCl_3$) δ 7.42 (d, *J* = 8.4 Hz, 1H), 7.34–7.30 (m, 1H), 7.26 (d, *J* = 2.2 Hz, 1H), 5.33 (ddd, *J* = 51.0, 7.6, 4.2 Hz, 1H), 3.90 (s, 3H), 3.67–3.57 (m, 1H), 3.32–3.12 (m, 1H); ^{13}C NMR (75 MHz, $CDCl_3$) δ 199.9 (d, *J* = 15.1 Hz), 159.9, 142.4 (d, *J* = 5.9 Hz), 134.9 (d, *J* = 1.1 Hz), 127.5 (d, *J* = 1.6 Hz), 125.7, 105.7 (d, *J* = 1.1 Hz), 91.0 (d, *J* = 189.3 Hz), 55.6, 32.7 (d, *J* = 21.2 Hz); ^{19}F NMR (376 MHz, $CDCl_3$) δ −193.2; HRMS (ESI) m/z 203.0492 $(M+Na)^+$, calc. for $C_{10}H_9O_2FNa^+$ 203.0484.

The ee was determined by HPLC analysis: CHIRALPAK ID (4.6 mm i.d. x 250 mm); Hexane/2-propanol = 90/10; flow rate 1.0 mL/min; 25 °C; 254 nm; retention time: 13.1 min (minor) and 14.3 min (major).

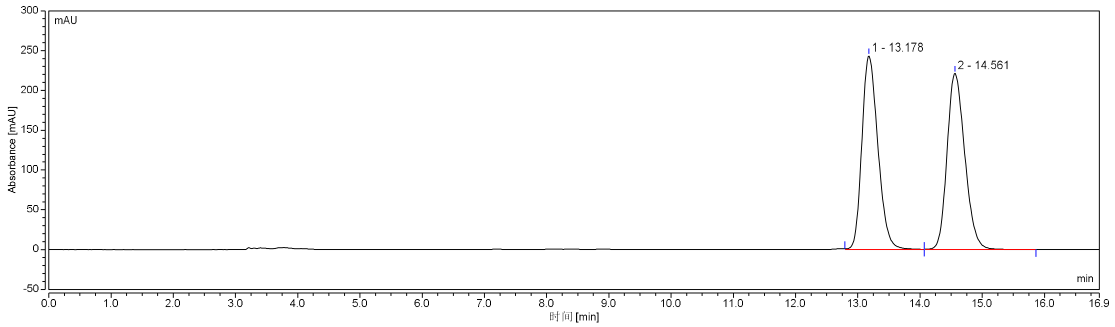

Entry	Retention Time	Area	Height	%Area
1	13.178	72.7721	243.42	49.96
2	14.561	72.8893	221.28	50.04

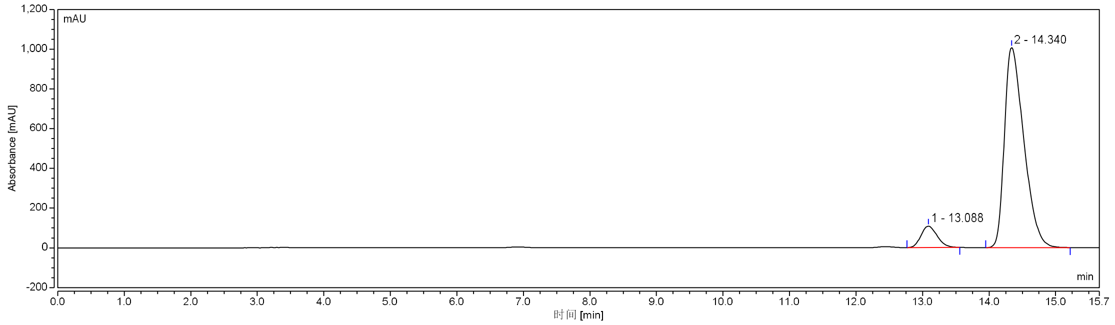

Entry	Retention Time	Area	Height	%Area
1	13.088	30.2802	107.65	8.16
2	14.340	340.8339	1006.39	91.84

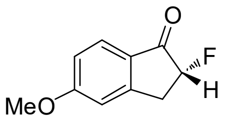

2x: white solid; Mp 122.9−124.1 ºC; 9.9 mg, 55% yield; 91% ee; $[\alpha]_D^{22}$ +11.2 (*c* 1.0, $CHCl_3$); ^{1}H NMR (300 MHz, $CDCl_3$) δ 7.73 (d, *J* = 8.6 Hz, 1H), 6.87 (s, 1H), 5.22 (ddd, *J* = 51.3, 7.6, 4.1 Hz, 1H), 3.90 (s, 3H), 3.68–3.43 (m, 1H), 3.15 (ddd, *J* = 25.3, 14.6, 6.0 Hz, 1H); ^{13}C NMR (75 MHz, $CDCl_3$) δ 197.8 (d, *J* = 15.0 Hz), 166.5, 152.8 (d, *J* = 5.6 Hz), 127.1, 126.6 (d, *J* = 1.2 Hz), 116.4, 109.9 (d, *J* = 1.6 Hz), 90.5 (d, *J* = 187.9 Hz), 55.8, 33.6 (d, *J* = 21.7 Hz); ^{19}F NMR (376 MHz, $CDCl_3$) δ −192.2; HRMS (ESI) m/z 203.0487 $(M+Na)^+$, calc. for $C_{10}H_9O_2FNa^+$ 203.0484.

The ee was determined by HPLC analysis: CHIRALPAK ID (4.6 mm i.d. x 250 mm); Hexane/2-propanol = 90/10; flow rate 1.0 mL/min; 25 ºC; 254 nm; retention time: 24.6 min (minor) and 28.3 min (major).

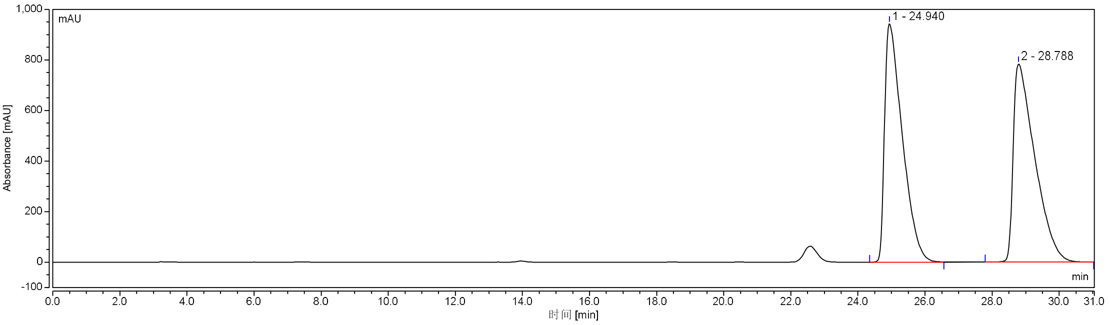

Entry	Retention Time	Area	Height	%Area
1	24.940	577.0831	943.36	49.72
2	28.788	583.5189	783.87	50.28

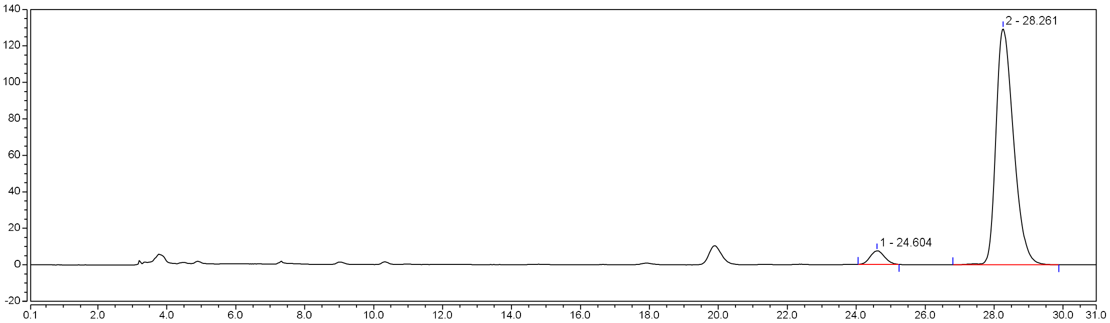

Entry	Retention Time	Area	Height	%Area
1	24.604	3.5844	7.47	4.47
2	28.261	76.5542	129.42	95.53

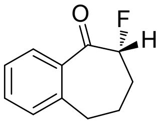

2y: yellow oil;15.2 mg, 85% yield; 93% ee; $[\alpha]_D^{22}$ +95.9 (*c* 1.0, $CHCl_3$); ^1H NMR (300 MHz, $CDCl_3$) δ 7.76 (d, *J* = 6.0 Hz, 1H), 7.43 (t, *J* = 15.0, 9.0, 6.0 Hz, 1H), 7.32 (t, *J* = 15.0, 9.0, 6.0 Hz, 1H), 7.22 (d, *J* = 9.0 Hz, 1H), 5.36–5.15 (m, 1H), 3.08–2.90 (m, 2H), 2.40–2.27 (m, 1H), 2.19–1.81 (m, 3H); ^{13}C NMR (75 MHz, $CDCl_3$) δ 200.2 (d, *J* = 19.5 Hz), 141.8, 135.8, 132.3, 130.1, 129.2 (d, *J* =7.5 Hz), 126.7, 94.7 (d, *J* = 183.0 Hz), 34.2, 30.5 (d, *J* = 21.0 Hz), 22.9 (d, *J* = 8.2 Hz); ^{19}F NMR (376 MHz, $CDCl_3$) δ −182.6; HRMS (ESI) m/z 201.1920 $(M+Na)^+$, calc. for $C_{10}H_9O_2FNa^+$ 201.1921.

The ee was determined by HPLC analysis: CHIRALPAK IE (4.6 mm i.d. x 250 mm); Hexane/2-propanol = 90/10; flow rate 1.0 mL/min; 25 ºC; 254 nm; retention time: 11.9 min (minor) and 11.0 min (major).

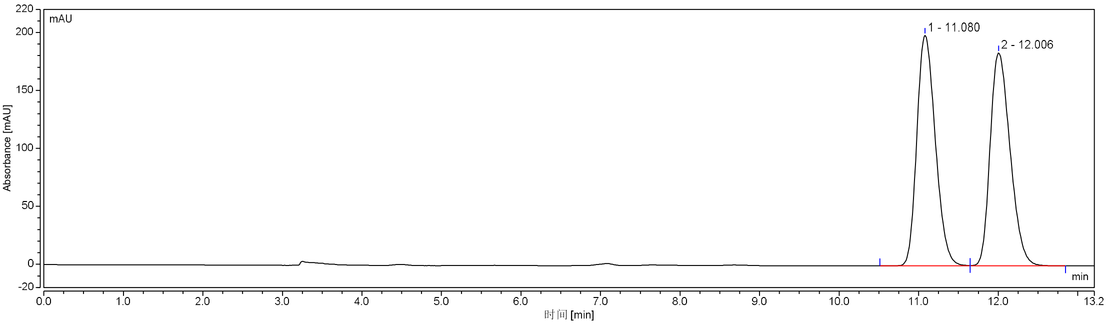

Entry	Retention Time	Area	Height	%Area
1	11.080	53.7328	198.94	50.13
2	12.006	53.4511	183.79	49.87

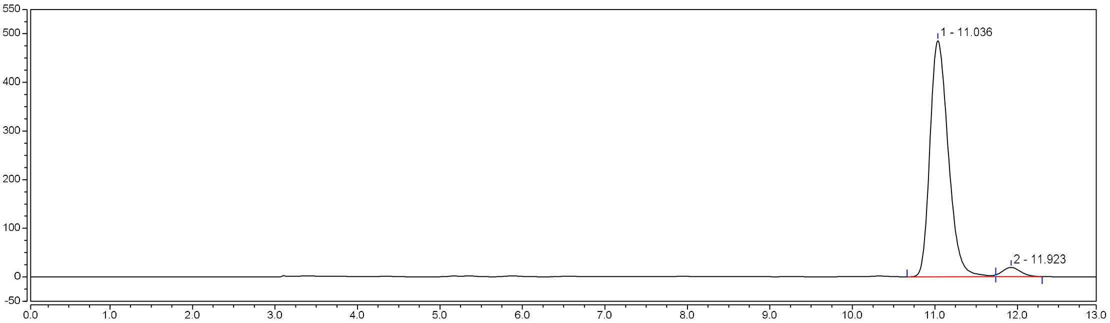

Entry	Retention Time	Area	Height	%Area
1	11.036	125.1085	485.78	96.28
2	11.923	4.8349	18.91	3.72

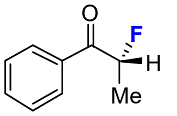

2za: colorless oil; 14.0 mg, 92% yield; 82% ee; $[\alpha]_D^{22}$ –55.1 (*c* 1.0, $CHCl_3$); ^{1}H NMR (300 MHz, $CDCl_3$) δ 7.95 (d, *J* = 7.8 Hz, 2H), 7.57 (t, *J* = 7.4 Hz, 1H), 7.45 (t, *J* = 7.6 Hz, 2H), 5.70 (dq, *J* = 48.6, 6.8 Hz, 1H), 1.63 (dd, *J* = 24.0, 6.8 Hz, 3H); ^{13}C NMR (75 MHz, $CDCl_3$) δ 196.8 (d, *J* = 19.4 Hz), 133.9 (d, *J* = 0.8 Hz), 133.7, 128.8 (d, *J* = 3.6 Hz), 128.6, 90.0 (d, *J* = 179.9 Hz), 18.2 (d, *J* = 22.8 Hz); ^{19}F NMR (376 MHz, $CDCl_3$) δ −191.7; HRMS (ESI) m/z 175.0533 $(M+Na)^+$, calc. for $C_9H_9OFNa^+$ 175.0529.

The ee was determined by HPLC analysis: CHIRALPAK AS-H (4.6 mm i.d. x 250 mm); Hexane/2-propanol = 98/2; flow rate 1.0 mL/min; 25 °C; 254 nm; retention time: 7.0 min (major) and 9.6 min (minor).

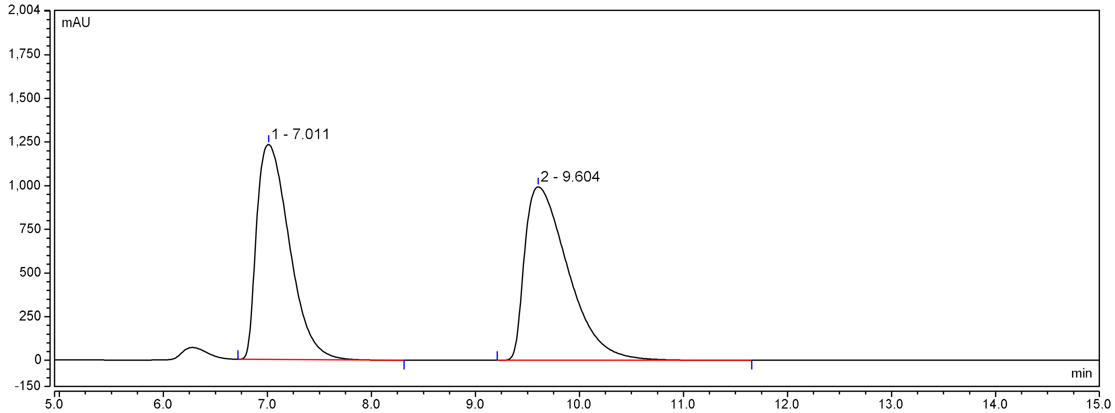

Entry	Retention Time	Area	Height	%Area
1	7.011	479.1758	1230.89	51.68
2	9.604	448.0692	992.85	48.32

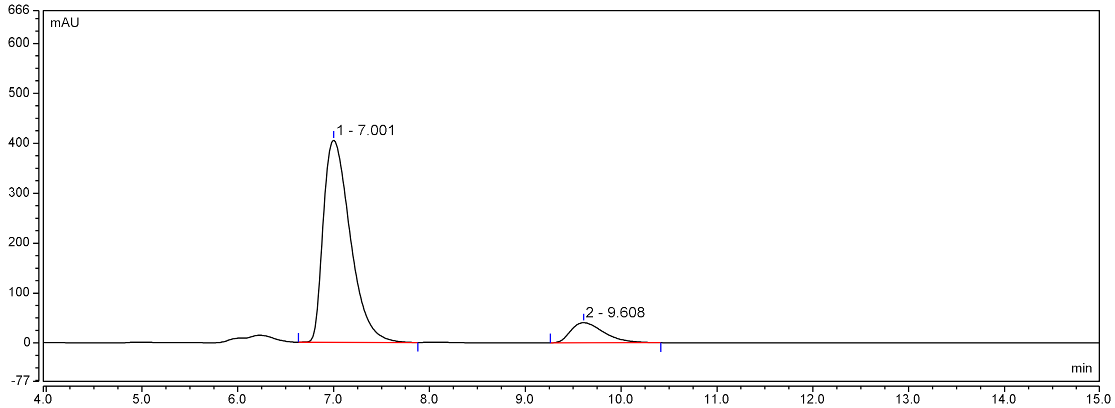

Entry	Retention Time	Area	Height	%Area
1	7.001	132.1674	404.57	90.81
2	9.608	13.3826	37.24	9.19

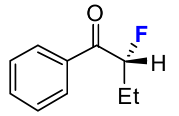

2zb: colorless oil; 13.4 mg, 81% yield; 90% ee; $[\alpha]_D^{22}$ –37.1 (*c* 1.0, $CHCl_3$); ^1H NMR (300 MHz, $CDCl_3$) δ 7.94 (d, *J* = 7.6 Hz, 2H), 7.58 (t, *J* = 7.4 Hz, 1H), 7.46 (t, *J* = 7.6 Hz, 2H), 5.50 (ddd, *J* = 49.3, 7.5, 4.7 Hz, 1H), 2.17 – 1.85 (m, 2H), 1.06 (t, *J* = 7.4 Hz, 3H); ^{13}C NMR (75 MHz, $CDCl_3$) δ 196.7 (d, *J* = 19.4 Hz), 134.3 (d, *J* = 0.7 Hz), 133.62, 128.7 (d, *J* = 3.8 Hz), 128.6, 94.6 (d, *J* = 183.4 Hz), 26.0 (d, *J* = 21.7 Hz), 8.9 (d, *J* = 4.5 Hz); ^{19}F NMR (376 MHz, $CDCl_3$) δ –191.0; HRMS (ESI) m/z 189.0688 $(M+Na)^+$, calc. for $C_{10}H_{11}OFNa^+$ 189.0686.

The ee was determined by HPLC analysis: CHIRALPAK IE−CHIRALPAK IE (4.6 mm i.d. x 250 mm); Hexane/2-propanol = 98/2; flow rate 1.0 mL/min; 25 °C; 254 nm; retention time: 14.1 min (major) and 15.8 min (minor).

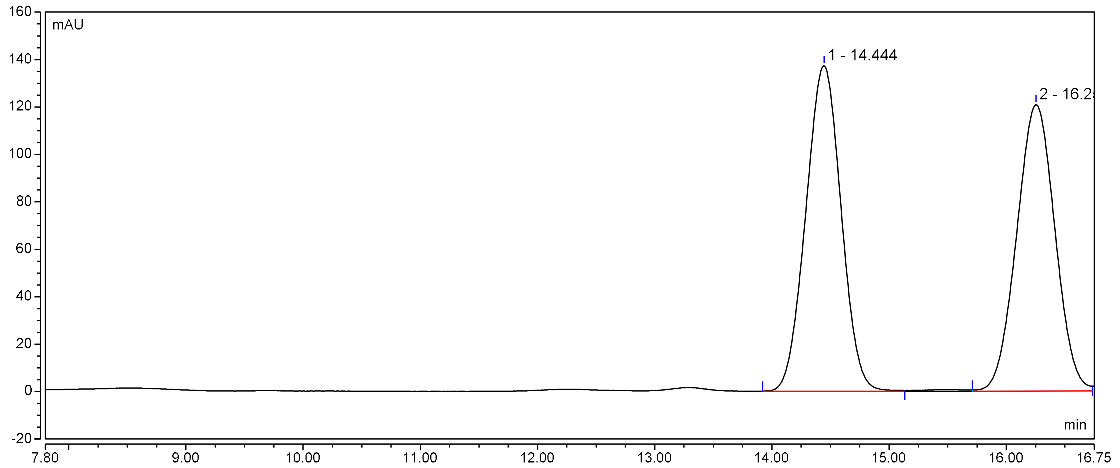

Entry	Retention Time	Area	Height	%Area
1	14.444	47.8291	137.24	51.16
2	16.254	45.6551	120.76	48.84

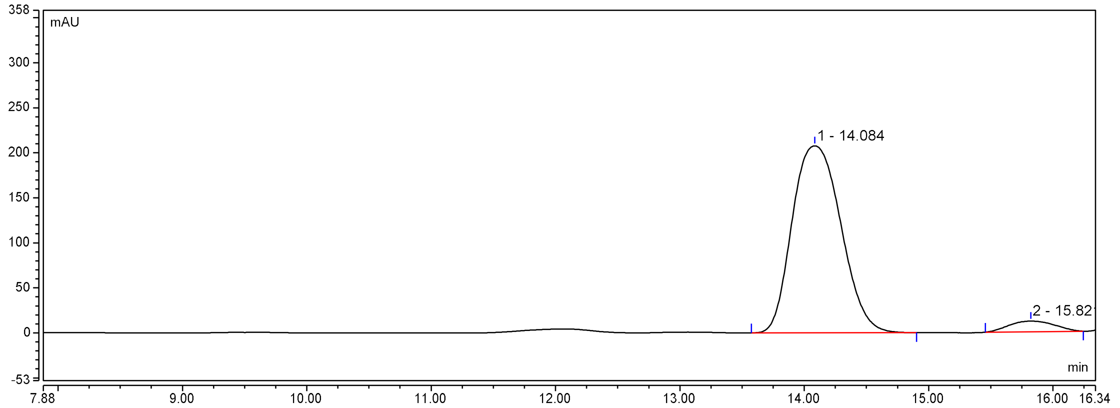

Entry	Retention Time	Area	Height	%Area
1	14.084	94.3707	207.68	95.00
2	15.821	4.9624	11.74	5.00

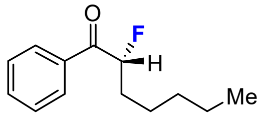

2zc: colorless oil; 18.3 mg, 88% yield; 83% ee; $[\alpha]_D^{22}$ −61.5 (*c* 1.0, $CHCl_3$); ^1H NMR (300 MHz, $CDCl_3$) δ 7.96 (d, *J* = 7.6 Hz, 2H), 7.60 (t, *J* = 7.4 Hz, 1H), 7.48 (t, *J* = 7.6 Hz, 2H), 5.56 (dt, *J* = 12.4, 5.9 Hz, 1H), 2.06 – 1.83 (m, 2H), 1.53 (d, *J* = 7.1 Hz, 2H), 1.38 – 1.27 (m, 4H), 0.89 (t, *J* = 6.8 Hz, 3H); ^{13}C NMR (75 MHz, $CDCl_3$) δ 197.0 (d, *J* = 19.6 Hz), 134.4 (d, *J* = 0.8 Hz), 133.7, 128.8 (d, *J* = 3.8 Hz), 128.7, 93.9 (d, *J* = 183.1 Hz), 32.7 (d, *J* = 21.3 Hz), 31.3, 24.4 (d, *J* = 3.1 Hz), 22.4, 13.9; ^{19}F NMR (376 MHz, $CDCl_3$) δ −189.5; HRMS (ESI) m/z 231.1152 $(M+Na)^+$, calc. for $C_{13}H_{17}OFNa^+$ 231.1155.

The ee was determined by HPLC analysis: CHIRALPAK AS-H (4.6 mm i.d. x 250 mm); Hexane/2-propanol = 98/2; flow rate 1.0 mL/min; 25 ºC; 254 nm; retention time: 4.9 min (major) and 7.9 min (minor).

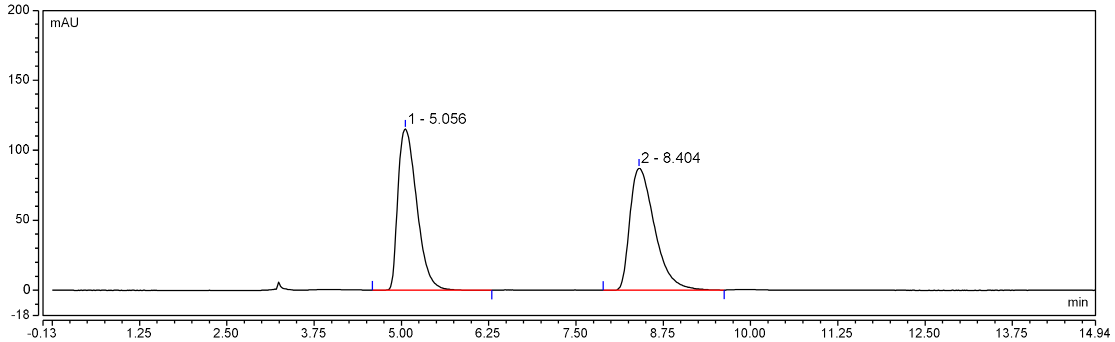

Entry	Retention Time	Area	Height	%Area
1	5.506	35.8924	114.95	50.26
2	8.404	35.5269	86.99	49.74

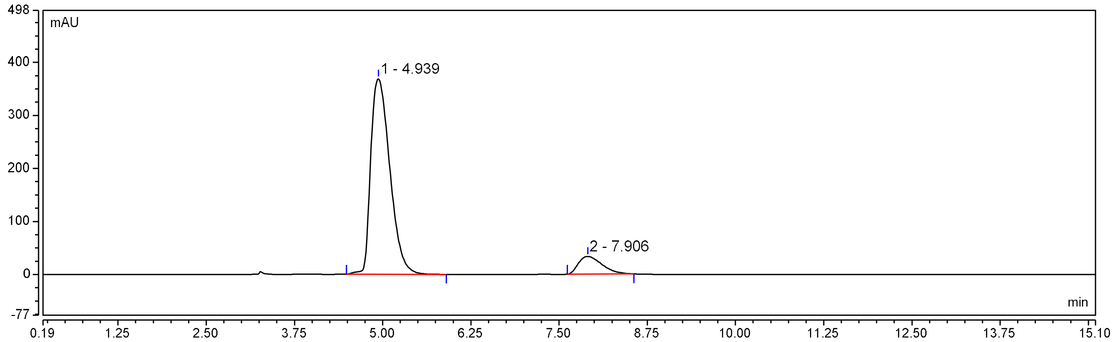

Entry	Retention Time	Area	Height	%Area
1	4.939	115.3989	369.10	91.69
2	7.906	10.4575	30.33	8.31

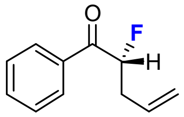

2zd: colorless oil; 15.8 mg, 89% yield; 90% ee; $[\alpha]_D^{22}$ –46.2 (*c* 1.0, $CHCl_3$); ^{1}H NMR (300 MHz, $CDCl_3$) δ 7.96 (d, *J* = 7.7 Hz, 2H), 7.61 (t, *J* = 7.4 Hz, 1H), 7.48 (t, *J* = 7.6 Hz, 2H), 5.88 (ddt, *J* = 17.3, 10.4, 6.9 Hz, 1H), 5.61 (ddd, *J* = 48.9, 7.3, 4.8 Hz, 1H), 5.20 (d, *J* = 6.4 Hz, 1H), 5.15 (s, 1H), 2.88 – 2.58 (m, 2H); ^{13}C NMR (75 MHz, $CDCl_3$) δ 196.1 (d, *J* = 19.5 Hz), 134.3, 133.8, 131.4 (d, *J* = 3.8 Hz), 128.9 (d, *J* = 3.9 Hz), 128.7, 92.8 (d, *J* = 185.1 Hz), 36.8 (d, *J* = 21.5 Hz); ^{19}F NMR (376 MHz, $CDCl_3$) δ −181.5; HRMS (ESI) m/z 201.0683 $(M+Na)^+$, calc. for $C_{11}H_{11}OFNa^+$ 201.0686.

The ee was determined by HPLC analysis: CHIRALPAK AS-H (4.6 mm i.d. x 250 mm); Hexane/2-propanol = 98/2; flow rate 1.0 mL/min; 25 ºC; 254 nm; retention time: 6.1 min (major) and 10.4 min (minor).

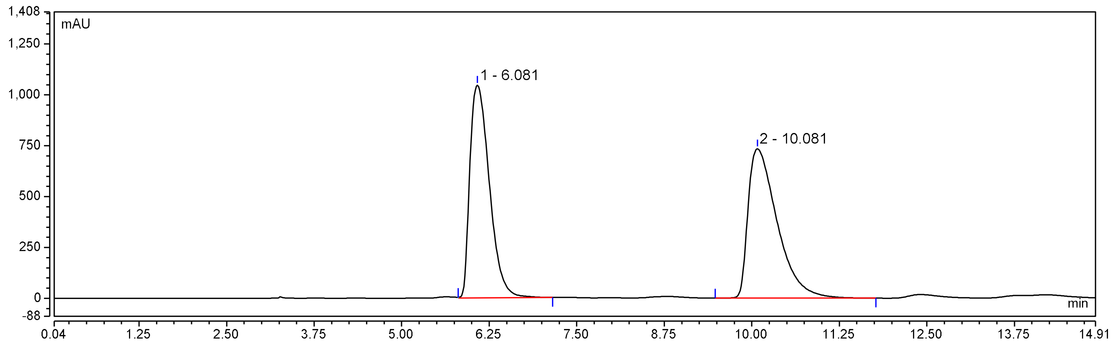

Entry	Retention Time	Area	Height	%Area
1	6.081	338.0516	1045.45	48.93
2	10.081	352.8729	734.21	51.07

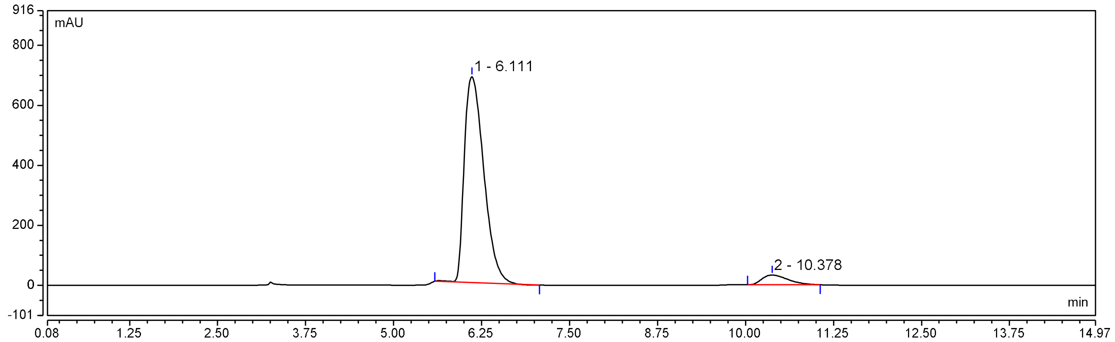

Entry	Retention Time	Area	Height	%Area
1	6.111	232.0810	695.35	95.15
2	10.387	11.8350	30.14	4.85

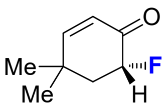

2ze: white solid; Mp 46.3–47.9 ºC; 10.6 mg, 75% yield; 90% ee; $[\alpha]_D^{22}$ –30.2 (*c* 1.0, $CHCl_3$); ^{1}H NMR (300 MHz, $CDCl_3$) δ 6.65 (d, *J* = 10.0 Hz, 1H), 5.84 (dd, *J* = 10.0, 4.4 Hz, 1H), 5.06 (ddd, *J* = 47.9, 13.2, 5.7 Hz, 1H), 2.24 (dt, *J* = 11.9, 5.9 Hz, 1H), 2.04 (dd, *J* = 22.4, 12.7 Hz, 1H), 1.22 (d, *J* = 13.3 Hz, 6H); ^{13}C NMR (75 MHz, $CDCl_3$) δ 194.5 (d, *J* = 14.7 Hz), 159.6 (d, *J* = 1.6 Hz), 124.4, 88.0 (d, *J* = 186.4 Hz), 42.2 (d, *J* = 16.8 Hz), 35.5 (d, *J* = 11.0 Hz), 30.5, 26.1; ^{19}F NMR (376 MHz, $CDCl_3$) δ -195.6 (six peaks), -195.7 (three peaks), -195.8 (three peaks); HRMS (ESI) m/z 165.0684 $(M+Na)^+$, calc. for $C_8H_{11}OFNa^+$ 165.0686.

The ee was determined by HPLC analysis: CHIRALPAK IG (4.6 mm i.d. x 250 mm); Hexane/2-propanol = 90/10; flow rate 1.0 mL/min; 25 ºC; 230 nm; retention time: 14.0 min (major) and 15.0 min (minor).

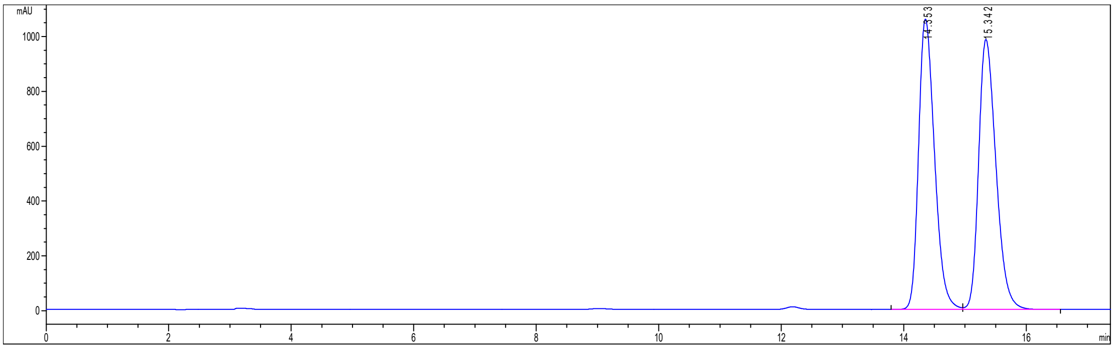

Entry	Retention Time	Area	Height	%Area
1	14.353	19164.6	1059.8	49.756
2	15.342	19352.4	985.4	50.244

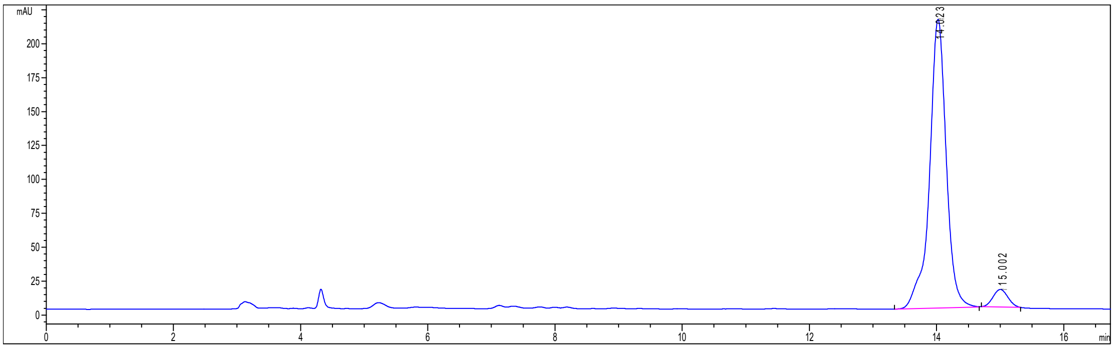

Entry	Retention Time	Area	Height	%Area
1	14.023	3890.7	213	94.860
2	15.002	210.8	13	5.140

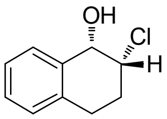

6a: white solid; Mp 80.4−81.8 ºC; 14.4 mg, 79% yield; 92% ee; > 20:1 dr; $[\alpha]_D^{22}$ −6.0 (*c* 1.0, $CHCl_3$); ^1H NMR (300 MHz, $CDCl_3$) δ 7.52–7.49 (m, 1H), 7.27–7.25 (m, 2H), 7.15 (d, *J* = 3.3 Hz, 1H), 4.86 (s, 1H), 4.56 (dd, *J* = 5.5, 2.9 Hz, 1H), 3.13 (dt, *J* = 16.7, 6.3 Hz, 1H), 2.90–2.80 (m, 1H), 2.44 (td, *J* = 14.2, 6.8 Hz, 2H), 2.23–2.19 (m, 1H); ^{13}C NMR (75 MHz, $CDCl_3$) δ 135.8, 134.8, 128.8, 128.5, 128.1, 126.5, 70.1, 63.2, 27.5, 26.6; HRMS (ESI) m/z 183.0575 $(M+H)^+$, calc. for $C_{10}H_{12}OCl^+$ 183.0577.

The ee was determined by HPLC analysis: CHIRALPAK IC (4.6 mm i.d. x 250 mm); Hexane/2-propanol = 95/5; flow rate 1.0 mL/min; 25 ºC; 210 nm; retention time: 8.9 min (minor) and 10.9 min (major).

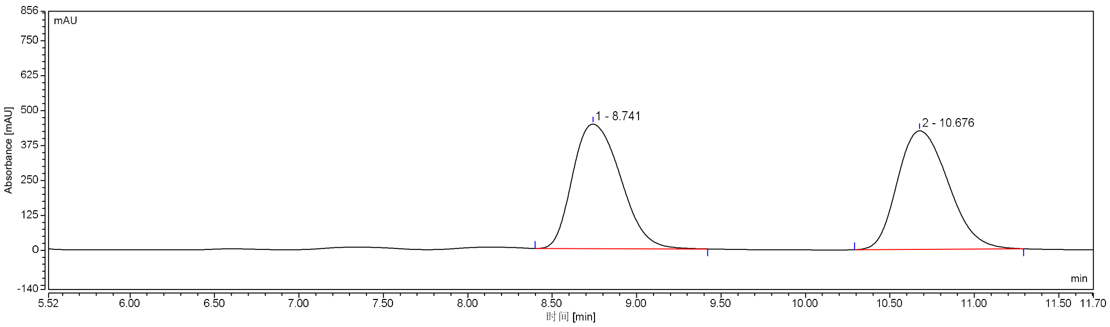

Entry	Retention Time	Area	Height	%Area
1	8.741	146.6750	445.57	49.82
2	10.676	147.7391	424.21	50.18

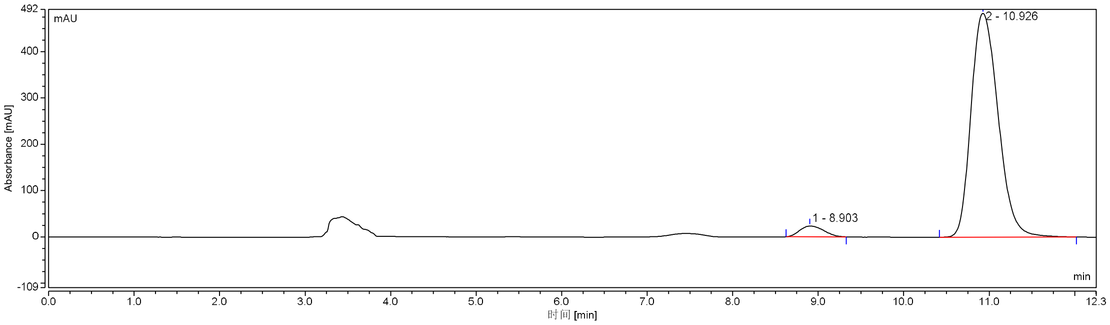

Entry	Retention Time	Area	Height	%Area
1	8.903	8.903	8.903	4.08
2	10.926	10.926	10.926	95.92

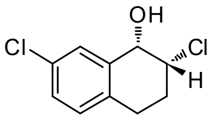

6b: white solid; Mp 84.9−86.5 °C; 13.0 mg, 60% yield; 90% ee; > 20:1 dr; $[\alpha]_D^{22}$ −56.1 (*c* 1.0, $CHCl_3$); ^{1}H NMR (300 MHz, $CDCl_3$) δ 7.51 (s, 1H), 7.20 (d, *J* = 8.2 Hz, 1H), 7.05 (d, *J* = 8.2 Hz, 1H), 4.80 (s, 1H), 4.56 (dd, *J* = 4.4, 3.0 Hz, 1H), 3.13–3.02 (m, 1H), 2.78 (dt, *J* = 17.3, 5.9 Hz, 1H), 2.47–2.34 (m, 2H), 2.24–2.15 (m, 1H); ^{13}C NMR (75 MHz, $CDCl_3$) δ 137.7, 133.2, 132.1, 129.9, 128.3, 128.2, 69.7, 62.7, 27.6, 25.4; HRMS (ESI) m/z 239.0003 $(M+Na)^+$, calc. for $C_{10}H_{10}ONaCl^+$ 239.0006.

The ee was determined by HPLC analysis: CHIRALPAK IC (4.6 mm i.d. x 250 mm); Hexane/2-propanol = 95/5; flow rate 1.0 mL/min; 25 °C; 210 nm; retention time: 8.2 min (minor) and 11.2 min (major).

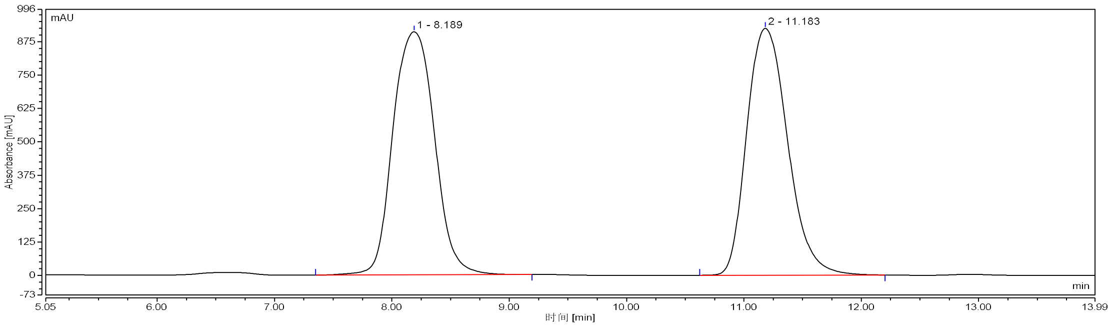

Entry	Retention Time	Area	Height	%Area
1	8.189	372.8273	909.62	49.96
2	11.183	373.3544	923.61	50.04

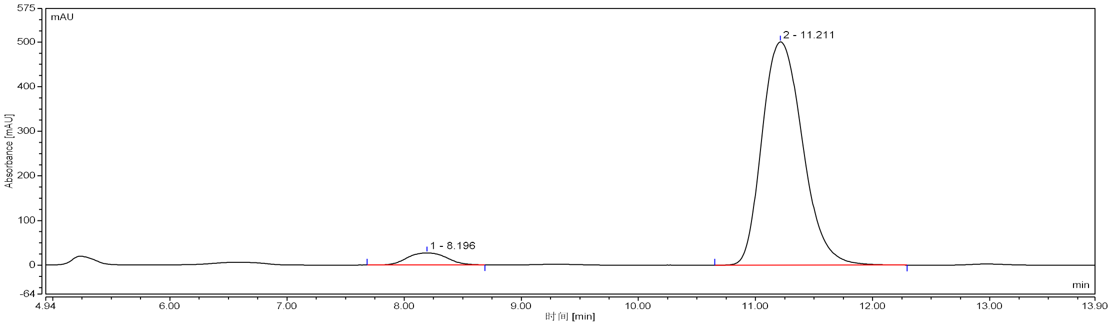

Entry	Retention Time	Area	Height	%Area
1	8.196	10.4827	26.91	4.96
2	11.211	200.6826	500.11	95.04

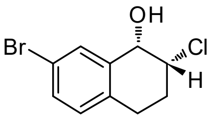

6c: white solid; Mp 90.2−92.1 °C; 16.0 mg, 61% yield; 84% ee; > 20:1 dr; $[\alpha]_D^{22}$ −46.6 (*c* 1.0, $CHCl_3$); ^1H NMR (300 MHz, $CDCl_3$) δ 7.67 (d, *J* = 1.4 Hz, 1H), 7.35 (dd, *J* = 8.2, 1.9 Hz, 1H), 6.99 (d, *J* = 8.2 Hz, 1H), 4.81 (d, *J* = 3.0 Hz, 1H), 4.65–4.46 (m, 1H), 3.11–3.01 (m, 1H), 2.76 (dt, *J* = 17.4, 6.0 Hz, 1H), 2.40 (td, *J* = 13.6, 5.9 Hz, 1H), 2.25–2.15 (m, 2H); ^{13}C NMR (75 MHz, $CDCl_3$) δ 138.1, 133.8, 131.3, 131.1, 130.2, 120.1, 69.7, 62.7, 27.6, 25.6; HRMS (ESI) m/z 282.9504 $(M+Na)^+$, calc. for $C_{10}H_{10}ONaClBr^+$ 282.9501.

The ee was determined by HPLC analysis: CHIRALPAK IC (4.6 mm i.d. x 250 mm); Hexane/2-propanol = 95/5; flow rate 1.0 mL/min; 25 °C; 210 nm; retention time: 8.4 min (minor) and 11.7 min (major).

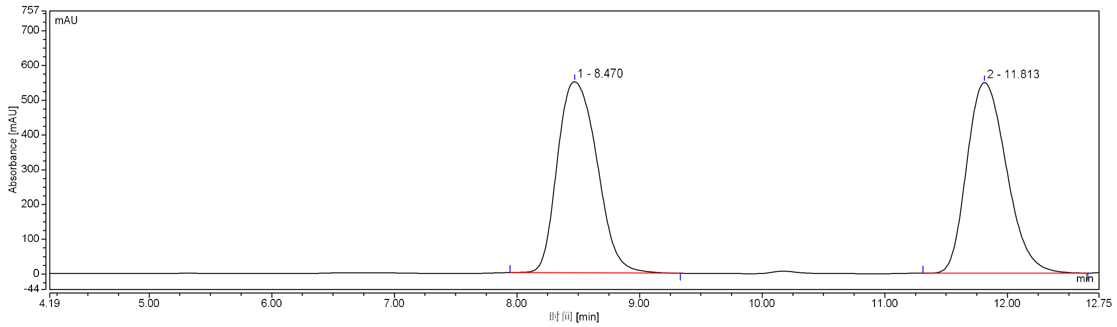

Entry	Retention Time	Area	Height	%Area
1	8.470	205.9142	549.95	50.06
2	11.813	205.4494	548.22	49.94

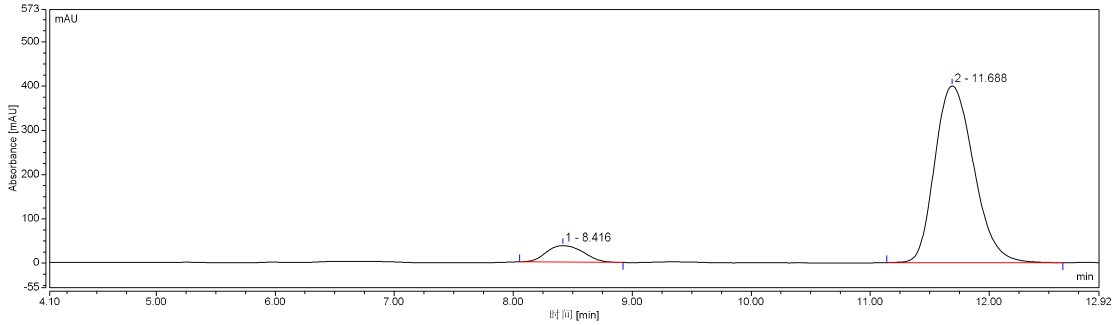

Entry	Retention Time	Area	Height	%Area
1	8.416	13.3986	37.15	8.13
2	11.688	151.3247	399.00	91.87

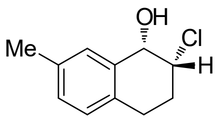

6d: white solid; Mp 103.9−105.1 ºC; 13.1 mg, 67% yield; 90% ee; > 20:1 dr; $[\alpha]_D^{22}$ −9.5 (*c* 1.0, $CHCl_3$); ^1H NMR (300 MHz, $CDCl_3$) δ 7.30 (s, 1H), 7.08–7.00 (m, 1H), 4.81 (d, *J* = 3.1 Hz, 1H), 4.53 (dt, *J* = 8.7, 3.0 Hz, 1H), 3.06 (dt, *J* = 16.8, 6.3 Hz, 1H), 2.85–2.75(m, 1H), 2.47–2.35 (m, 1H), 2.33 (s, 2H), 2.18 (dtd, *J* = 9.3, 6.6, 2.7 Hz, 1H); ^{13}C NMR (75 MHz, $CDCl_3$) δ 136.1, 135.5, 131.8, 129.3, 129.1, 128.5, 70.1, 63.4, 27.7, 26.4, 21.0; HRMS (ESI) m/z 219.0554 $(M+Na)^+$, calc. for $C_{11}H_{13}OClNa^+$ 219.0553.

The ee was determined by HPLC analysis: CHIRALPAK IC (4.6 mm i.d. x 250 mm); Hexane/2-propanol = 95/5; flow rate 1.0 mL/min; 25 ºC; 210 nm; retention time: 8.9 min (minor) and 10.9 min (major).

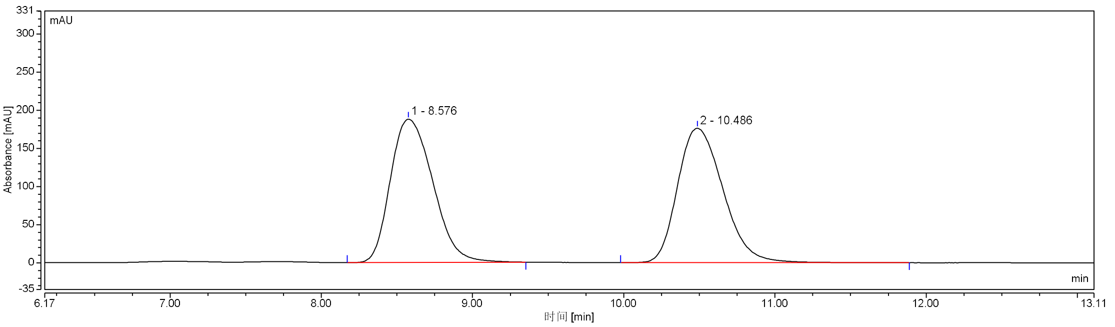

Entry	Retention Time	Area	Height	%Area
1	8.576	61.1470	187.91	49.87
2	10.486	61.4718	176.27	50.13

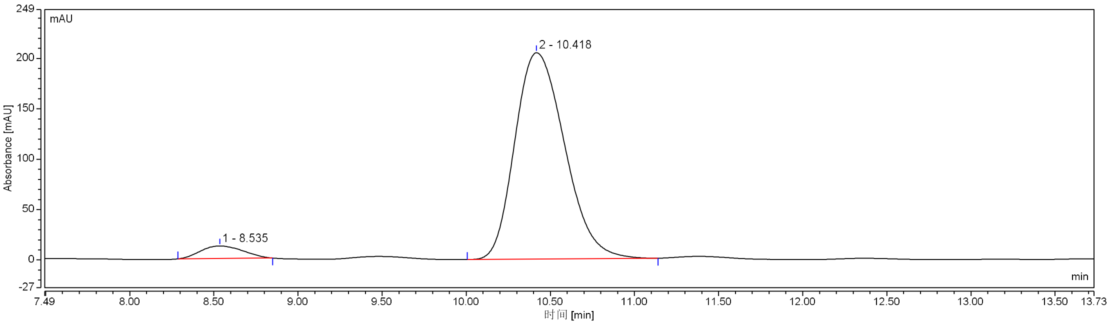

Entry	Retention Time	Area	Height	%Area
1	8.903	3.6232	12.39	4.91
2	10.926	70.2000	204.76	95.09

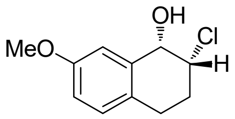

6e: white solid; Mp 94.9−96.5 ºC; 16.0 mg, 75% yield; 90% ee; > 20:1 dr; $[\alpha]_D^{22}$ −5.0 (*c* 1.0, $CHCl_3$); ^{1}H NMR (300 MHz, $CDCl_3$) δ 7.03 (d, *J* = 8.4 Hz, 2H), 6.82 (dd, *J* = 8.4, 2.4 Hz, 1H), 4.81 (s, 1H), 4.56 (dd, *J* = 4.8, 3.2 Hz, 1H), 3.80 (s, 3H), 3.09–3.00 (m, 1H), 2.76 (dt, *J* = 16.9, 6.2 Hz, 1H), 2.39 (dt, *J* = 13.9, 6.2 Hz, 2H), 2.19 (ddd, *J* = 13.7, 9.7, 4.0 Hz, 1H); ^{13}C NMR (75 MHz, $CDCl_3$) δ 158.2, 136.9, 129.6, 126.7, 115.0, 112.6, 70.2, 63.3, 55.3, 27.8, 25.4; HRMS (ESI) m/z 235.0503 $(M+Na)^+$, calc. for $C_{11}H_{13}O_2NaCl^+$ 235.0502.

The ee was determined by HPLC analysis: CHIRALPAK IE (4.6 mm i.d. x 250 mm); Hexane/2-propanol = 95/5; flow rate 1.0 mL/min; 25 ºC; 210 nm; retention time: 21.6 min (major) and 24.6 min (minor).

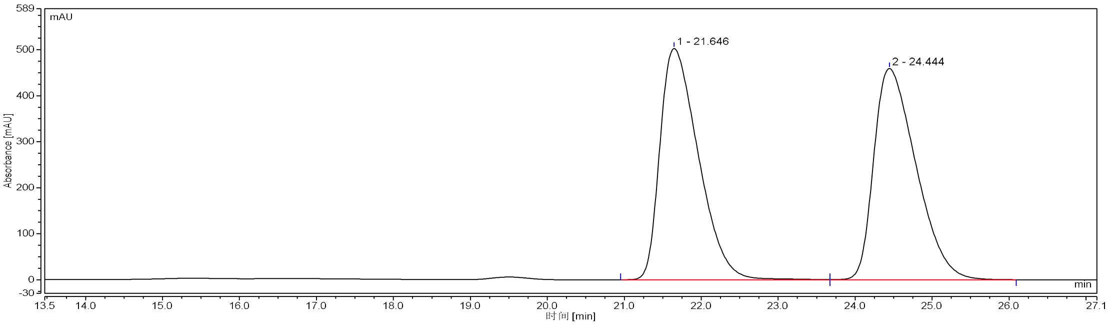

Entry	Retention Time	Area	Height	%Area
1	21.646	291.1048	502.78	49.98
2	24.444	291.2826	458.95	50.02

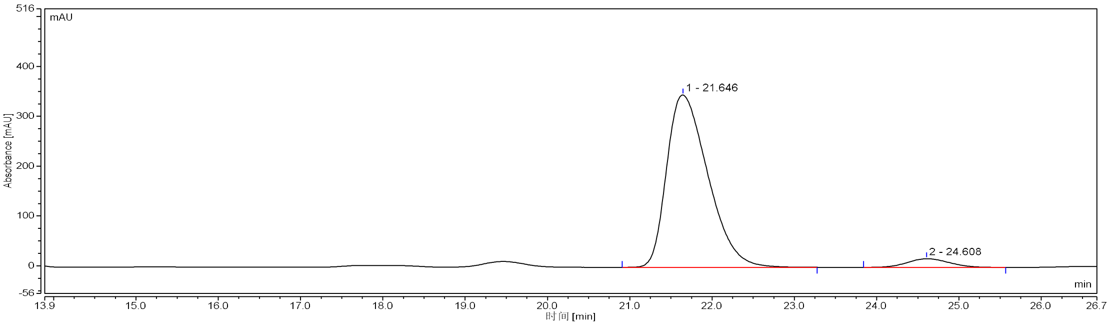

Entry	Retention Time	Area	Height	%Area
1	21.646	200.3435	345.93	95.12
2	24.608	10.2791	17.30	4.88

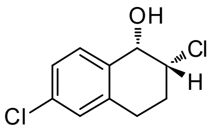

6f: white solid; Mp 133.9−136.8 ºC; 14.1 mg, 65% yield; 90% ee; > 20:1 dr; $[\alpha]_D^{22}$ −7.3 (*c* 1.0, $CHCl_3$); ^1H NMR (300 MHz, $CDCl_3$) δ 7.44 (d, *J* = 8.3 Hz, 1H), 7.20 (d, *J* = 8.3 Hz, 1H), 7.12 (s, 1H), 4.81 (d, *J* = 3.2 Hz, 1H), 4.54 (dt, *J* = 7.9, 3.0 Hz, 1H), 3.09 (dt, *J* = 17.3, 6.8 Hz, 1H), 2.80 (dt, *J* = 17.5, 6.2 Hz, 1H), 2.40 (dt, *J* = 14.1, 6.2 Hz, 2H), 2.19 (ddd, *J* = 13.9, 8.6, 2.6 Hz, 1H); ^{13}C NMR (75 MHz, $CDCl_3$) δ 136.8, 134.4, 133.8, 130.1, 128.3, 126.8, 69.6, 62.8, 27.4, 26.2; HRMS (ESI) m/z 239.0011 $(M+Na)^+$, calc. for $C_{10}H_{10}OCl_2Na^+$ 239.0006.

The ee was determined by HPLC analysis: CHIRALPAK IC (4.6 mm i.d. x 250 mm); Hexane/2-propanol = 95/5; flow rate 1.0 mL/min; 25 ºC; 210 nm; retention time: 8.0 min (minor) and 9.6 min (major).

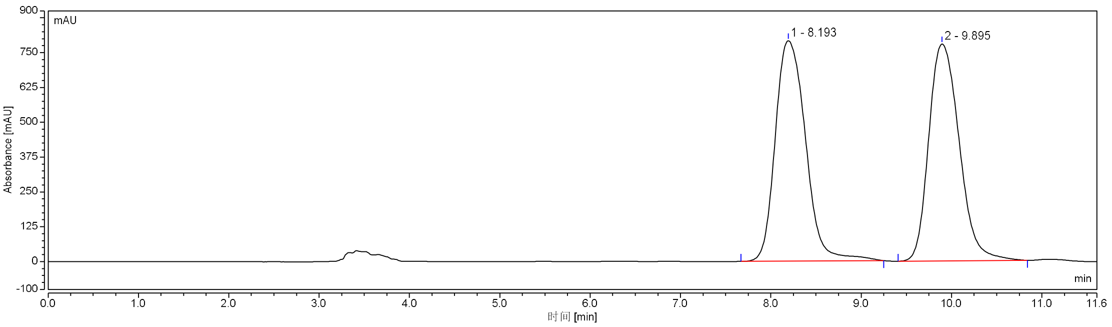

Entry	Retention Time	Area	Height	%Area
1	8.193	307.8653	791.58	50.31
2	9.895	304.1234	779.17	49.69

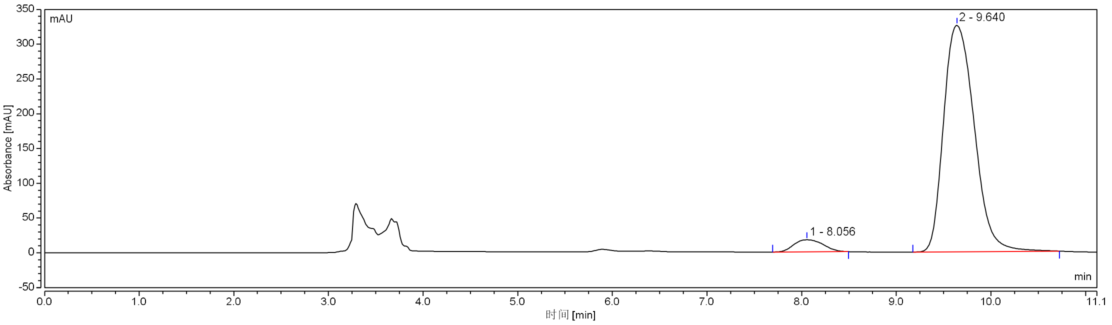

Entry	Retention Time	Area	Height	%Area
1	8.056	6.2293	17.42	4.85
2	9.640	122.1139	325.71	95.15

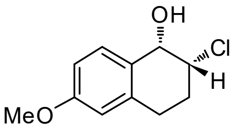

6g: white solid; Mp 115.7−117.1 ºC; 15.3 mg, 72% yield; 94% ee; > 20:1 dr; $[\alpha]_D^{22}$ +8.1 (*c* 1.0, $CHCl_3$); ^1H NMR (300 MHz, $CDCl_3$) δ 7.38 (d, *J* = 8.6 Hz, 1H), 6.80 (dd, *J* = 8.5, 2.3 Hz, 1H), 6.64 (s, 1H), 4.80 (d, *J* = 2.9 Hz, 1H), 4.50 (dt, *J* = 9.0, 2.9 Hz, 1H), 3.79 (s, 3H), 3.07 (dt, *J* = 17.3, 6.2 Hz, 1H), 2.87–2.76 (m, 1H), 2.41 (dt, *J* = 13.8, 7.4 Hz, 1H), 2.17 (dtd, *J* = 8.9, 6.3, 2.7 Hz, 2H); ^{13}C NMR (75 MHz, $CDCl_3$) δ 159.4, 136.4, 130.3, 128.1, 113.0, 112.8, 69.7, 63.4, 55.2, 27.4, 27.2; HRMS (ESI) m/z 235.0500 $(M+Na)^+$, calc. for $C_{11}H_{13}O_2NaCl^+$ 235.0502.

The ee was determined by HPLC analysis: CHIRALPAK IC (4.6 mm i.d. x 250 mm); Hexane/2-propanol = 95/5; flow rate 1.0 mL/min; 25 ºC; 210 nm; retention time: 18.0 min (major) and 19.0 min (minor).

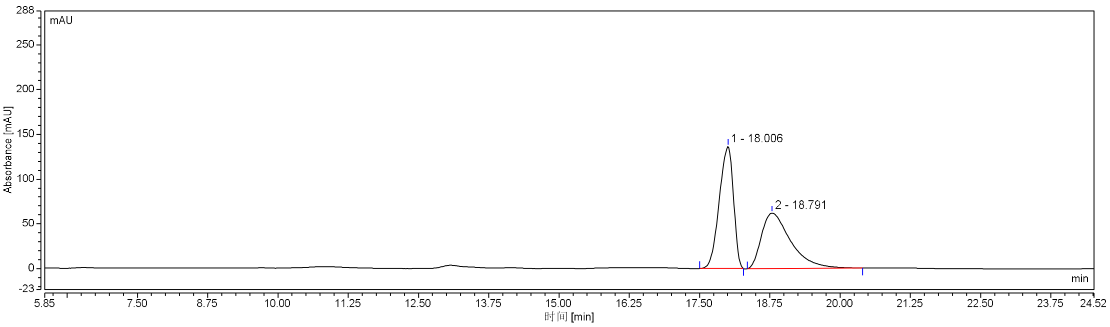

Entry	Retention Time	Area	Height	%Area
1	18.006	39.7601	136.18	51.53
2	18.791	37.3934	62.07	48.47

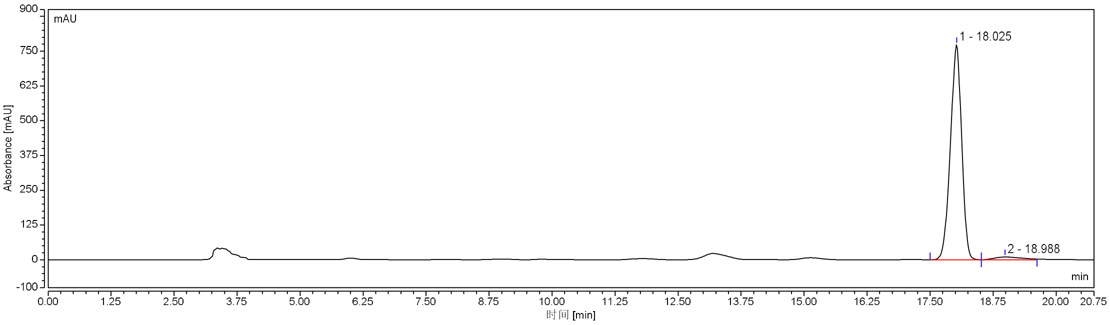

Entry	Retention Time	Area	Height	%Area
1	18.025	200.7196	773.56	97.01
2	18.988	6.1952	9.81	2.99

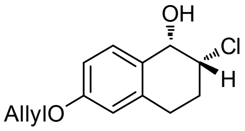

6h: white solid; Mp 92.8−93.9 ºC; 16.5 mg, 69% yield; 94% ee; > 20:1 dr; $[\alpha]_D^{22}$ +3.8 (*c* 1.0, $CHCl_3$); ^1H NMR (300 MHz, $CDCl_3$) δ 7.37 (d, *J* = 8.5 Hz, 1H), 6.81 (dd, *J* = 8.5, 2.4 Hz, 1H), 6.65 (d, *J* = 2.1 Hz, 1H), 6.04 (ddd, *J* = 22.4, 10.5, 5.3 Hz, 1H), 5.40 (dd, *J* = 17.3, 1.4 Hz, 1H), 5.28 (dd, *J* = 10.5, 1.2 Hz, 1H), 4.80 (s, 1H), 4.52–4.51 (m, 2H), 4.48 (t, *J* = 3.1 Hz, 1H), 3.06 (dt, *J* = 17.3, 6.2 Hz, 1H), 2.81 (dt, *J* = 17.3, 6.9 Hz, 1H), 2.46–2.35 (m, 2H), 2.16 (dtd, *J* = 8.9, 6.3, 2.7 Hz, 1H); ^{13}C NMR (75 MHz, $CDCl_3$) δ 158.4, 136.4, 133.1, 130.3, 128.3, 117.7, 113.9, 113.5, 69.7, 68.7, 63.3, 27.4, 27.2; HRMS (ESI) m/z 261.0653 $(M+Na)^+$, calc. for $C_{13}H_{15}O_2NaCl^+$ 261.0651.

The ee was determined by HPLC analysis: CHIRALPAK IE (4.6 mm i.d. x 250 mm); Hexane/2-propanol = 95/5; flow rate 1.0 mL/min; 25 ºC; 210 nm; retention time: 24.6 min (major) and 28.4 min (minor).

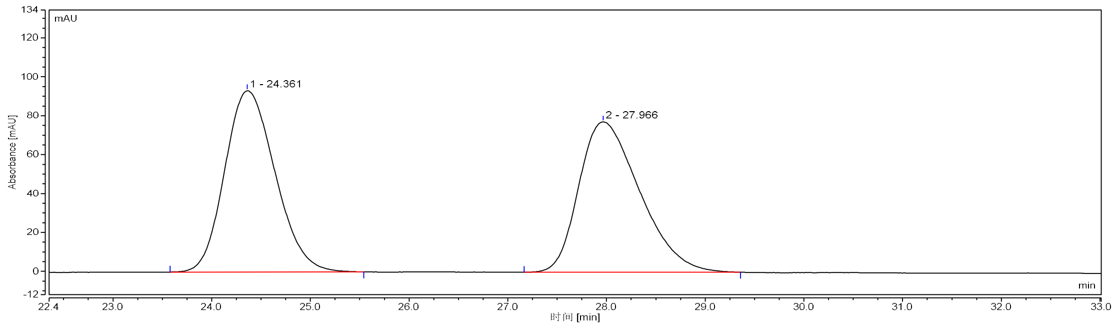

Entry	Retention Time	Area	Height	%Area
1	25.199	53.0888	86.83	49.84
2	29.038	53.4388	72.23	50.16

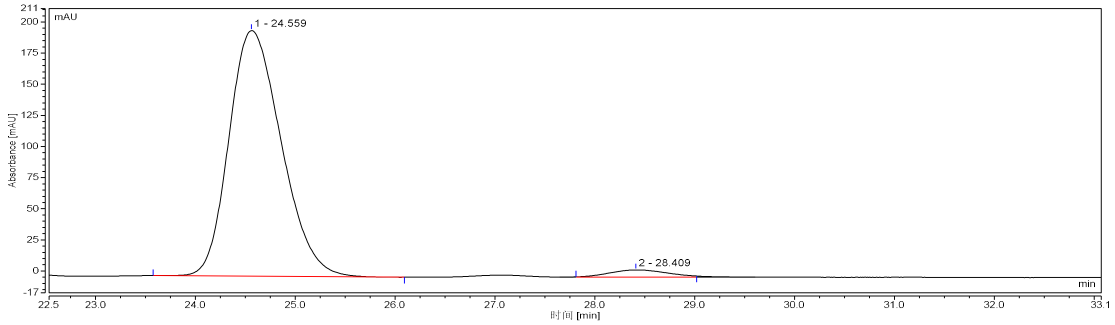

Entry	Retention Time	Area	Height	%Area
1	24.559	119.6923	197.34	96.90
2	28.409	3.8348	5.77	3.10

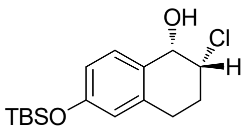

6i: colourless oil; 20.3 mg, 65% yield; 90% ee; > 20:1 dr; $[\alpha]_D^{22}$ +8.0 (*c* 1.0, $CHCl_3$) ; ^{1}H NMR (300 MHz, $CDCl_3$) δ 7.30 (d, *J* = 8.4 Hz, 1H), 6.71 (dd, *J* = 8.4, 2.4 Hz, 1H), 6.58 (d, *J* = 2.2 Hz, 1H), 4.78 (d, *J* = 2.3 Hz, 1H), 4.47 (dt, *J* = 9.3, 3.1 Hz, 1H), 3.02 (dt, *J* = 17.3, 6.1 Hz, 1H), 2.78 (dt, *J* = 17.3, 7.0 Hz, 1H), 2.47–2.34 (m, 2H), 2.14 (dtd, *J* = 9.0, 6.2, 2.8 Hz, 1H), 0.99 (s, 9H), 0.20 (s, 6H); ^{13}C NMR (75 MHz, $CDCl_3$) δ 155.5, 136.3, 130.3, 128.6, 119.4, 118.5, 69.8, 63.3, 27.3, 27.2, 25.6, 18.1, −4.4; HRMS (ESI) m/z 335.1205 $(M+Na)^+$, calc. for $C_{16}H_{25}O_2NaCl^+$ 335.1203.

The ee was determined by HPLC analysis: CHIRALPAK IC (4.6 mm i.d. x 250 mm); Hexane/2-propanol = 95/5; flow rate 1.0 mL/min; 25 ºC; 210 nm; retention time: 12.4 min (minor) and 13.6 min (major).

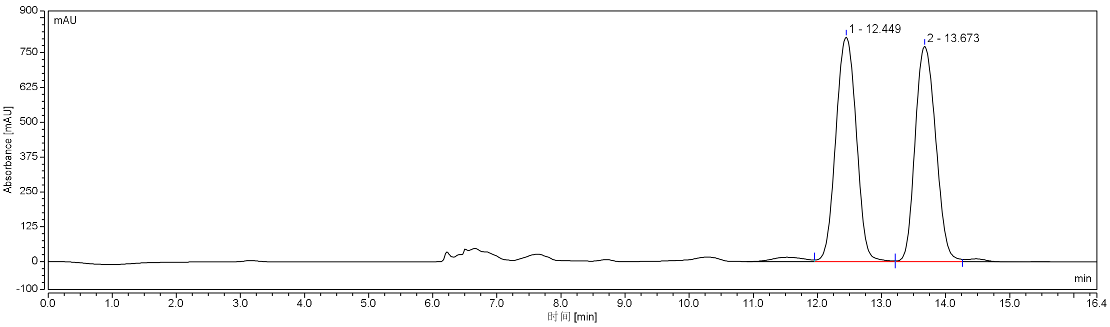

Entry	Retention Time	Area	Height	%Area
1	12.449	298.9512	805.95	50.93
2	13.673	288.0571	772.92	49.07

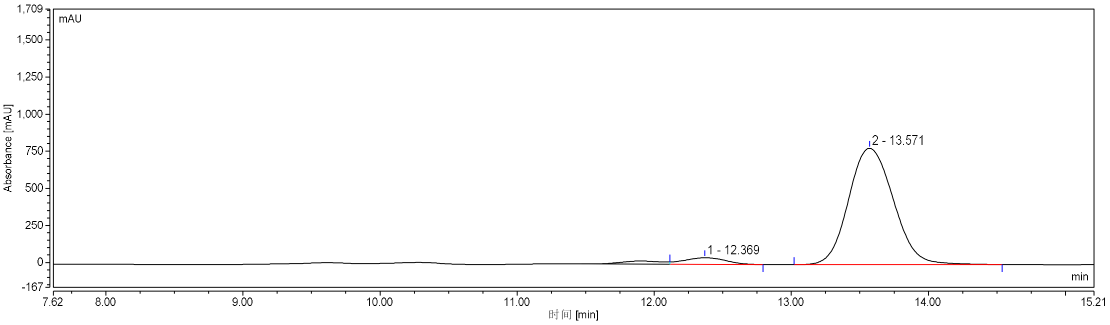

Entry	Retention Time	Area	Height	%Area
1	12.369	15.3647	43.85	4.93
2	13.571	296.4483	783.38	95.07

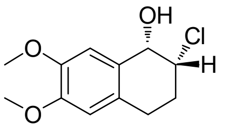

6j: colourless oil; 17.7 mg, 73% yield; 93% ee; > 20:1 dr; $[\alpha]_D^{22}$ −4.7 (*c* 1.0, $CHCl_3$); ^{1}H NMR (300 MHz, $CDCl_3$) δ 6.96 (s, 1H), 6.57 (s, 1H), 4.78 (d, *J* = 2.6 Hz, 1H), 4.51 (dt, *J* = 8.5, 2.8 Hz, 1H), 3.86 (s, 3H), 3.84 (s, 3H), 3.01 (dt, *J* = 16.8, 6.4 Hz, 1H), 2.75 (dt, *J* = 16.9, 6.5 Hz, 1H), 2.38 (dt, *J* = 14.5, 6.6 Hz, 2H), 2.15 (dtd, *J* = 9.0, 6.6, 2.4 Hz, 1H); ^{13}C NMR (75 MHz, $CDCl_3$) δ 148.9, 147.7, 127.6, 127.1, 111.1, 110.7, 69.8, 63.3, 55.8, 55.8, 27.6, 26.3; HRMS (ESI) m/z 265.0602 $(M+Na)^+$, calc. for $C_{10}H_9O_2FNa^+$ 265.0599.

The ee was determined by HPLC analysis: CHIRALPAK IE (4.6 mm i.d. x 250 mm); Hexane/2-propanol = 95/5; flow rate 1.0 mL/min; 25 ºC; 210 nm; retention time: 16.4 min (major) and 19.9 min (minor).

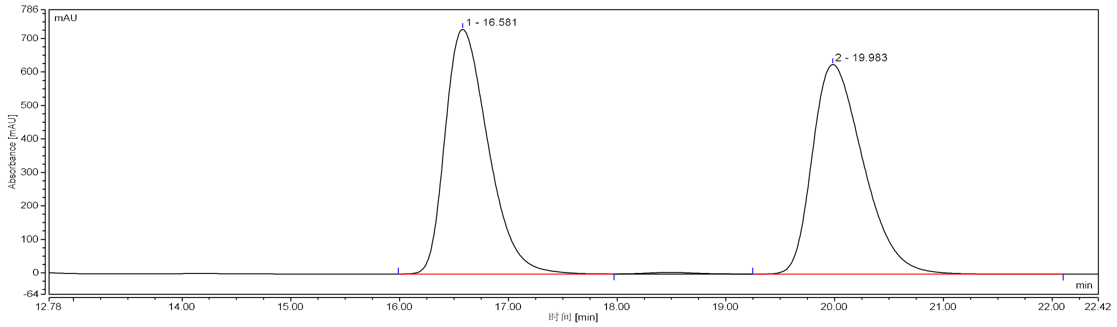

Entry	Retention Time	Area	Height	%Area
1	16.581	335.1269	730.99	49.63
2	19.983	340.0737	626.04	50.37

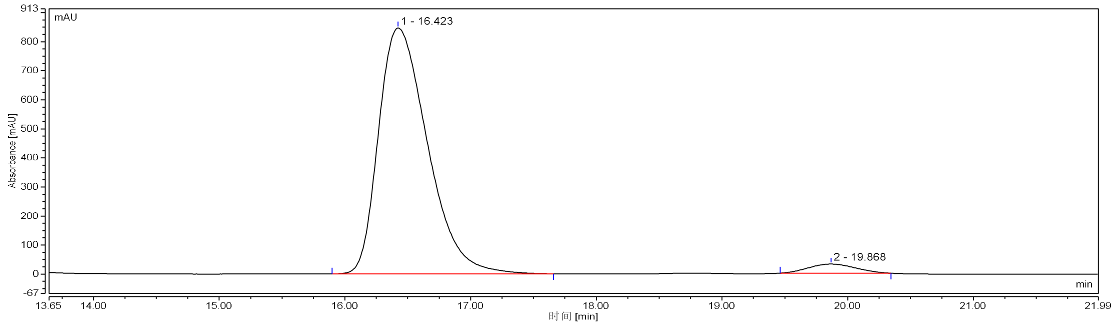

Entry	Retention Time	Area	Height	%Area
1	16.423	381.9103	846.15	96.55
2	19.868	13.6577	31.54	3.45

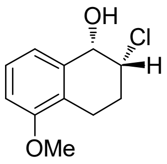

6k: white solid; Mp 119.8−121.1 °C; 17.4 mg, 82% yield; 91% ee; > 20:1 dr; $[\alpha]_D^{22}$ −13.0 (*c* 1.0, $CHCl_3$); ^{1}H NMR (300 MHz, $CDCl_3$) δ 7.22 (d, *J* = 7.9 Hz, 1H), 7.11 (d, *J* = 7.7 Hz, 1H), 6.79 (d, *J* = 8.1 Hz, 1H), 4.83 (d, *J* = 3.1 Hz, 1H), 4.51 (dt, *J* = 8.7, 3.0 Hz, 1H), 3.83 (s, 3H), 2.96 (dt, *J* = 18.1, 6.6 Hz, 1H), 2.70 (dt, *J* = 18.1, 6.7 Hz, 1H), 2.47–2.35 (m, 1H), 2.18 (dtd, *J* = 9.1, 6.5, 2.5 Hz, 2H); ^{13}C NMR (75 MHz, $CDCl_3$) δ 156.8, 137.0, 127.1, 124.0, 120.6, 109.2, 70.1, 63.0, 55.3, 27.0, 21.2; HRMS (ESI) m/z 235.0499 $(M+Na)^+$, calc. for $C_{11}H_{13}O_2NaCl^+$ 235.0502.

The ee was determined by HPLC analysis: CHIRALPAK IC (4.6 mm i.d. x 250 mm); Hexane/2-propanol = 95/5; flow rate 1.0 mL/min; 25 °C; 210 nm; retention time: 10.7 min (minor) and 12.9 min (major).

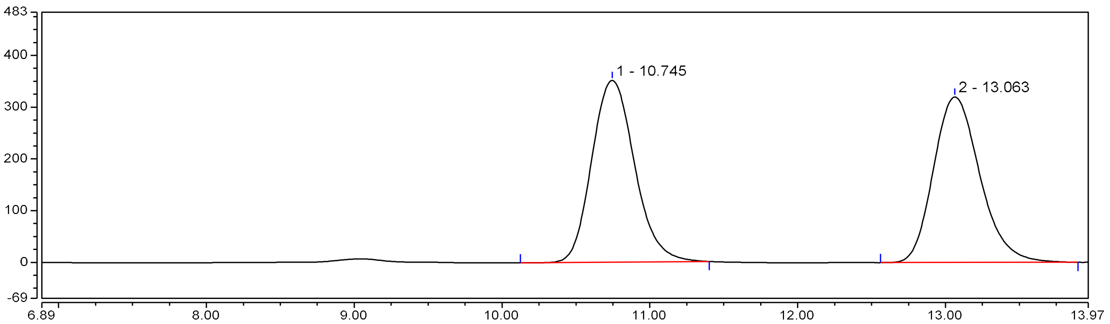

Entry	Retention Time	Area	Height	%Area
1	10.745	118.4533	351.74	50.41
2	13.063	116.5267	319.96	49.59

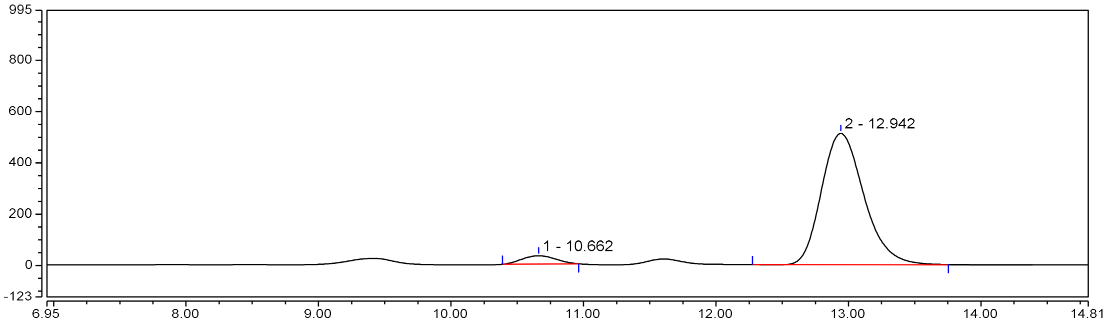

Entry	Retention Time	Area	Height	%Area
1	10.662	9.4449	32.01	4.73
2	12.942	190.1631	512.34	95.27

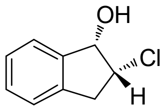

6l: white solid; Mp 111.4−112.6 ºC; 10.9 mg, 65% yield; 87% ee; >20:1 dr; $[\alpha]_D^{22}$ −7.4 (*c* 1.0, $CHCl_3$) ; ^1H NMR (300 MHz, $CDCl_3$) δ 7.49–7.46 (m, 1H), 7.34–7.28 (m, 3H), 5.18 (d, *J* = 4.5 Hz, 1H), 4.83 (dd, *J* = 8.4, 5.0 Hz, 1H), 3.33 (qd, *J* = 16.6, 4.2 Hz, 2H), 2.53 (s, 1H); ^{13}C NMR (75 MHz, $CDCl_3$) δ 141.4, 138.9, 128.8, 127.5, 125.0, 124.7, 76.4, 65.9, 39.7; HRMS (ESI) m/z 191.0242 $(M+Na)^+$, calc. for $C_{10}H_9O_2FNa^+$ 191.0240.

The ee was determined by HPLC analysis: CHIRALPAK IC (4.6 mm i.d. x 250 mm); Hexane/2-propanol = 95/5; flow rate 1.0 mL/min; 25 ºC; 210 nm; retention time: 8.5 min (minor) and 10.3 min (major).

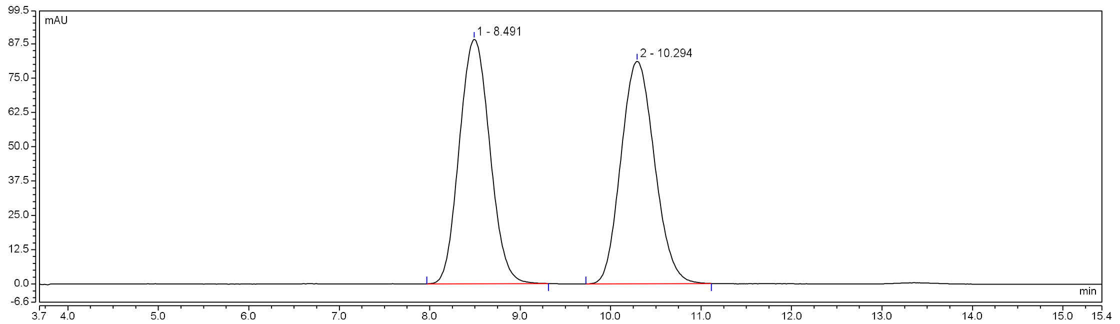

Entry	Retention Time	Area	Height	%Area
1	8.491	34.78	34.7752	49.93
2	10.294	34.87	34.8705	50.07

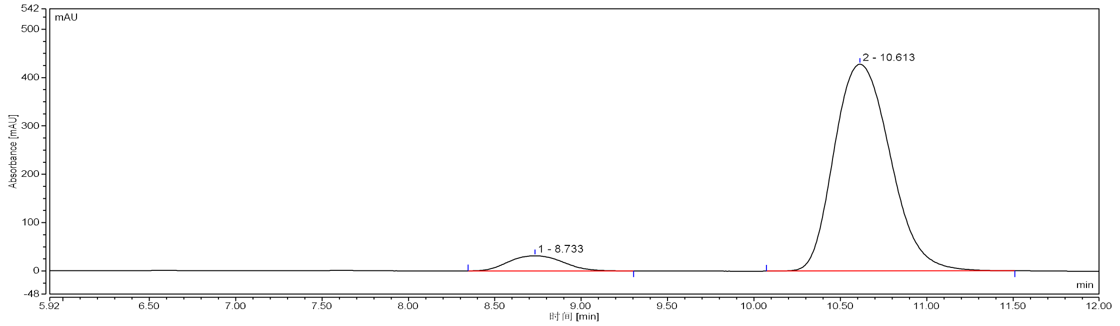

Entry	Retention Time	Area	Height	%Area
1	8.478	11.4190	31.25	6.63
2	10.294	160.9188	427.47	93.37

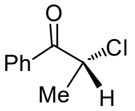

5m: colorless oil; 12.3 mg, 73% yield; 82% ee; $[\alpha]_D^{22}$ −28.2 (*c* 1.0, $CHCl_3$); ^{1}H NMR (300 MHz, $CDCl_3$) δ 8.06 – 7.97 (m, 2H), 7.59 (t, *J* = 7.4 Hz, 1H), 7.48 (t, *J* = 7.5 Hz, 2H), 5.25 (q, *J* = 6.7 Hz, 1H), 1.74 (d, *J* = 6.7 Hz, 3H); ^{13}C NMR (75 MHz, $CDCl_3$) δ 193.6, 134.1, 133.7, 128.9, 128.7, 52.8, 19.9; HRMS (ESI) m/z 191.0236 $(M+Na)^+$, calc. for $C_9H_9OClNa^+$ 191.0234.

The ee was determined by HPLC analysis: CHIRALPAK IE−CHIRALPAK IE (4.6 mm i.d. x 250 mm); Hexane/2-propanol = 98/2; flow rate 1.0 mL/min; 25 °C; 254 nm; retention time: 12.6 min (major) and 13.6 min (minor).

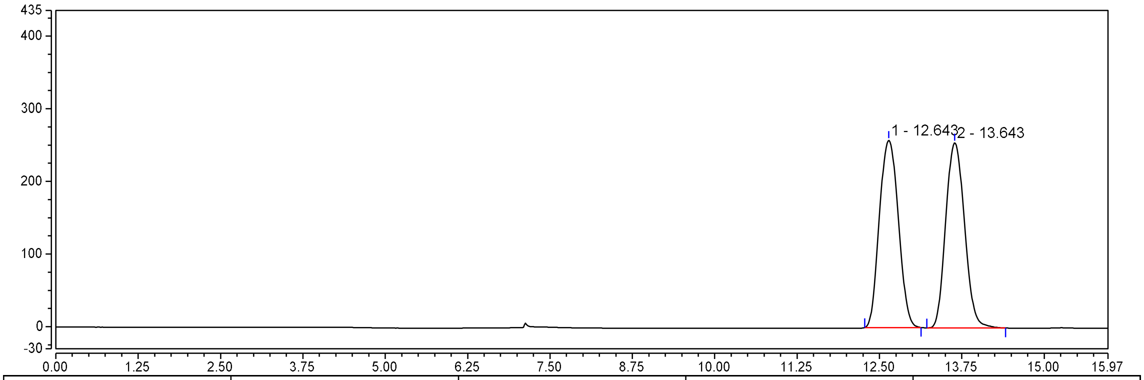

Entry	Retention Time	Area	Height	%Area
1	12.643	86.1726	257.62	50.46
2	13.643	84.5988	254.76	49.54

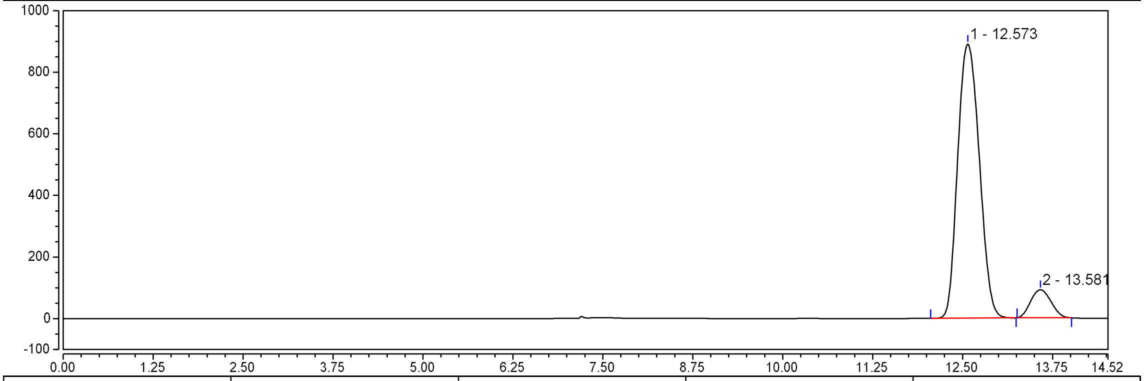

Entry	Retention Time	Area	Height	%Area
1	12.573	310.2536	889.72	91.25
2	13.581	29.7635	90.48	8.75

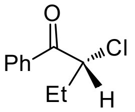

5n: colorless oil; 14.2 mg, 78% yield; 82% ee; $[\alpha]_D^{22}$ −41.3 (*c* 1.0, $CHCl_3$); ^1H NMR (300 MHz, $CDCl_3$) δ 8.00 (d, *J* = 7.4 Hz, 2H), 7.60 (t, *J* = 7.3 Hz, 1H), 7.49 (t, *J* = 7.6 Hz, 2H), 5.06 (dd, *J* = 7.7, 5.9 Hz, 1H), 2.27 – 1.91 (m, 2H), 1.08 (t, *J* = 7.3 Hz, 3H); ^{13}C NMR (75 MHz, DMSO) δ 193.5, 134.5, 133.6, 128.8, 128.7, 59.3, 27.0, 10.8; HRMS (ESI) m/z 205.0393 $(M+Na)^+$, calc. for $C_{10}H_{11}OClNa^+$ 205.0390.

The ee was determined by HPLC analysis: CHIRALPAK ID−CHIRALPAK ID (4.6 mm i.d. x 250 mm); Hexane/2-propanol = 98/2; flow rate 1.0 mL/min; 25 ºC; 254 nm; retention time: 11.3 min (minor) and 12.1 min (major).

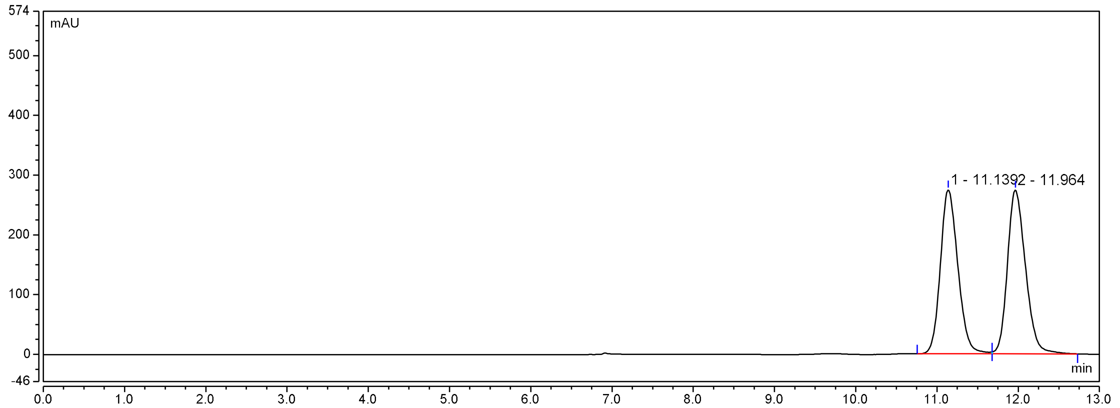

Entry	Retention Time	Area	Height	%Area
1	11.139	68.7268	274.42	49.31
2	11.964	70.6429	274.22	50.69

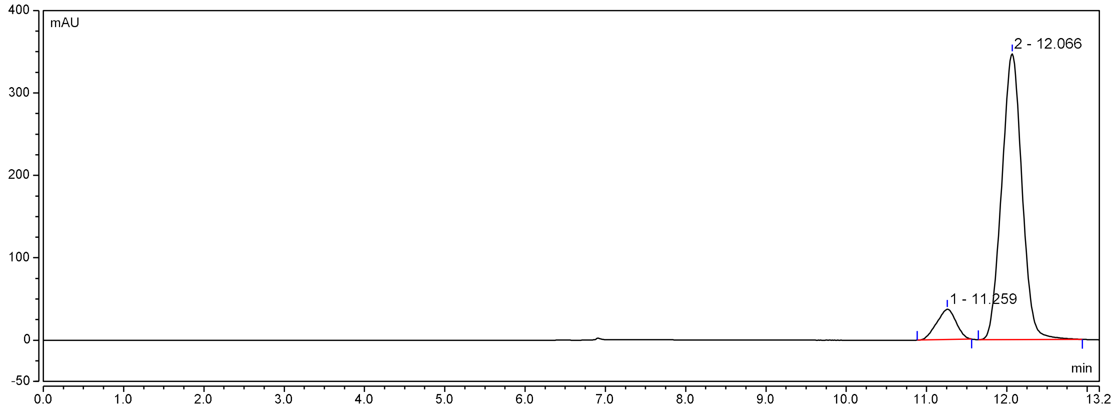

Entry	Retention Time	Area	Height	%Area
1	11.259	10.3188	36.76	9.24
2	12.066	101.4056	346.86	90.76

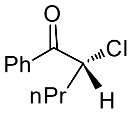

5o: colorless oil; 16.3 mg, 83% yield; 80% ee; $[\alpha]_D^{22}$ −67.9 (*c* 1.0, $CHCl_3$); ^1H NMR (300 MHz, $CDCl_3$) δ 8.05 – 7.95 (m, 2H), 7.59 (t, *J* = 7.4 Hz, 1H), 7.48 (t, *J* = 7.5 Hz, 2H), 5.13 (dd, *J* = 8.1, 5.8 Hz, 1H), 2.17 – 1.90 (m, 2H), 1.69 – 1.35 (m, 2H), 0.97 (t, *J* = 7.4 Hz, 3H); ^{13}C NMR (75 MHz, $CDCl_3$) δ 193.6, 134.5, 133.6, 128.8, 128.7, 57.5, 35.5, 19.5, 13.5; HRMS (ESI) m/z 219.0544 $(M+Na)^+$, calc. for $C_{11}H_{13}OClNa^+$ 219.0547.

The ee was determined by HPLC analysis: CHIRALPAK IE−CHIRALPAK IE (4.6 mm i.d. x 250 mm); Hexane/2-propanol = 98/2; flow rate 1.0 mL/min; 25 ºC; 254 nm; retention time: 11.9 min (major) and 13.7 min (minor).

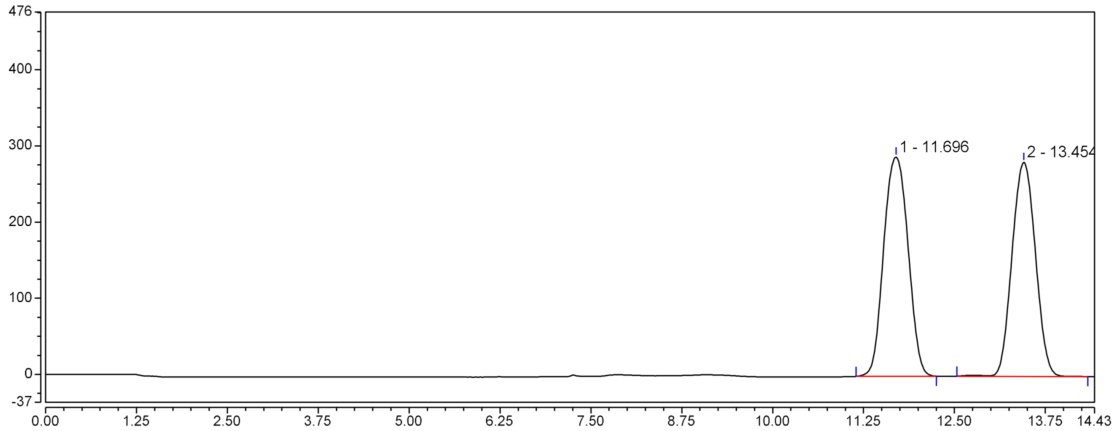

Entry	Retention Time	Area	Height	%Area
1	11.696	112.0732	287.85	51.66
2	13.454	104.8790	280.93	48.34

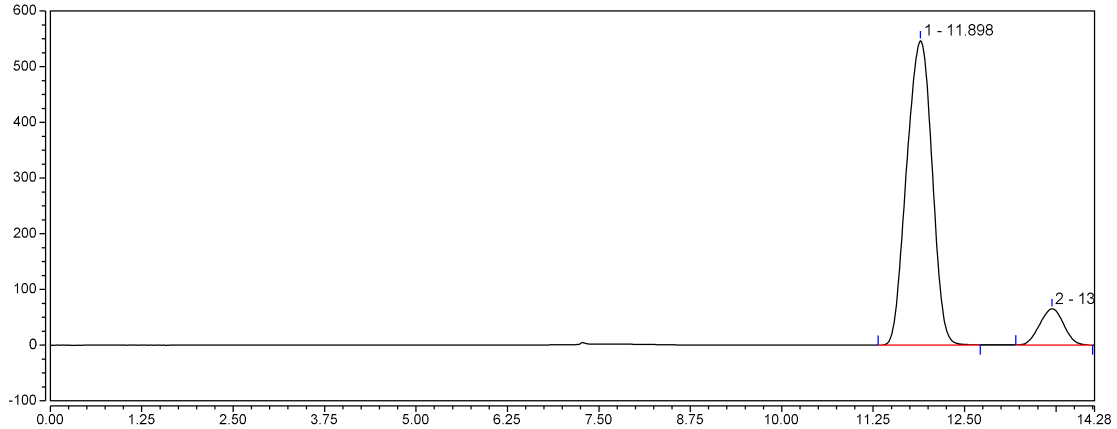

Entry	Retention Time	Area	Height	%Area
1	11.898	546.49	220.4470	89.89
2	13.696	65.04	24.8064	10.11

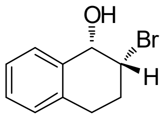

8: white solid; Mp 62.7−64.0 ºC; 13.8 mg, 61% yield; 90% ee; > 20:1 dr; $[\alpha]_D^{22}$ −5.4 (*c* 1.0, $CHCl_3$); ^1H NMR (300 MHz, $CDCl_3$) δ 7.50–7.47 (m, 1H), 7.26–7.23 (m, 2H), 7.13–7.10 (m, 1H), 4.80 (d, *J* = 3.0 Hz, 1H), 4.71 (dt, *J* = 8.3, 3.0 Hz, 1H), 3.11 (dt, *J* = 17.2, 6.5 Hz, 1H), 2.87 (dt, *J* = 17.2, 6.3 Hz, 1H), 2.52 (td, *J* = 14.3, 6.4 Hz, 1H), 2.30 (dtd, *J* = 9.4, 6.9, 2.7 Hz, 1H); ^{13}C NMR (75 MHz, $CDCl_3$) δ 136.0, 134.6, 128.6, 128.5, 128.2, 126.5, 70.2, 58.4, 28.3, 27.5; HRMS (ESI) m/z 248.9886 $(M+Na)^+$, calc. for $C_{10}H_{11}ONaBr^+$ 248.9891.

The ee was determined by HPLC analysis: CHIRALPAK IC (4.6 mm i.d. x 250 mm); Hexane/2-propanol = 95/5; flow rate 0.5 mL/min; 25 ºC; 210 nm; retention time: 17.3 min (minor) and 20.9 min (major).

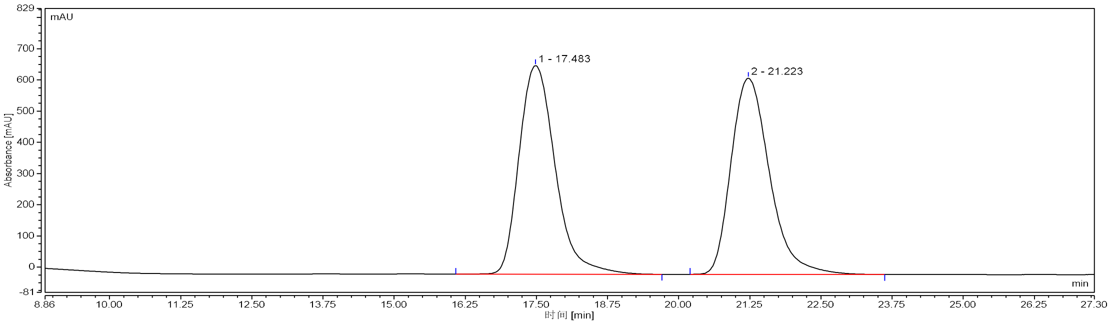

Entry	Retention Time	Area	Height	%Area
1	17.483	492.8670	668.75	50.02
2	21.223	492.5678	628.54	49.98

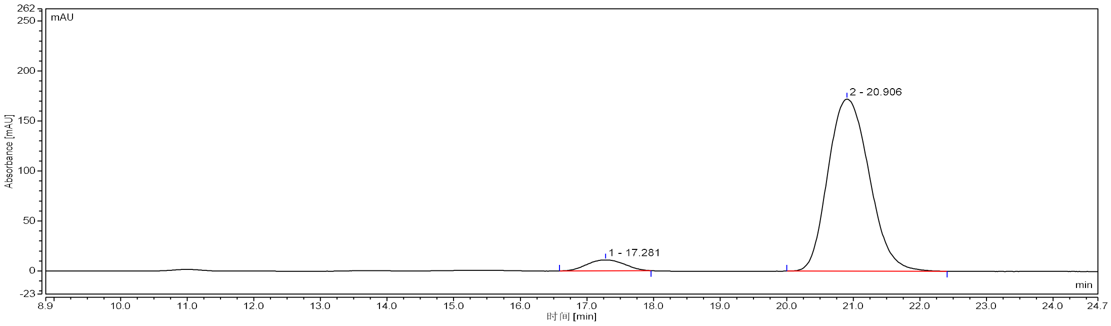

Entry	Retention Time	Area	Height	%Area
1	17.281	6.5102	10.44	5.00
2	20.906	123.7149	172.28	95.00

9. Crude ^{1}H NMR spectra to determine dr

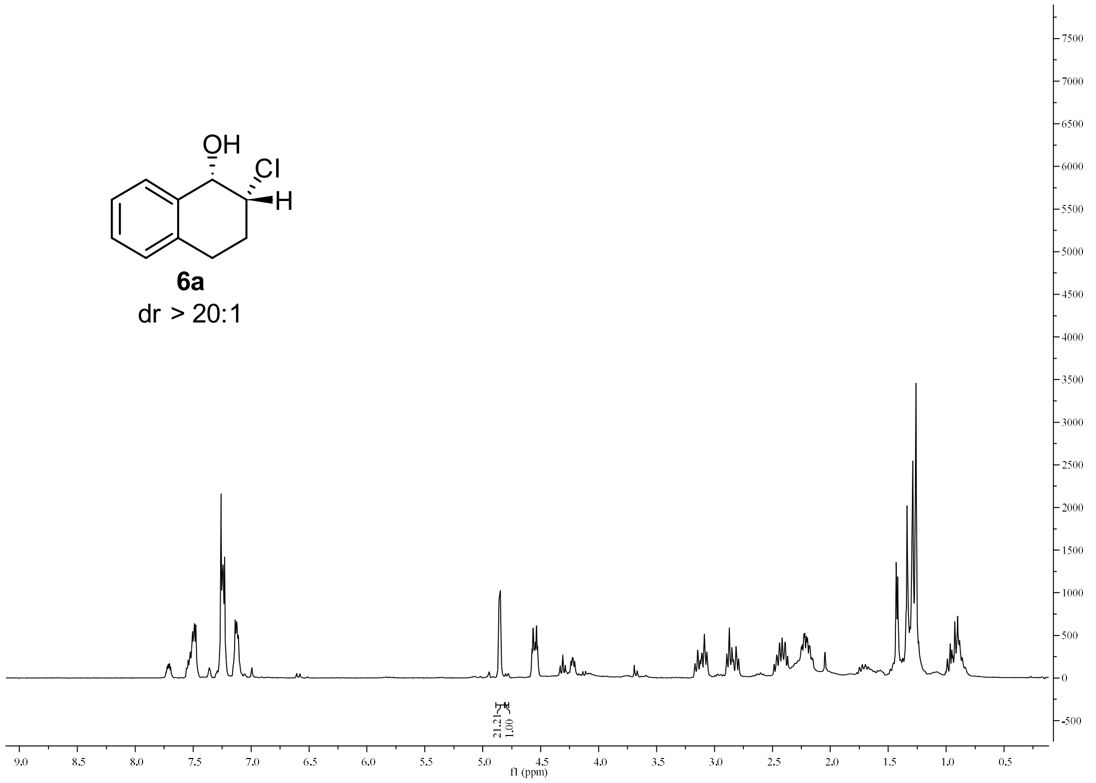

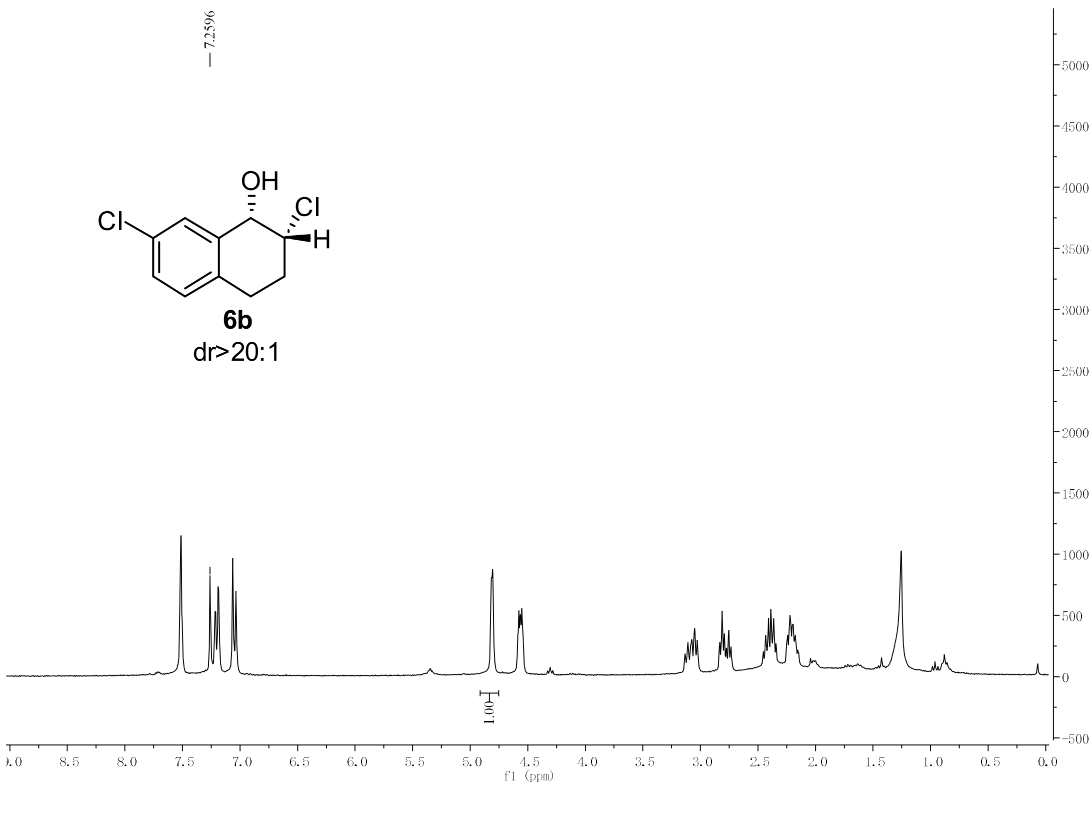

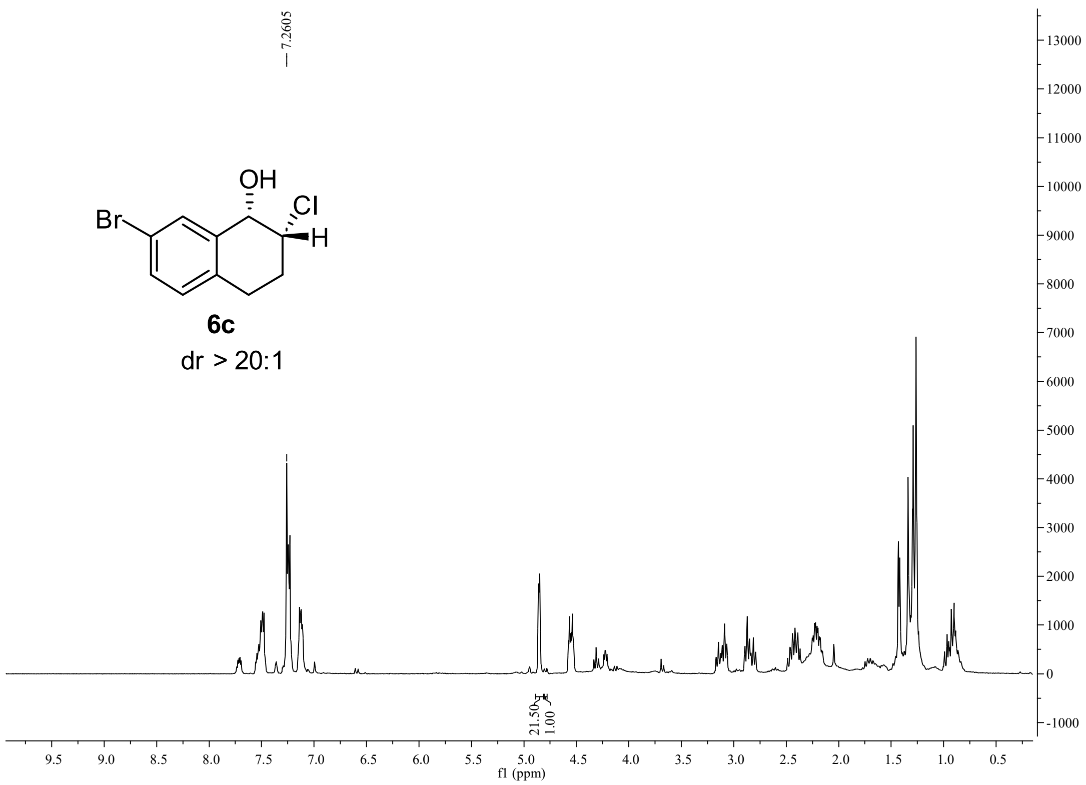

7.2605
OH
Cl
Br
H
6c
dr > 20:1
21.50
1.00
f1 (ppm)

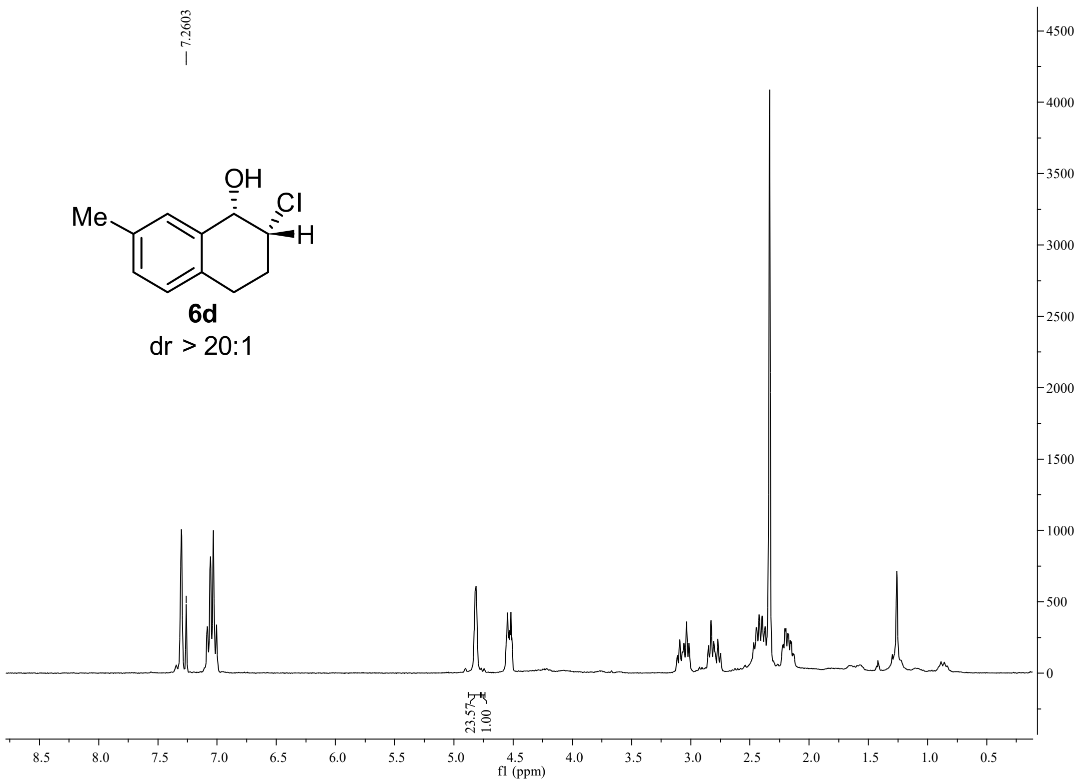

7.2603
OH
Cl
Me
H
6d
dr > 20:1
23.57
1.00
f1 (ppm)

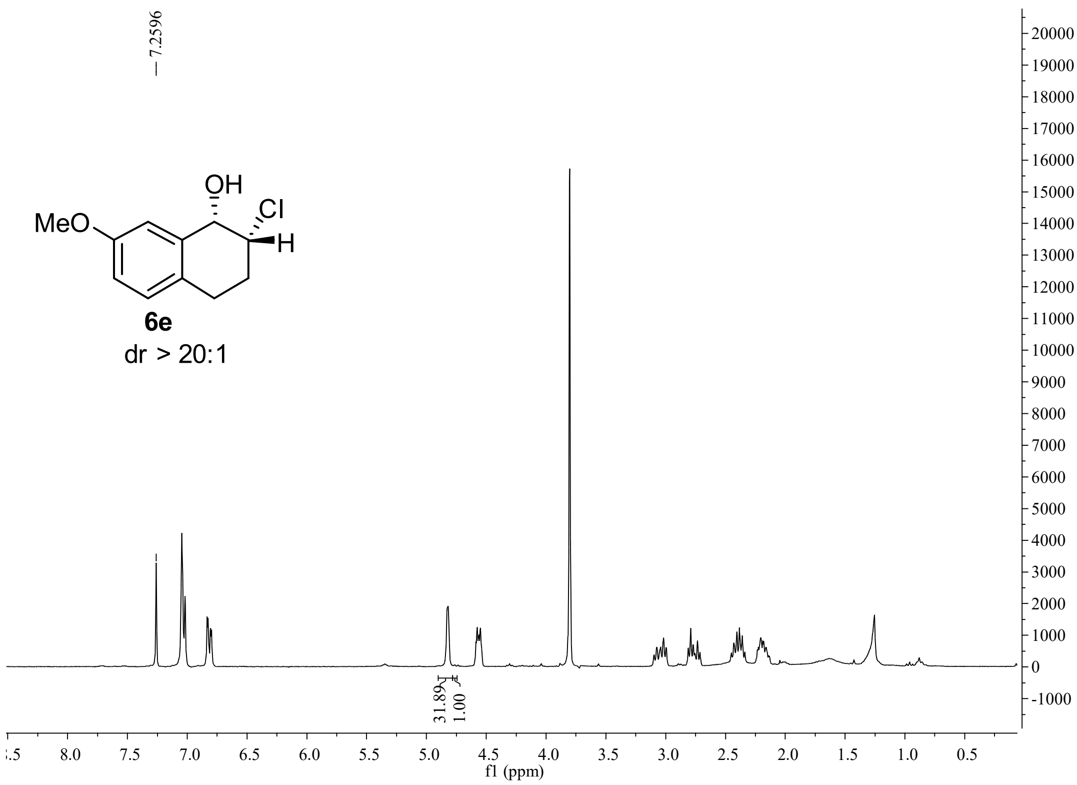

7.2596
MeO
OH
Cl
H
6e
dr > 20:1
31.89
1.00
f1 (ppm)

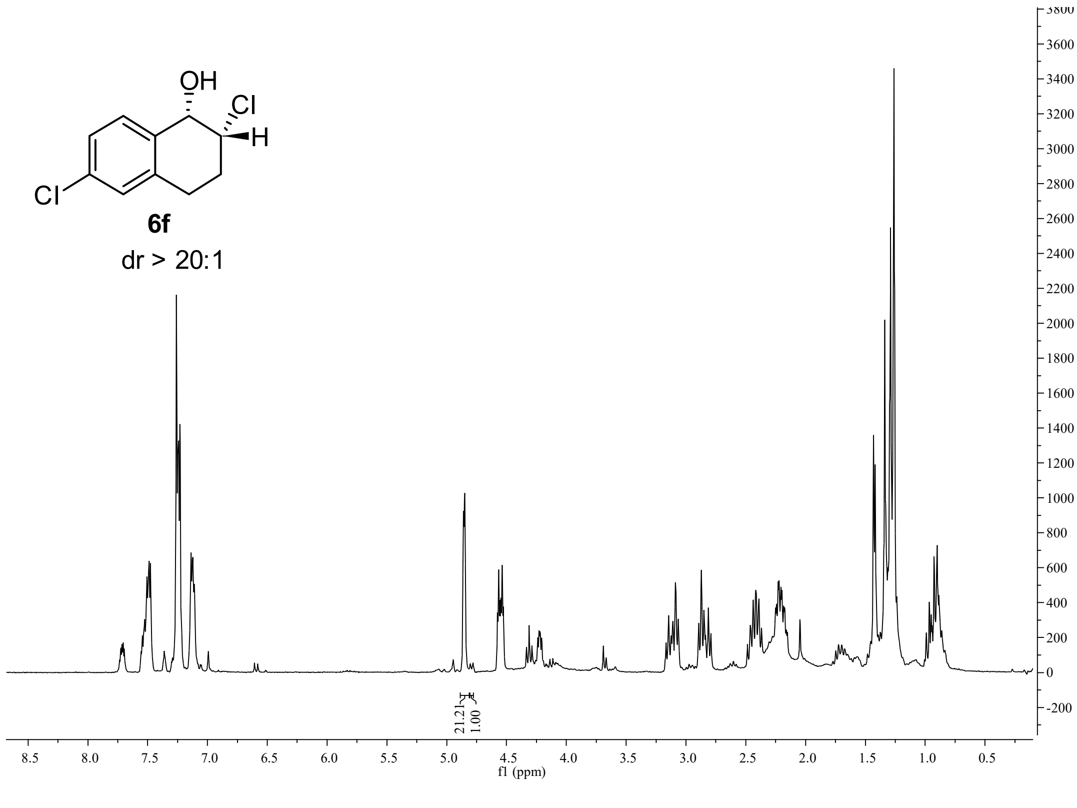

OH
Cl
H
Cl
6f
dr > 20:1
21.21
1.00
f1 (ppm)

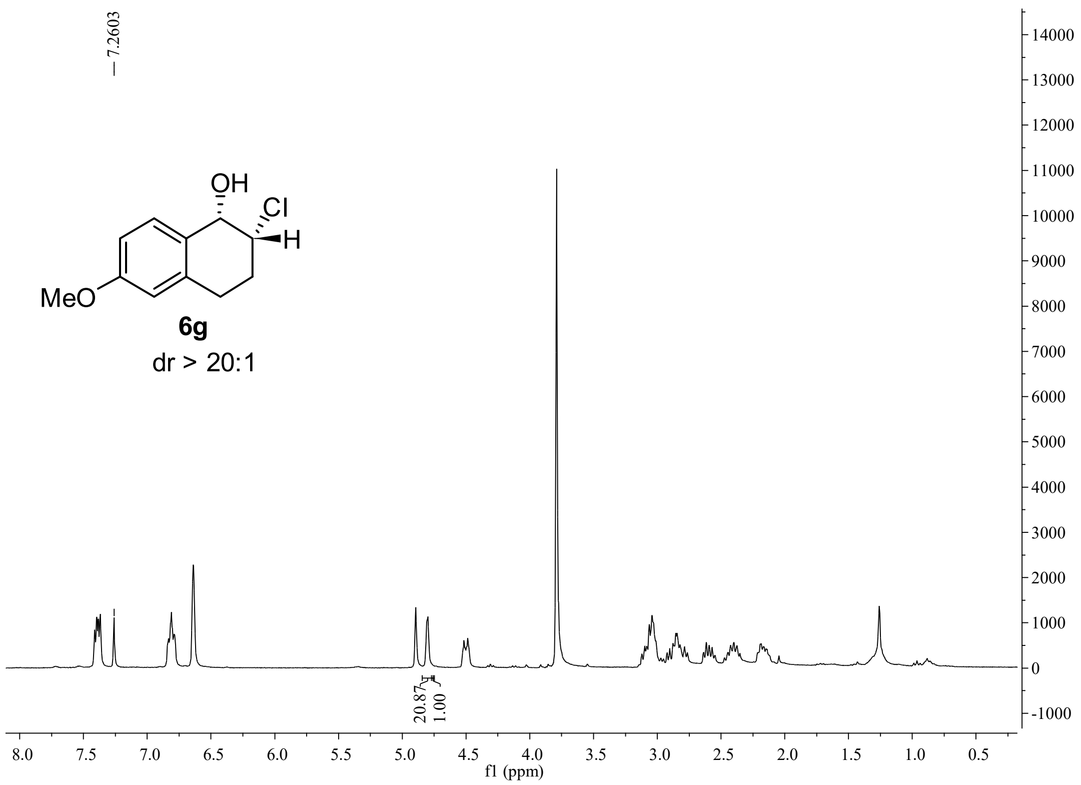
7.2603
OH
Cl
H
MeO
6g
dr > 20:1
20.87
1.00
f1 (ppm)

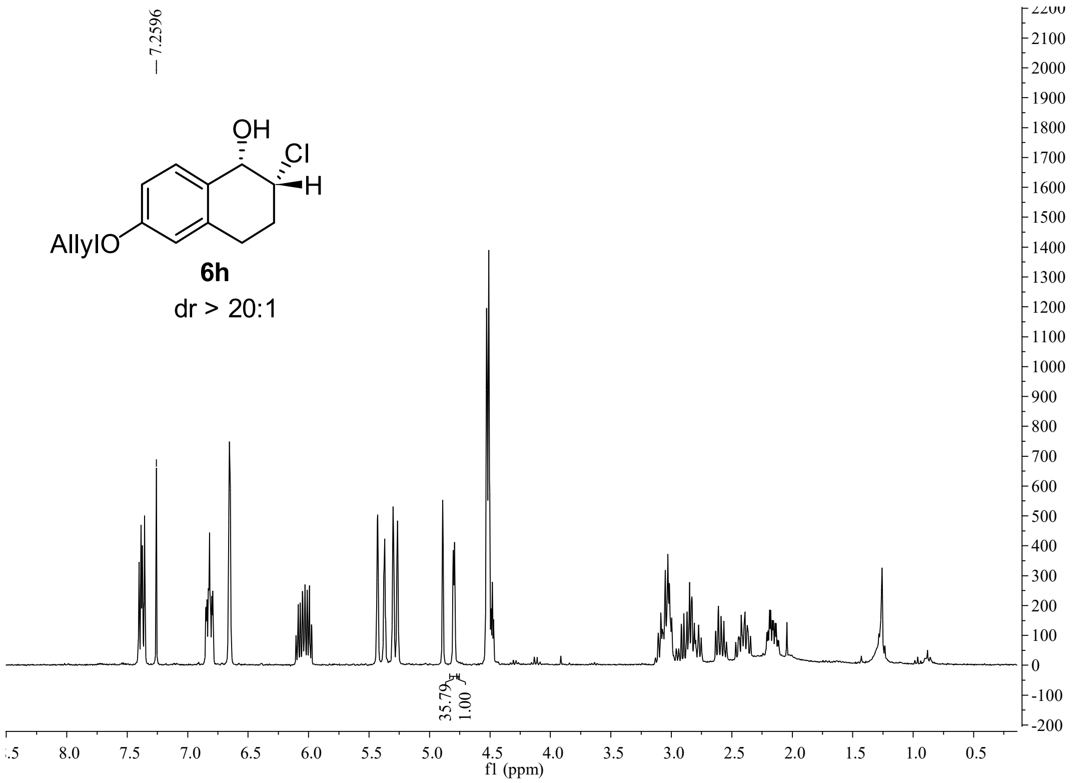
7.2596
OH
Cl
H
AllylO
6h
dr > 20:1
35.79
1.00
f1 (ppm)

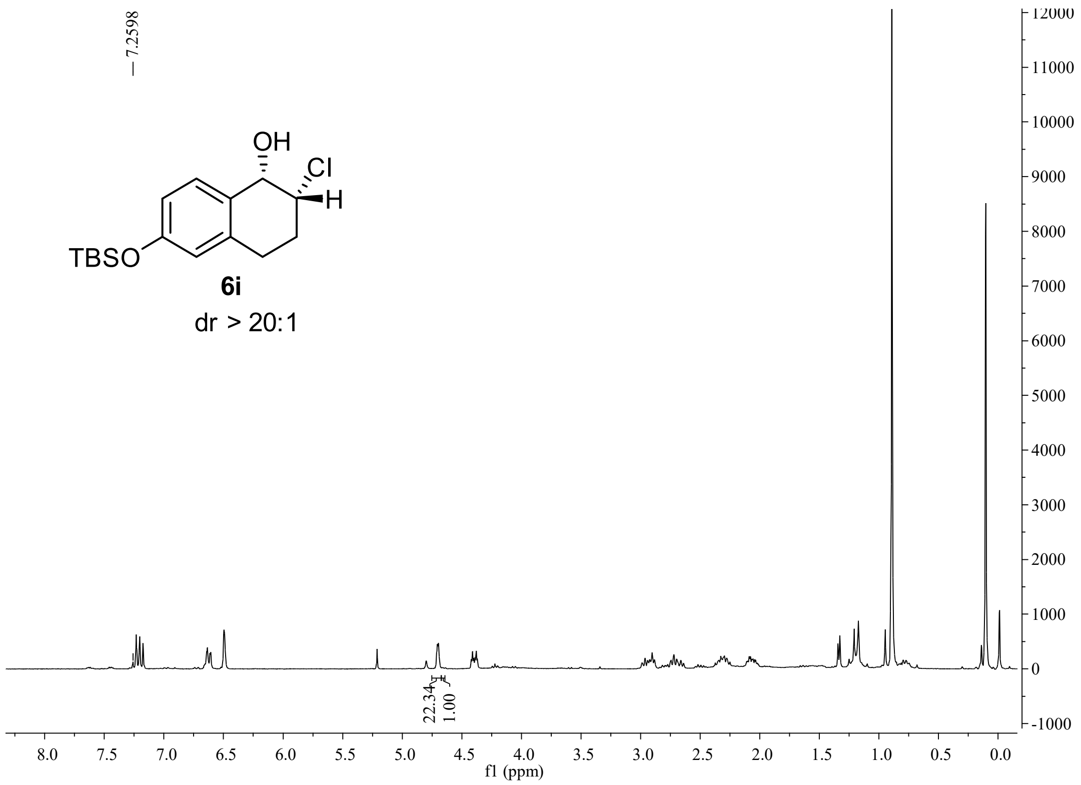
7.2598
OH
Cl
H
TBSO
6i
dr > 20:1
22.34
1.00
8.0 7.5 7.0 6.5 6.0 5.5 5.0 4.5 4.0 3.5 3.0 2.5 2.0 1.5 1.0 0.5 0.0
f1 (ppm)
12000 11000 10000 9000 8000 7000 6000 5000 4000 3000 2000 1000 0 -1000

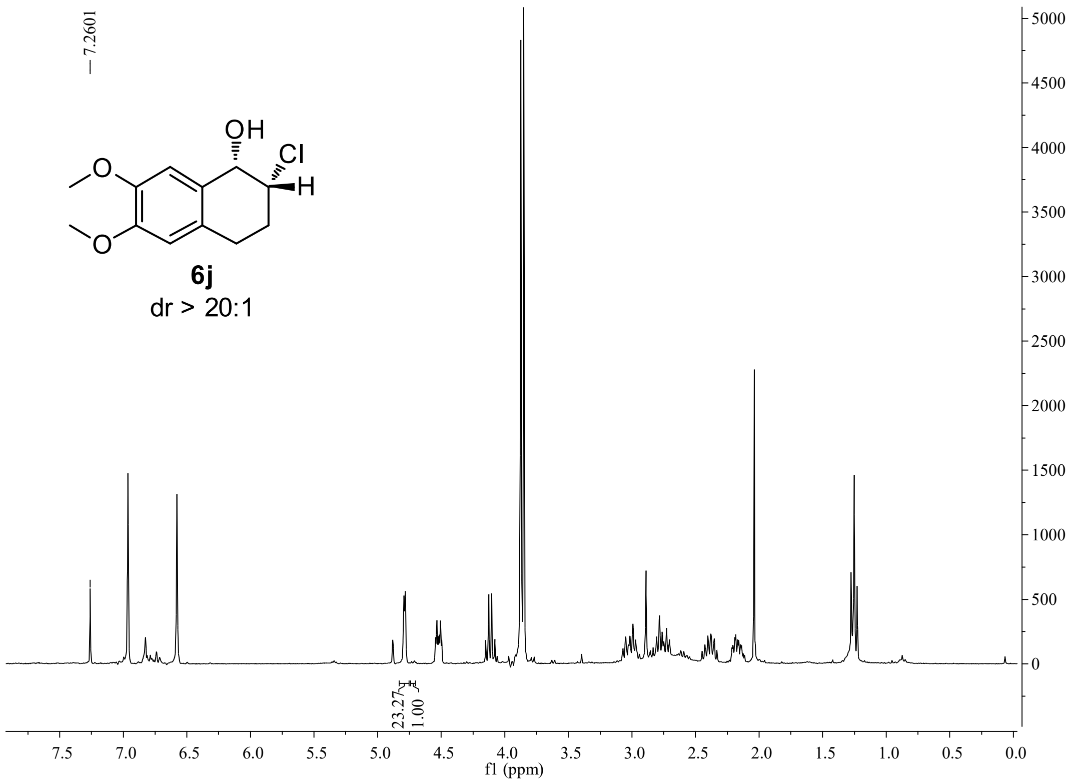
7.2601
OH
Cl
H
O
O
6j
dr > 20:1
23.27
1.00
7.5 7.0 6.5 6.0 5.5 5.0 4.5 4.0 3.5 3.0 2.5 2.0 1.5 1.0 0.5 0.0
f1 (ppm)
5000 4500 4000 3500 3000 2500 2000 1500 1000 500 0

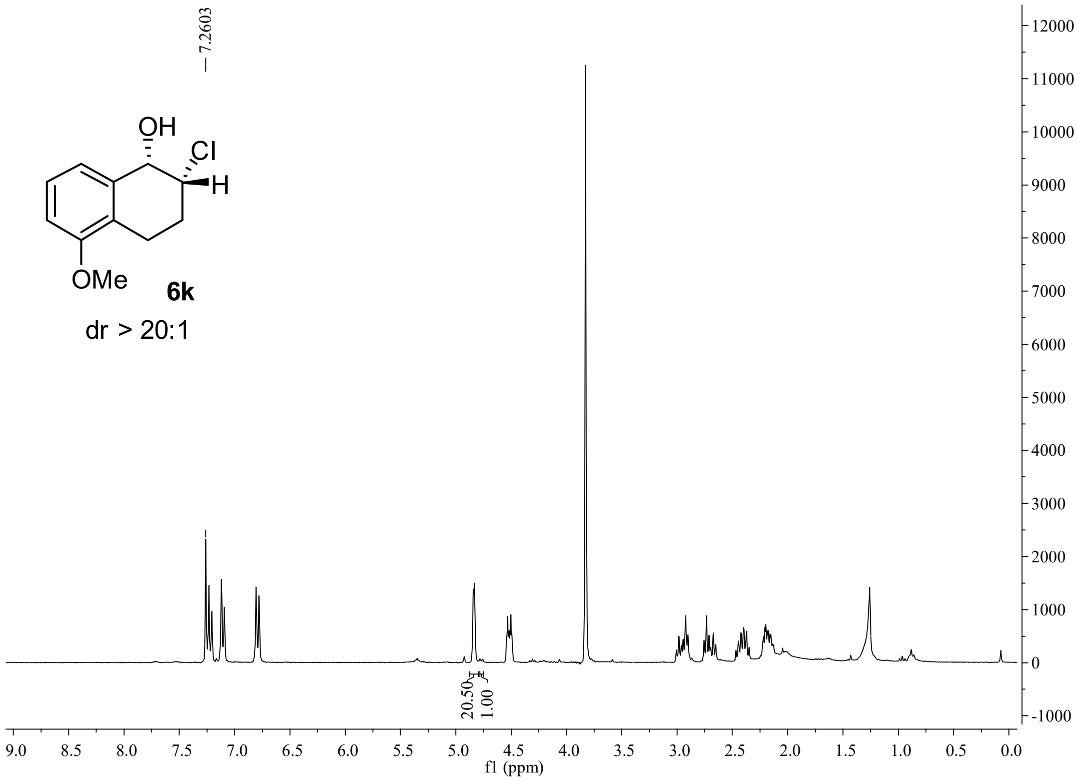

6k

dr > 20:1

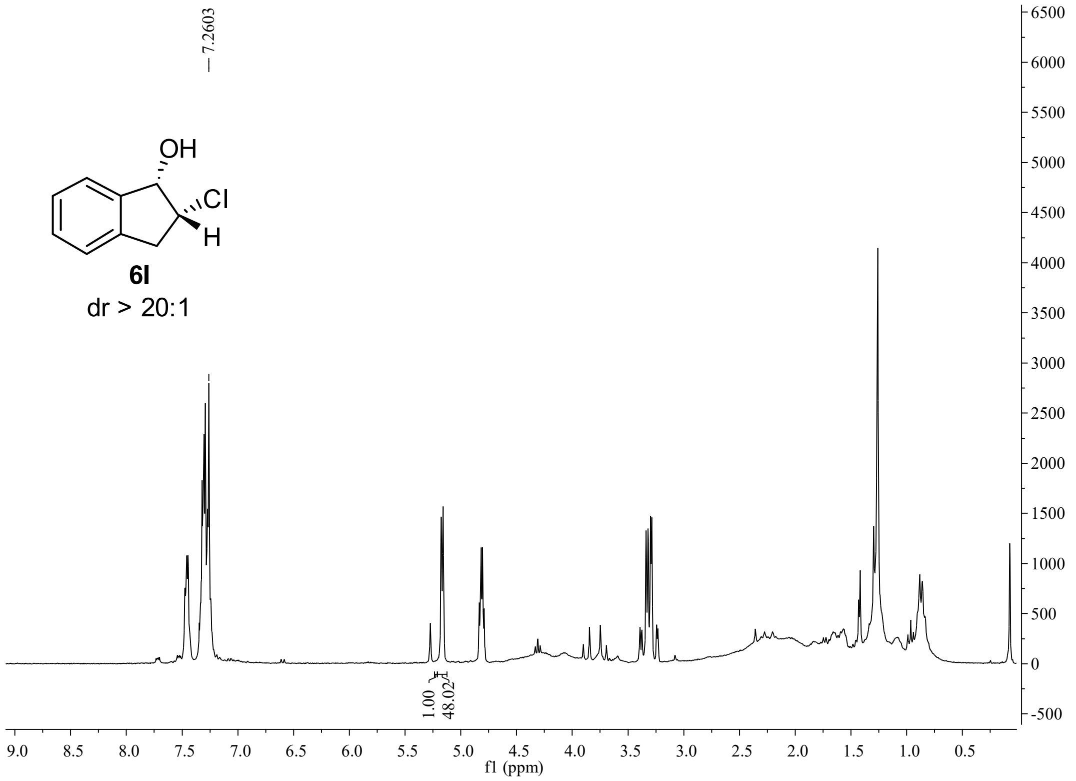

6l

dr > 20:1

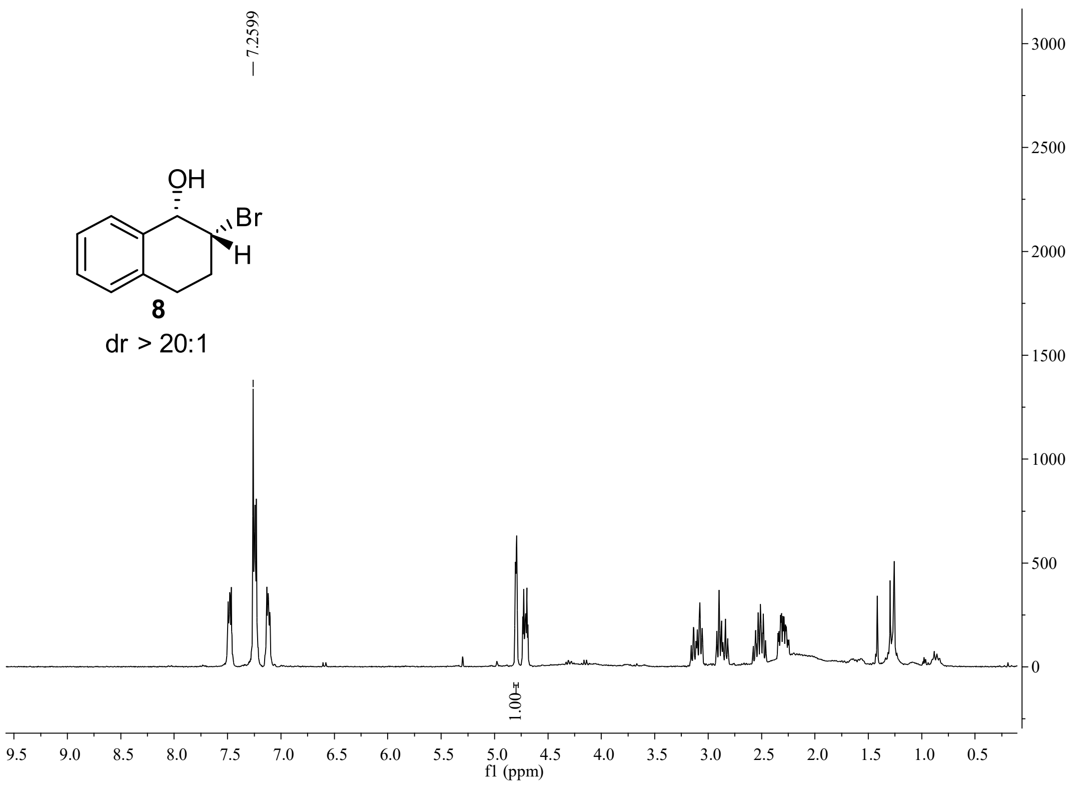

7.2599
OH
Br
H
8
dr > 20:1
1.00
9.5 9.0 8.5 8.0 7.5 7.0 6.5 6.0 5.5 5.0 4.5 4.0 3.5 3.0 2.5 2.0 1.5 1.0 0.5
f1 (ppm)
3000 2500 2000 1500 1000 500 0

10. Copies of NMR spectra

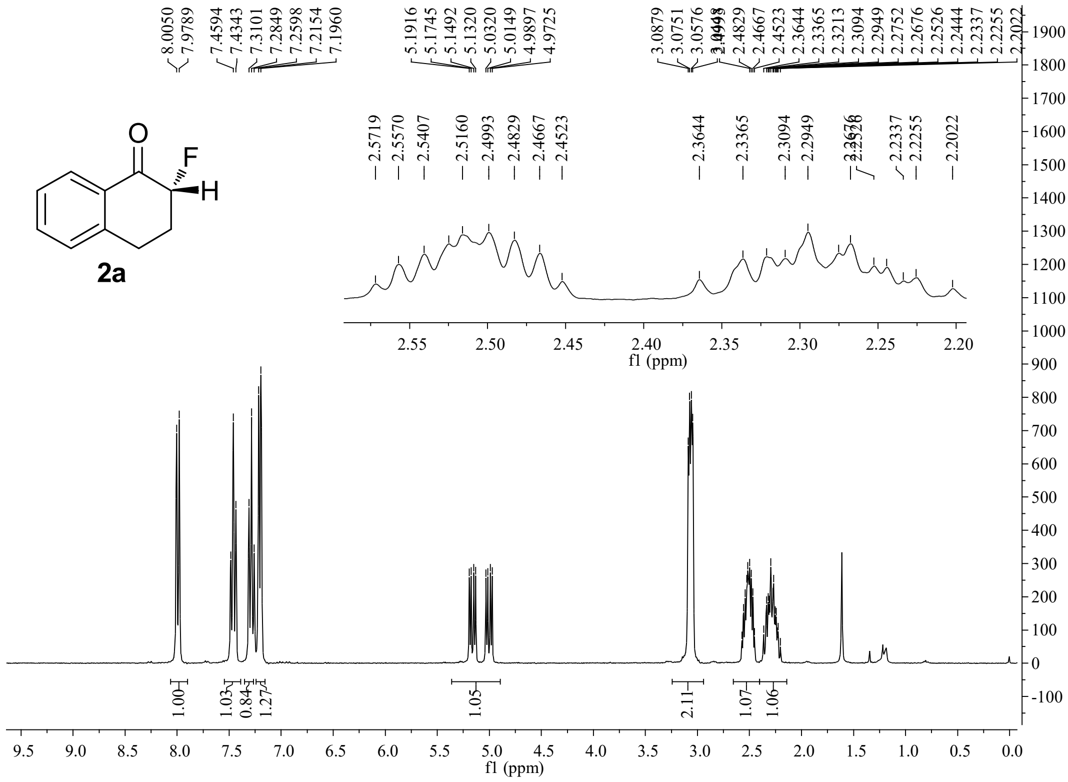

2a

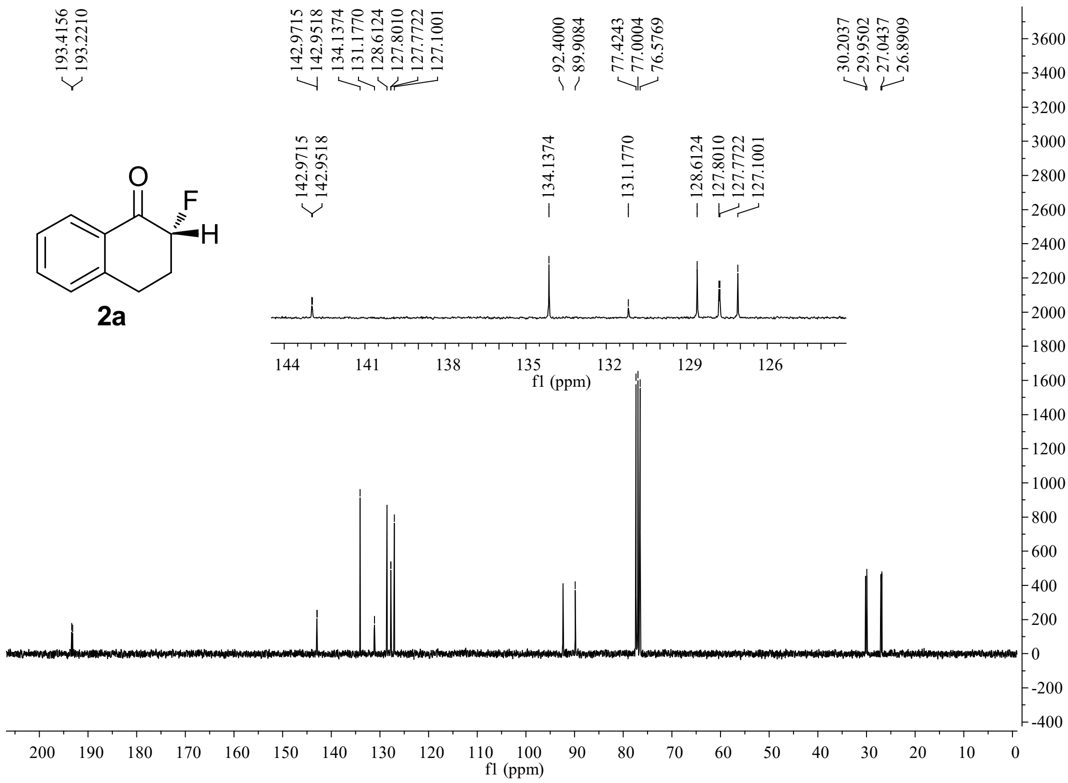

2a

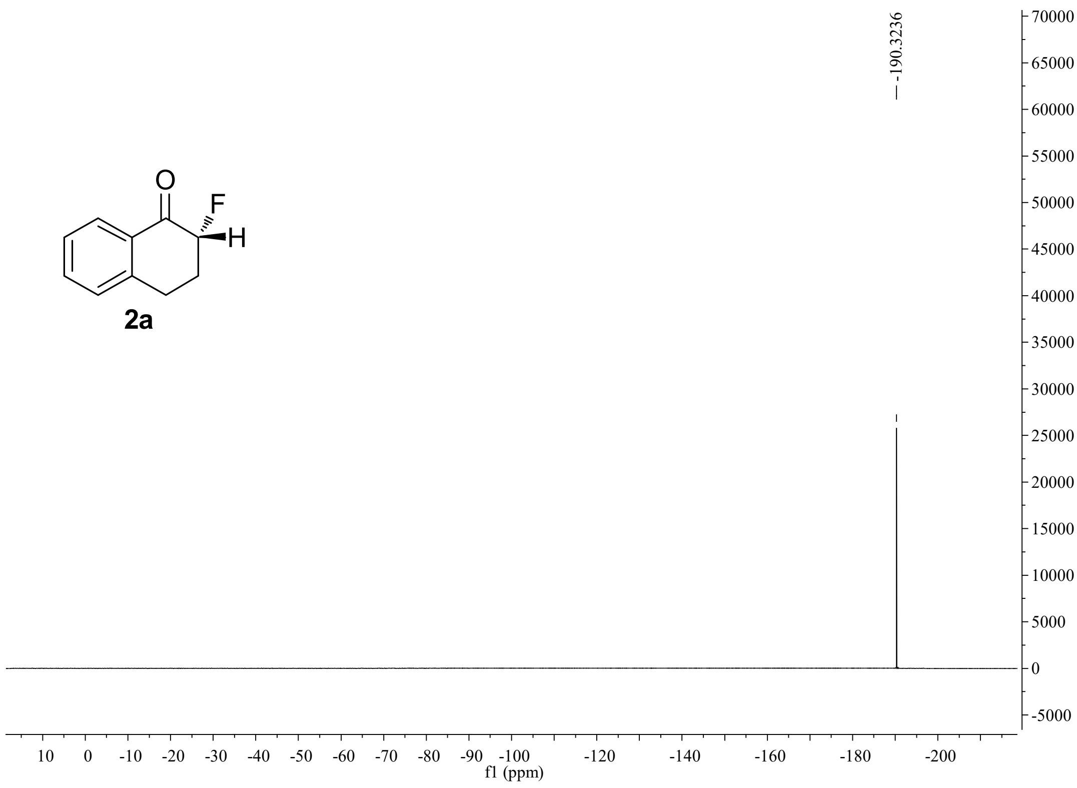

2a

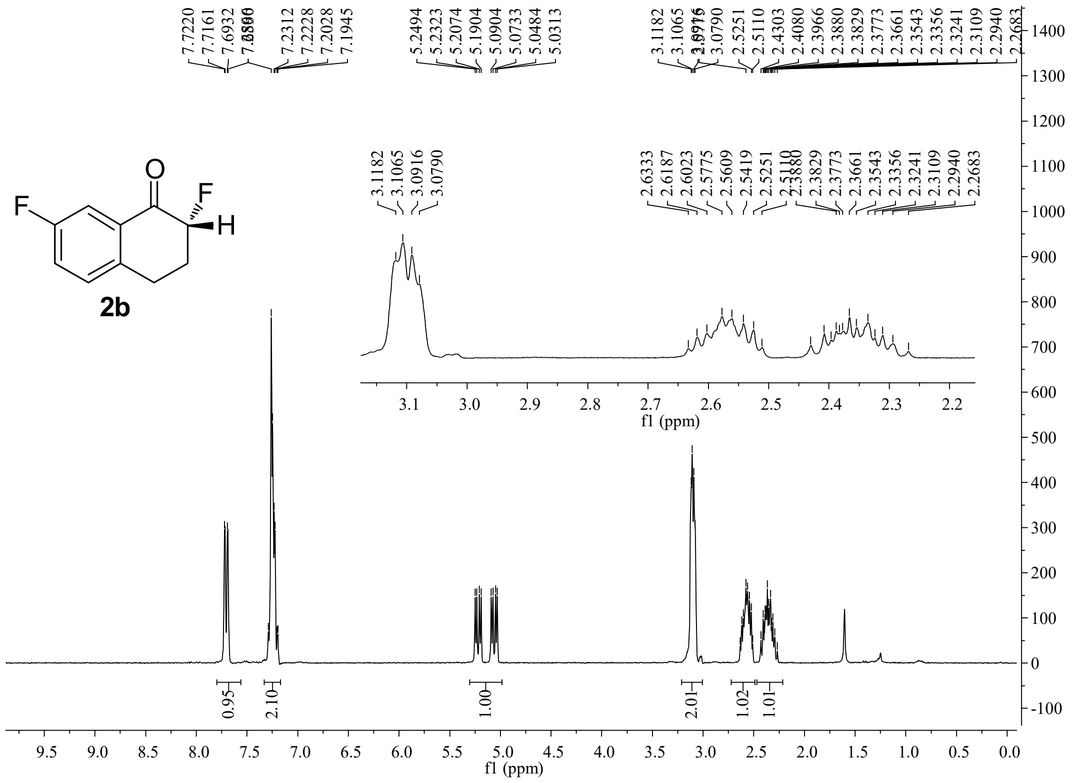

2b

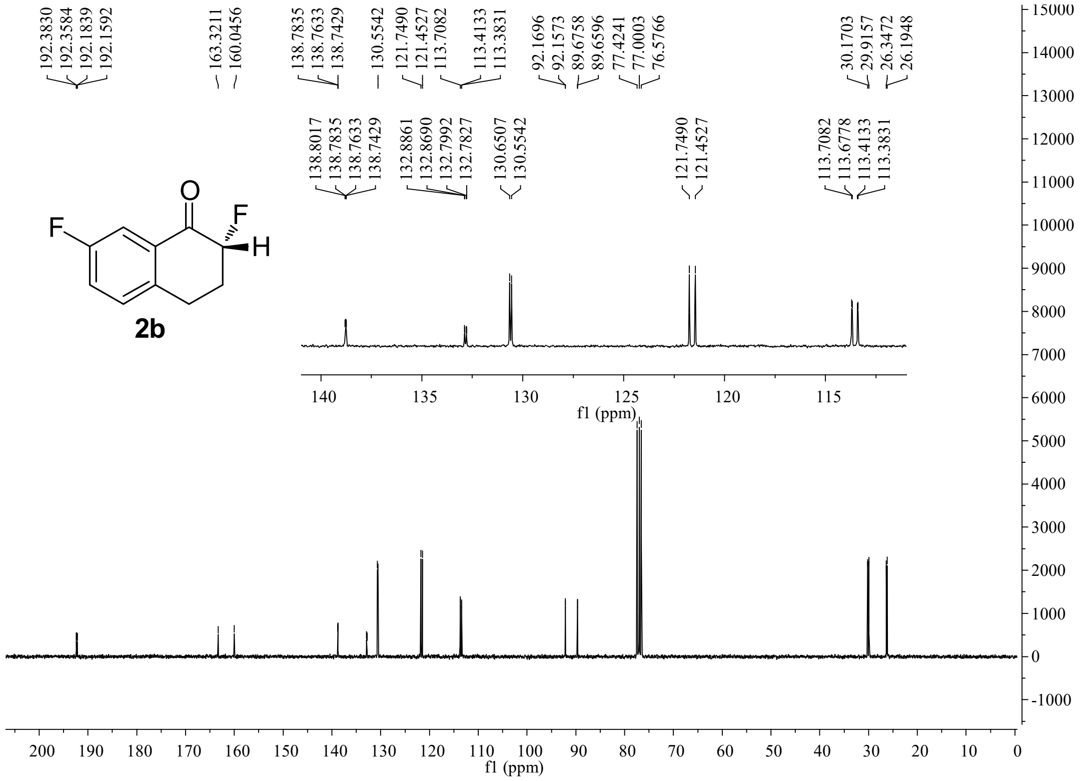
192.3830
192.3584
192.1839
192.1592
163.3211
160.0456
138.7835
138.7633
138.7429
130.5542
121.7490
121.4527
113.7082
113.4133
113.3831
92.1696
92.1573
89.6758
89.6596
77.4241
77.0003
76.5766
30.1703
29.9157
26.3472
26.1948
2b
f1 (ppm)

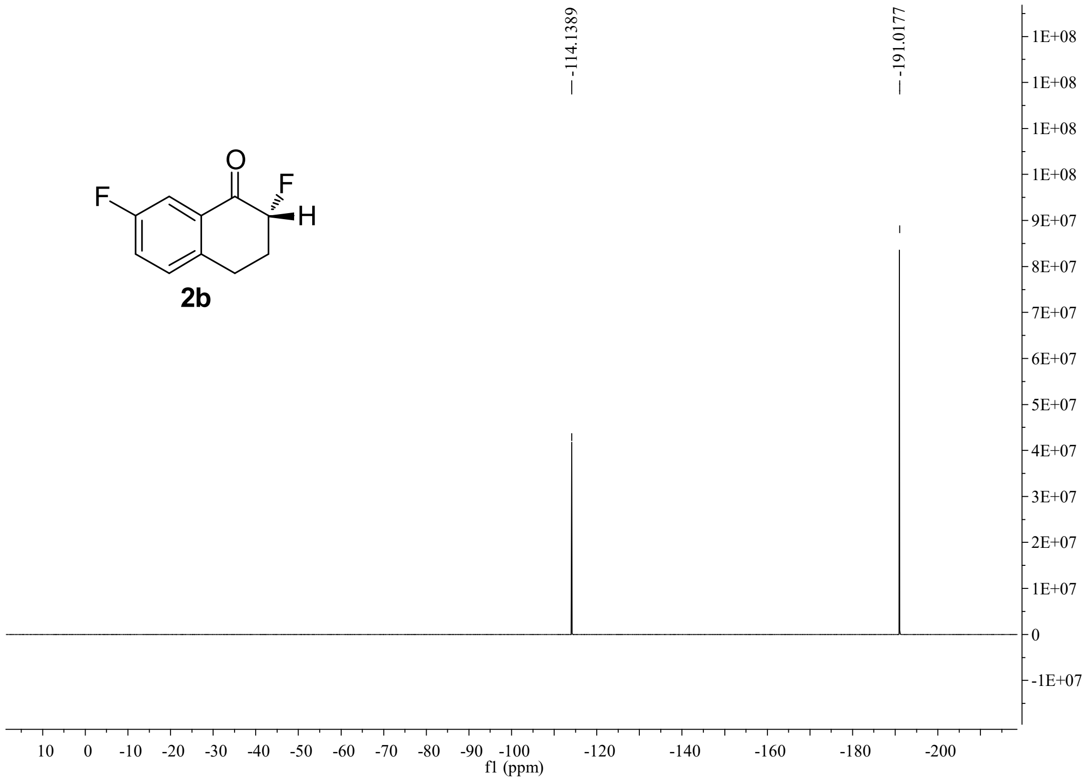
-114.1389
-191.0177
2b
f1 (ppm)

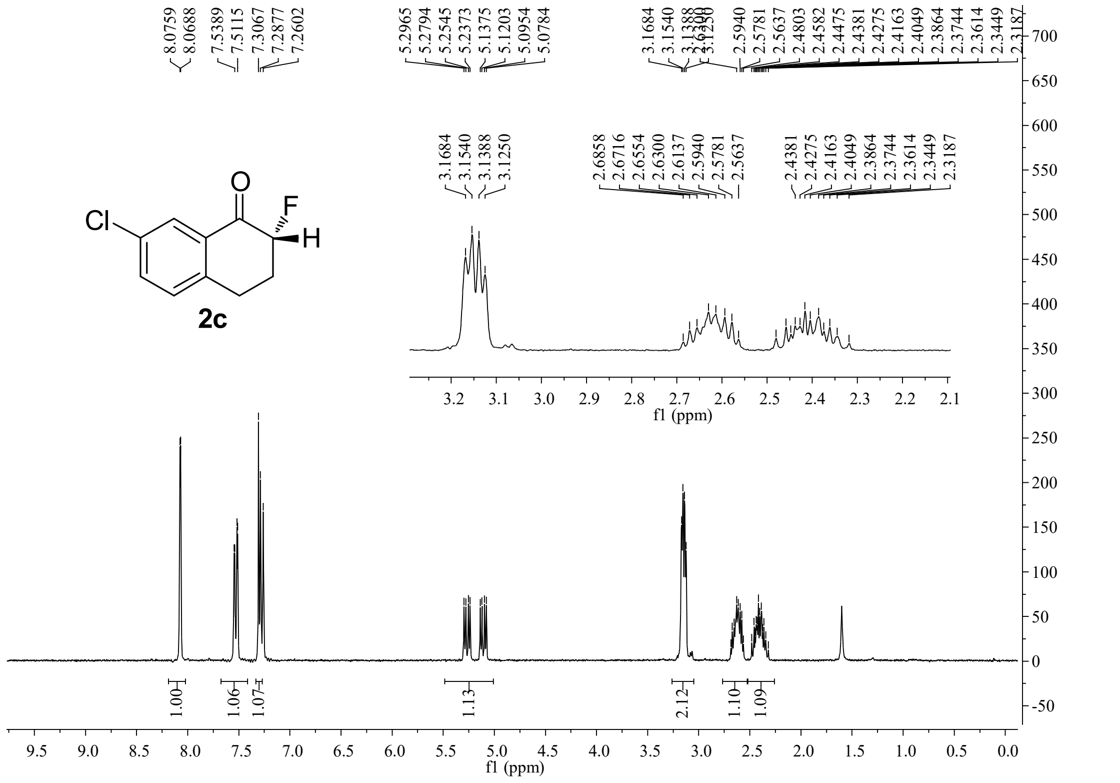

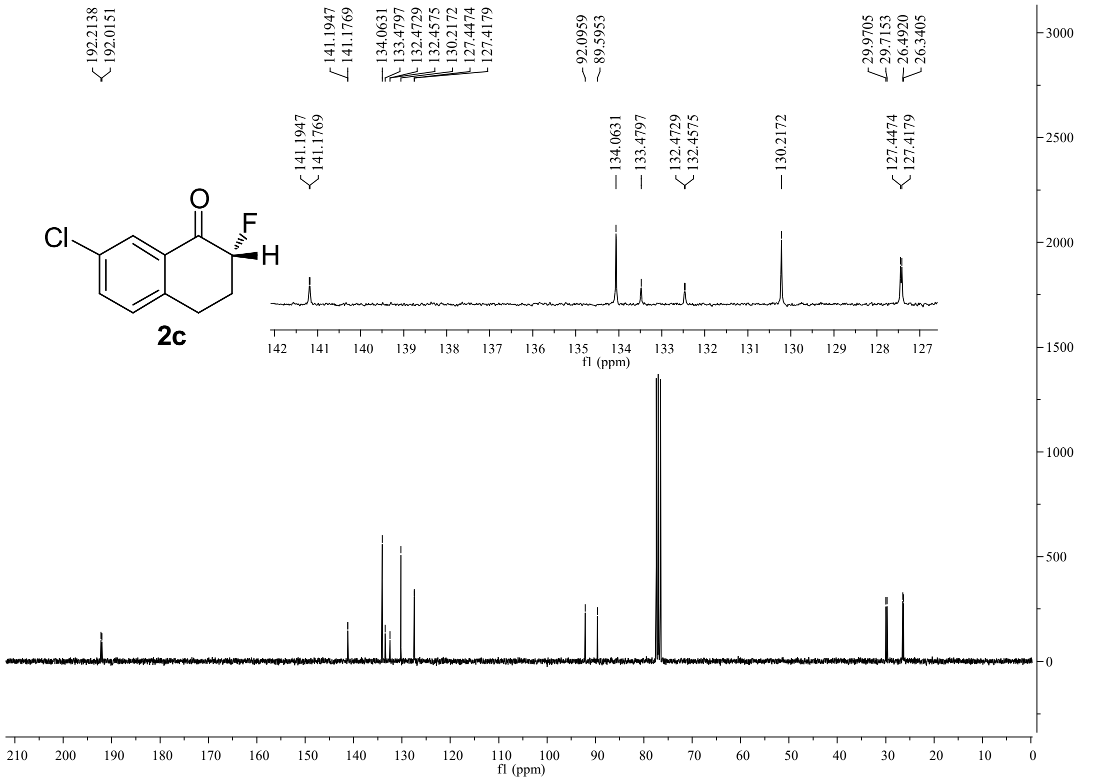

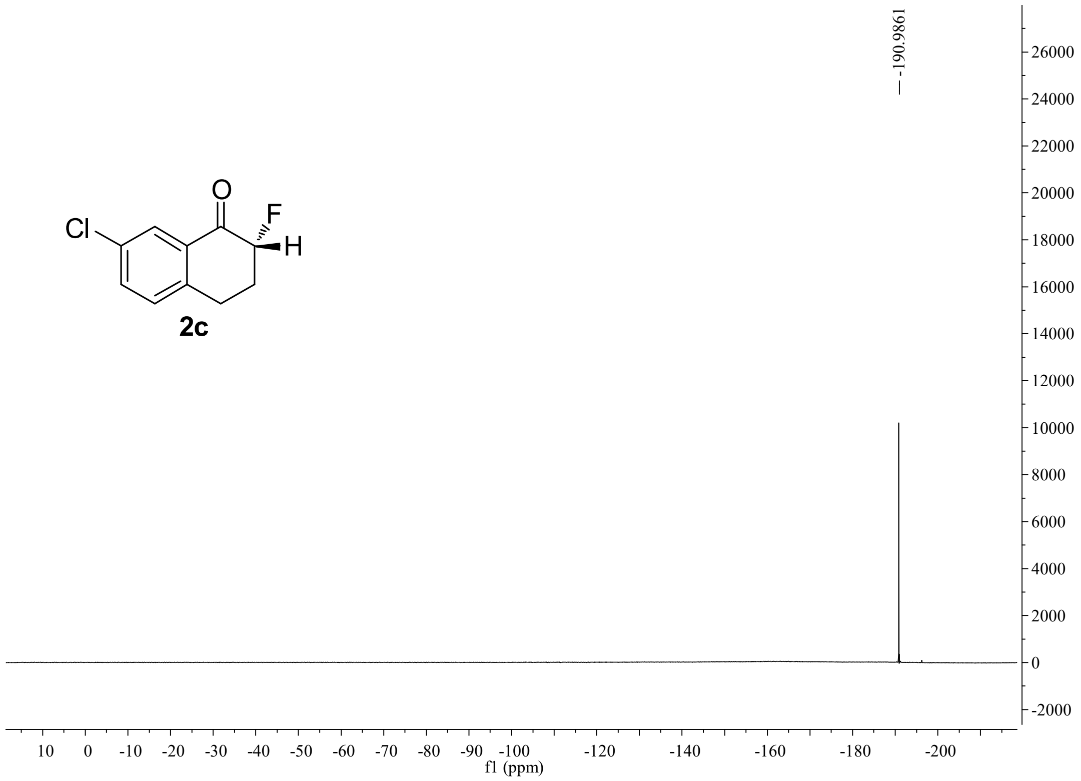
2c

-190.9861
f1 (ppm)

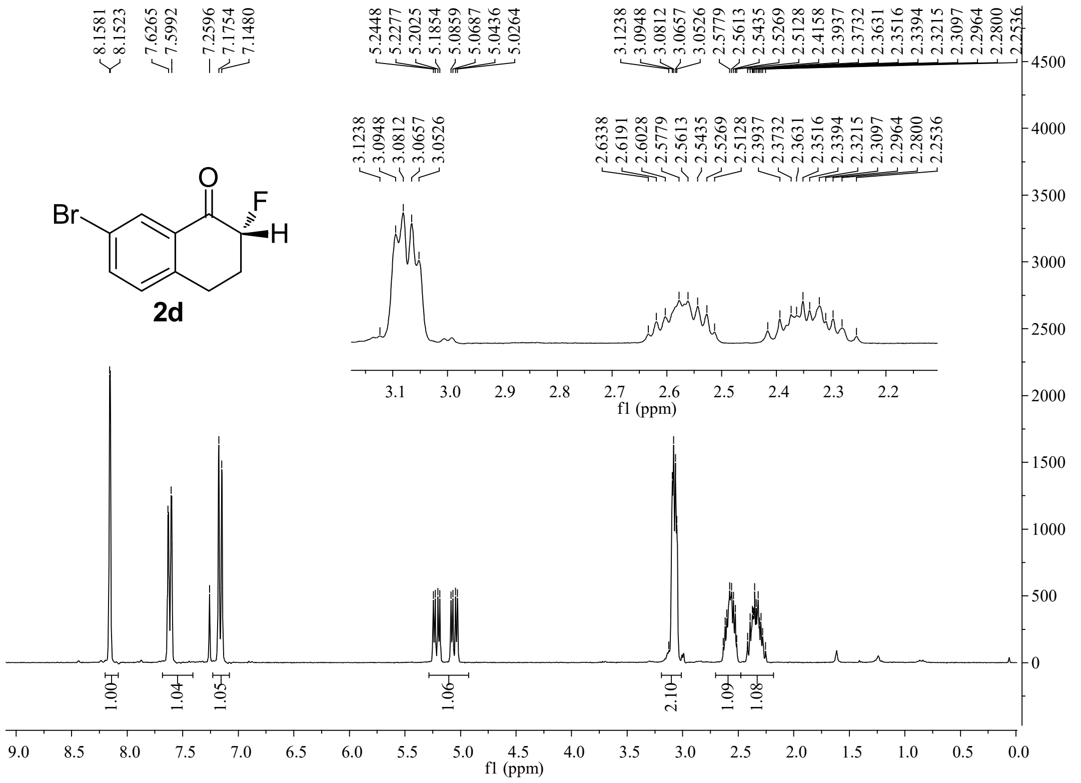
2d

8.1581
8.1523
7.6265
7.5992
7.2596
7.1754
7.1480
5.2448
5.2277
5.2025
5.1854
5.0859
5.0687
5.0436
5.0264
3.1238
3.0948
3.0812
3.0657
3.0526
2.6338
2.6191
2.6028
2.5779
2.5613
2.5435
2.5269
2.5128
2.4158
2.3937
2.3732
2.3631
2.3516
2.3394
2.3215
2.3097
2.2964
2.2800
2.2536
1.00
1.04
1.05
1.06
2.10
1.09
1.08
f1 (ppm)

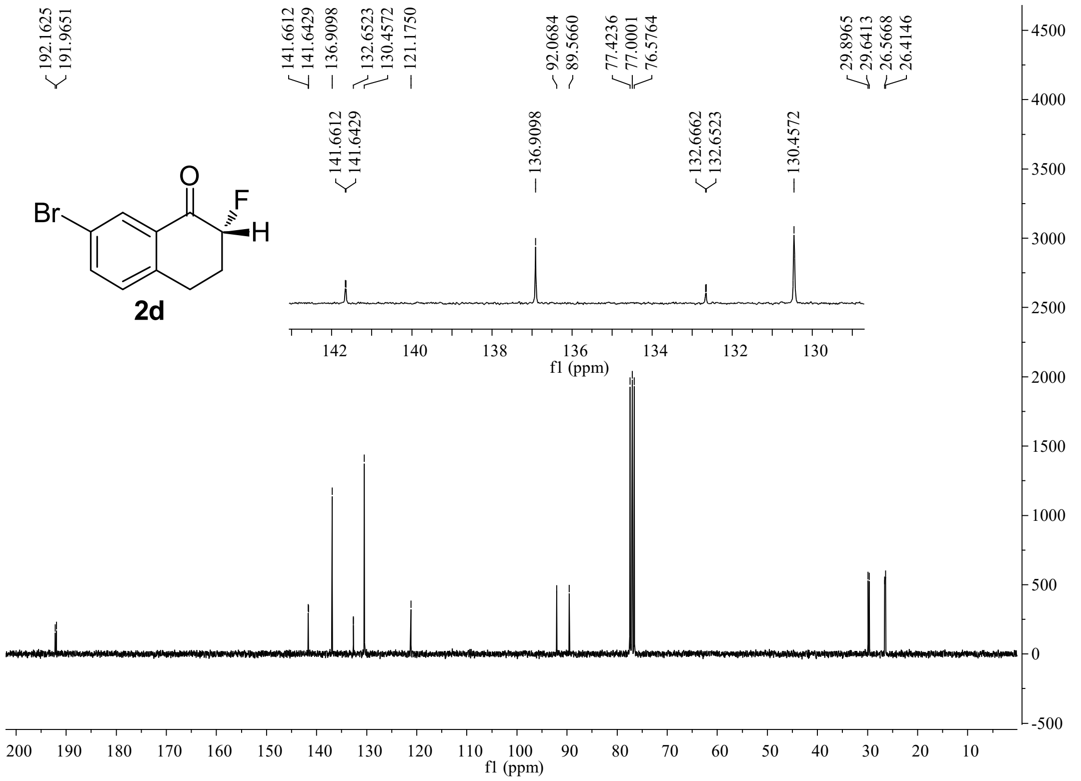
192.1625
191.9651
141.6612
141.6429
136.9098
132.6523
130.4572
121.1750
92.0684
89.5660
77.4236
77.0001
76.5764
29.8965
29.6413
26.5668
26.4146
141.6612
141.6429
136.9098
132.6662
132.6523
130.4572
Br
O
F
H
2d
fl (ppm)

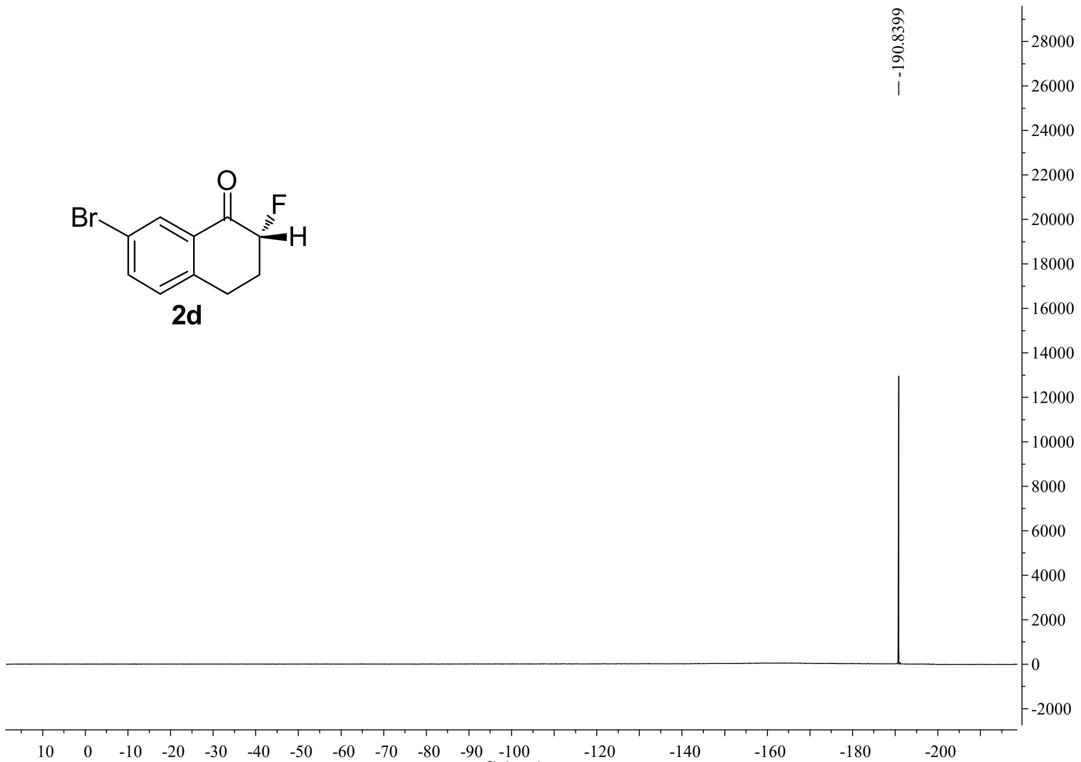
-190.8399
Br
O
F
H
2d
fl (ppm)

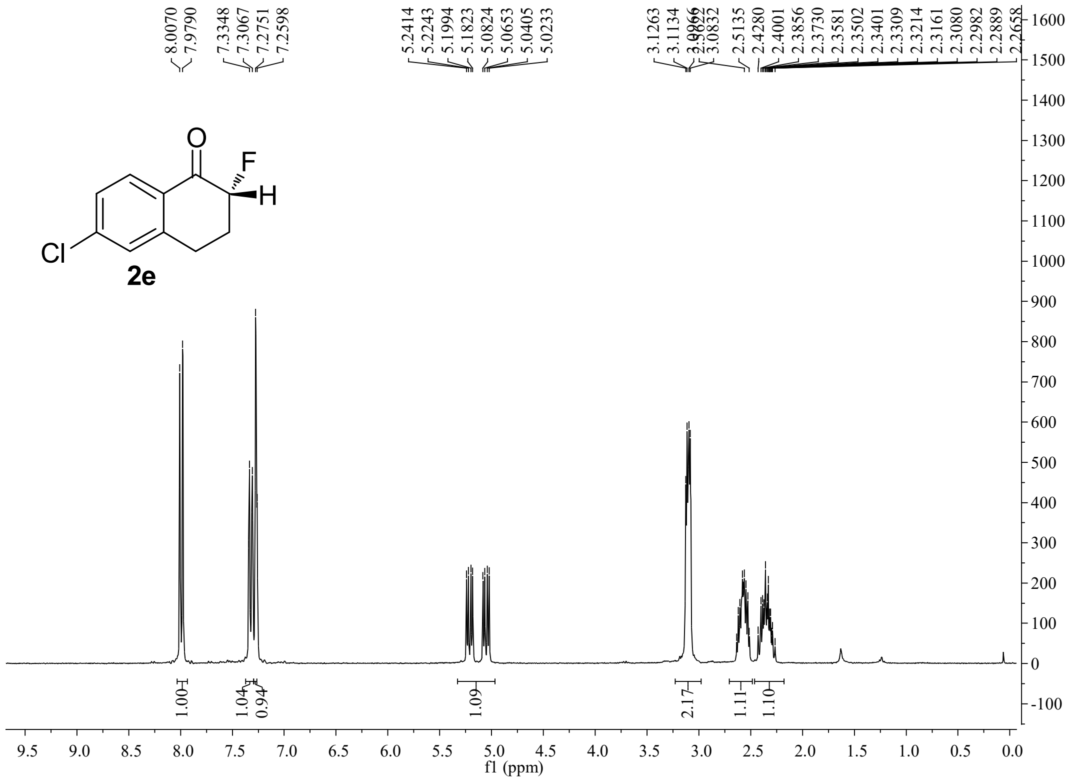
2e
O
F
H
Cl
8.0070
7.9790
7.3348
7.3067
7.2751
7.2598
5.2414
5.2243
5.1994
5.1823
5.0824
5.0653
5.0405
5.0233
3.1263
3.1134
2.5135
2.4280
2.4001
2.3856
2.3730
2.3581
2.3502
2.3401
2.3309
2.3214
2.3161
2.3080
2.2982
2.2889
1.00
1.04
0.94
1.09
2.17
1.11
1.10
f1 (ppm)

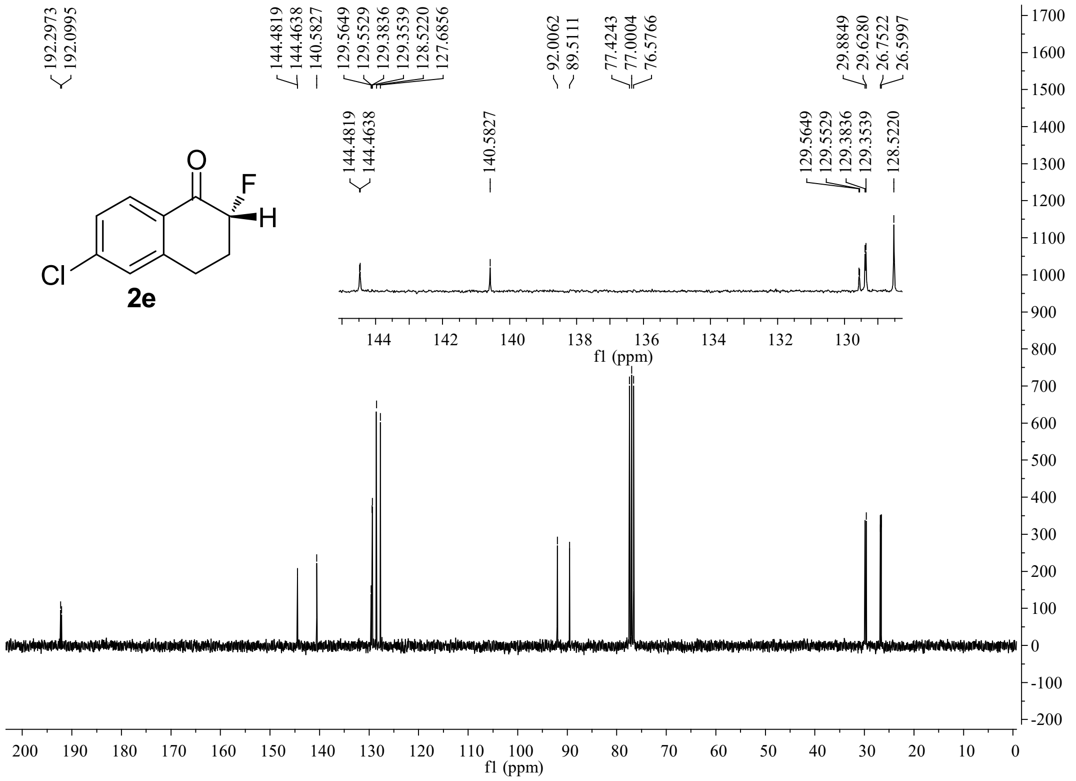
2e
O
F
H
Cl
192.2973
192.0995
144.4819
144.4638
140.5827
129.5649
129.5529
129.3836
129.3539
128.5220
127.6856
92.0062
89.5111
77.4243
77.0004
76.5766
29.8849
29.6280
26.7522
26.5997
f1 (ppm)

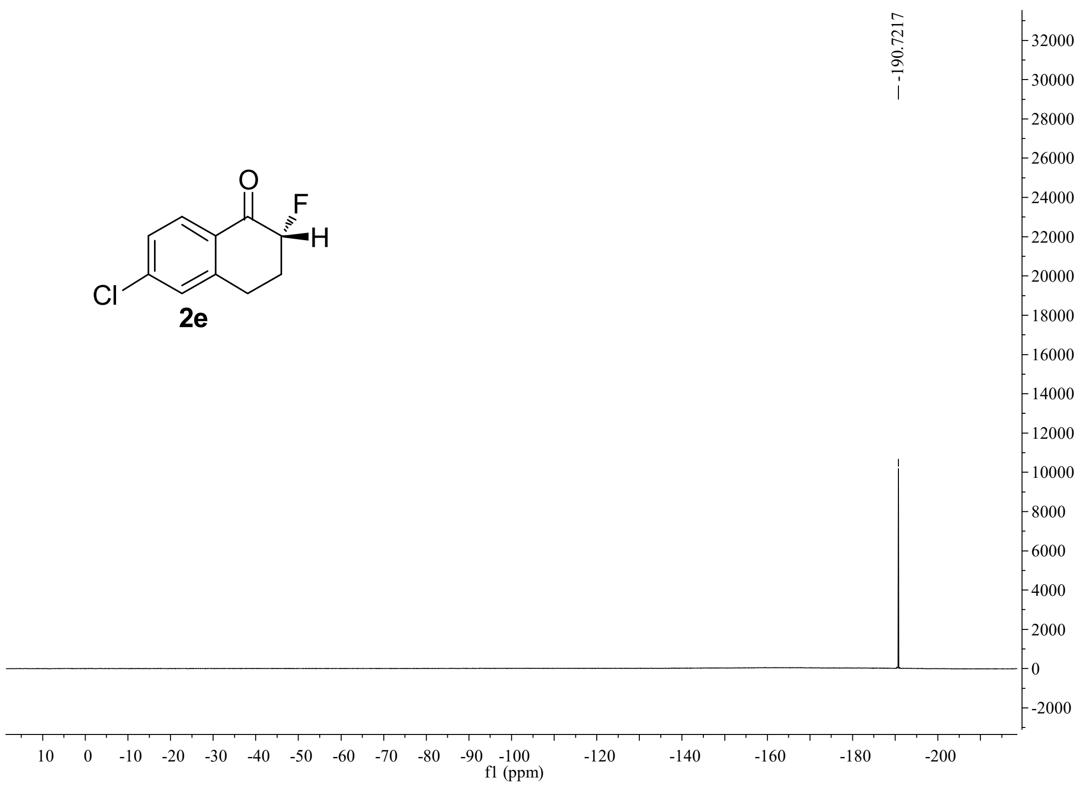
-190.7217
2e
f1 (ppm)

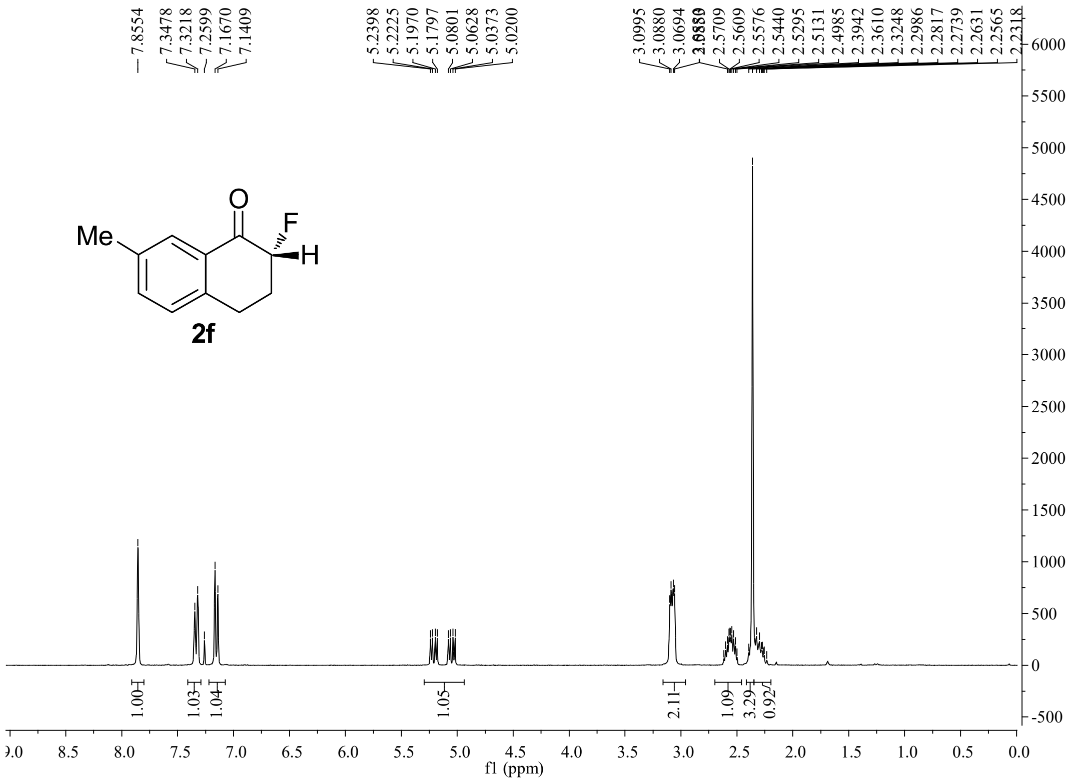
2f
f1 (ppm)

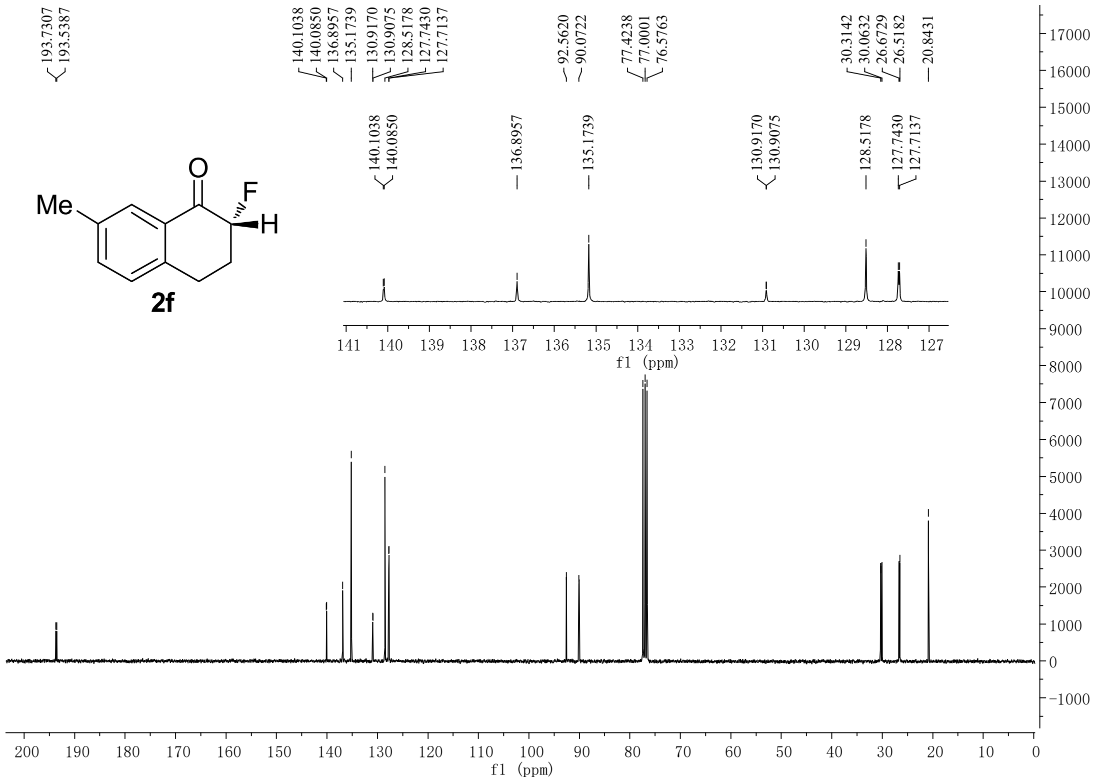
193.7307
193.5387
140.1038
140.0850
136.8957
135.1739
130.9170
130.9075
128.5178
127.7430
127.7137
92.5620
90.0722
77.4238
77.0001
76.5763
30.3142
30.0632
26.6729
26.5182
20.8431
Me
O
F
H
2f
f1 (ppm)

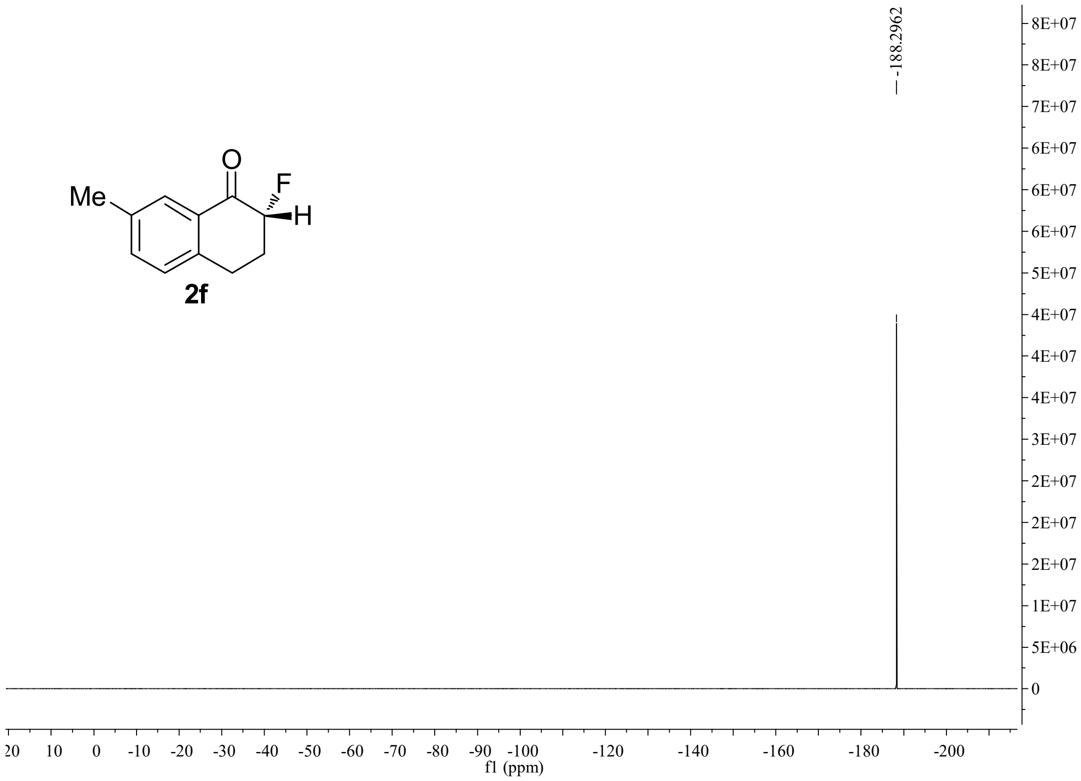
-188.2962
Me
O
F
H
2f
f1 (ppm)

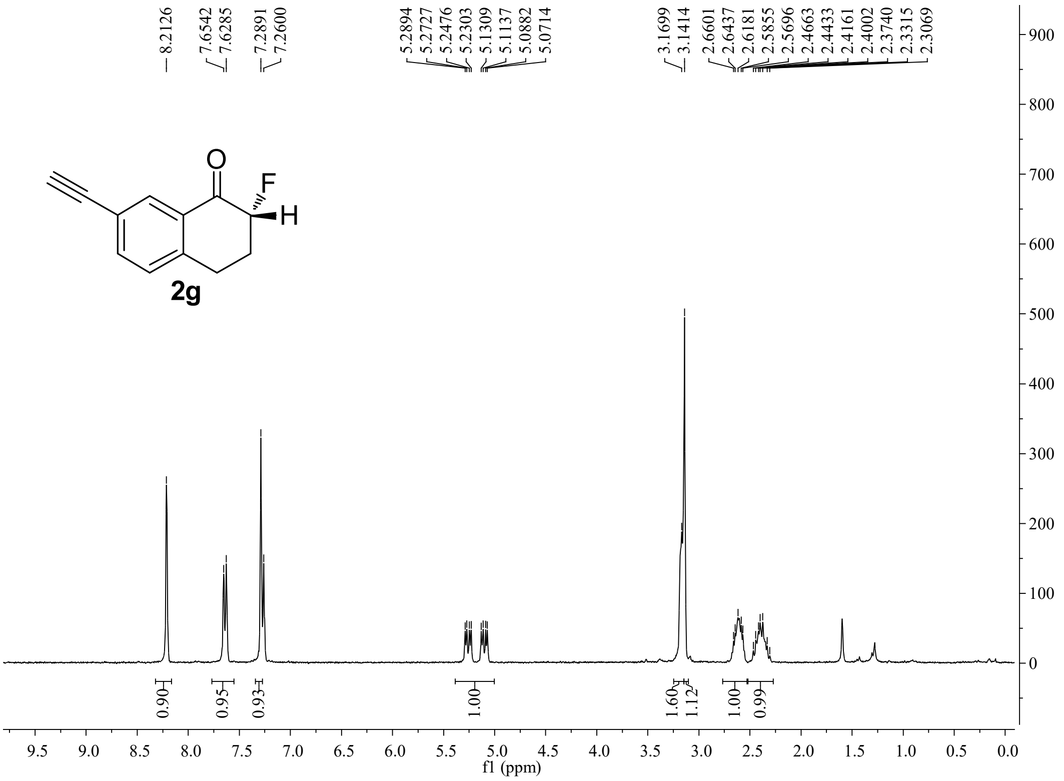

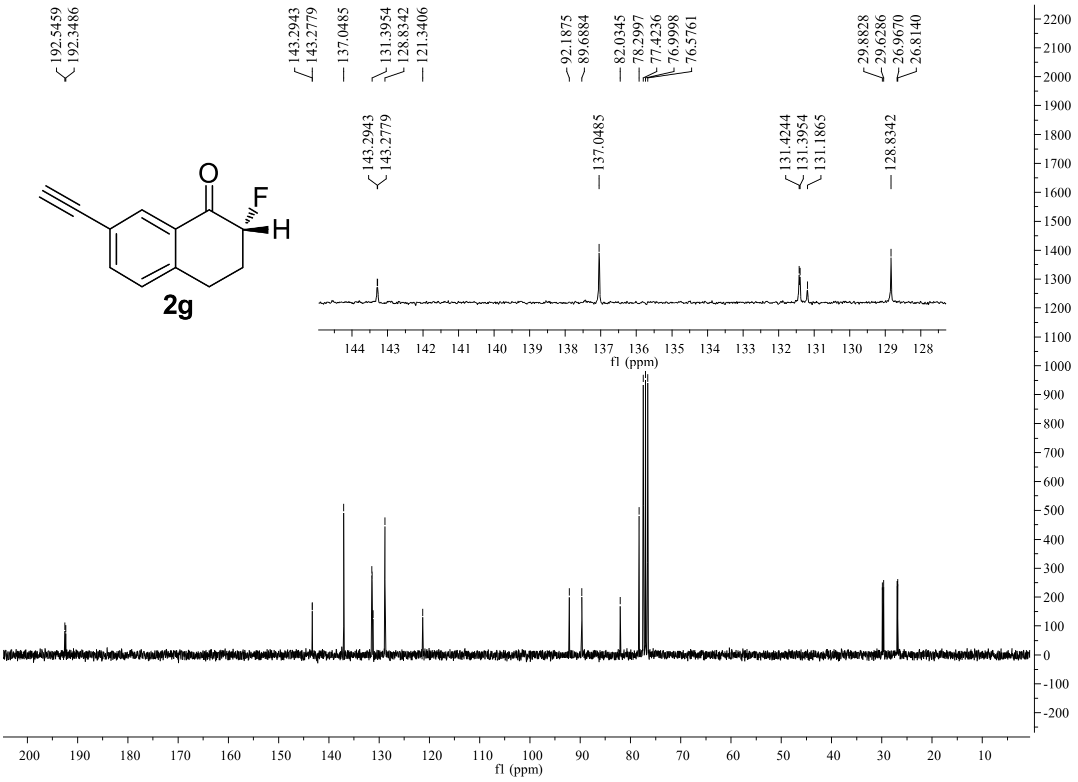

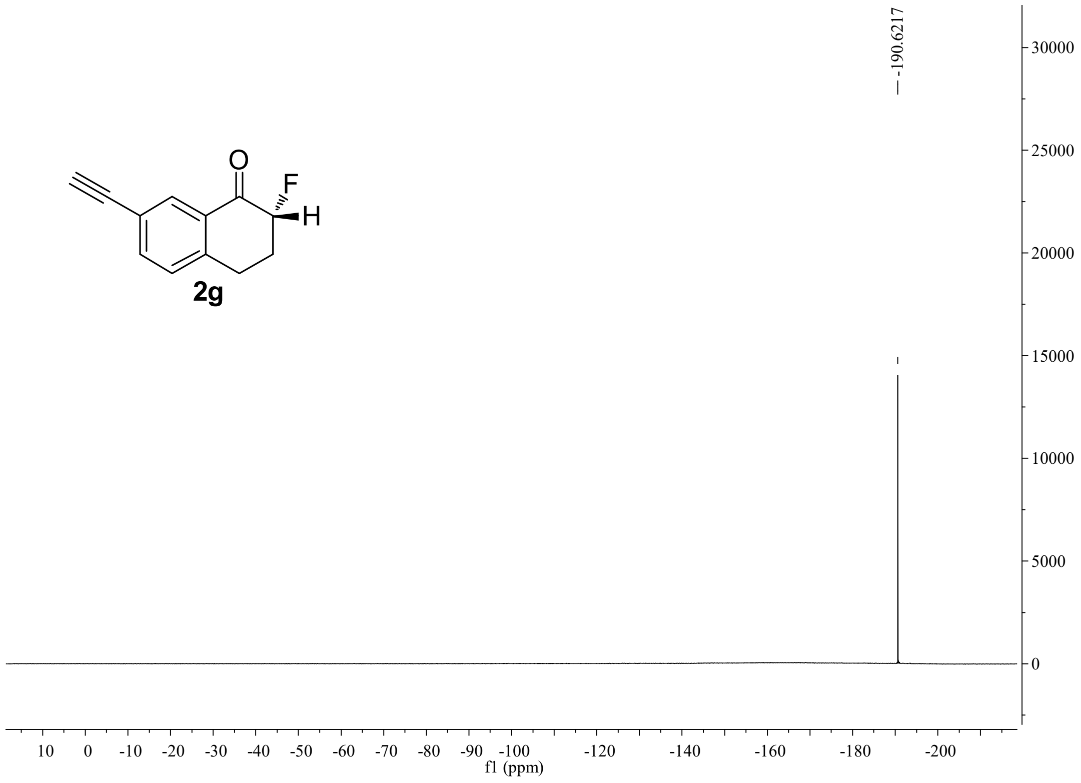
2g
-190.6217
f1 (ppm)

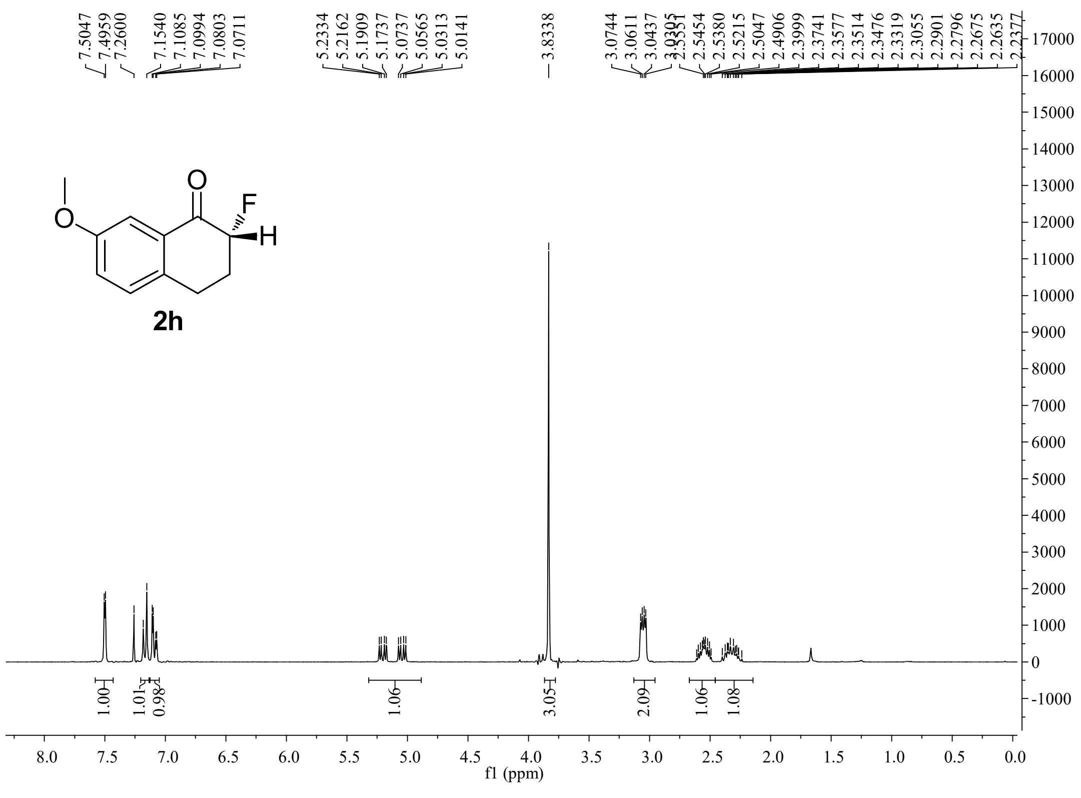
2h
3.8338
f1 (ppm)

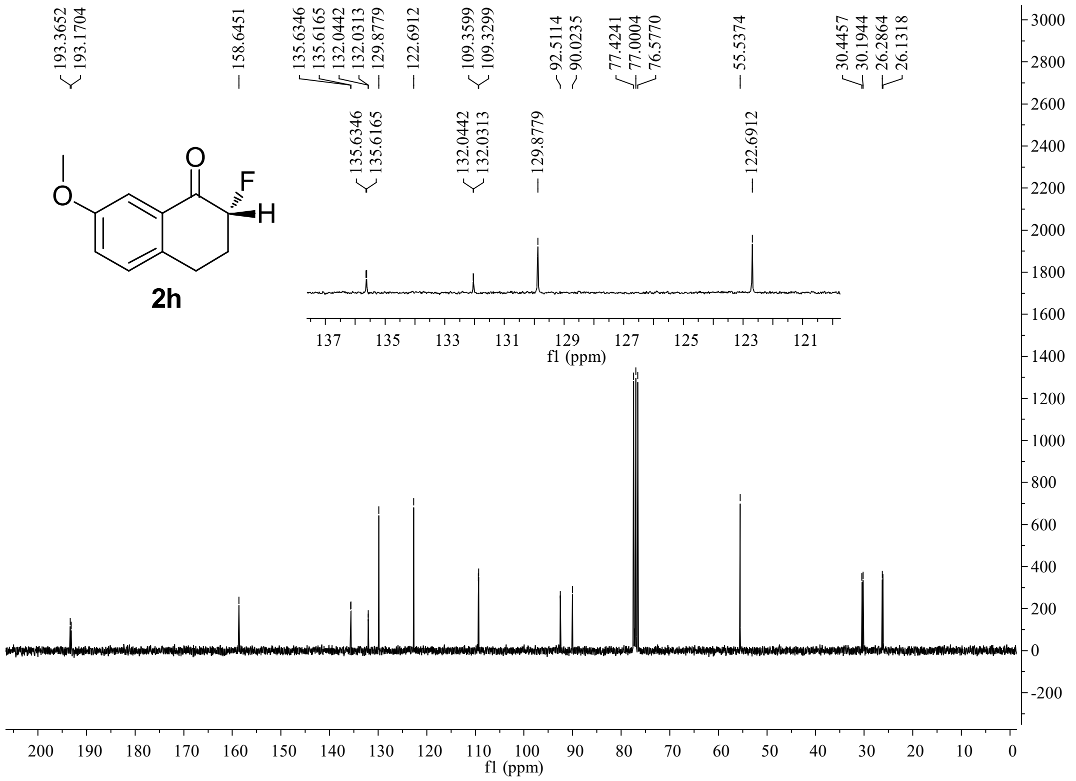
2h
193.3652
193.1704
158.6451
135.6346
135.6165
132.0442
132.0313
129.8779
122.6912
109.3599
109.3299
92.5114
90.0235
77.4241
77.0004
76.5770
55.5374
30.4457
30.1944
26.2864
26.1318
f1 (ppm)

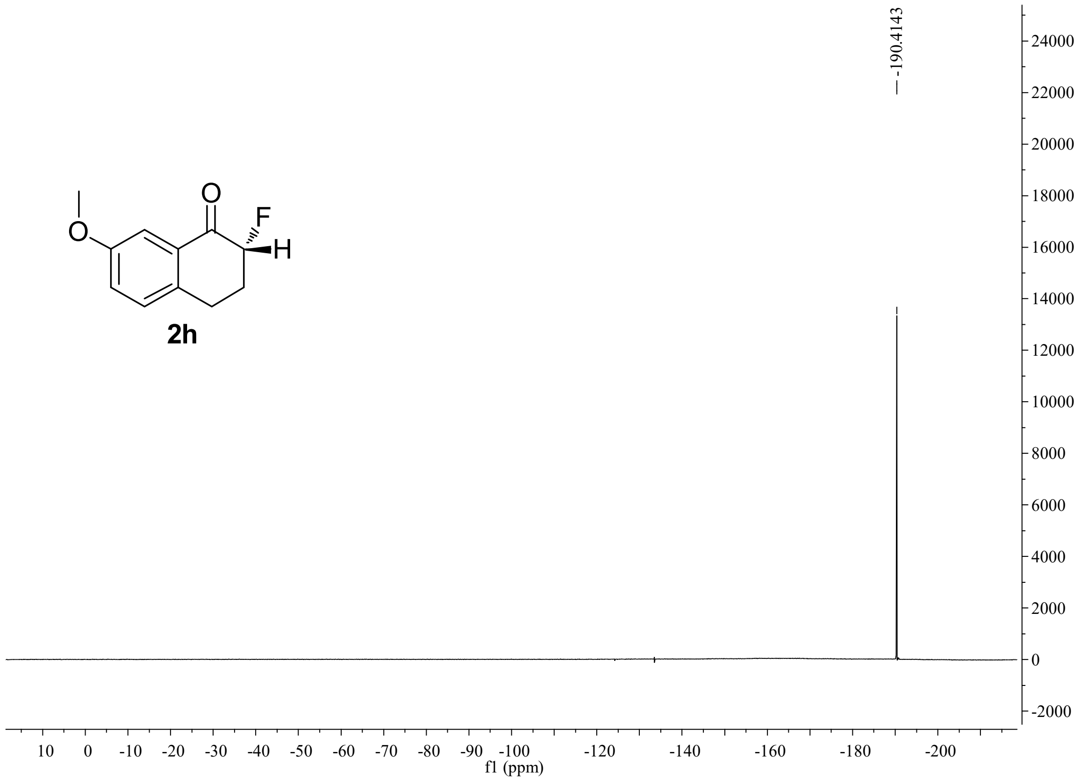
2h
-190.4143
f1 (ppm)

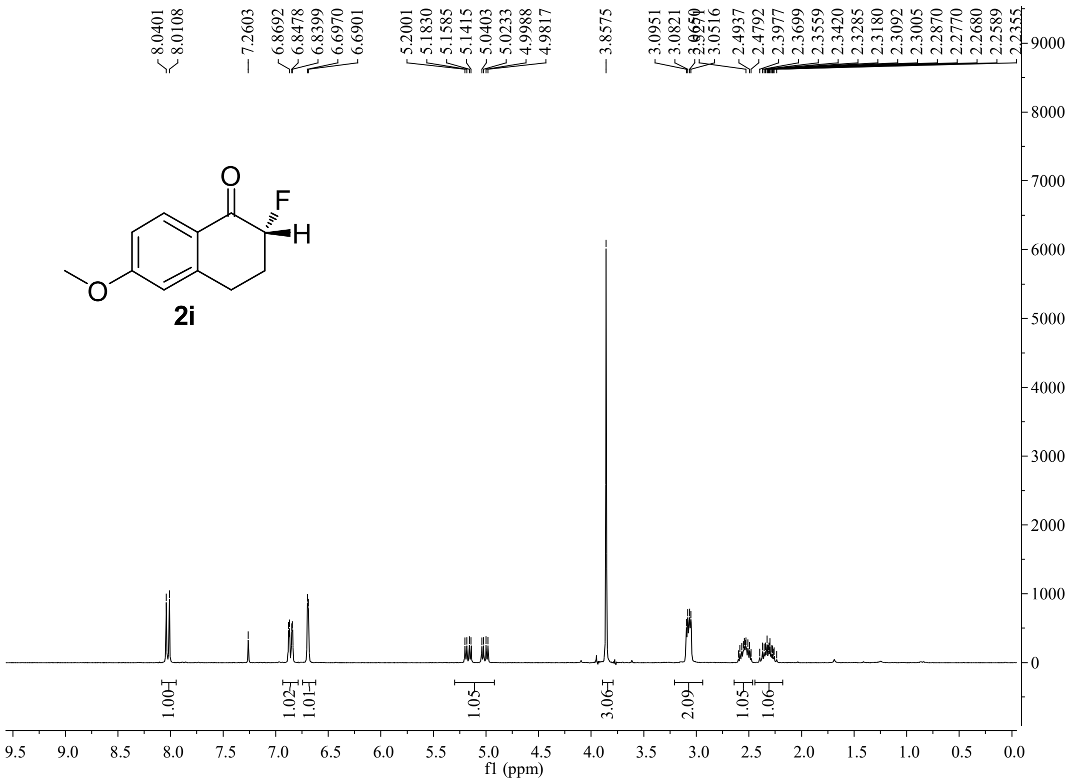
2i
O
F
H
8.0401
8.0108
7.2603
6.8692
6.8478
6.8399
6.6970
6.6901
5.2001
5.1830
5.1585
5.1415
5.0403
5.0233
4.9988
4.9817
3.8575
3.0951
3.0821
3.0516
2.4937
2.4792
2.3977
2.3699
2.3559
2.3420
2.3285
2.3180
2.3092
2.3005
2.2870
2.2770
2.2680
2.2589
1.00
1.02
1.01
1.05
3.06
2.09
1.05
1.06
f1 (ppm)

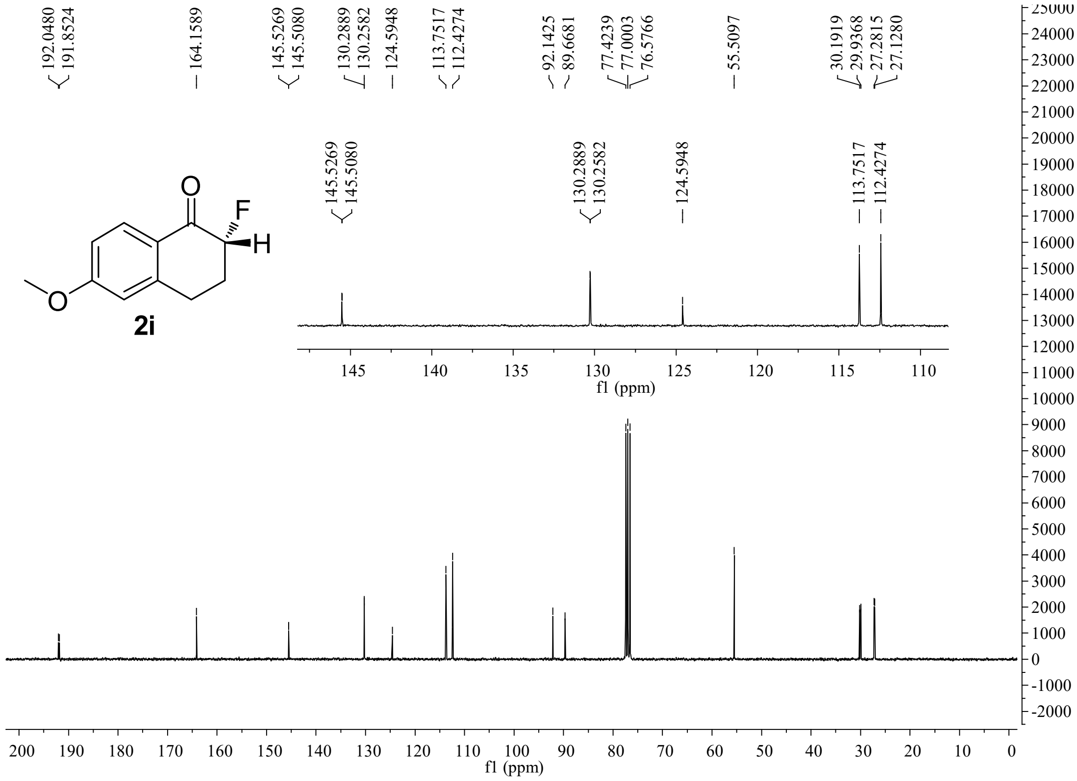
2i
O
F
H
192.0480
191.8524
164.1589
145.5269
145.5080
130.2889
130.2582
124.5948
113.7517
112.4274
92.1425
89.6681
77.4239
77.0003
76.5766
55.5097
30.1919
29.9368
27.2815
27.1280
f1 (ppm)

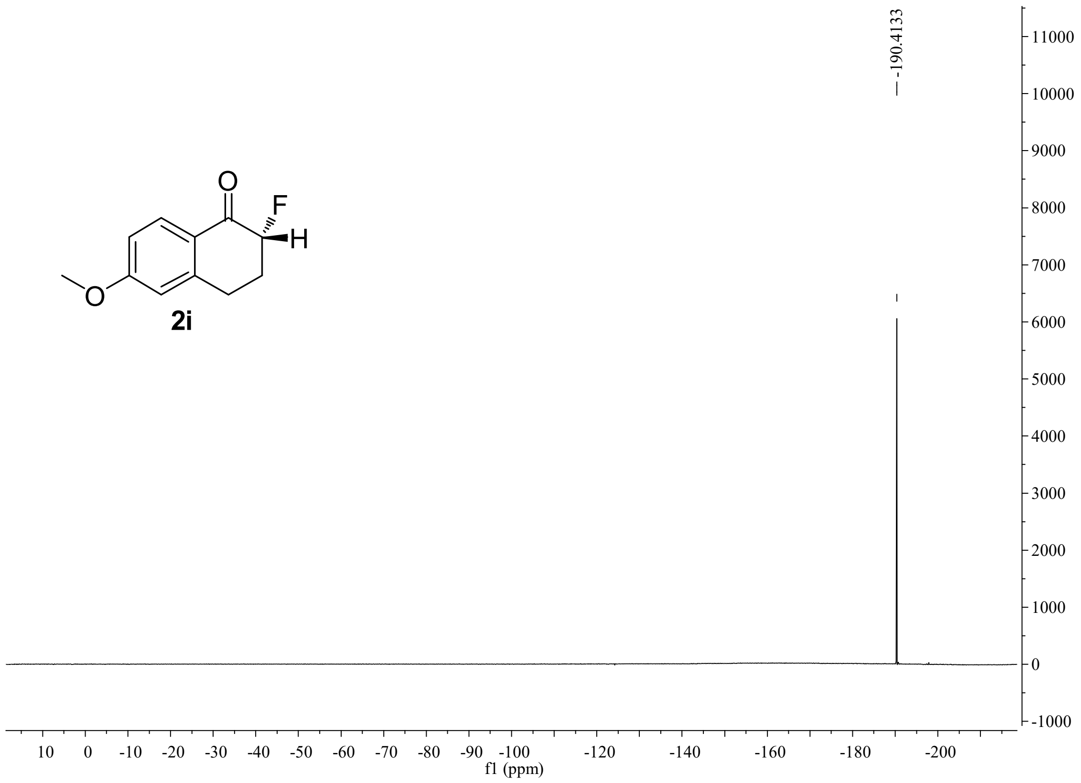

2i

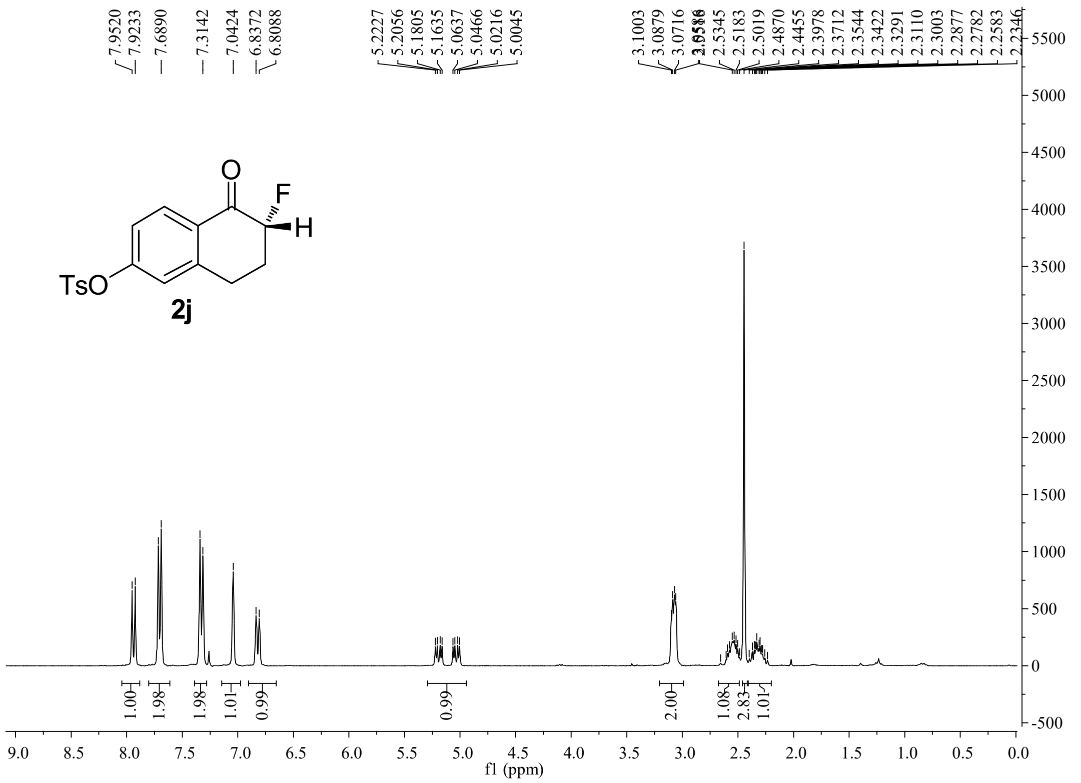

2j

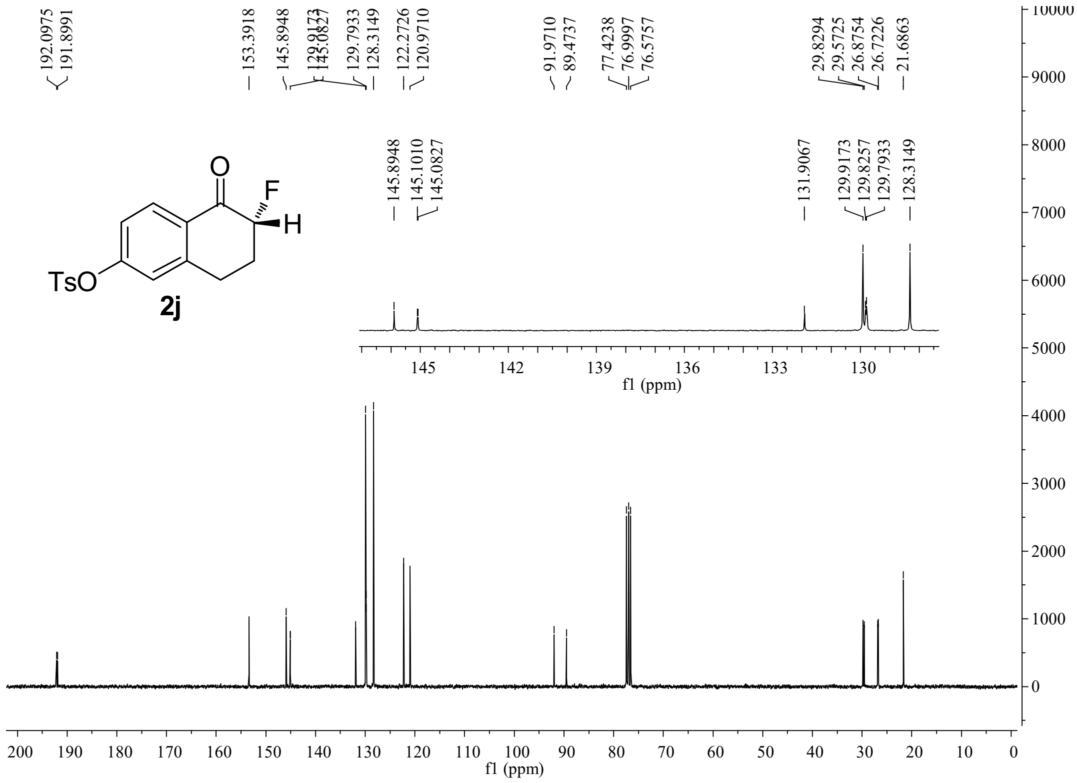
192.0975
191.8991
153.3918
145.8948
129.9173
145.0827
129.7933
128.3149
122.2726
120.9710
91.9710
89.4737
77.4238
76.9997
76.5757
29.8294
29.5725
26.8754
26.7226
21.6863
145.8948
145.1010
145.0827
131.9067
129.9173
129.8257
129.7933
128.3149
TsO
O
F
H
2j
f1 (ppm)

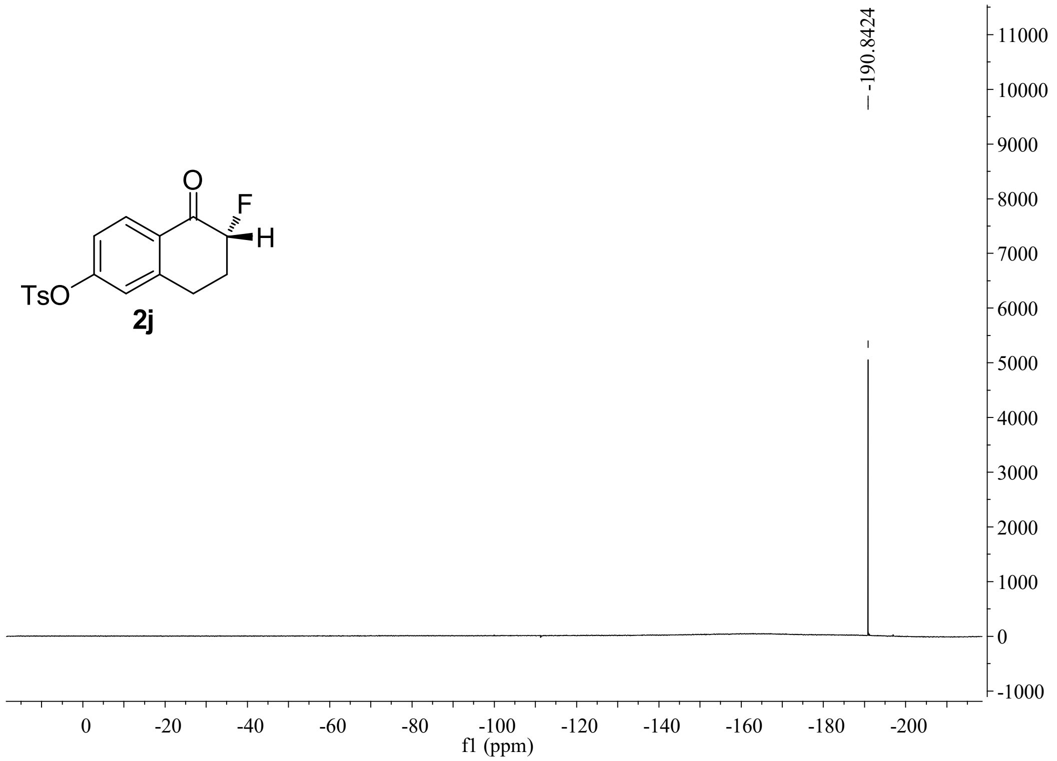
-190.8424
TsO
O
F
H
2j
f1 (ppm)

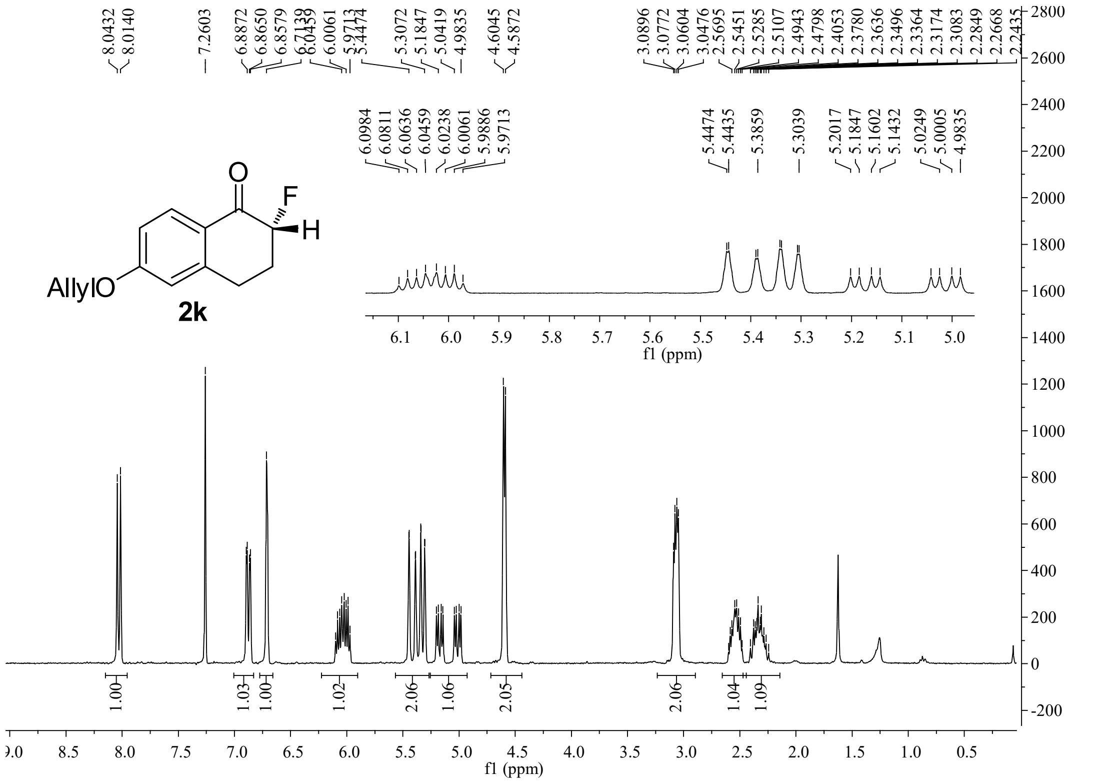
8.0432
8.0140
7.2603
6.8872
6.8650
6.8579
4.6045
4.5872
6.0984
6.0811
6.0636
6.0459
6.0238
6.0061
5.9886
5.9713
5.4474
5.4435
5.3859
5.3039
5.2017
5.1847
5.1602
5.1432
5.0249
5.0005
4.9835
O
F
H
AllylO
2k
f1 (ppm)
1.00
1.03
1.00
1.02
2.06
1.06
2.05
2.06
1.04
1.09

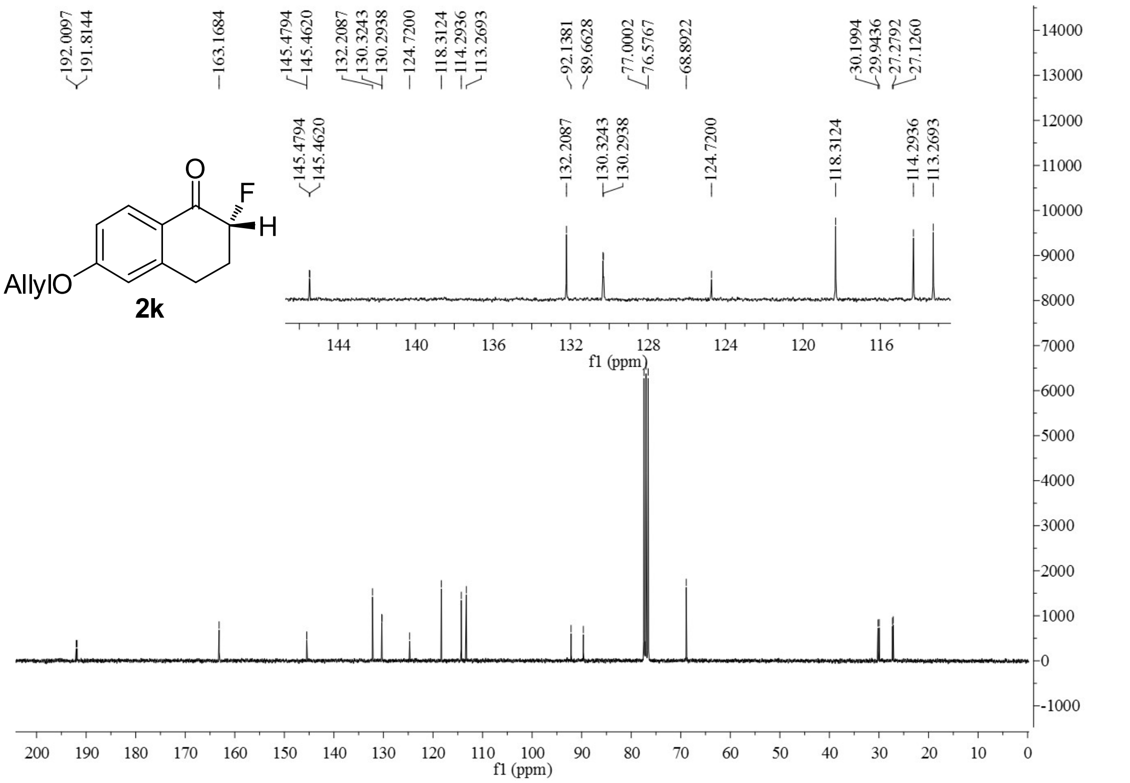
192.0097
191.8144
163.1684
145.4794
145.4620
132.2087
130.3243
130.2938
124.7200
118.3124
114.2936
113.2693
92.1381
89.6628
77.0002
76.5767
68.8922
30.1994
29.9436
27.2792
27.1260
O
F
H
AllylO
2k
f1 (ppm)

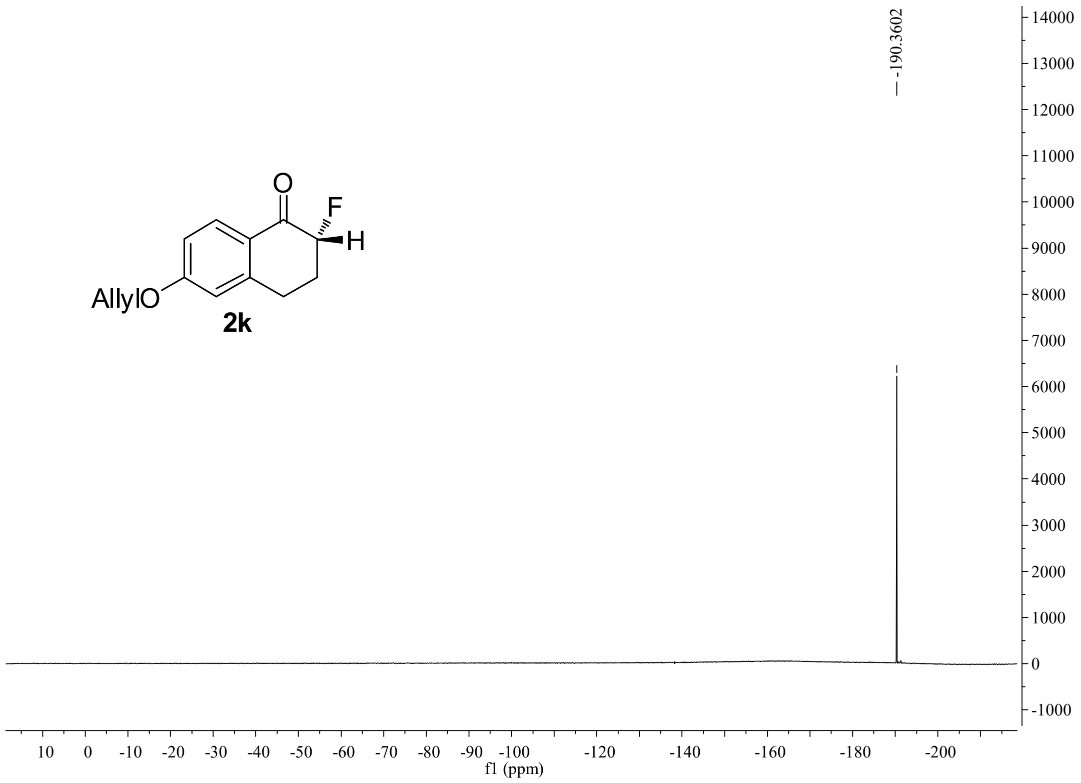

AllylO
O
F
H
2k
-190.3602
f1 (ppm)

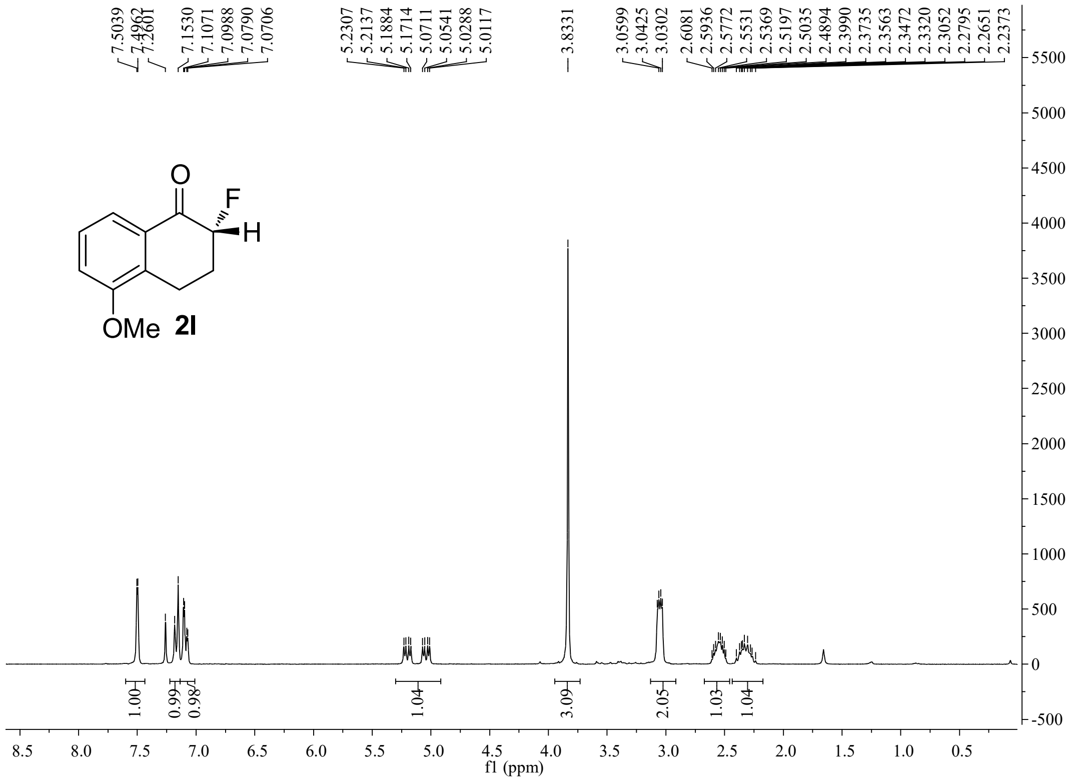

7.5039
7.4962
7.2601
7.1530
7.1071
7.0988
7.0790
7.0706
5.2307
5.2137
5.1884
5.1714
5.0711
5.0541
5.0288
5.0117
3.8331
3.0599
3.0425
3.0302
2.6081
2.5936
2.5772
2.5531
2.5369
2.5197
2.5035
2.4894
2.3990
2.3735
2.3563
2.3472
2.3320
2.3052
2.2795
2.2651
2.2373
O
F
H
OMe
2l
1.00
0.99
0.98
1.04
3.09
2.05
1.03
1.04
f1 (ppm)

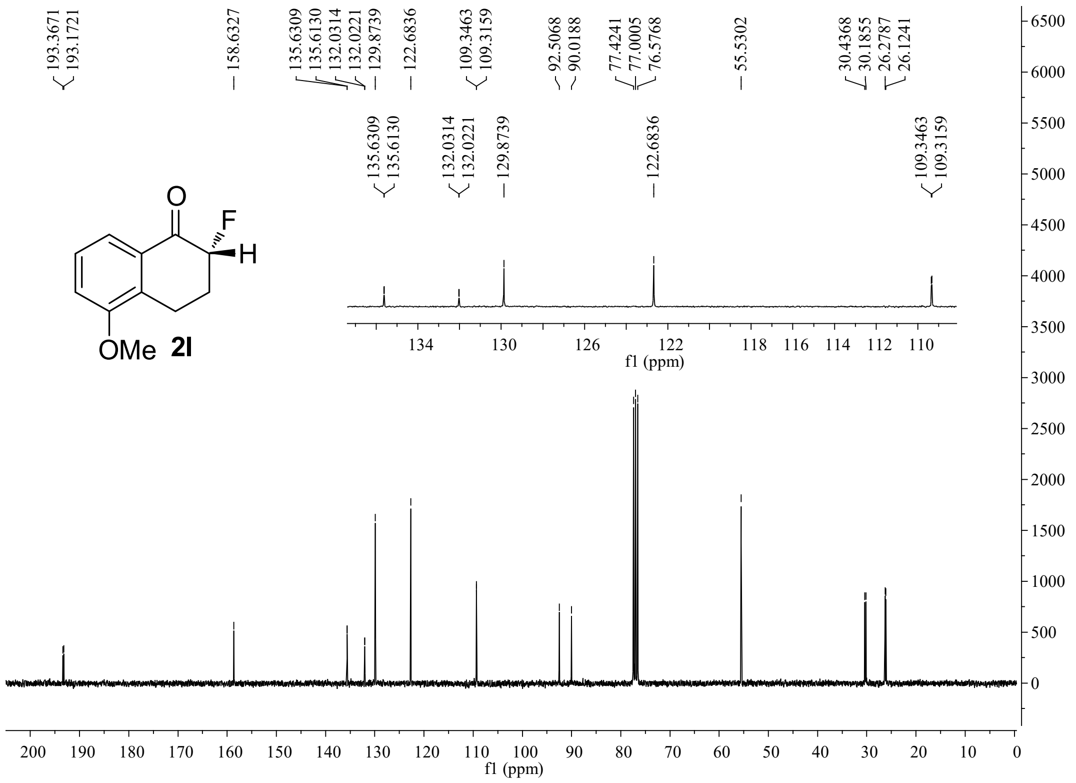

2l

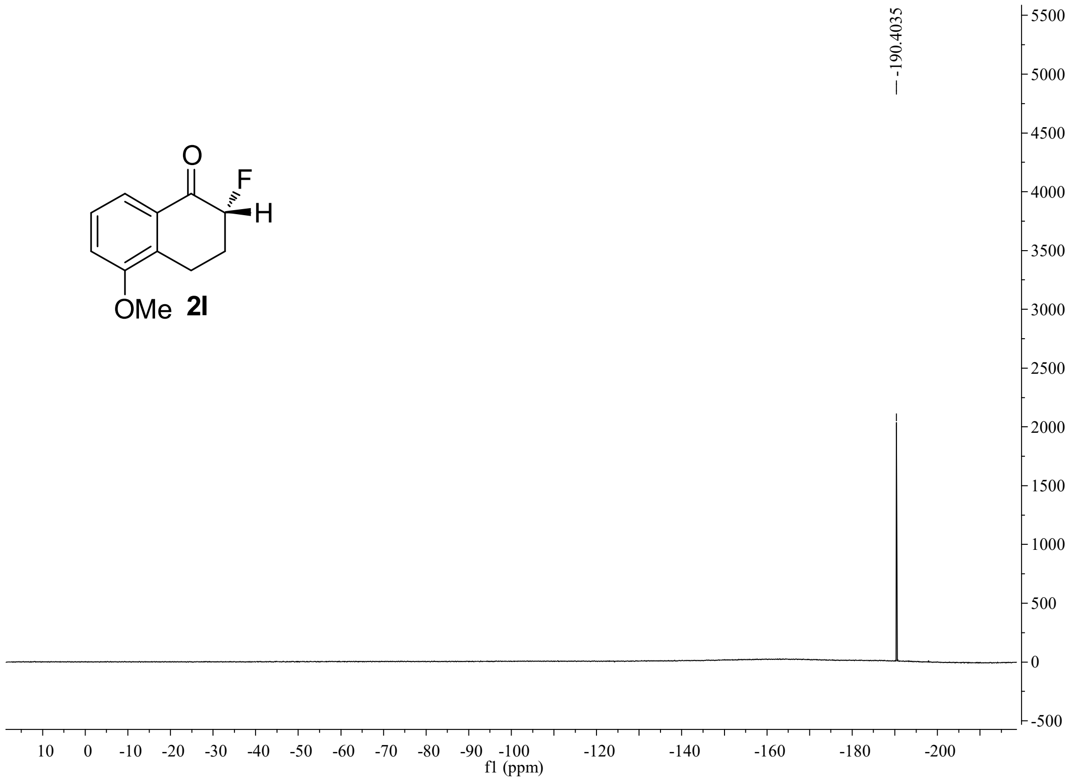

2l

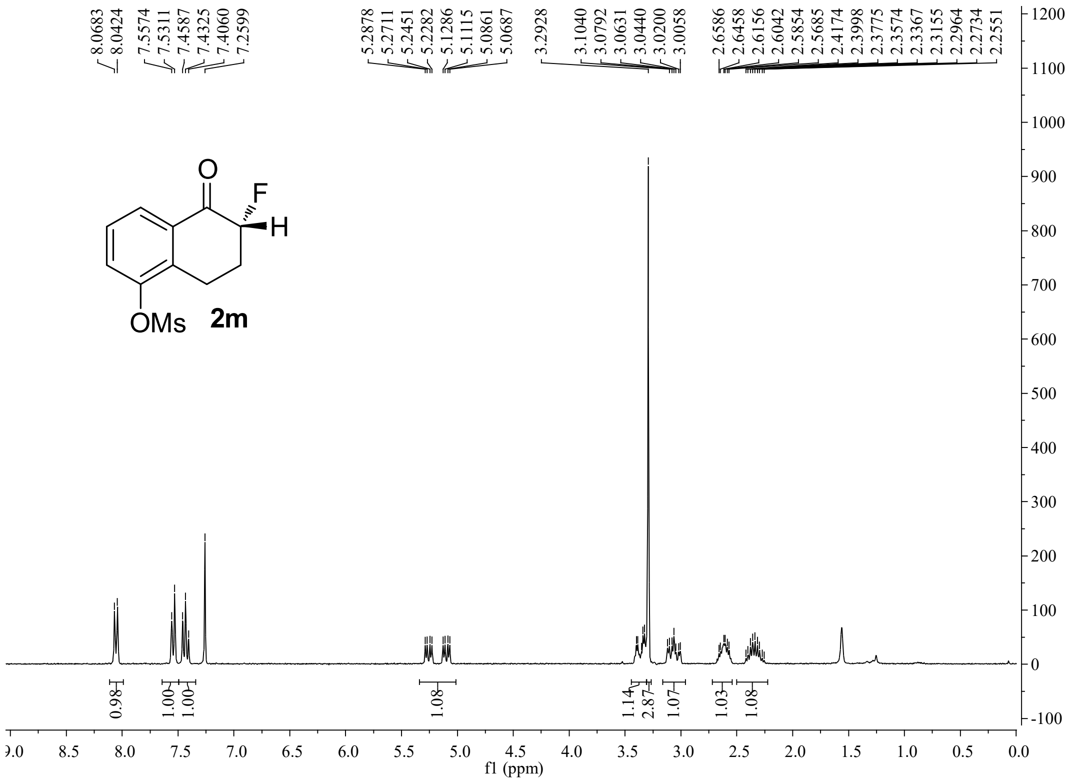
8.0683
8.0424
7.5574
7.5311
7.4587
7.4325
7.4060
7.2599
5.2878
5.2711
5.2451
5.2282
5.1286
5.1115
5.0861
5.0687
3.2928
3.1040
3.0792
3.0631
3.0440
3.0200
3.0058
2.6586
2.6458
2.6156
2.6042
2.5854
2.5685
2.4174
2.3998
2.3775
2.3574
2.3367
2.3155
2.2964
2.2734
2.2551
O
F
H
OMs
2m
0.98
1.00
1.00
1.08
1.14
2.87
1.07
1.03
1.08
f1 (ppm)

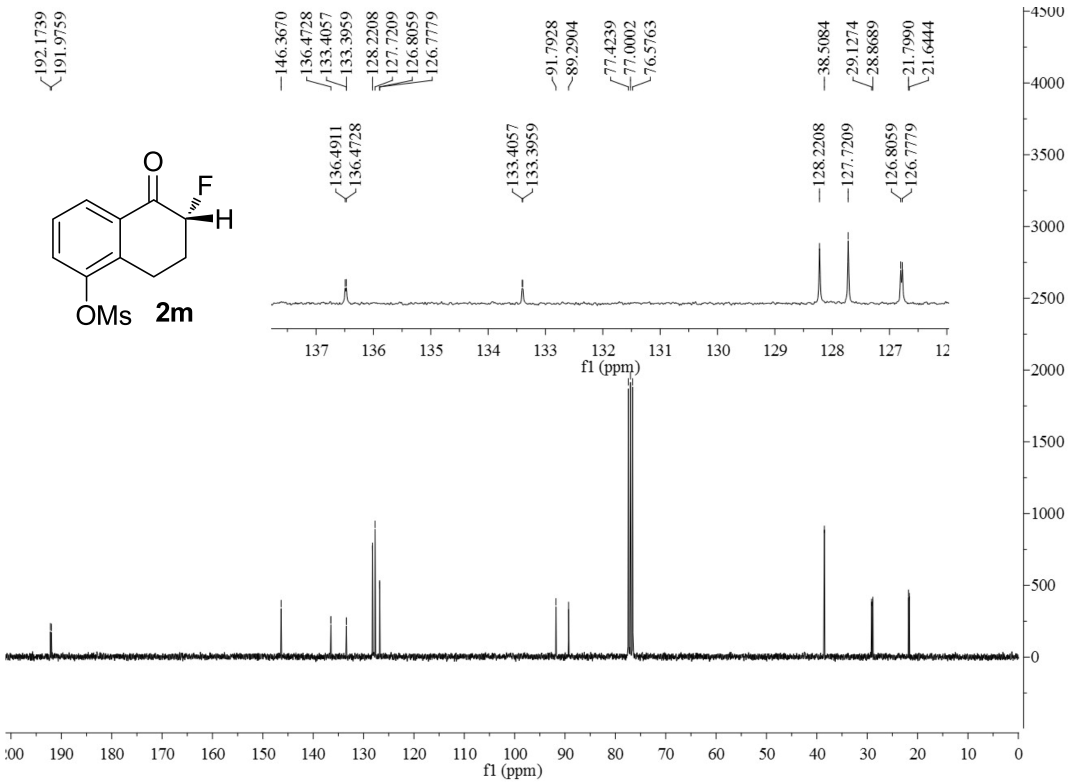
192.1739
191.9759
146.3670
136.4728
133.4057
133.3959
128.2208
127.7209
126.8059
126.7779
91.7928
89.2904
77.4239
77.0002
76.5763
38.5084
29.1274
28.8689
21.7990
21.6444
136.4911
136.4728
133.4057
133.3959
128.2208
127.7209
126.8059
126.7779
O
F
H
OMs
2m
f1 (ppm)
f1 (ppm)

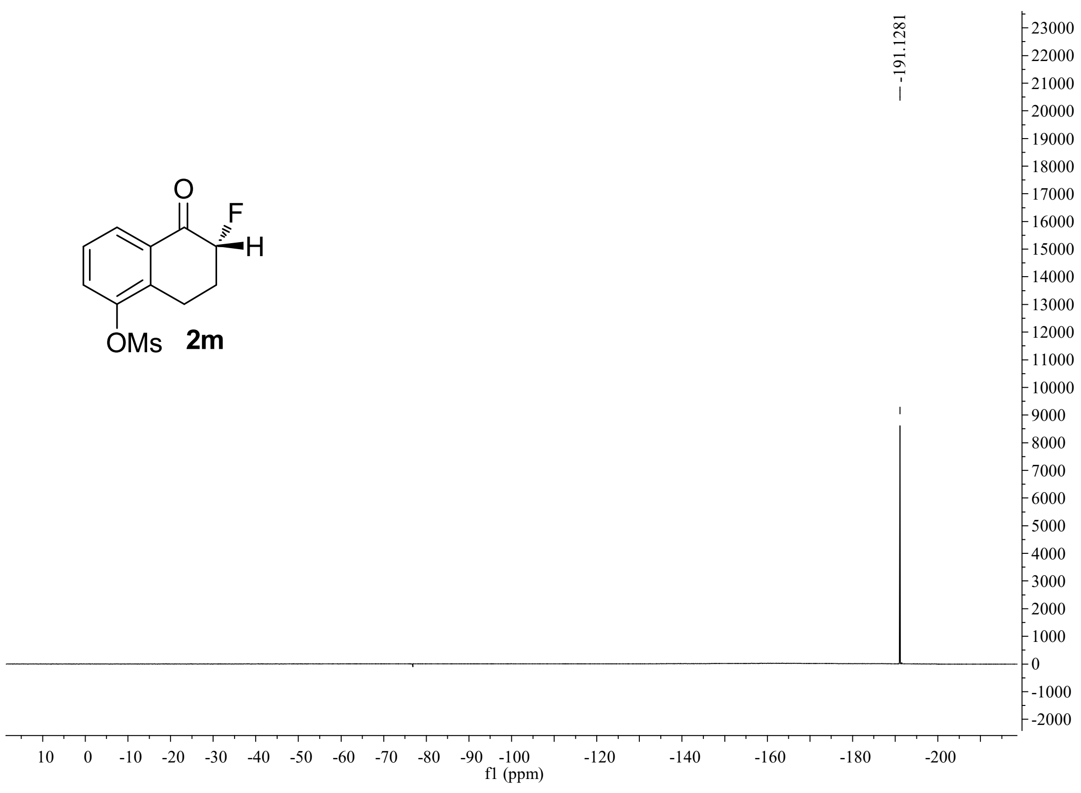
-191.1281
OMs 2m
fl (ppm)

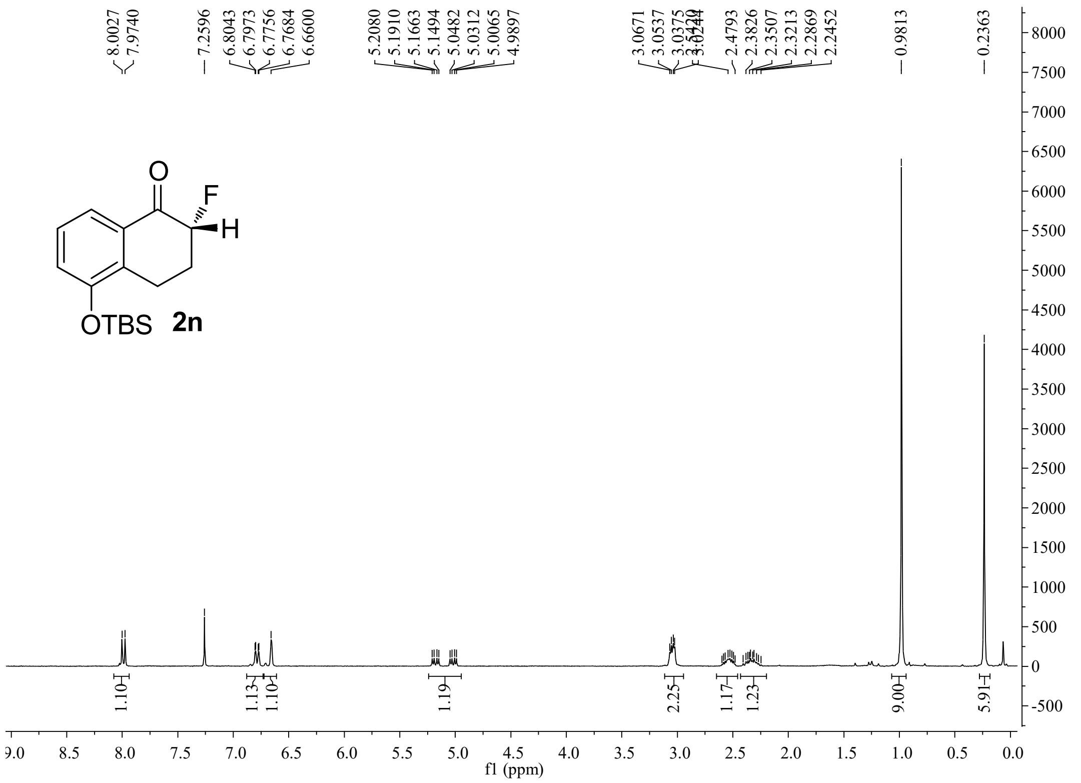
OTBS 2n
fl (ppm)

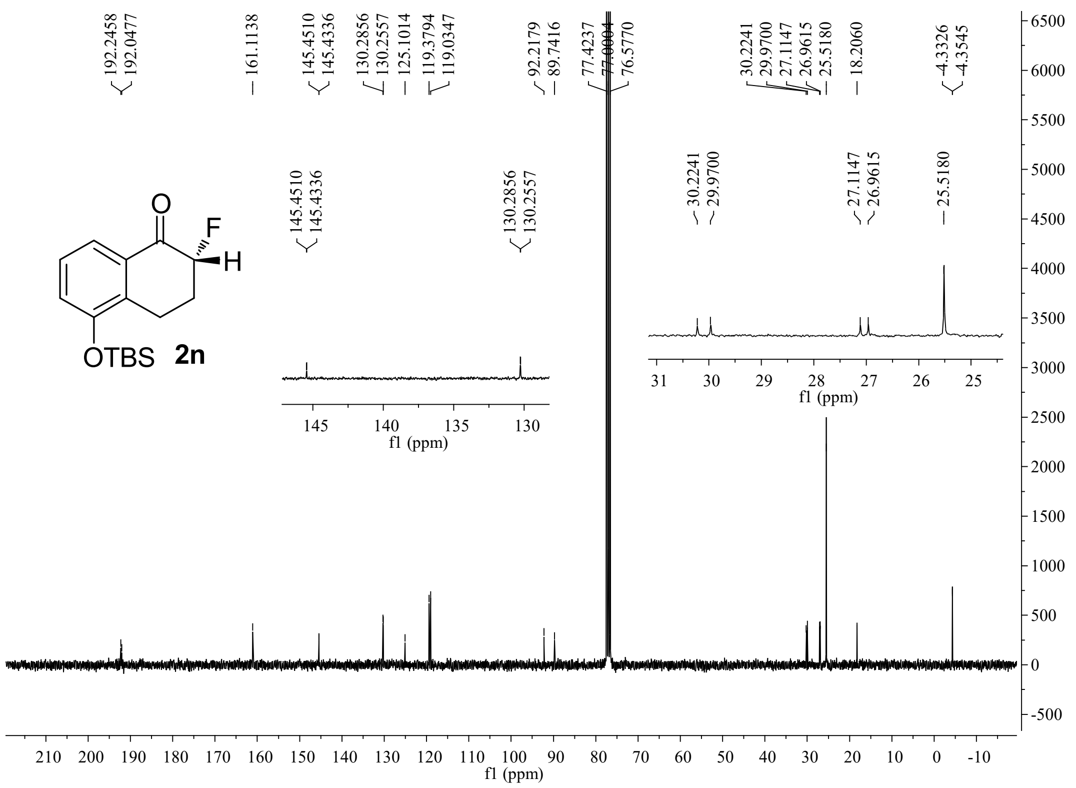

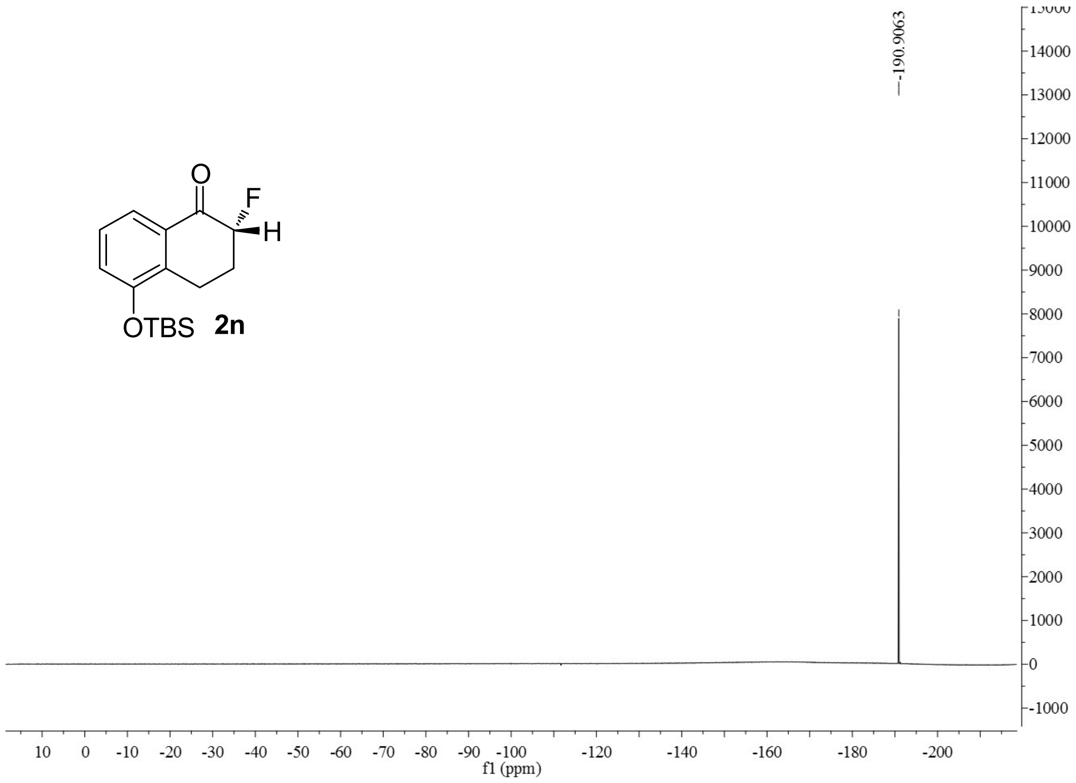

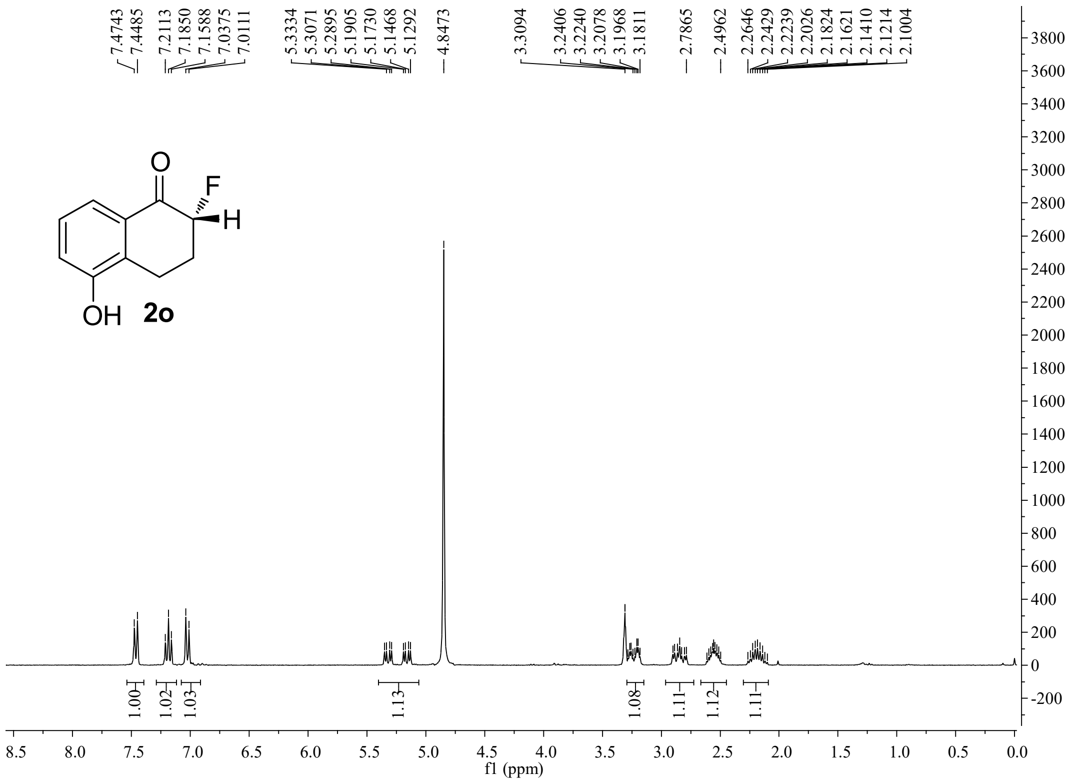
7.4743
7.4485
7.2113
7.1850
7.1588
7.0375
7.0111
5.3334
5.3071
5.2895
5.1905
5.1730
5.1468
5.1292
4.8473
3.3094
3.2406
3.2240
3.2078
3.1968
3.1811
2.7865
2.4962
2.2646
2.2429
2.2239
2.2026
2.1824
2.1621
2.1410
2.1214
2.1004
O
F
H
OH
2o
1.00
1.02
1.03
1.13
1.08
1.11
1.12
1.11
f1 (ppm)

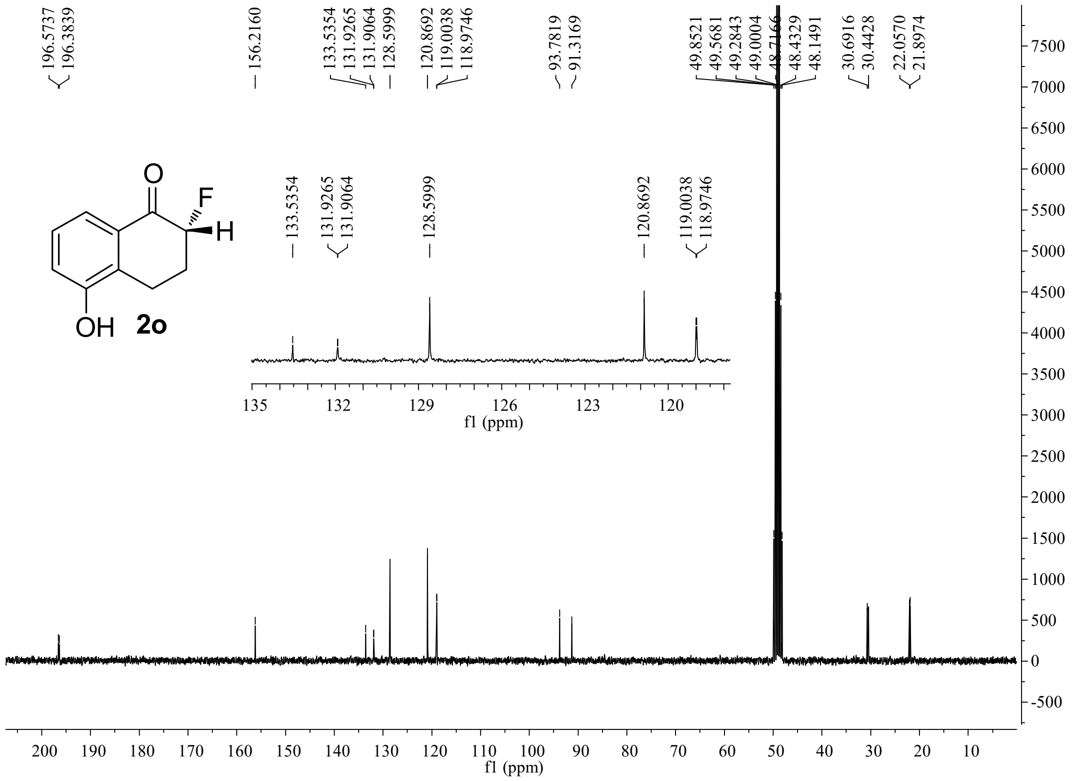
196.5737
196.3839
156.2160
133.5354
131.9265
131.9064
128.5999
120.8692
119.0038
118.9746
93.7819
91.3169
49.8521
49.5681
49.2843
49.0004
48.4329
48.1491
30.6916
30.4428
22.0570
21.8974
O
F
H
OH
2o
f1 (ppm)

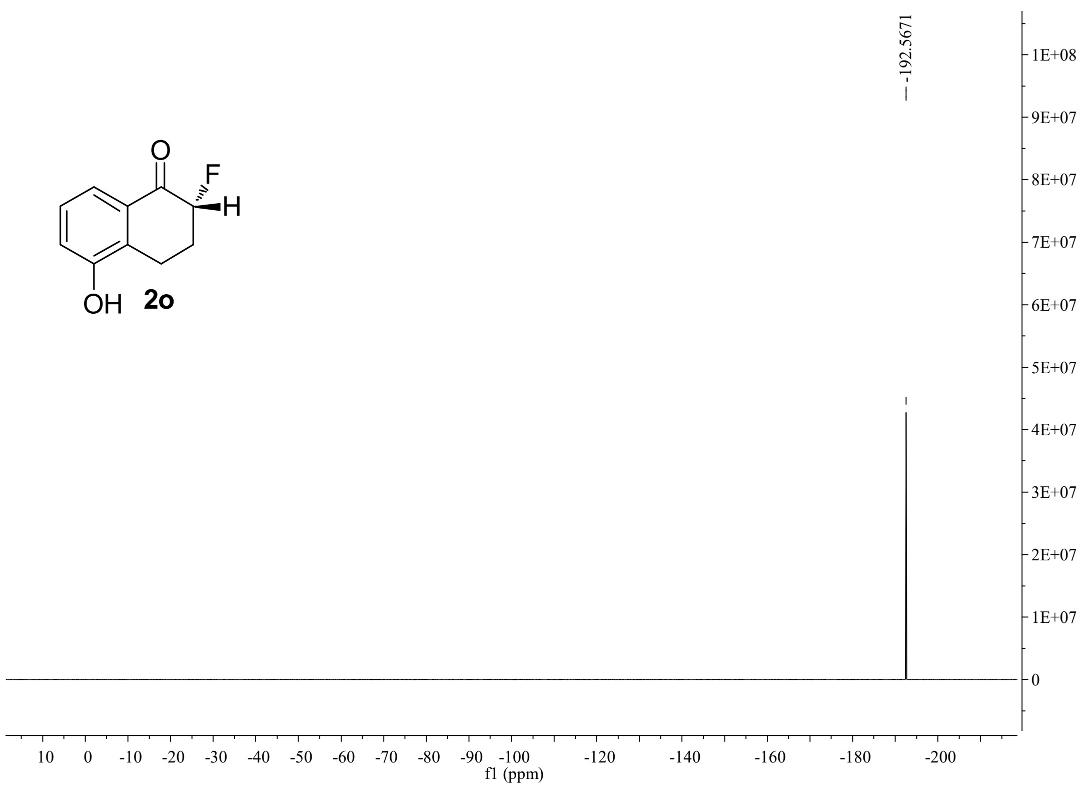
--192.5671
O
F
H
OH
2o
1E+08
9E+07
8E+07
7E+07
6E+07
5E+07
4E+07
3E+07
2E+07
1E+07
0
10 0 -10 -20 -30 -40 -50 -60 -70 -80 -90 -100 -120 -140 -160 -180 -200
f1 (ppm)

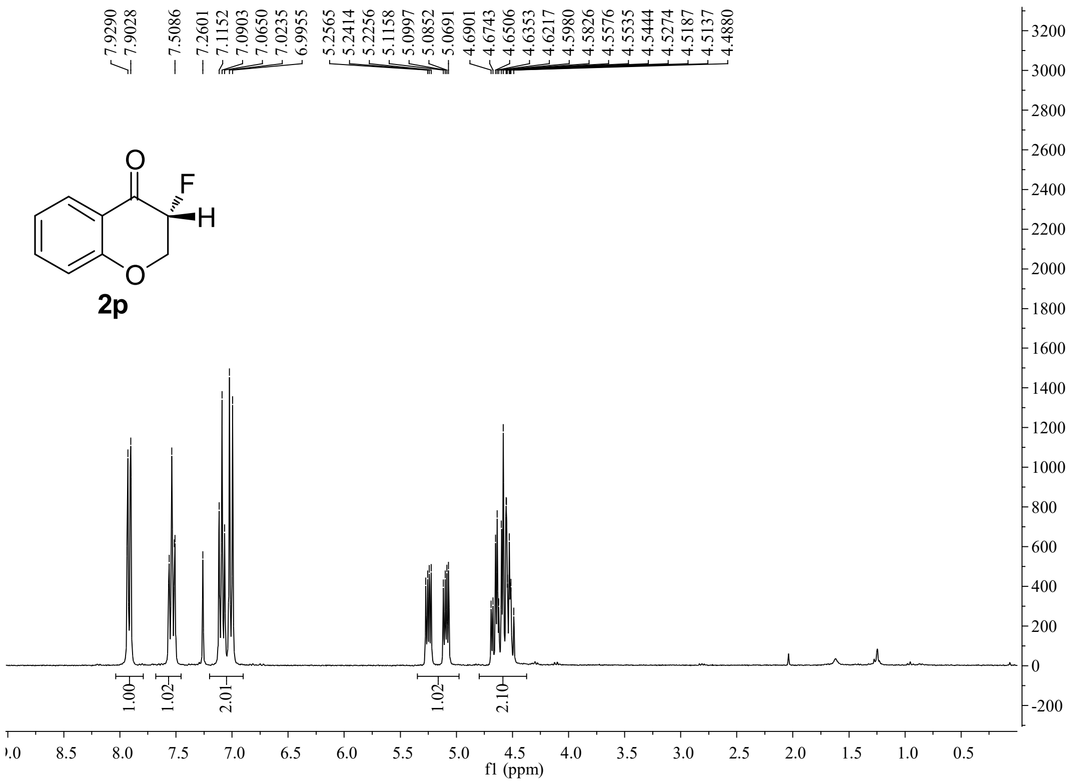
7.9290
7.9028
7.5086
7.2601
7.1152
7.0903
7.0650
7.0235
6.9955
5.2565
5.2414
5.2256
5.1158
5.0997
5.0852
5.0691
4.6901
4.6743
4.6506
4.6353
4.6217
4.5980
4.5826
4.5576
4.5535
4.5444
4.5274
4.5187
4.5137
4.4880
O
F
H
O
2p
1.00
1.02
2.01
1.02
2.10
3200
3000
2800
2600
2400
2200
2000
1800
1600
1400
1200
1000
800
600
400
200
0
-200
8.5 8.0 7.5 7.0 6.5 6.0 5.5 5.0 4.5 4.0 3.5 3.0 2.5 2.0 1.5 1.0 0.5
f1 (ppm)

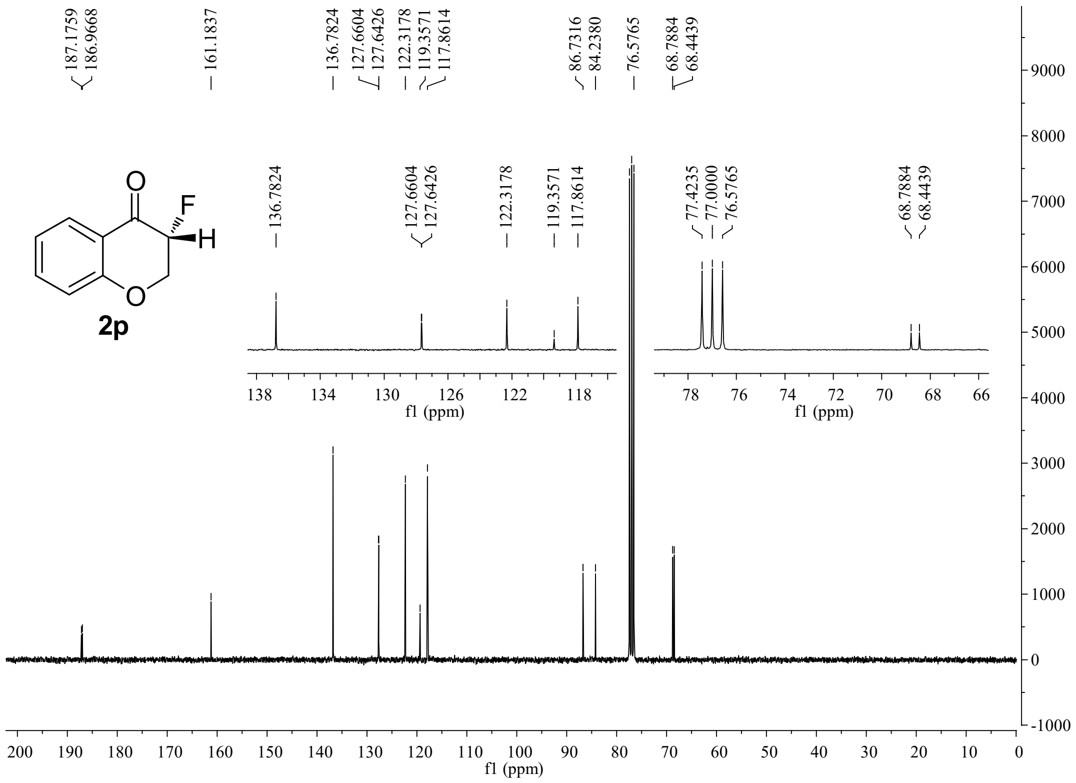
187.1759
186.9668
161.1837
136.7824
127.6604
127.6426
122.3178
119.3571
117.8614
86.7316
84.2380
76.5765
68.7884
68.4439
77.4235
77.0000
76.5765
2p
f1 (ppm)

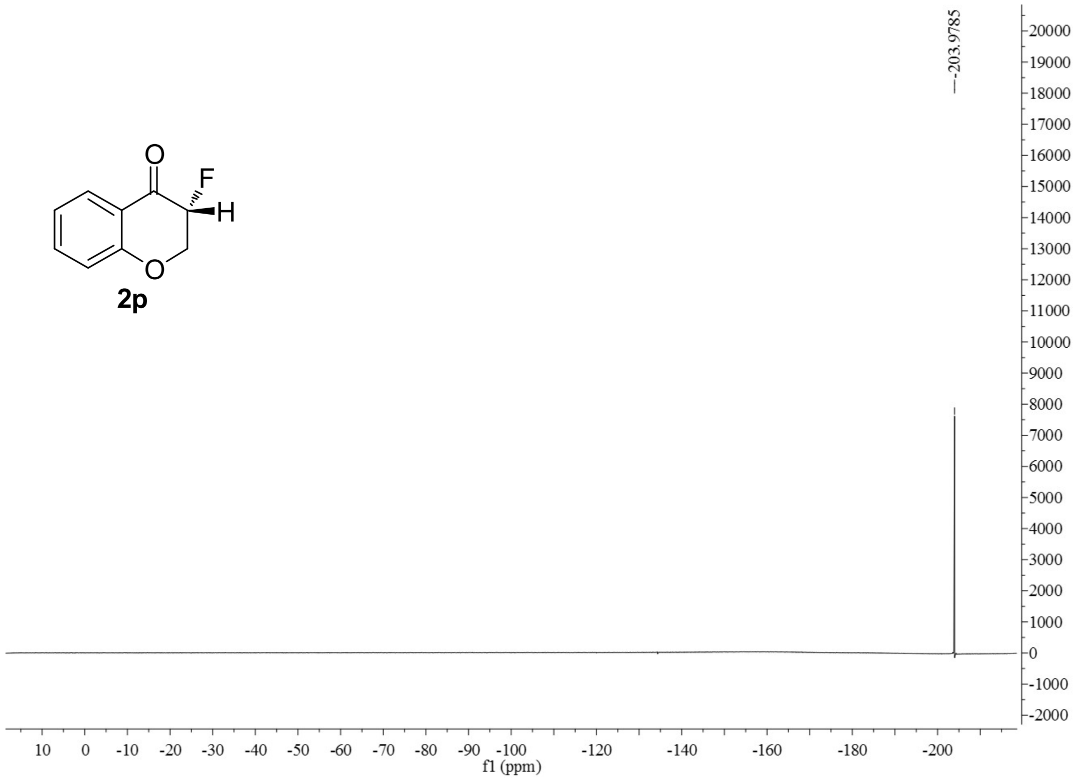
-203.9785
2p
f1 (ppm)

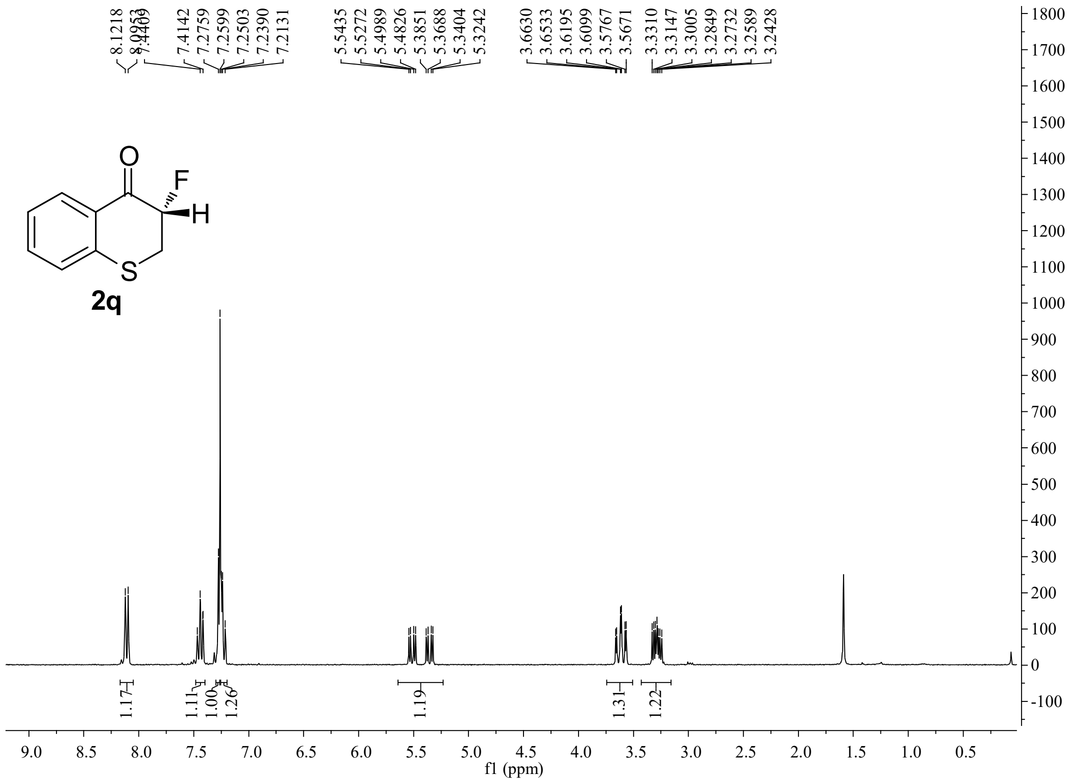
8.1218
7.4142
7.2759
7.2599
7.2503
7.2390
7.2131
5.5435
5.5272
5.4989
5.4826
5.3851
5.3688
5.3404
5.3242
3.6630
3.6533
3.6195
3.6099
3.5767
3.5671
3.3310
3.3147
3.3005
3.2849
3.2732
3.2589
3.2428
O
F
H
S
2q
1.17
1.11
1.00
1.26
1.19
1.31
1.22
f1 (ppm)

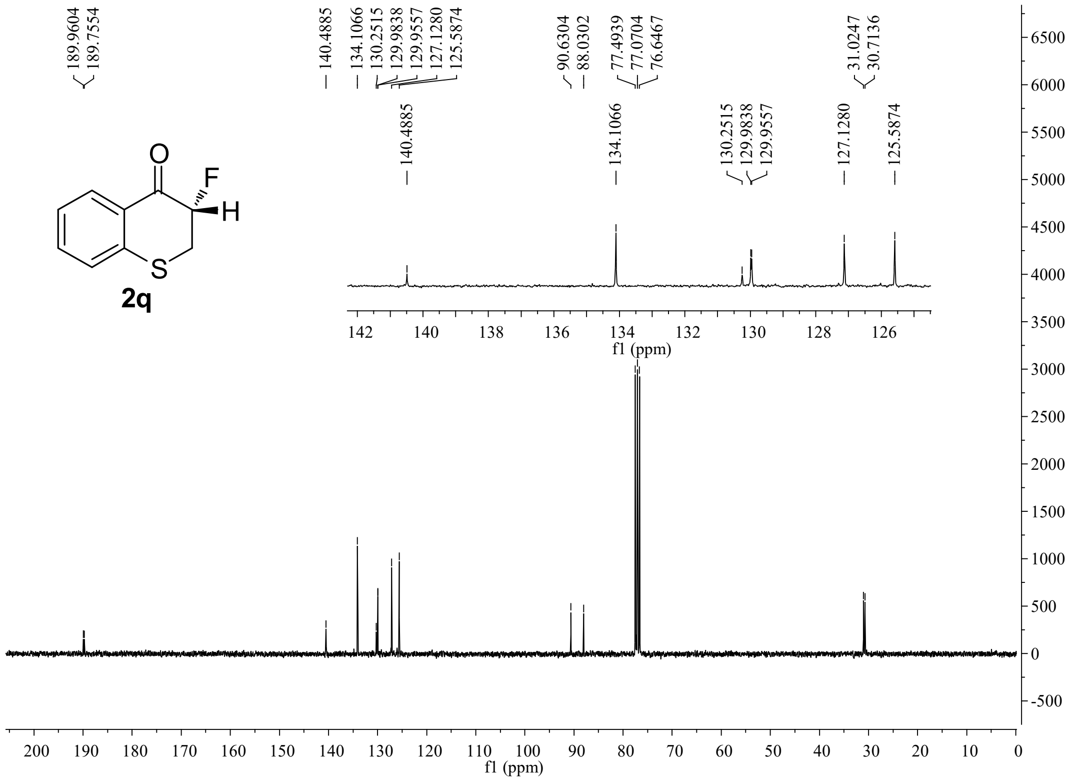
189.9604
189.7554
140.4885
134.1066
130.2515
129.9838
129.9557
127.1280
125.5874
90.6304
88.0302
77.4939
77.0704
76.6467
31.0247
30.7136
O
F
H
S
2q
f1 (ppm)

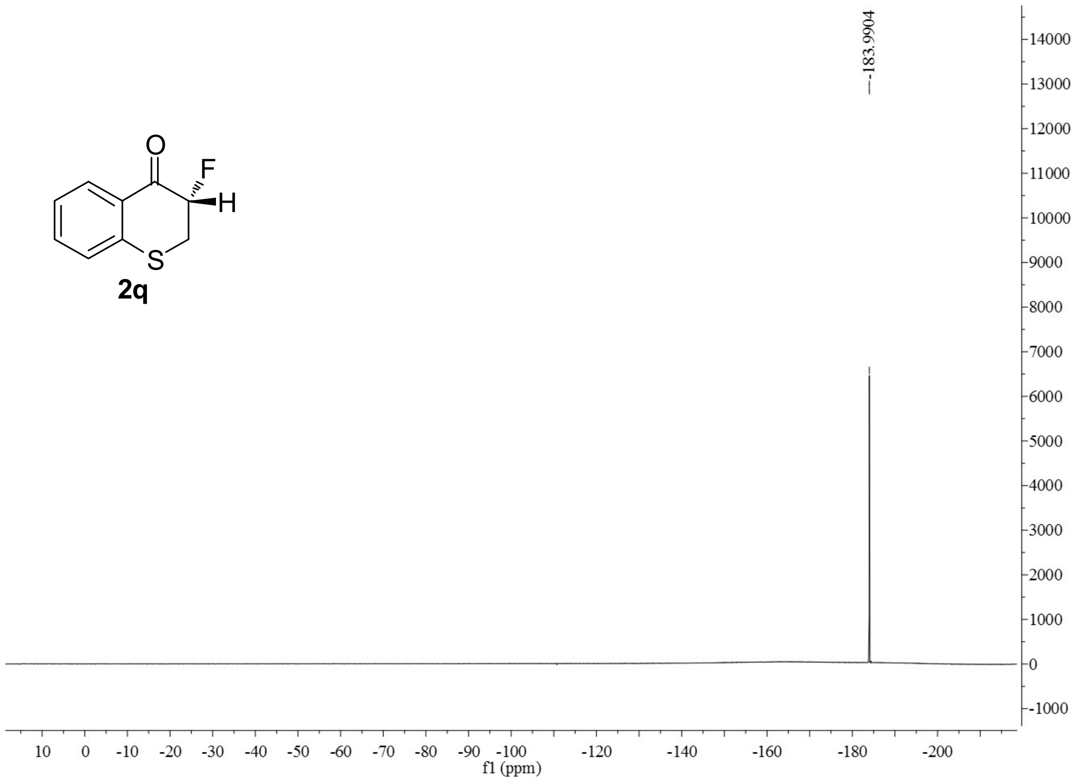

2q

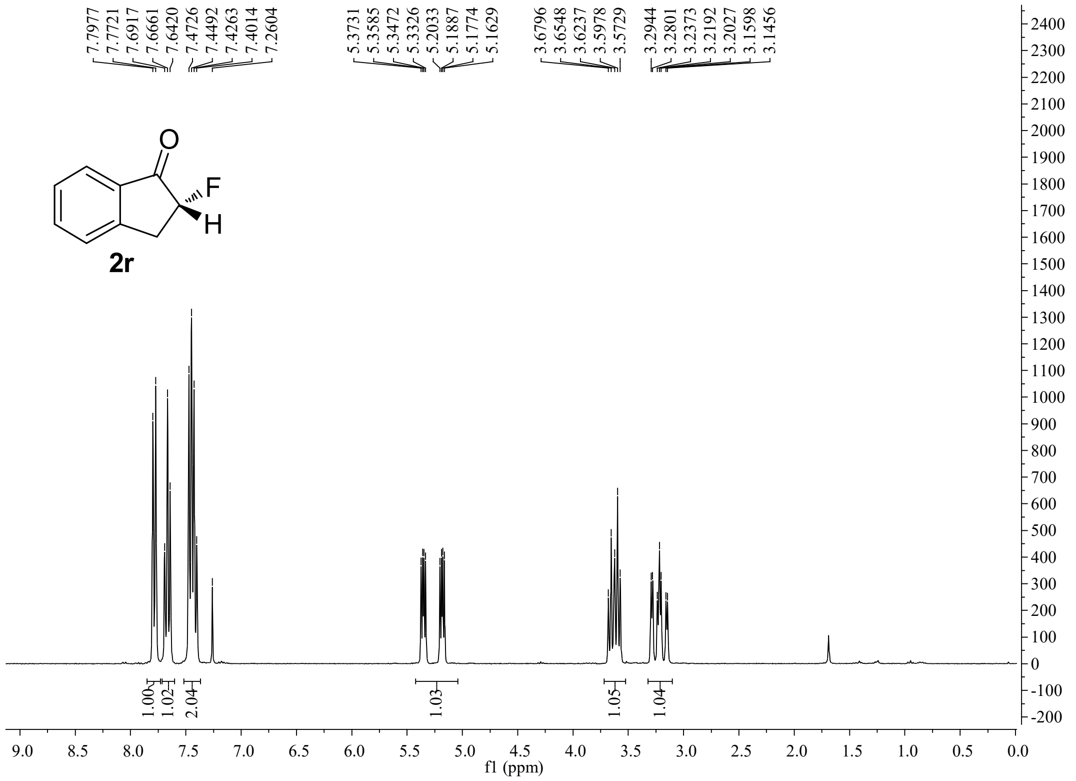

2r

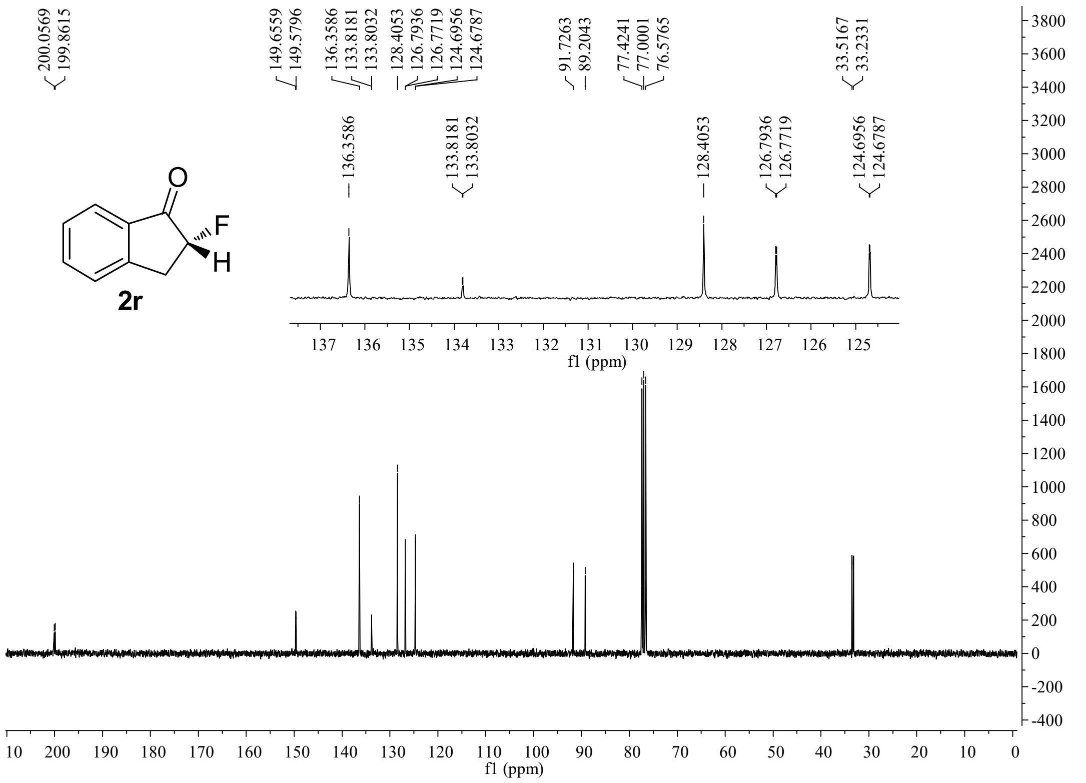
200.0569
199.8615
149.6559
149.5796
136.3586
133.8181
133.8032
128.4053
126.7936
126.7719
124.6956
124.6787
91.7263
89.2043
77.4241
77.0001
76.5765
33.5167
33.2331
2r
f1 (ppm)

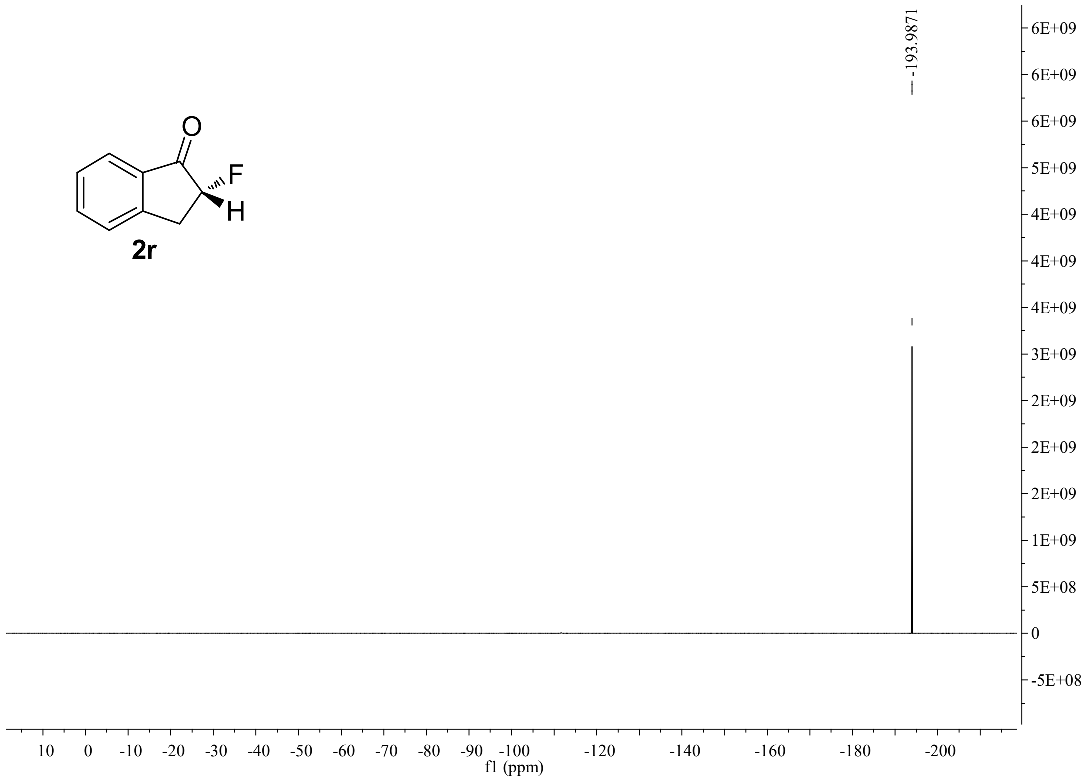
-193.9871
2r
f1 (ppm)

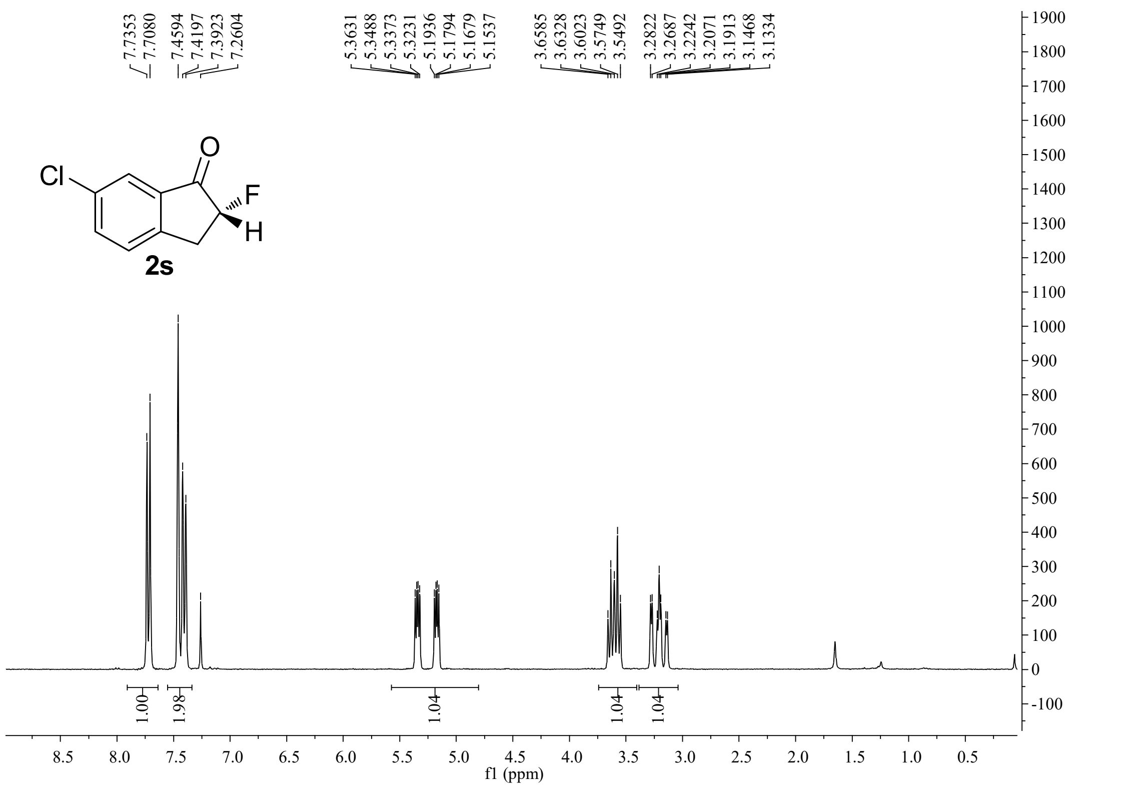
7.7353
7.7080
7.4594
7.4197
7.3923
7.2604
5.3631
5.3488
5.3373
5.3231
5.1936
5.1794
5.1679
5.1537
3.6585
3.6328
3.6023
3.5749
3.5492
3.2822
3.2687
3.2242
3.2071
3.1913
3.1468
3.1334
Cl
O
F
H
2s
1.00
1.98
1.04
1.04
1.04
f1 (ppm)

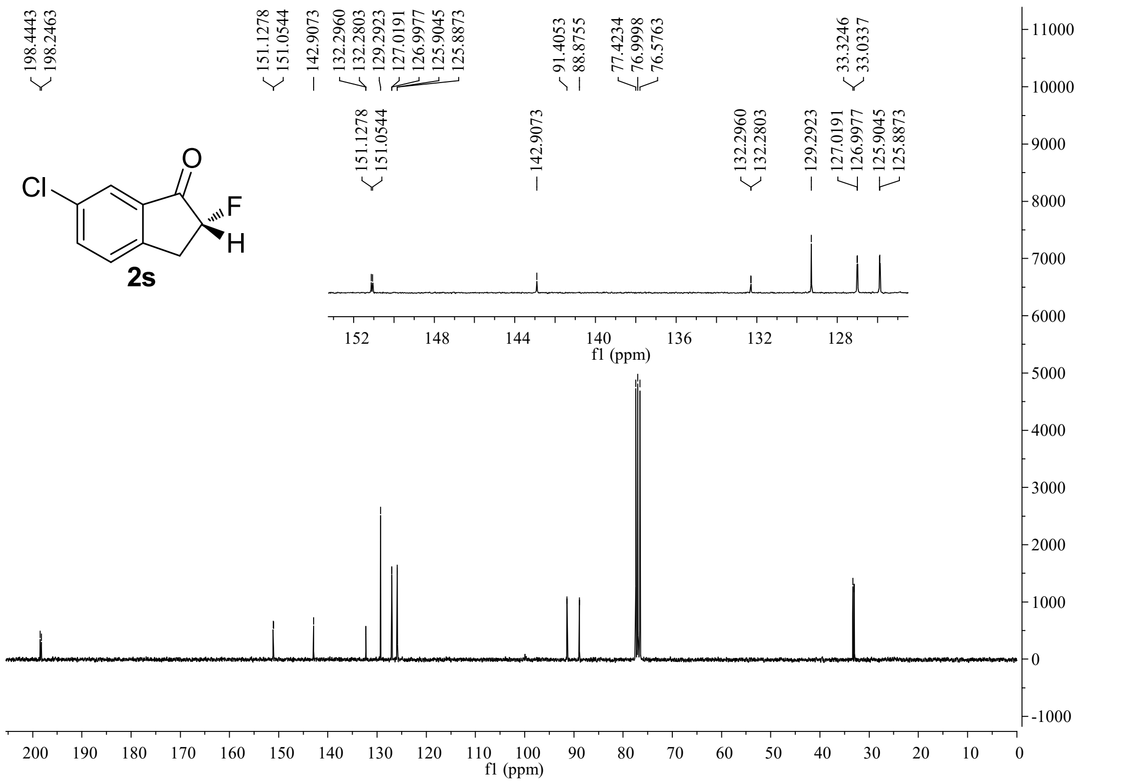
198.4443
198.2463
151.1278
151.0544
142.9073
132.2960
132.2803
129.2923
127.0191
126.9977
125.9045
125.8873
91.4053
88.8755
77.4234
76.9998
76.5763
33.3246
33.0337
Cl
O
F
H
2s
f1 (ppm)
f1 (ppm)

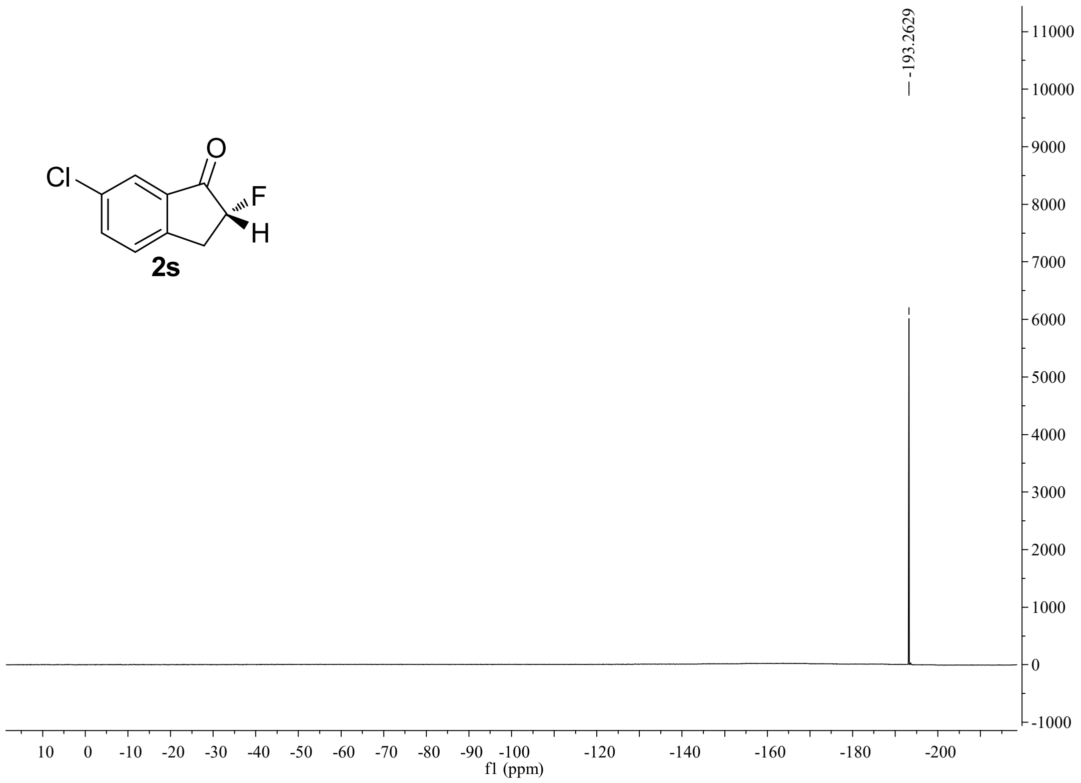

O
Cl
F
H
2s
-193.2629
fl (ppm)

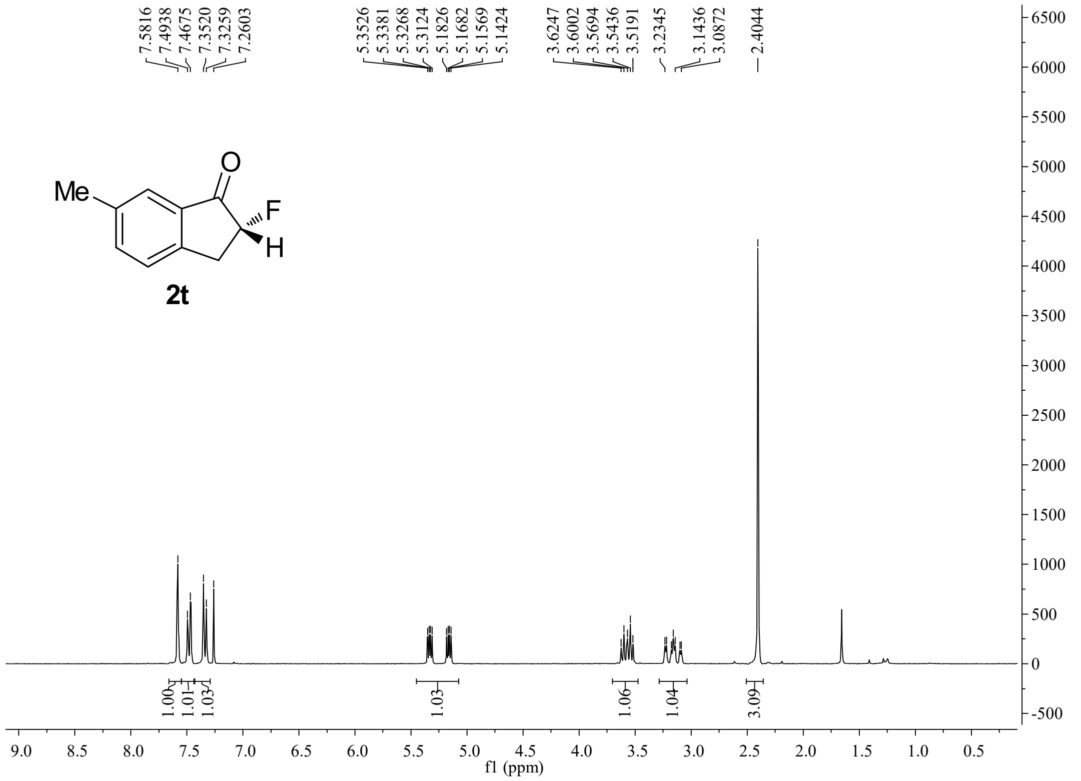

O
Me
F
H
2t
7.5816
7.4938
7.4675
7.3520
7.3259
7.2603
5.3526
5.3381
5.3268
5.3124
5.1826
5.1682
5.1569
5.1424
3.6247
3.6002
3.5694
3.5436
3.5191
3.2345
3.1436
3.0872
2.4044
1.00
1.01
1.03
1.03
1.06
1.04
3.09
fl (ppm)

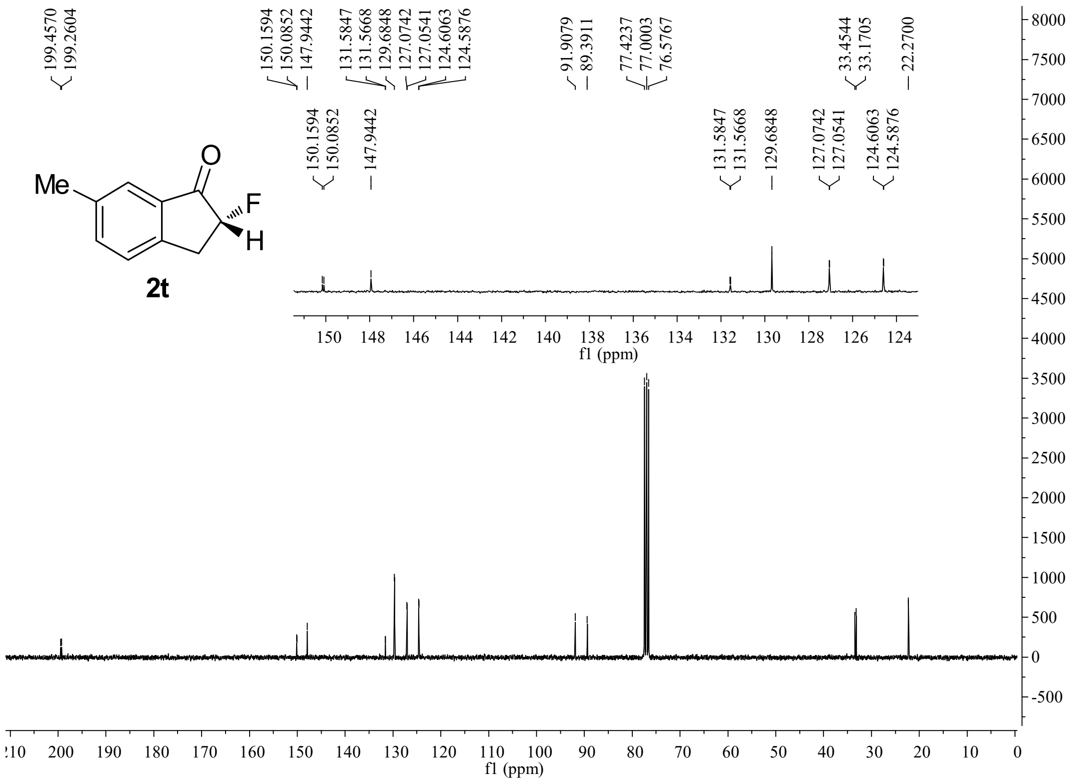
199.4570
199.2604
150.1594
150.0852
147.9442
131.5847
131.5668
129.6848
127.0742
127.0541
124.6063
124.5876
91.9079
89.3911
77.4237
77.0003
76.5767
33.4544
33.1705
22.2700
Me
O
F
H
2t
fl (ppm)
fl (ppm)

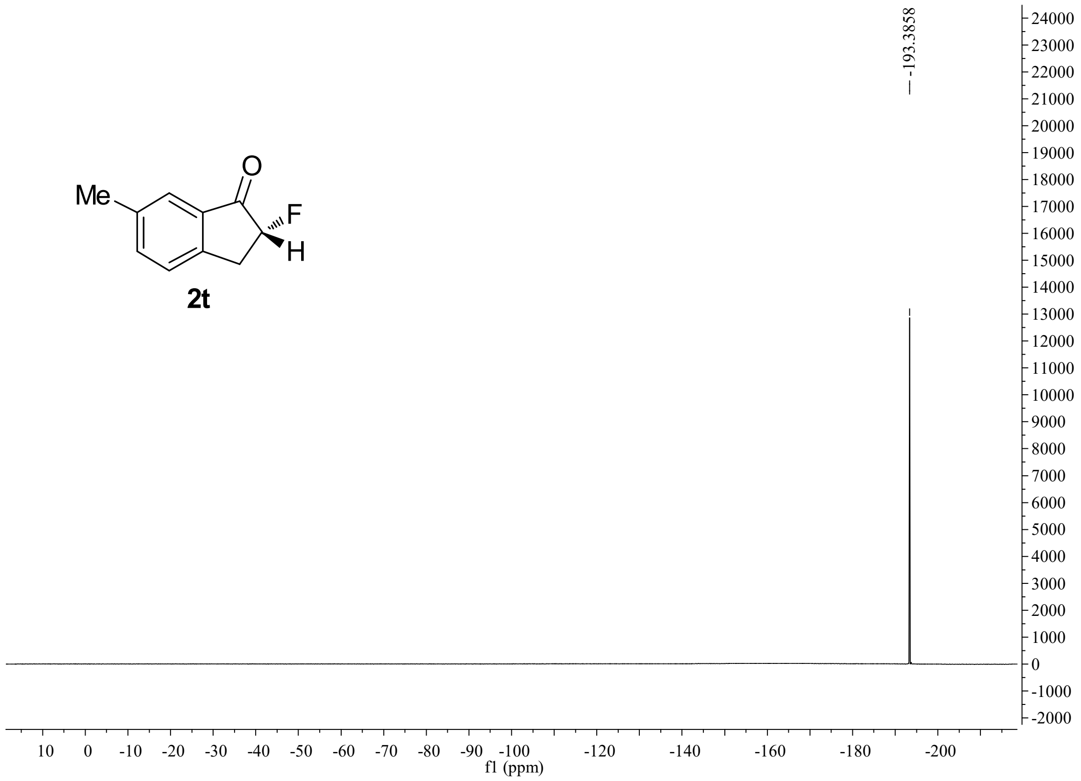
-193.3858
Me
O
F
H
2t
fl (ppm)

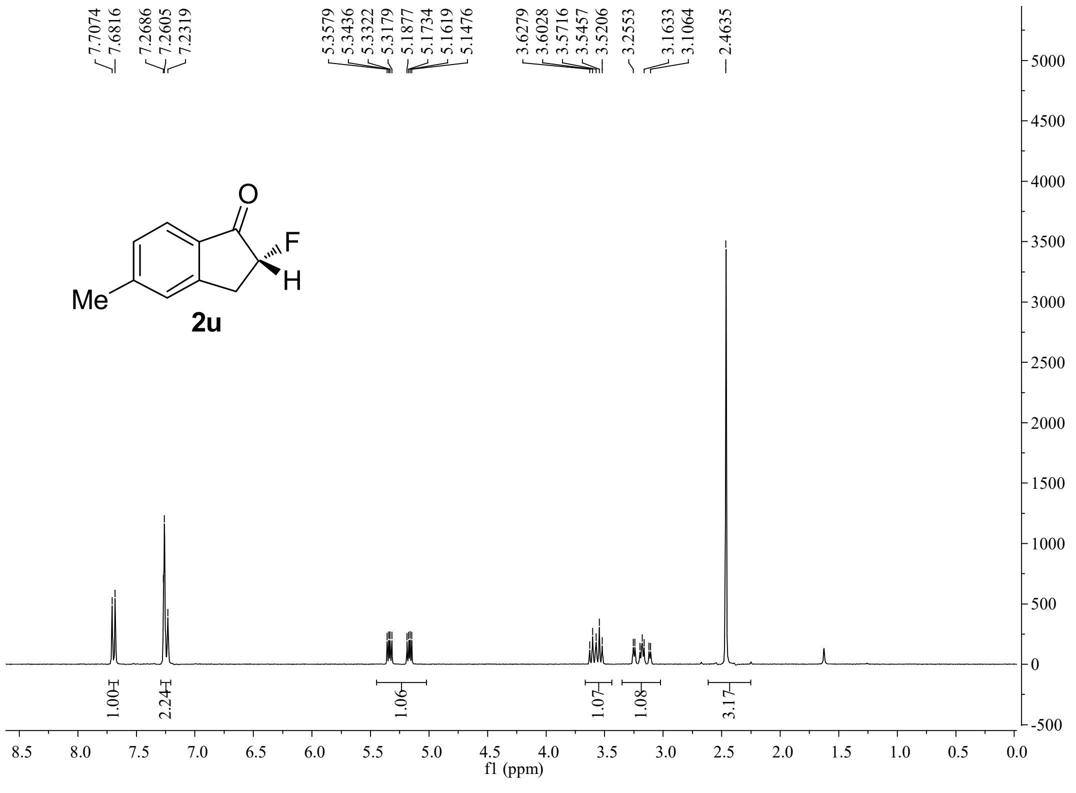
7.7074
7.6816
7.2686
7.2605
7.2319
5.3579
5.3436
5.3322
5.3179
5.1877
5.1734
5.1619
5.1476
3.6279
3.6028
3.5716
3.5457
3.5206
3.2553
3.1633
3.1064
2.4635
O
F
H
Me
2u
1.00
2.24
1.06
1.07
1.08
3.17
f1 (ppm)

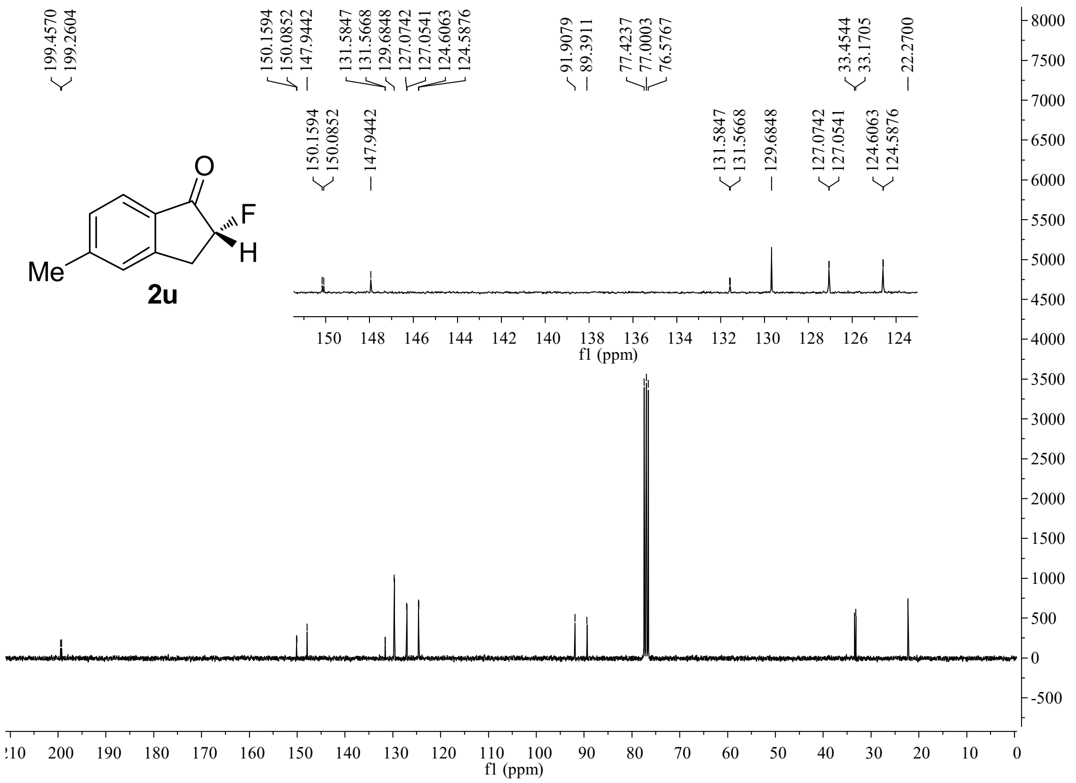
199.4570
199.2604
150.1594
150.0852
147.9442
131.5847
131.5668
129.6848
127.0742
127.0541
124.6063
124.5876
91.9079
89.3911
77.4237
77.0003
76.5767
33.4544
33.1705
22.2700
O
F
H
Me
2u
f1 (ppm)

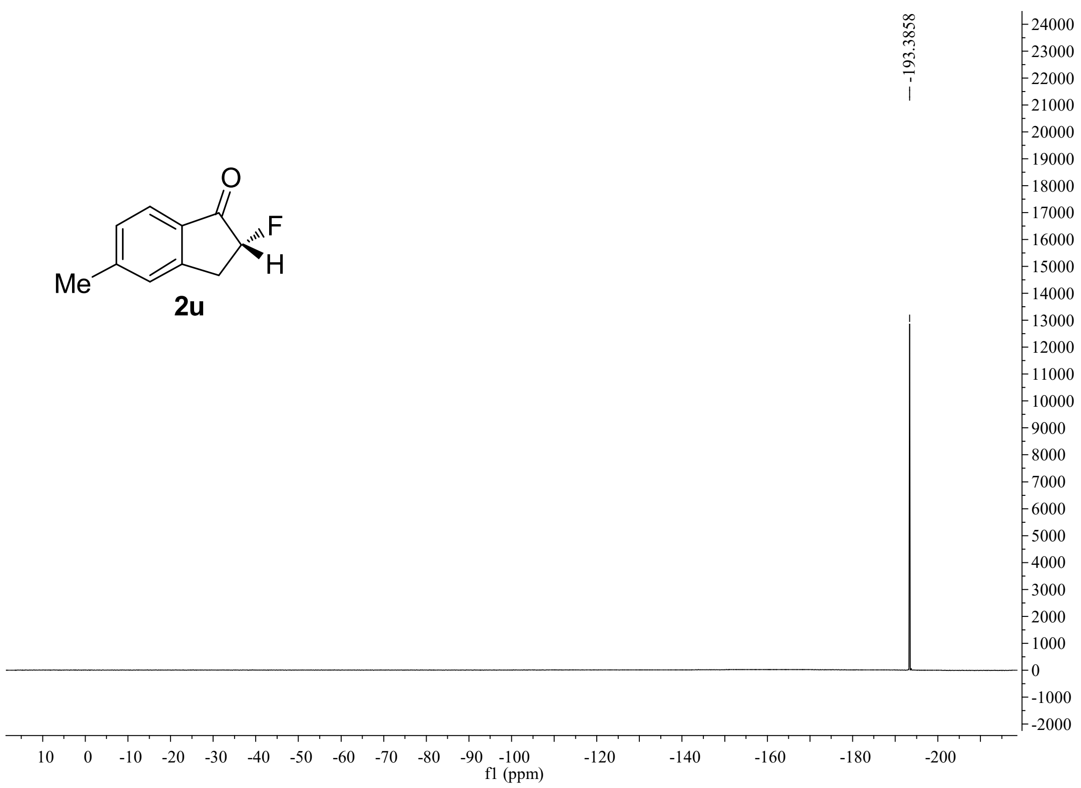
-193.3858
2u
Me
O
F
H
fl (ppm)

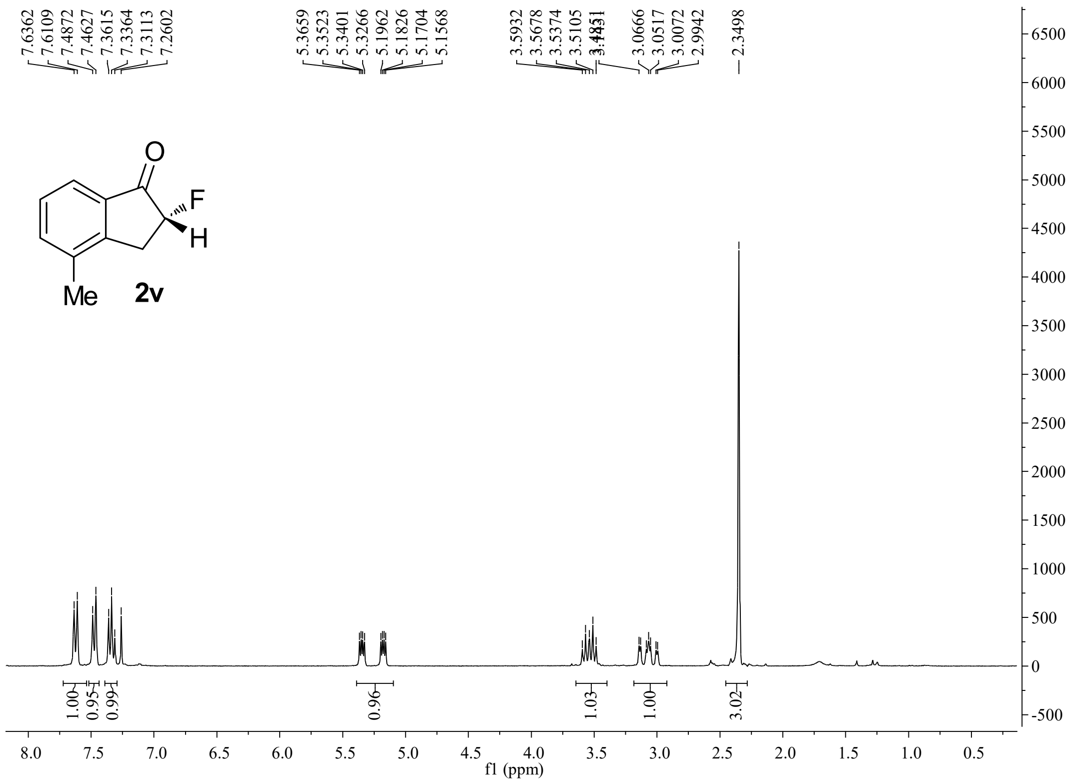
7.6362
7.6109
7.4872
7.4627
7.3615
7.3364
7.3113
7.2602
5.3659
5.3523
5.3401
5.3266
5.1962
5.1826
5.1704
5.1568
3.5932
3.5678
3.5374
3.5105
3.0666
3.0517
3.0072
2.9942
2.3498
2v
Me
O
F
H
1.00
0.95
0.99
0.96
1.03
1.00
3.02
fl (ppm)

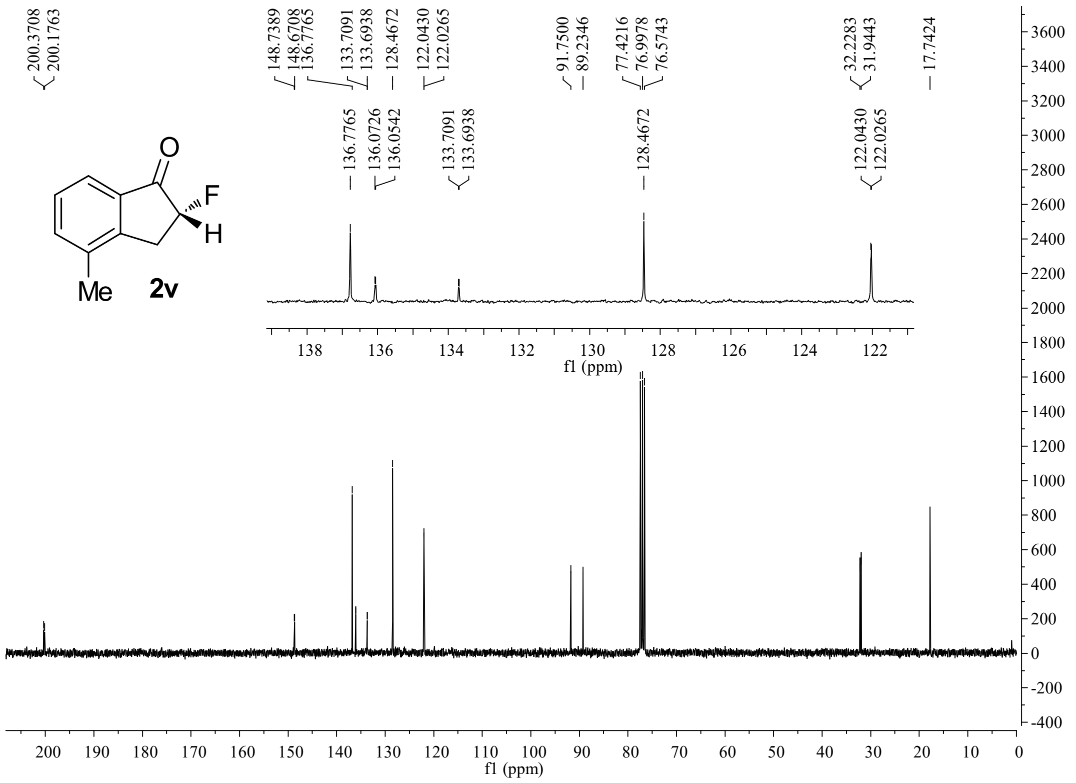
200.3708
200.1763
148.7389
148.6708
136.7765
133.7091
133.6938
128.4672
122.0430
122.0265
91.7500
89.2346
77.4216
76.9978
76.5743
32.2283
31.9443
17.7424
136.7765
136.0726
136.0542
133.7091
133.6938
128.4672
122.0430
122.0265
O
F
H
Me
2v
f1 (ppm)

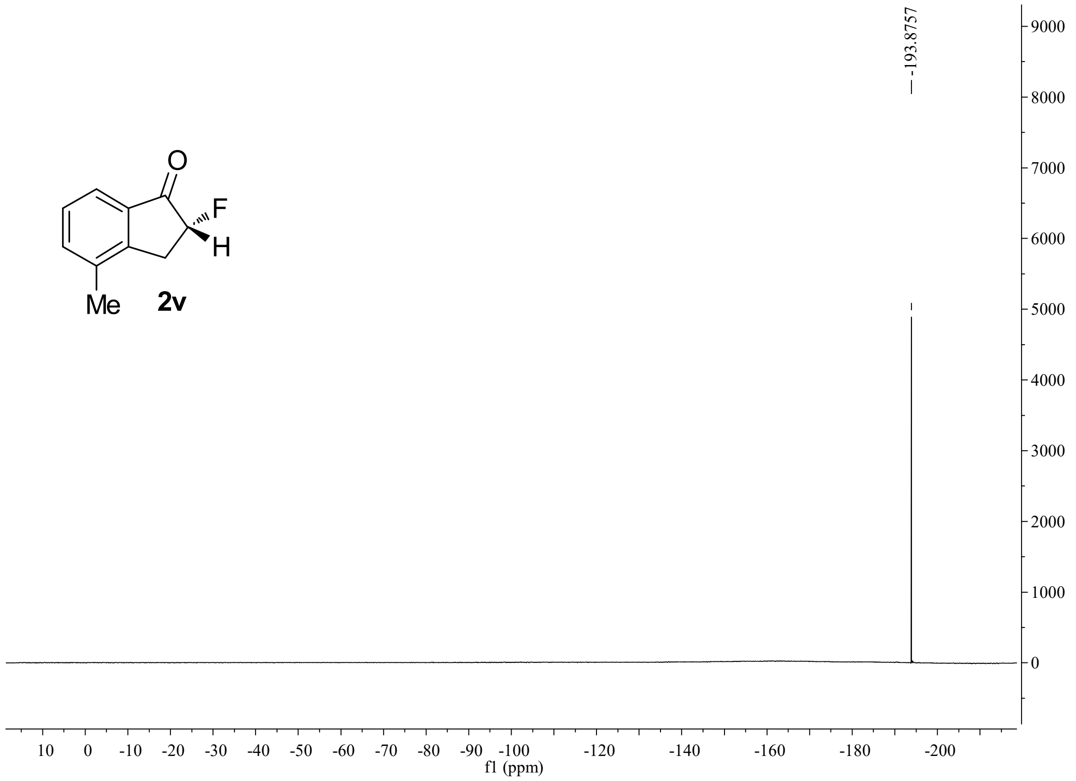
-193.8757
O
F
H
Me
2v
f1 (ppm)

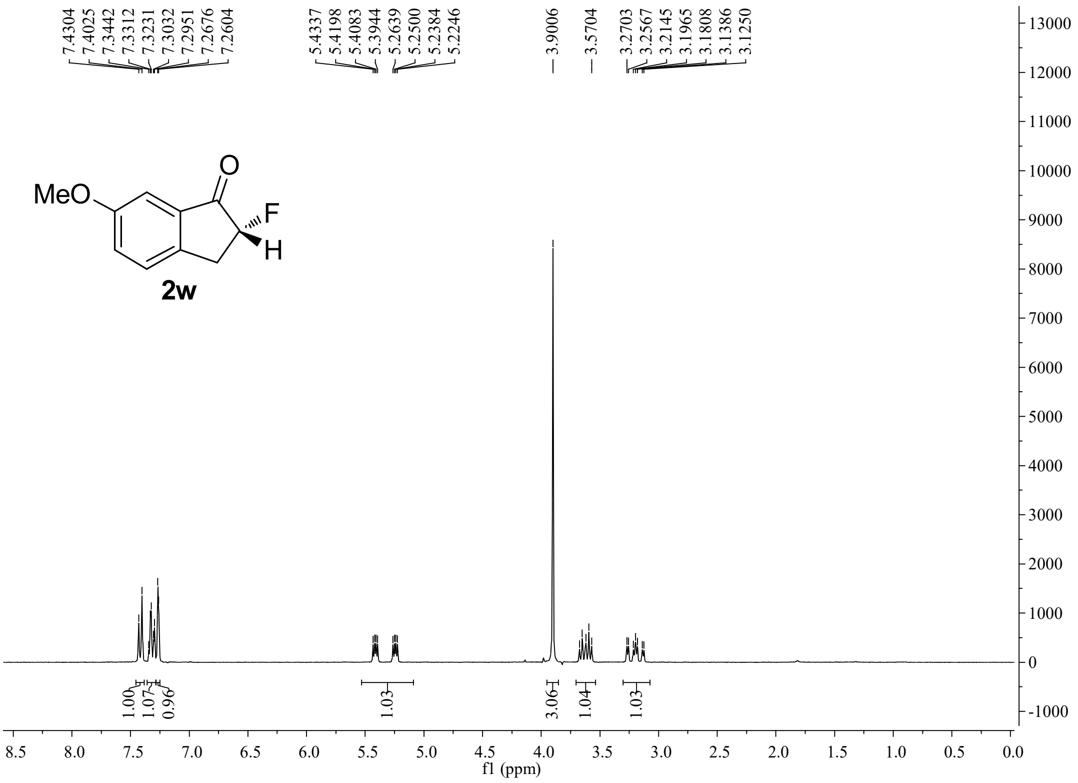
7.4304
7.4025
7.3442
7.3312
7.3231
7.3032
7.2951
7.2676
7.2604
5.4337
5.4198
5.4083
5.3944
5.2639
5.2500
5.2384
5.2246
3.9006
3.5704
3.2703
3.2567
3.2145
3.1965
3.1808
3.1386
3.1250
MeO
O
F
H
2w
1.00
1.07
0.96
1.03
3.06
1.04
1.03
f1 (ppm)

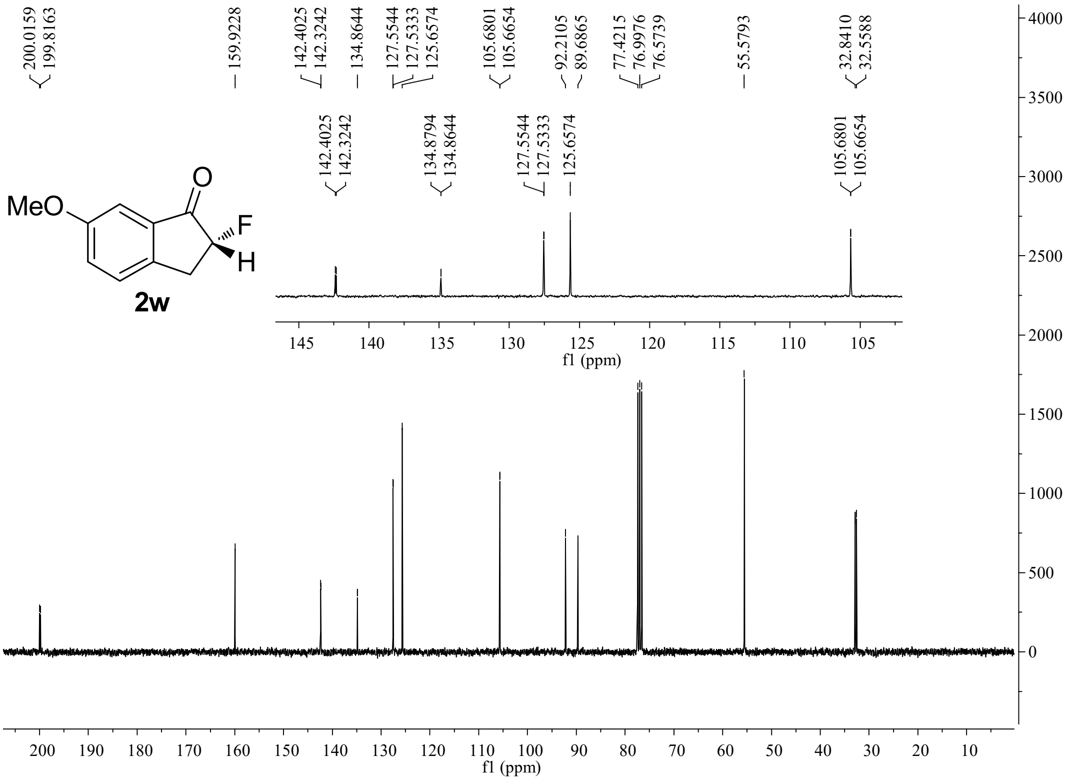
200.0159
199.8163
159.9228
142.4025
142.3242
134.8644
127.5544
127.5333
125.6574
105.6801
105.6654
92.2105
89.6865
77.4215
76.9976
76.5739
55.5793
32.8410
32.5588
134.8794
MeO
O
F
H
2w
f1 (ppm)

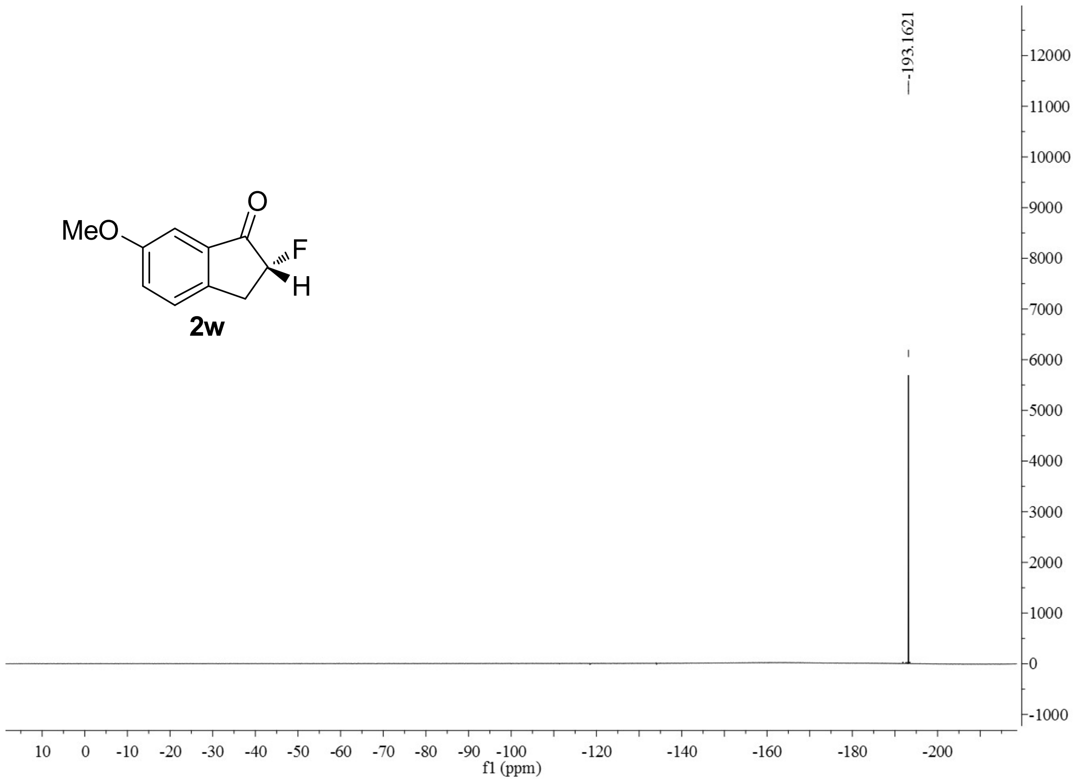
-193.1621
MeO
O
F
H
2w
f1 (ppm)

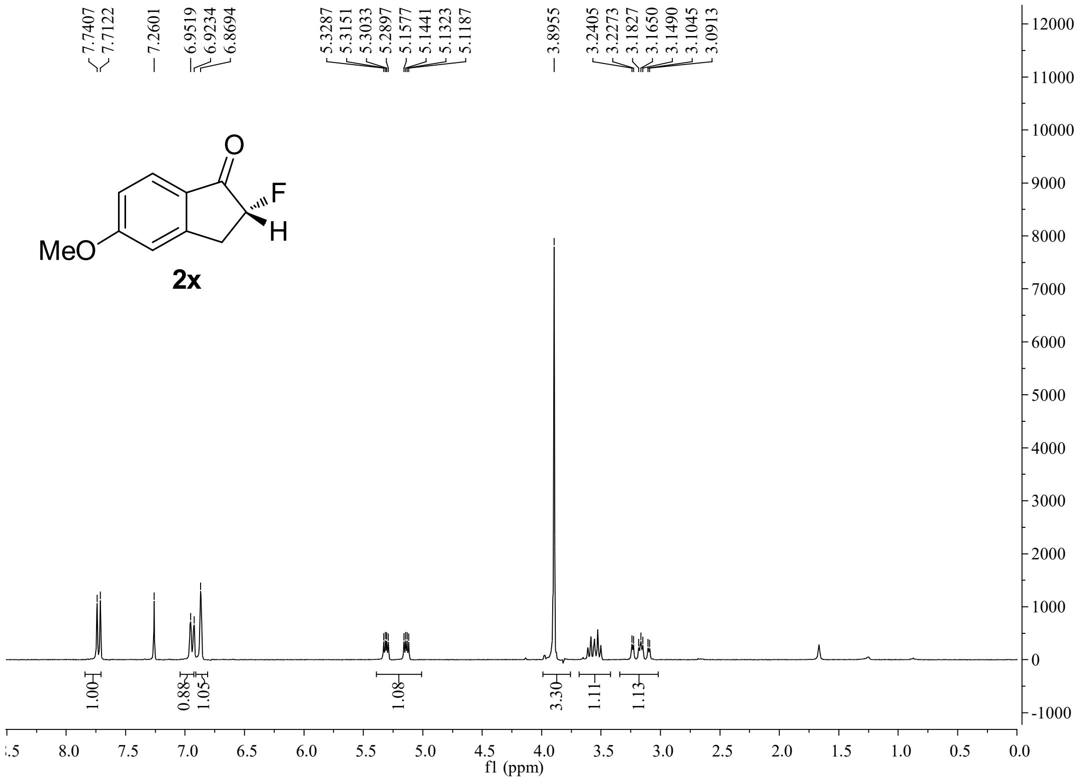
7.7407
7.7122
7.2601
6.9519
6.9234
6.8694
5.3287
5.3151
5.3033
5.2897
5.1577
5.1441
5.1323
5.1187
3.8955
3.2405
3.2273
3.1827
3.1650
3.1490
3.1045
3.0913
O
F
H
MeO
2x
1.00
0.88
1.05
1.08
3.30
1.11
1.13
f1 (ppm)

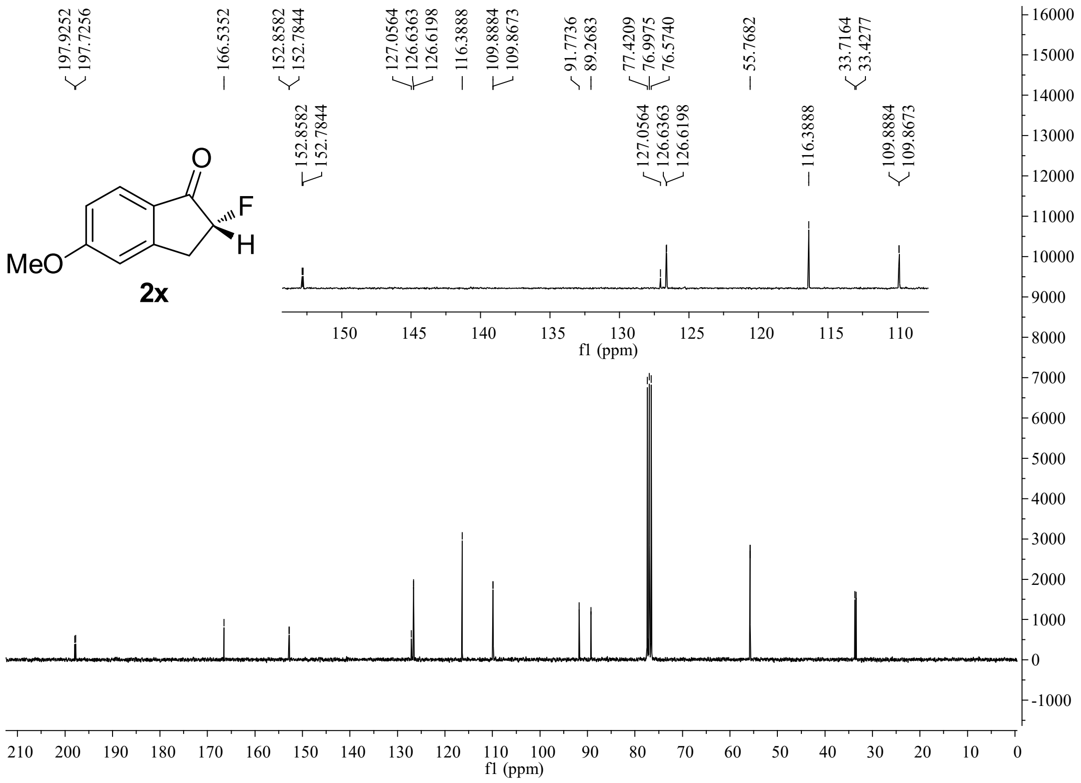
197.9252
197.7256
166.5352
152.8582
152.7844
127.0564
126.6363
126.6198
116.3888
109.8884
109.8673
91.7736
89.2683
77.4209
76.9975
76.5740
55.7682
33.7164
33.4277
MeO
O
F
H
2x
fl (ppm)

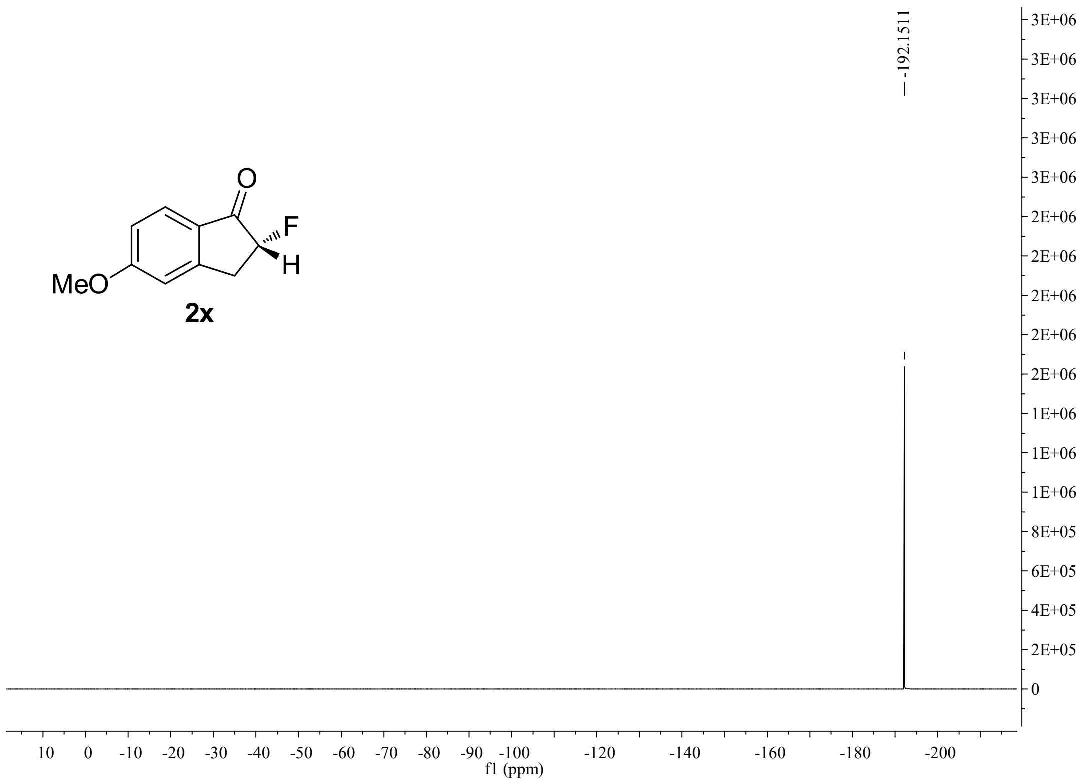
-192.1511
MeO
O
F
H
2x
fl (ppm)

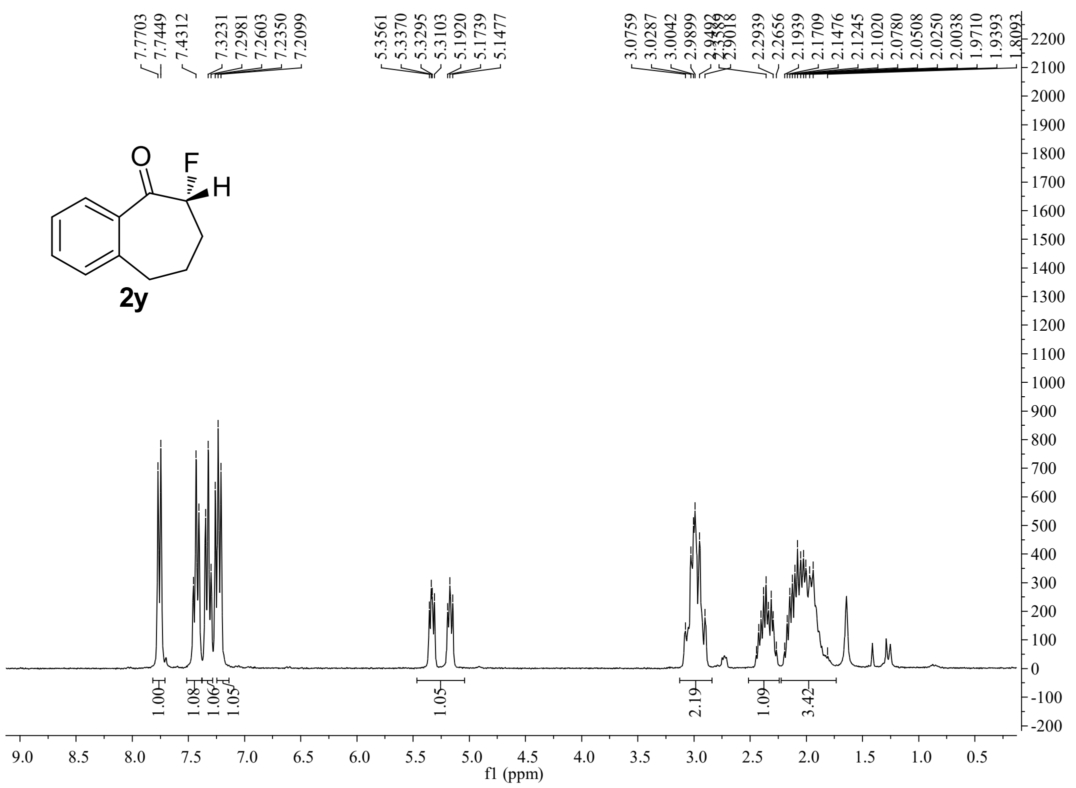
7.7703
7.7449
7.4312
7.3231
7.2981
7.2603
7.2350
7.2099
5.3561
5.3370
5.3295
5.3103
5.1920
5.1739
5.1477
3.0759
3.0287
3.0042
2.9899
2.9018
2.2939
2.2656
2.1939
2.1709
2.1476
2.1245
2.1020
2.0780
2.0508
2.0250
2.0038
1.9710
1.9393
2y
1.00
1.08
1.06
1.05
1.05
2.19
1.09
3.42
f1 (ppm)

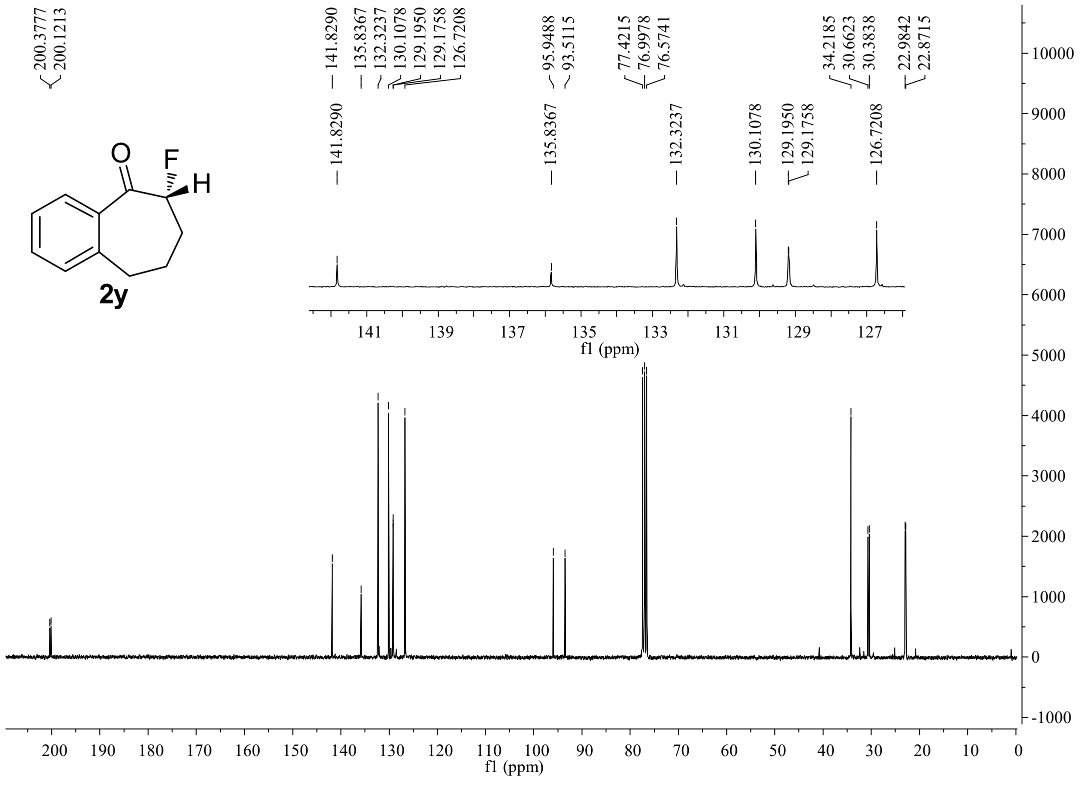
200.3777
200.1213
141.8290
135.8367
132.3237
130.1078
129.1950
129.1758
126.7208
95.9488
93.5115
77.4215
76.9978
76.5741
34.2185
30.6623
30.3838
22.9842
22.8715
2y
f1 (ppm)

2y

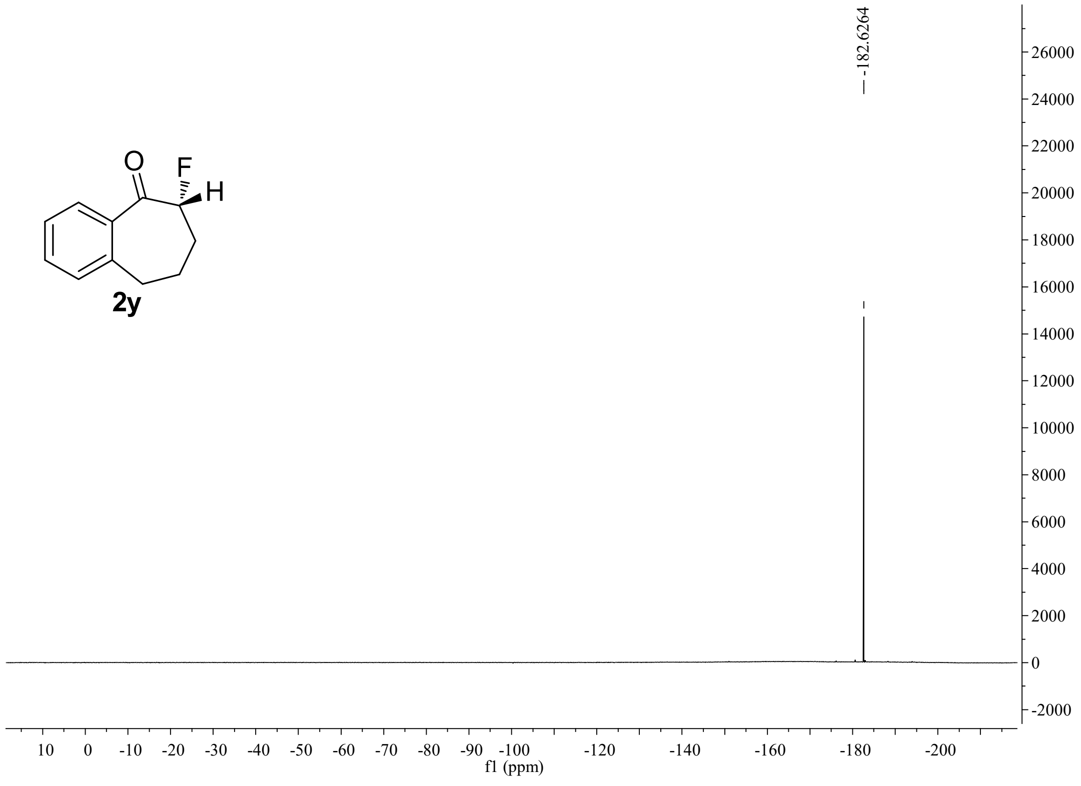
F
H
O
-182.6264
26000
24000
22000
20000
18000
16000
14000
12000
10000
8000
6000
4000
2000
0
-2000
10 0 -10 -20 -30 -40 -50 -60 -70 -80 -90 -100 -120 -140 -160 -180 -200
f1 (ppm)

2za

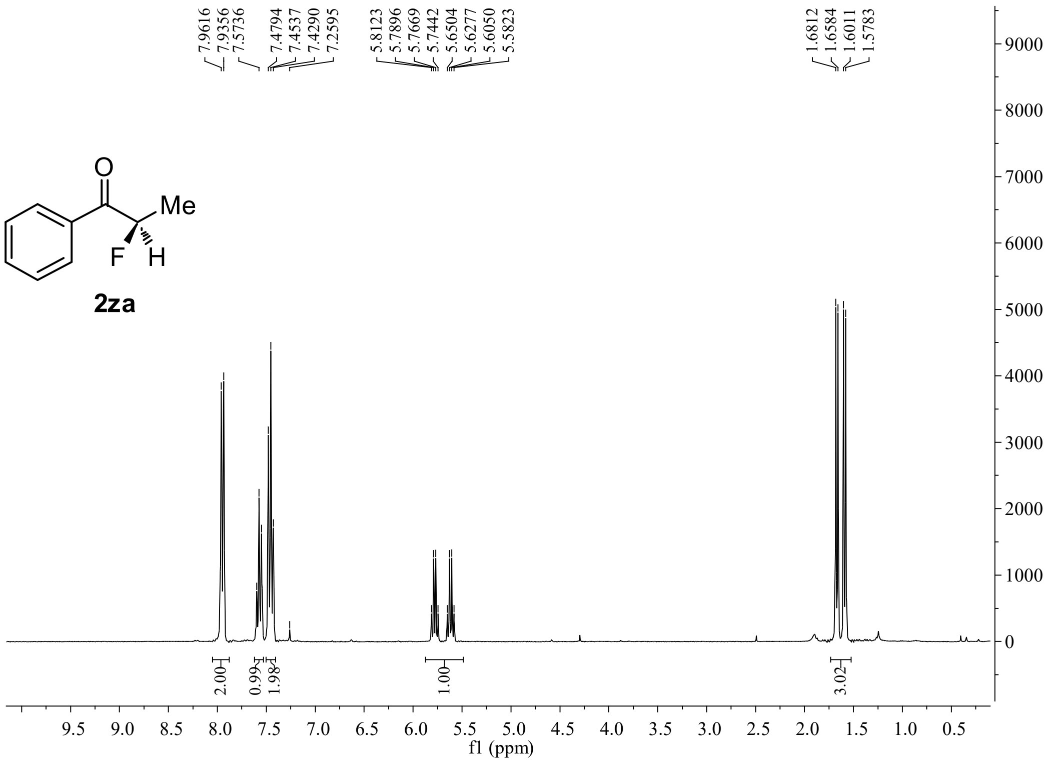
O
Me
F
H
7.9616
7.9356
7.5736
7.4794
7.4537
7.4290
7.2595
5.8123
5.7896
5.7669
5.7442
5.6504
5.6277
5.6050
5.5823
1.6812
1.6584
1.6011
1.5783
9000
8000
7000
6000
5000
4000
3000
2000
1000
0
2.00
0.99
1.98
1.00
3.02
9.5 9.0 8.5 8.0 7.5 7.0 6.5 6.0 5.5 5.0 4.5 4.0 3.5 3.0 2.5 2.0 1.5 1.0 0.5
f1 (ppm)

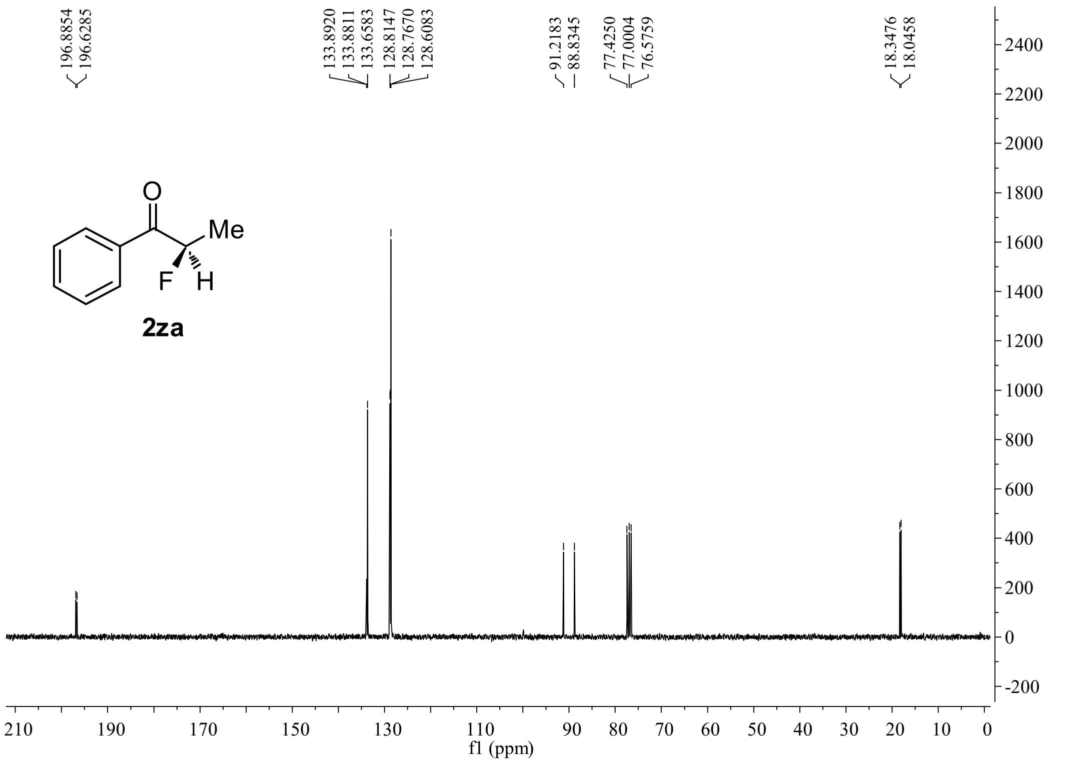
196.8854
196.6285
133.8920
133.8811
133.6583
128.8147
128.7670
128.6083
91.2183
88.8345
77.4250
77.0004
76.5759
18.3476
18.0458
O
Me
F
H
2za
2400
2200
2000
1800
1600
1400
1200
1000
800
600
400
200
0
-200
210
190
170
150
130
110
90
80
70
60
50
40
30
20
10
0
f1 (ppm)

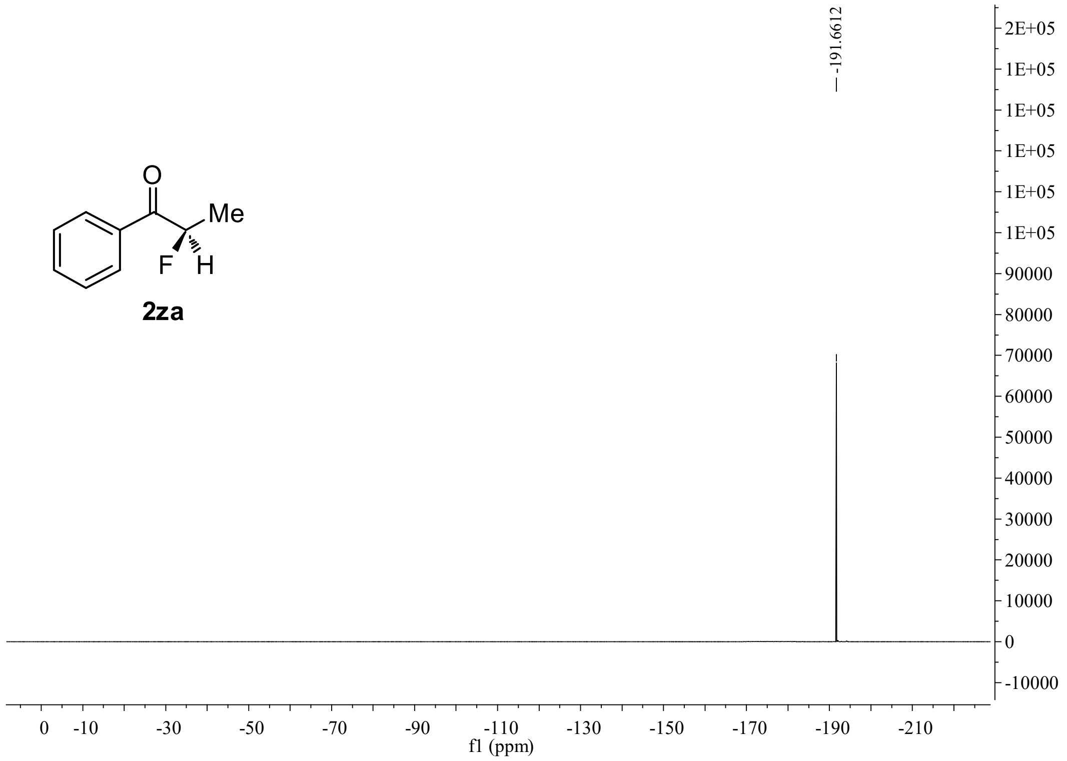
-191.6612
O
Me
F
H
2za
2E+05
1E+05
1E+05
1E+05
1E+05
1E+05
90000
80000
70000
60000
50000
40000
30000
20000
10000
0
-10000
0
-10
-30
-50
-70
-90
-110
-130
-150
-170
-190
-210
f1 (ppm)

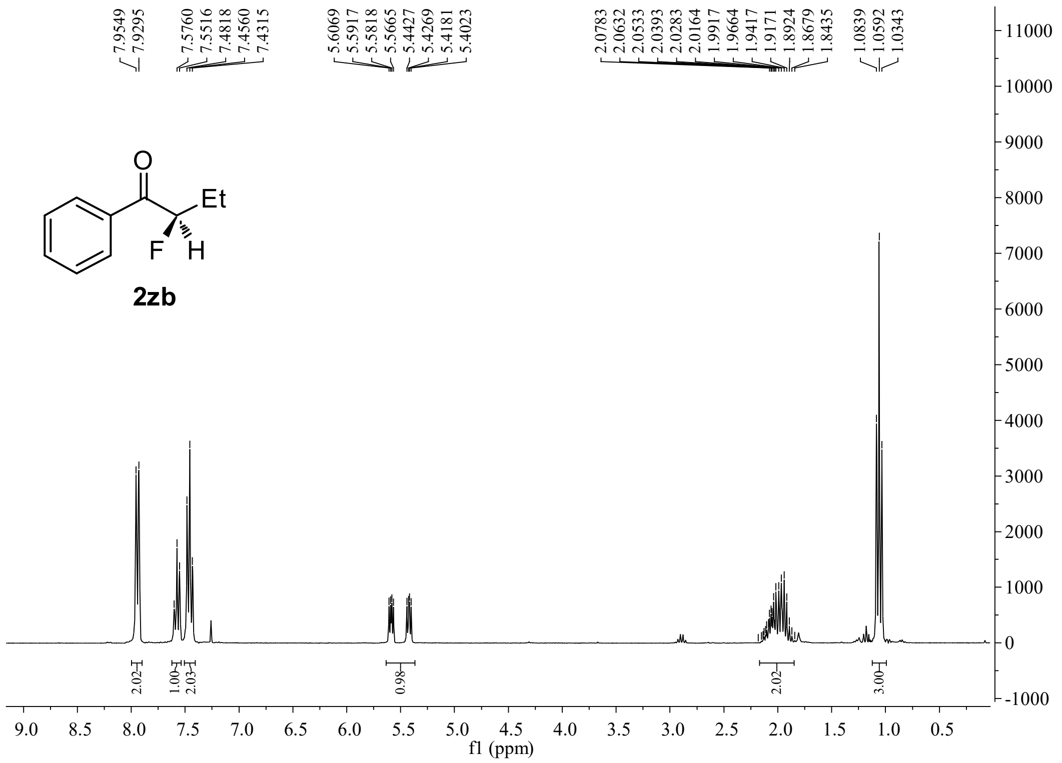
7.9549
7.9295
7.5760
7.5516
7.4818
7.4560
7.4315
5.6069
5.5917
5.5818
5.5665
5.4427
5.4269
5.4181
5.4023
2.0783
2.0632
2.0533
2.0393
2.0283
2.0164
1.9917
1.9664
1.9417
1.9171
1.8924
1.8679
1.8435
1.0839
1.0592
1.0343
O
Et
F
H
2zb
2.02
1.00
2.03
0.98
2.02
3.00
f1 (ppm)

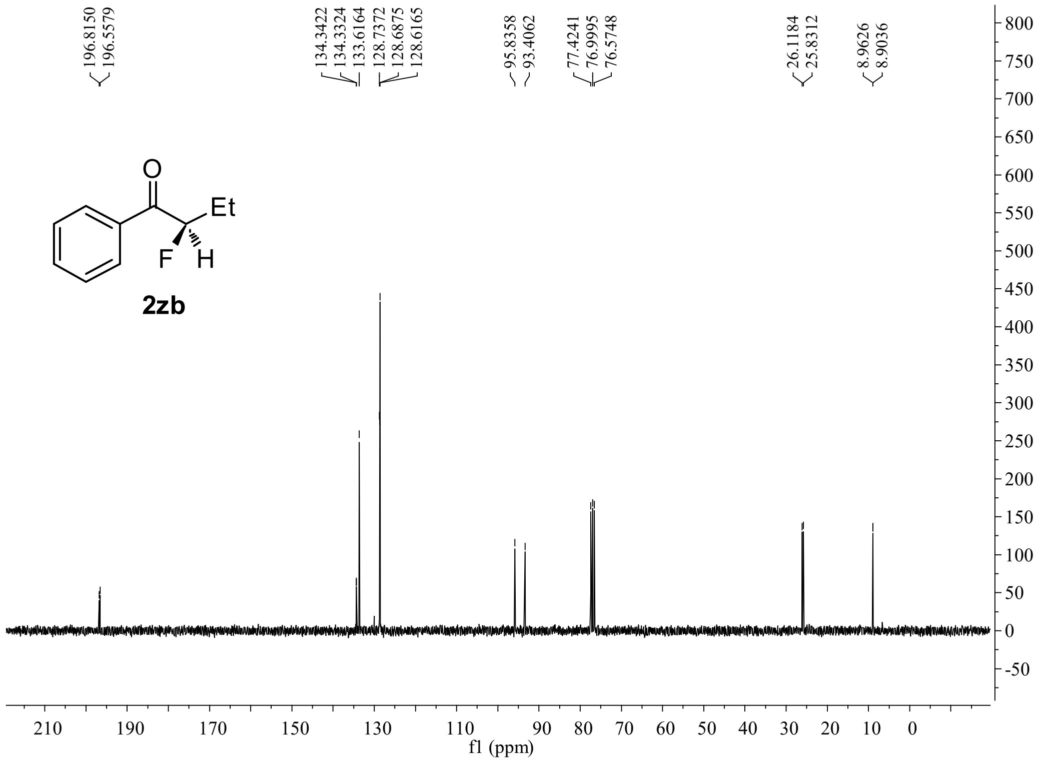
196.8150
196.5579
134.3422
134.3324
133.6164
128.7372
128.6875
128.6165
95.8358
93.4062
77.4241
76.9995
76.5748
26.1184
25.8312
8.9626
8.9036
O
Et
F
H
2zb
f1 (ppm)

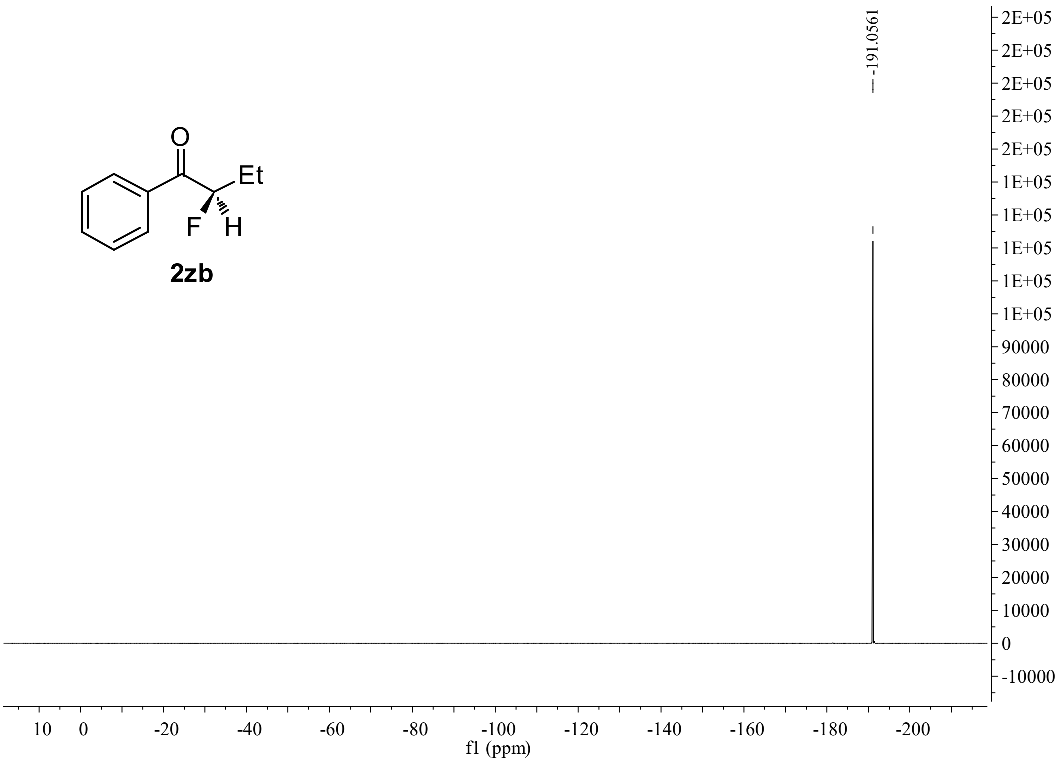
-191.0561
O
Et
F H
2zb
2E+05
1E+05
90000
80000
70000
60000
50000
40000
30000
20000
10000
0
-10000
10 0 -20 -40 -60 -80 -100 -120 -140 -160 -180 -200
f1 (ppm)

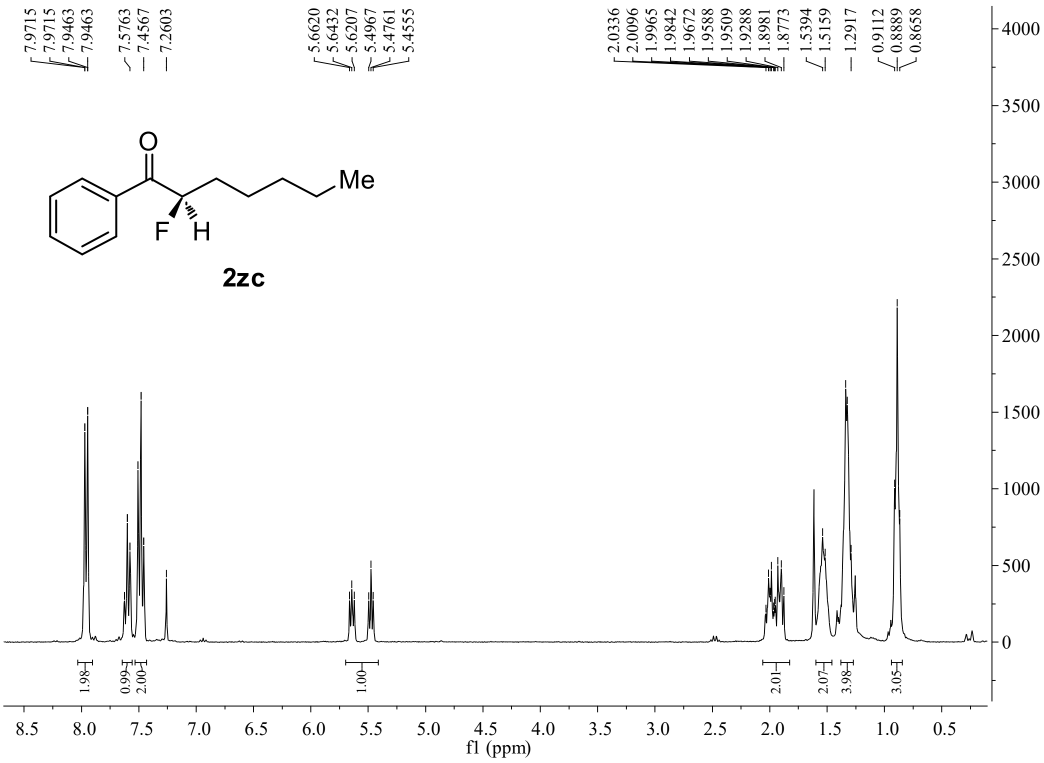
7.9715
7.9715
7.9463
7.9463
7.5763
7.4567
7.2603
5.6620
5.6432
5.6207
5.4967
5.4761
5.4555
2.0336
2.0096
1.9965
1.9842
1.9672
1.9588
1.9509
1.9288
1.8981
1.8773
1.5394
1.5159
1.2917
0.9112
0.8889
0.8658
O
Me
F H
2zc
4000
3500
3000
2500
2000
1500
1000
500
0
1.98
0.99
2.00
1.00
2.01
2.07
3.98
3.05
8.5 8.0 7.5 7.0 6.5 6.0 5.5 5.0 4.5 4.0 3.5 3.0 2.5 2.0 1.5 1.0 0.5
f1 (ppm)

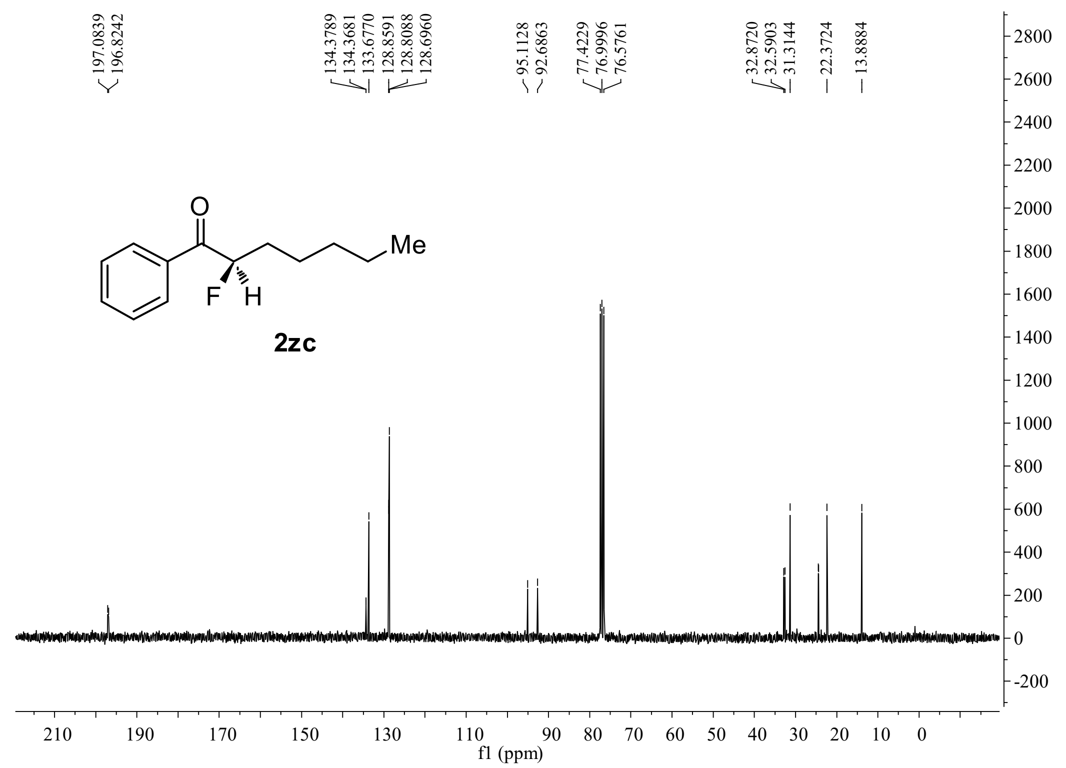
197.0839
196.8242
134.3789
134.3681
133.6770
128.8591
128.8088
128.6960
95.1128
92.6863
77.4229
76.9996
76.5761
32.8720
32.5903
31.3144
22.3724
13.8884
O
Me
F
H
2zc
f1 (ppm)

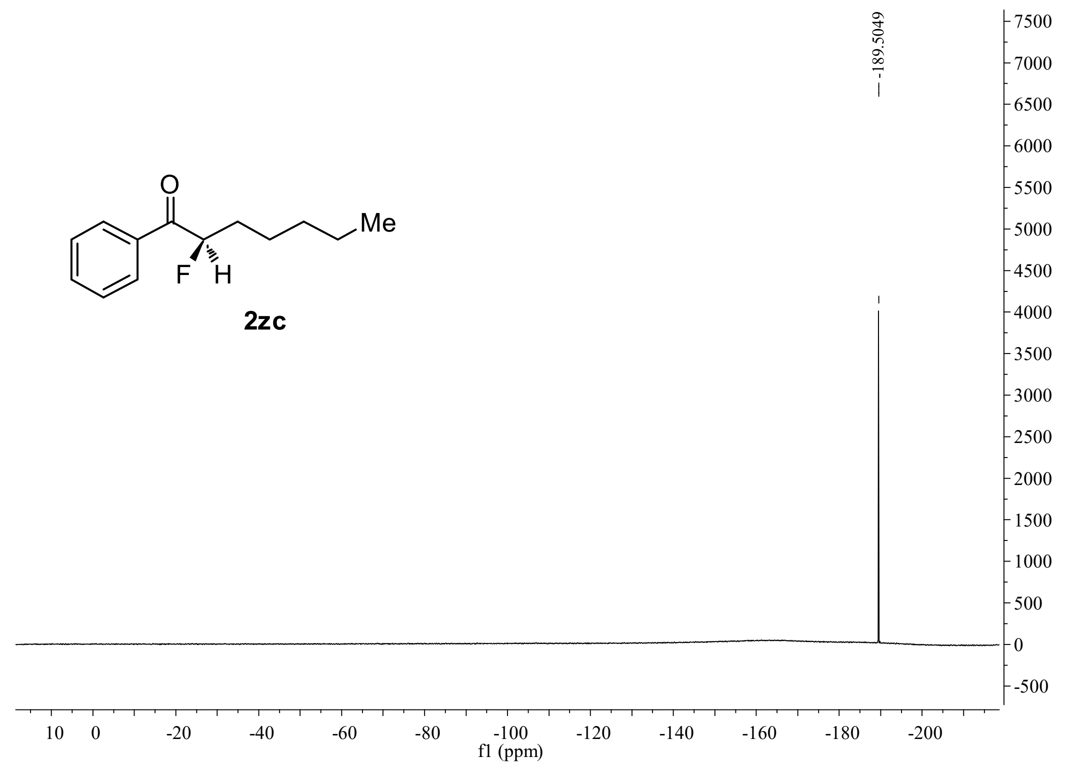
-189.5049
O
Me
F
H
2zc
f1 (ppm)

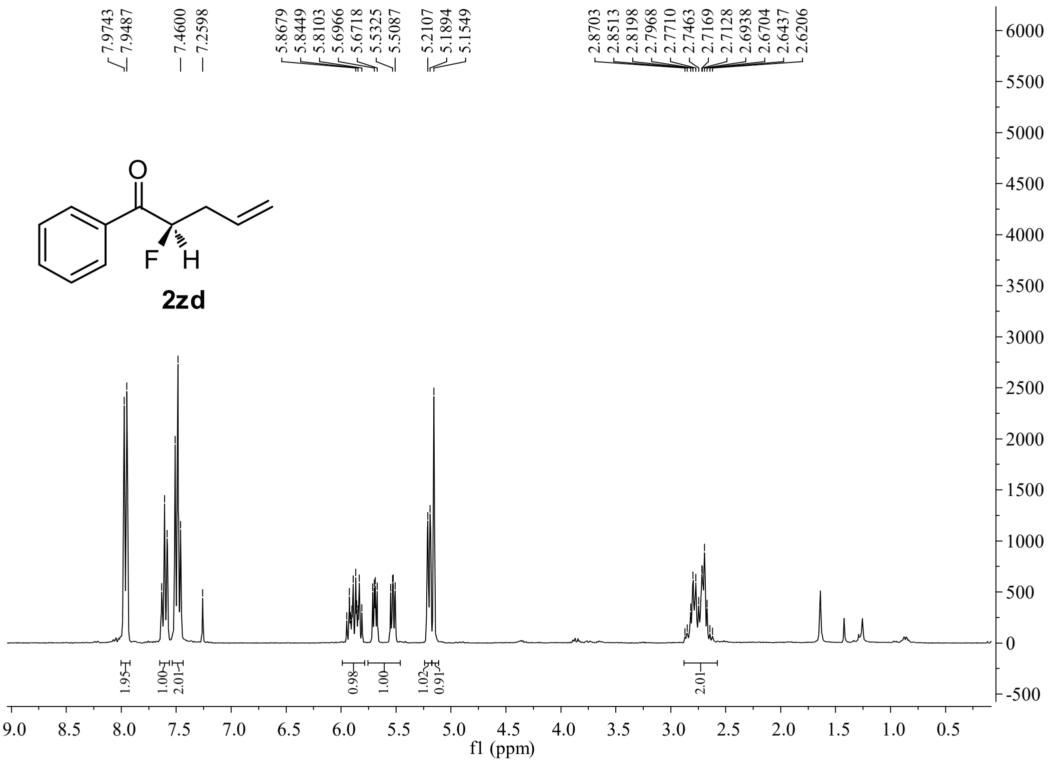
7.9743
7.9487
7.4600
7.2598
5.8679
5.8449
5.8103
5.6966
5.6718
5.5325
5.5087
5.2107
5.1894
5.1549
2.8703
2.8513
2.8198
2.7968
2.7710
2.7463
2.7169
2.7128
2.6938
2.6704
2.6437
2.6206
O
F
H
2zd
1.95
1.00
2.01
0.98
1.00
1.02
0.91
2.01
f1 (ppm)

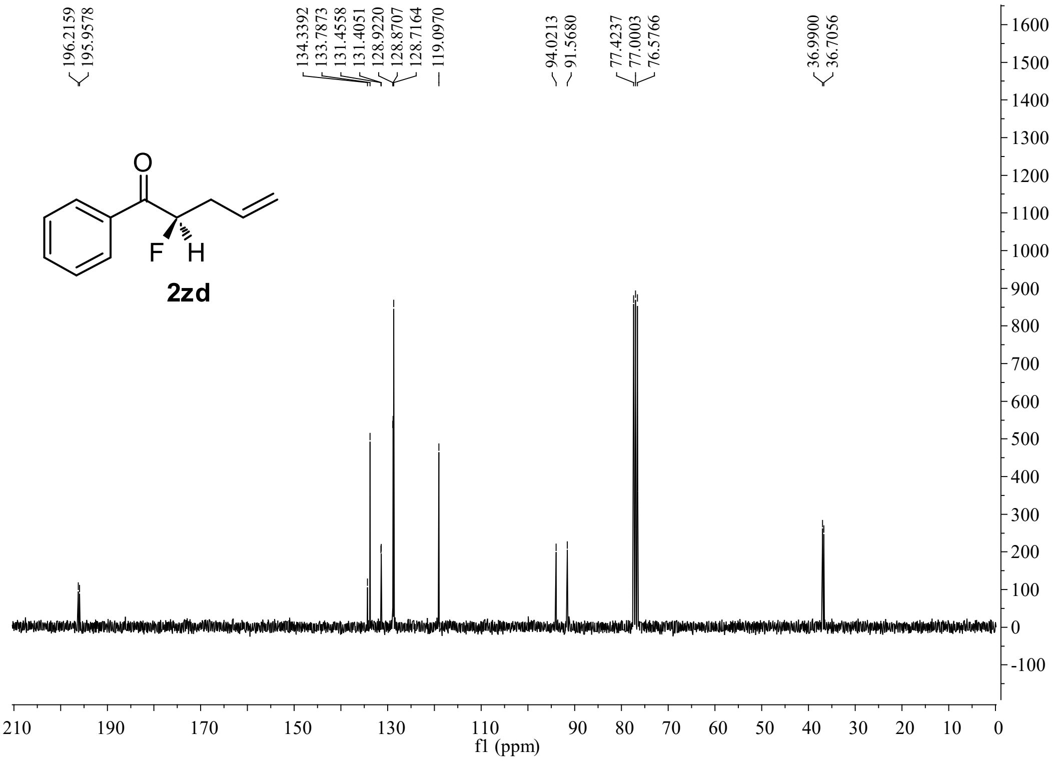
196.2159
195.9578
134.3392
133.7873
131.4558
131.4051
128.9220
128.8707
128.7164
119.0970
94.0213
91.5680
77.4237
77.0003
76.5766
36.9900
36.7056
O
F
H
2zd
f1 (ppm)

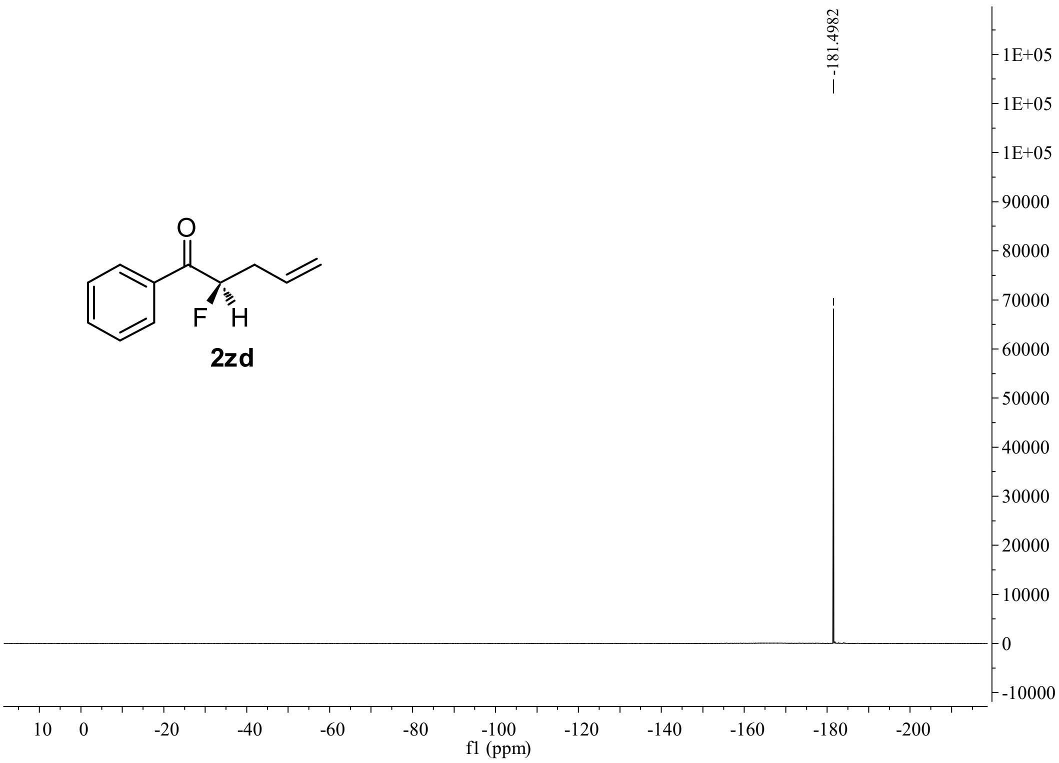
-181.4982
2zd
O
F
H
f1 (ppm)

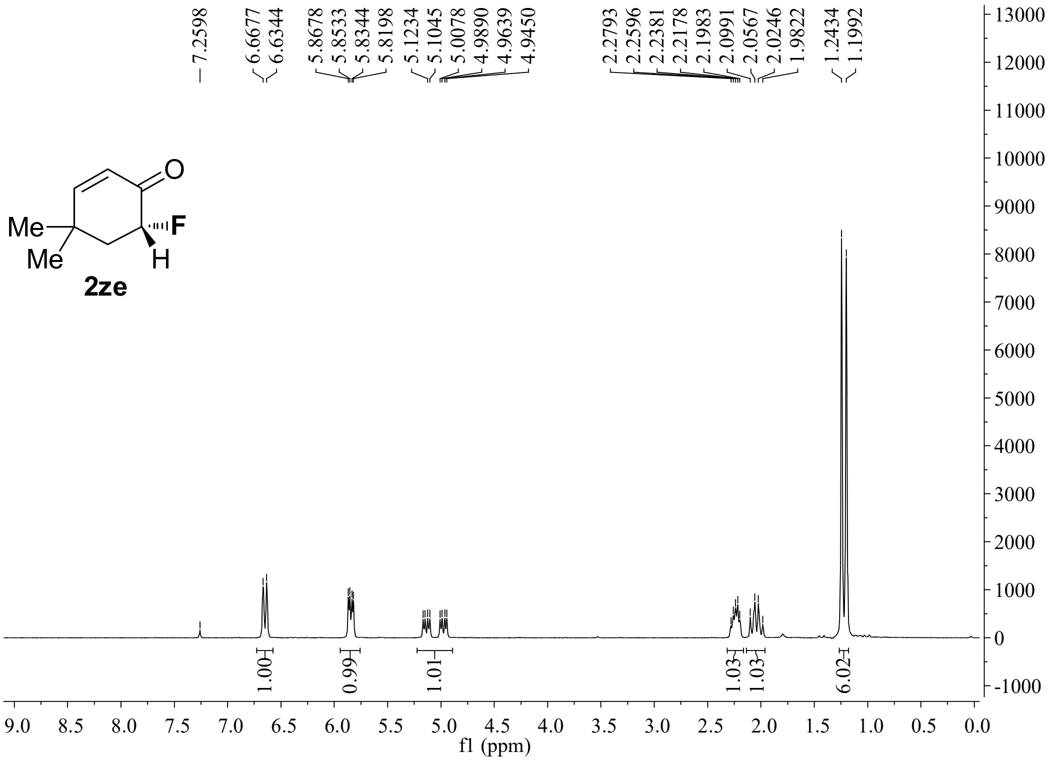
7.2598
6.6677
6.6344
5.8678
5.8533
5.8344
5.8198
5.1234
5.1045
5.0078
4.9890
4.9639
4.9450
2.2793
2.2596
2.2381
2.2178
2.1983
2.0991
2.0567
2.0246
1.9822
1.2434
1.1992
2ze
O
F
H
Me
Me
1.00
0.99
1.01
1.03
1.03
6.02
f1 (ppm)

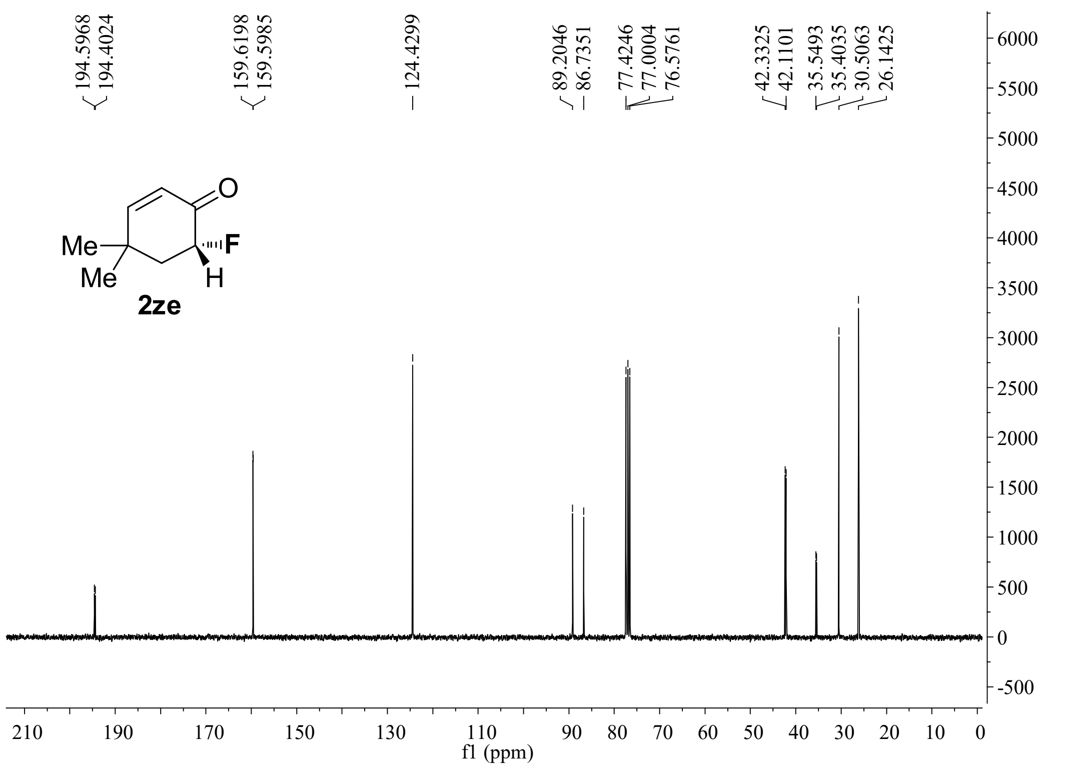
194.5968
194.4024
159.6198
159.5985
124.4299
89.2046
86.7351
77.4246
77.0004
76.5761
42.3325
42.1101
35.5493
35.4035
30.5063
26.1425
Me
Me
O
F
H
2ze
f1 (ppm)

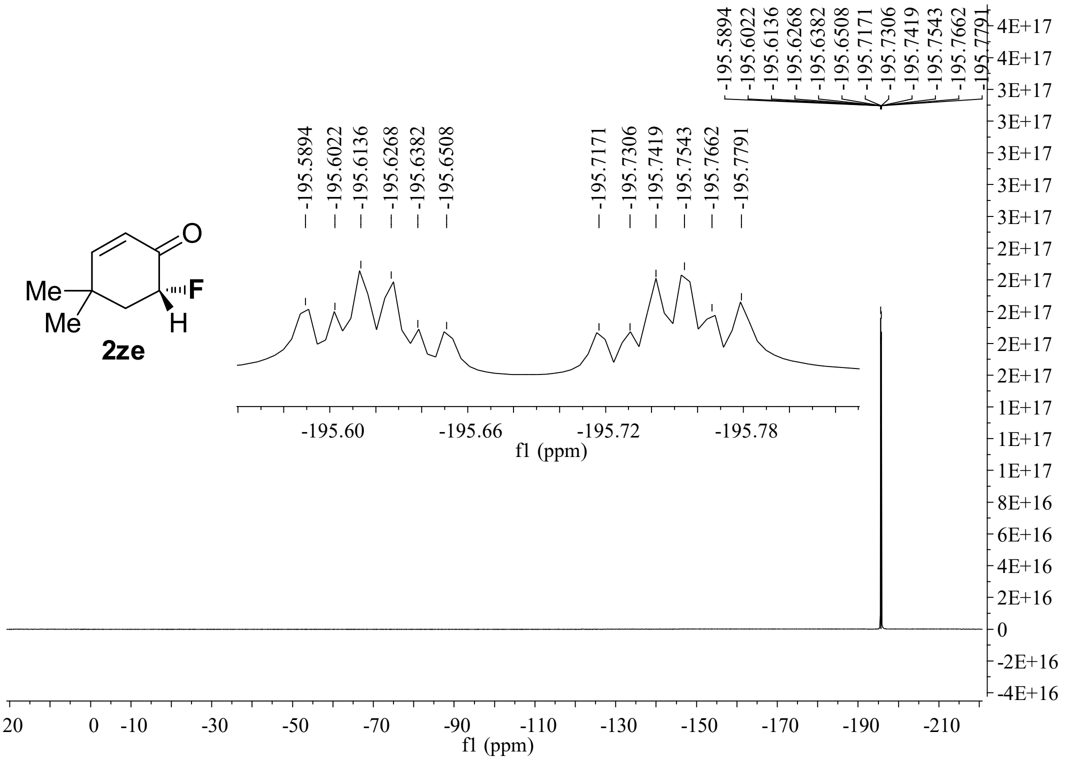
-195.5894
-195.6022
-195.6136
-195.6268
-195.6382
-195.6508
-195.7171
-195.7306
-195.7419
-195.7543
-195.7662
-195.7791
Me
Me
O
F
H
2ze
f1 (ppm)

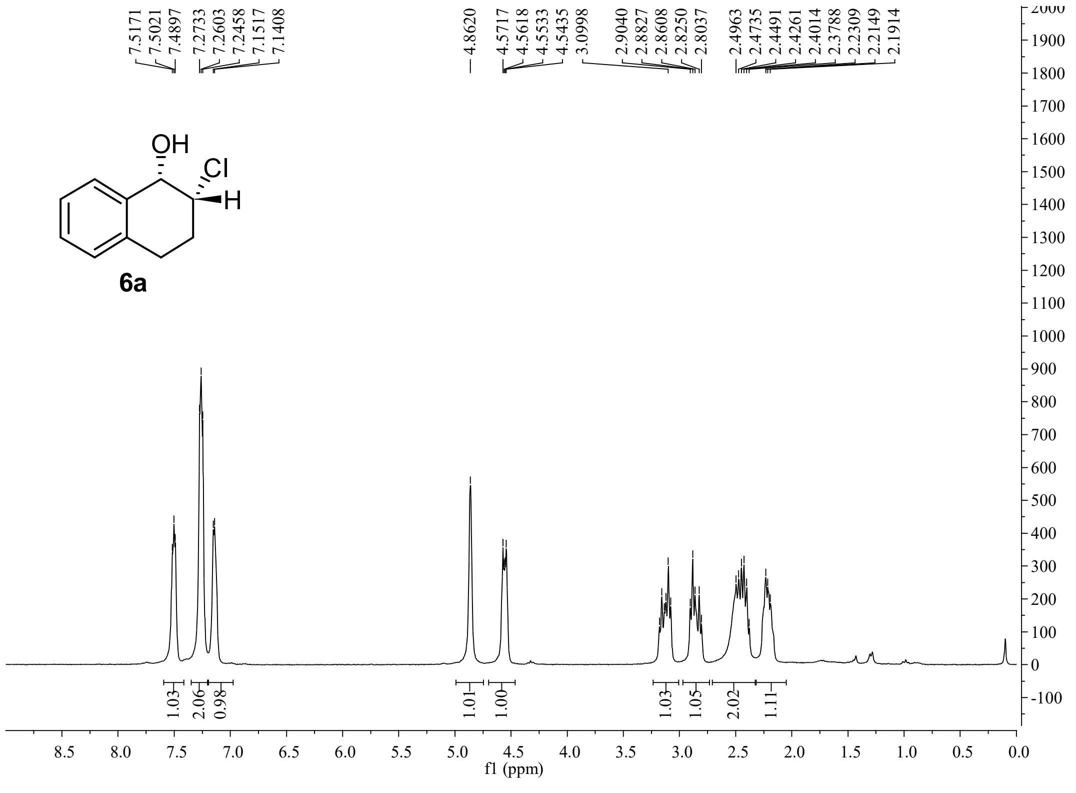
7.5171
7.5021
7.4897
7.2733
7.2603
7.2458
7.1517
7.1408
4.8620
4.5717
4.5618
4.5533
4.5435
3.0998
2.9040
2.8827
2.8608
2.8250
2.8037
2.4963
2.4735
2.4491
2.4261
2.4014
2.3788
2.2309
2.2149
2.1914
OH
Cl
H
6a
1.03
2.06
0.98
1.01
1.00
1.03
1.05
2.02
1.11
f1 (ppm)

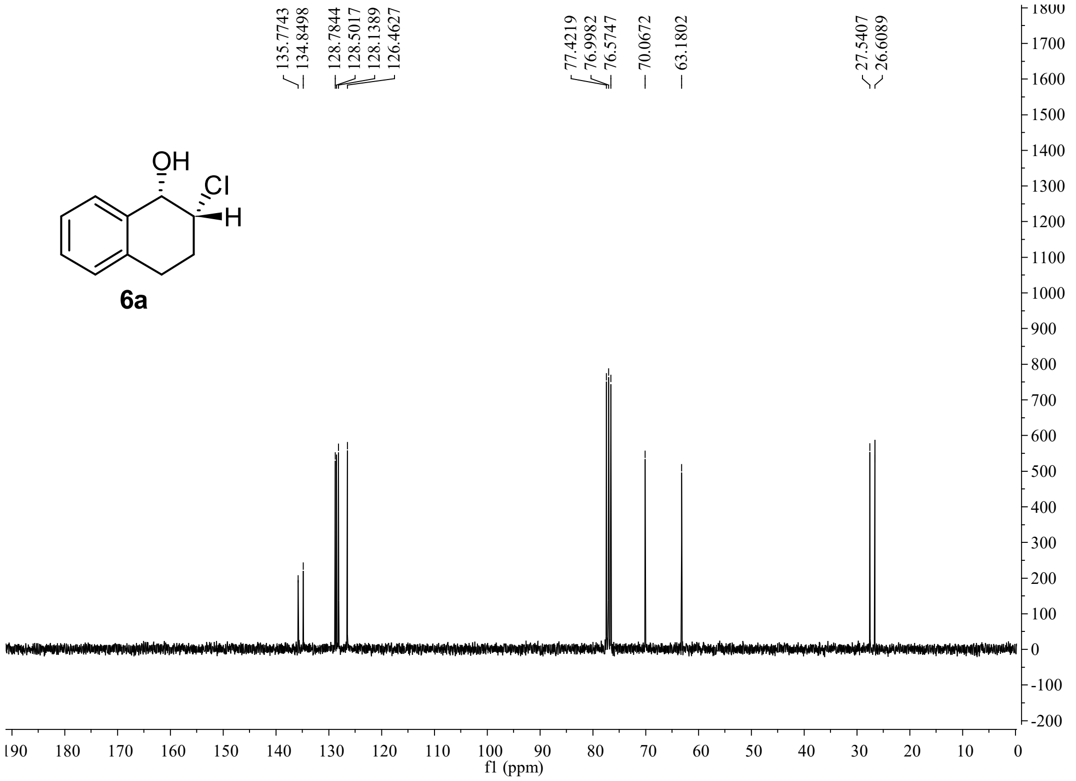
135.7743
134.8498
128.7844
128.5017
128.1389
126.4627
77.4219
76.9982
76.5747
70.0672
63.1802
27.5407
26.6089
OH
Cl
H
6a
f1 (ppm)

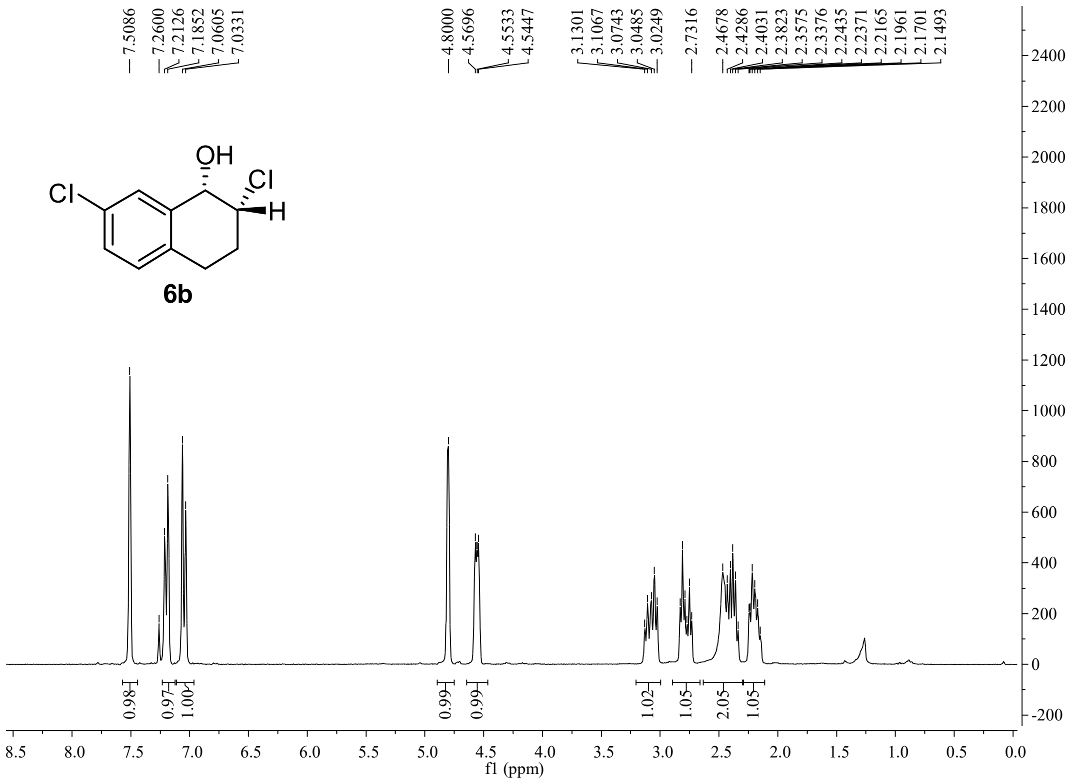
7.5086
7.2600
7.2126
7.1852
7.0605
7.0331
4.8000
4.5696
4.5533
4.5447
3.1301
3.1067
3.0743
3.0485
3.0249
2.7316
2.4678
2.4286
2.4031
2.3823
2.3575
2.3376
2.2435
2.2371
2.2165
2.1961
2.1701
2.1493
OH
Cl
Cl
H
6b
0.98
0.97
1.00
0.99
0.99
1.02
1.05
2.05
1.05
f1 (ppm)

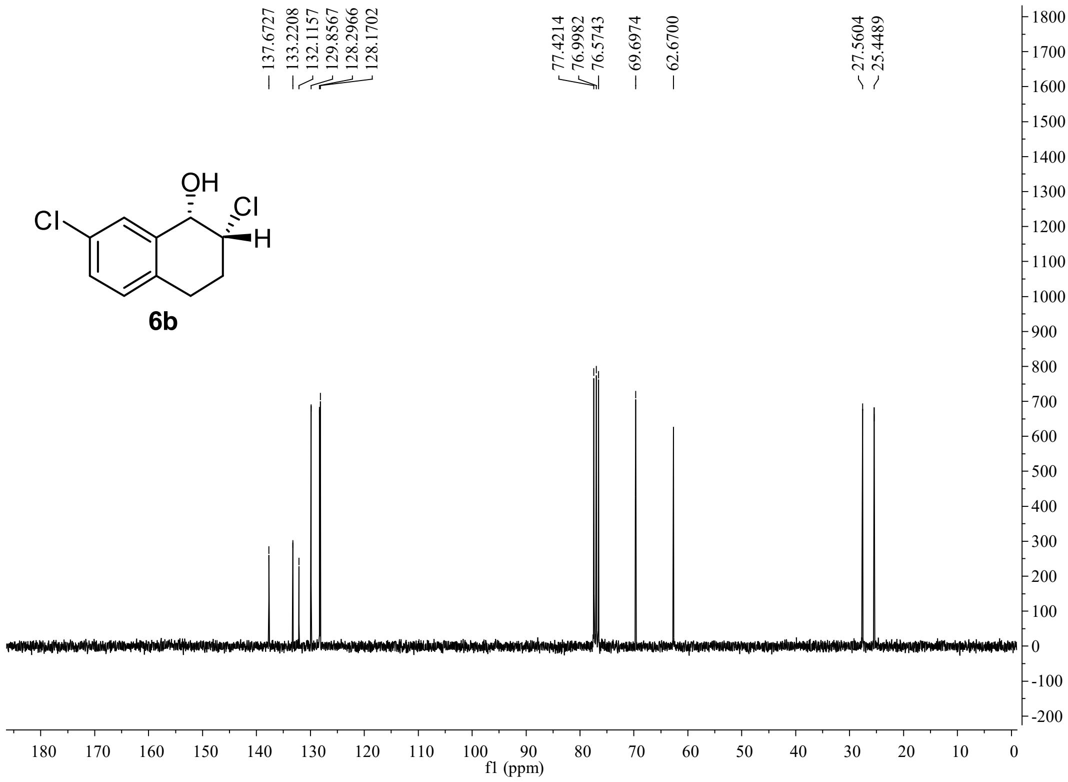
137.6727
133.2208
132.1157
129.8567
128.2966
128.1702
77.4214
76.9982
76.5743
69.6974
62.6700
27.5604
25.4489
OH
Cl
Cl
H
6b
f1 (ppm)

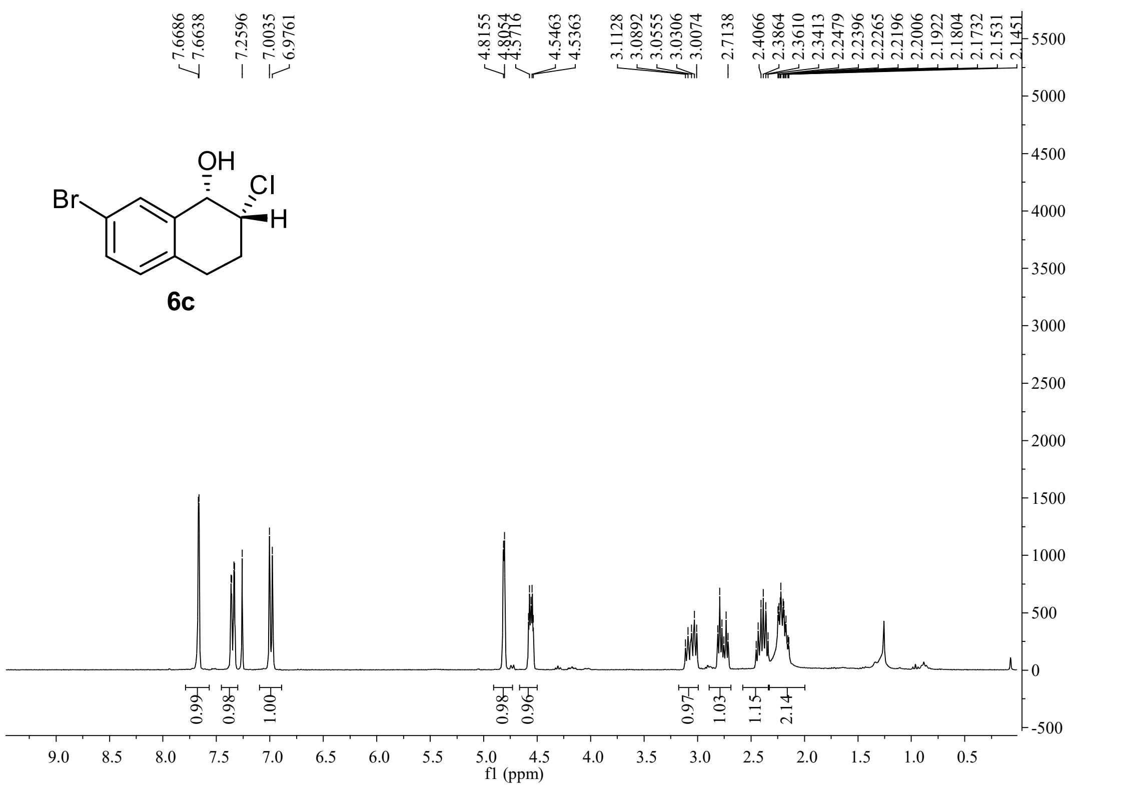
7.6686
7.6638
7.2596
7.0035
6.9761
4.8155
4.8054
4.5716
4.5463
4.5363
3.1128
3.0892
3.0555
3.0306
3.0074
2.7138
2.4066
2.3864
2.3610
2.3413
2.2479
2.2396
2.2265
2.2196
2.2006
2.1922
2.1804
2.1732
2.1531
2.1451
OH
Cl
Br
H
6c
0.99
0.98
1.00
0.98
0.96
0.97
1.03
1.15
2.14
f1 (ppm)

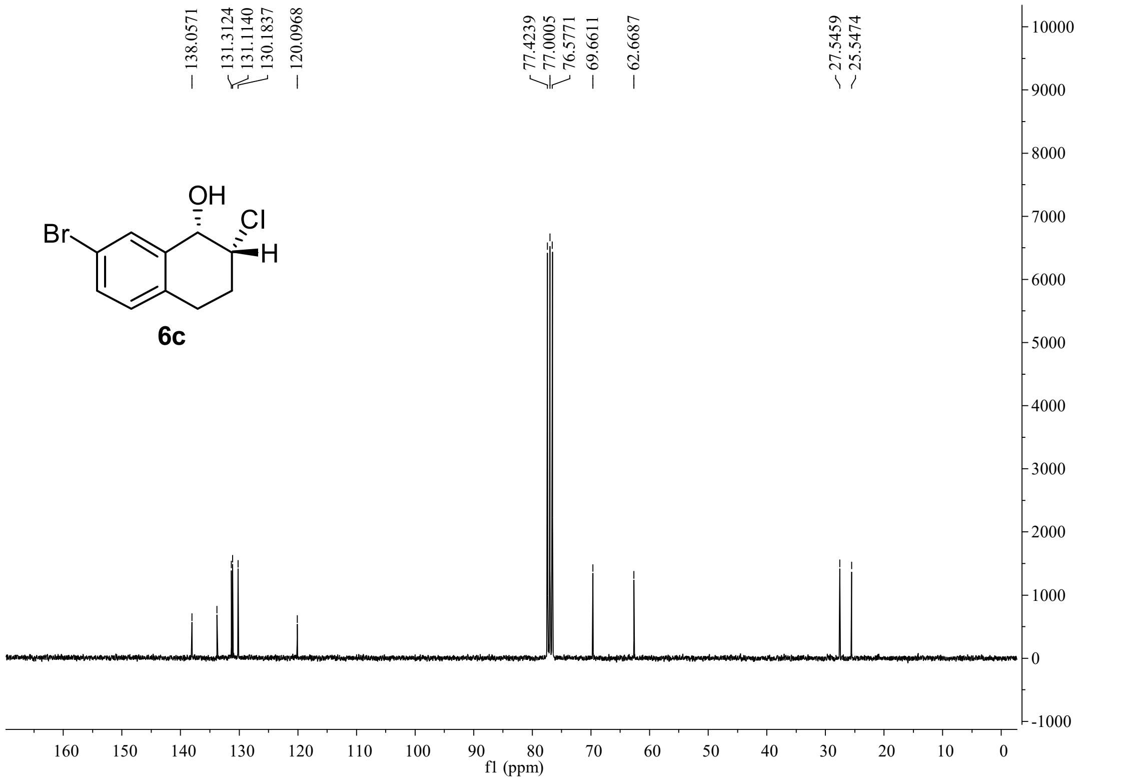
138.0571
131.3124
131.1140
130.1837
120.0968
77.4239
77.0005
76.5771
69.6611
62.6687
27.5459
25.5474
OH
Cl
Br
H
6c
f1 (ppm)

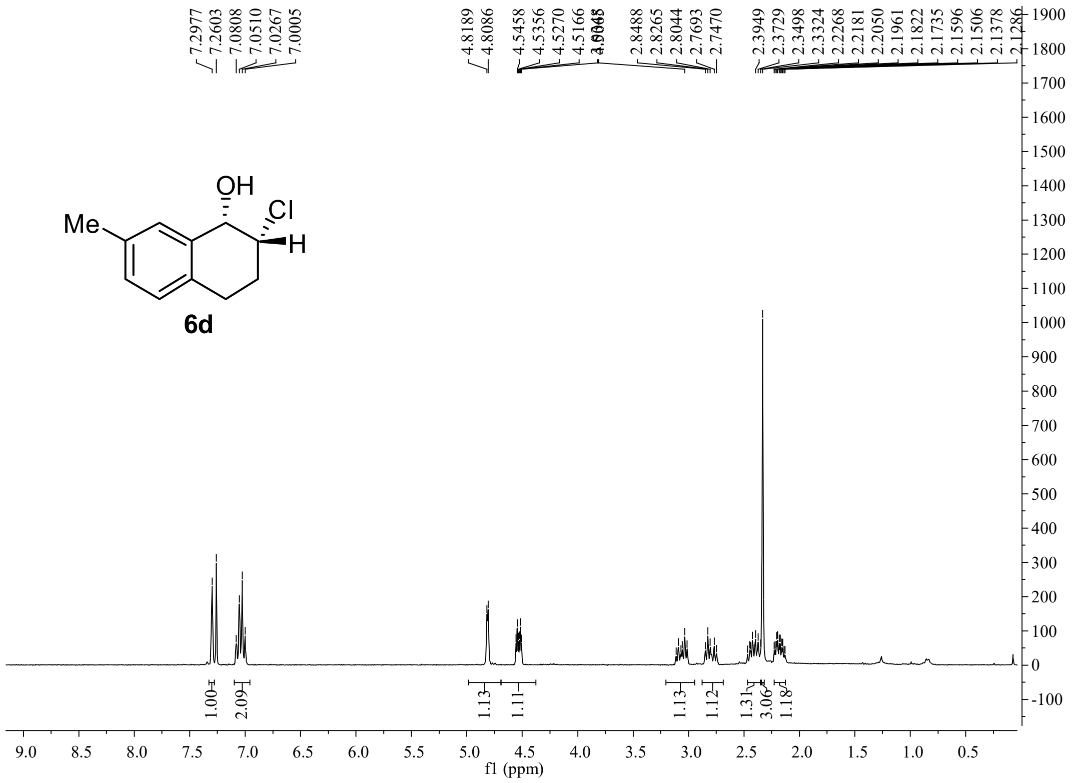
7.2977
7.2603
7.0808
7.0510
7.0267
7.0005
4.8189
4.8086
4.5458
4.5356
4.5270
4.5166
2.8488
2.8265
2.8044
2.7693
2.7470
2.3949
2.3729
2.3498
2.3324
2.2268
2.2181
2.2050
2.1961
2.1822
2.1735
2.1596
2.1506
2.1378
2.1286
OH
Cl
Me
H
6d
1.00
2.09
1.13
1.11
1.13
1.12
1.31
3.06
1.18
f1 (ppm)

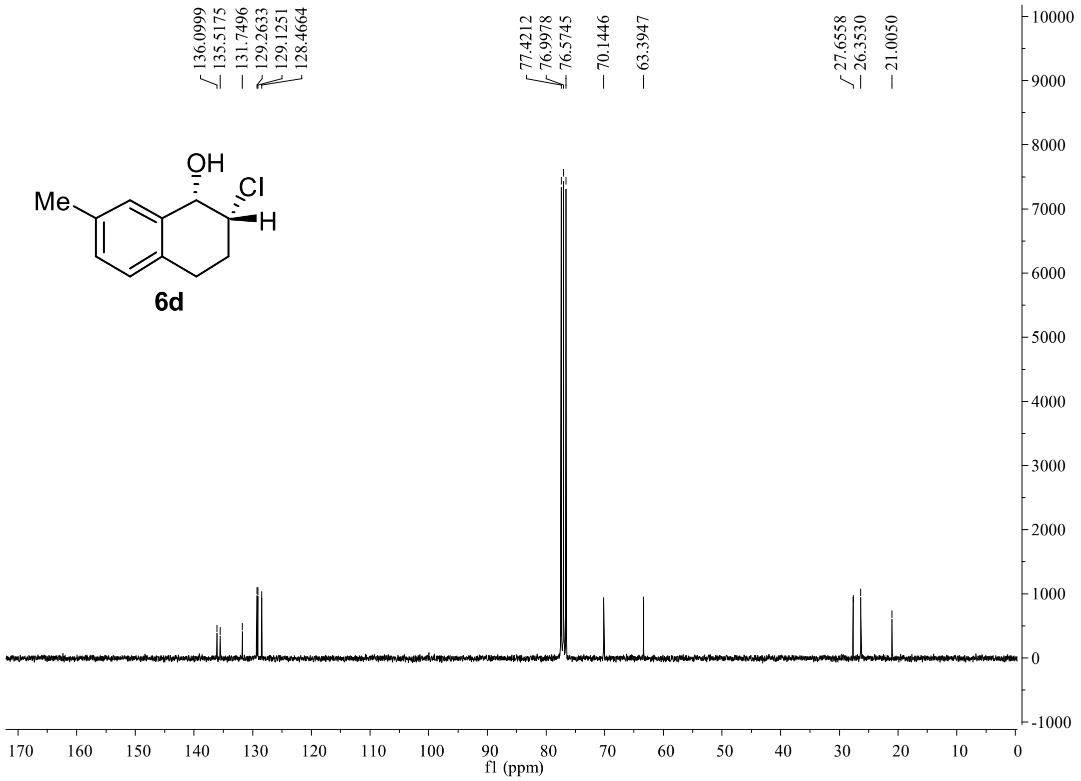
136.0999
135.5175
131.7496
129.2633
129.1251
128.4664
77.4212
76.9978
76.5745
70.1446
63.3947
27.6558
26.3530
21.0050
OH
Cl
Me
H
6d
f1 (ppm)

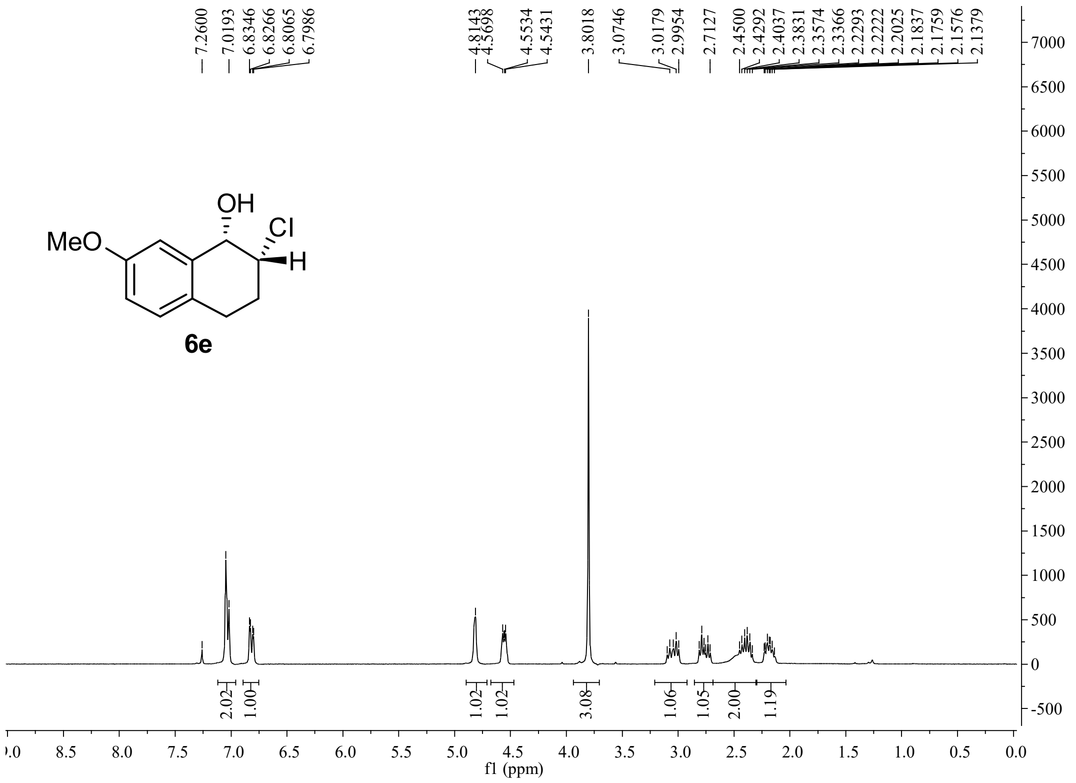

7.2600
7.0193
6.8346
6.8266
6.8065
6.7986
4.8143
4.5698
4.5534
4.5431
3.8018
3.0746
3.0179
2.9954
2.7127
2.4500
2.4292
2.4037
2.3831
2.3574
2.3366
2.2293
2.2222
2.2025
2.1837
2.1759
2.1576
2.1379
MeO
OH
Cl
H
6e
2.02
1.00
1.02
1.02
3.08
1.06
1.05
2.00
1.19
fl (ppm)

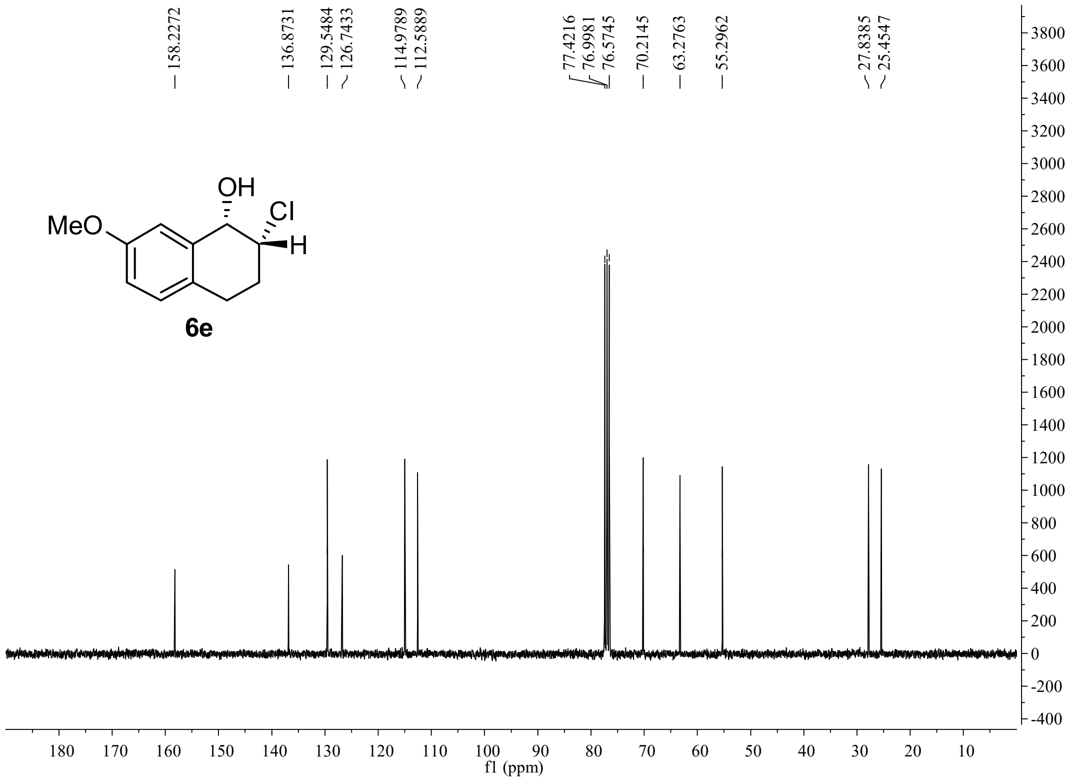

158.2272
136.8731
129.5484
126.7433
114.9789
112.5889
77.4216
76.9981
76.5745
70.2145
63.2763
55.2962
27.8385
25.4547
MeO
OH
Cl
H
6e
fl (ppm)

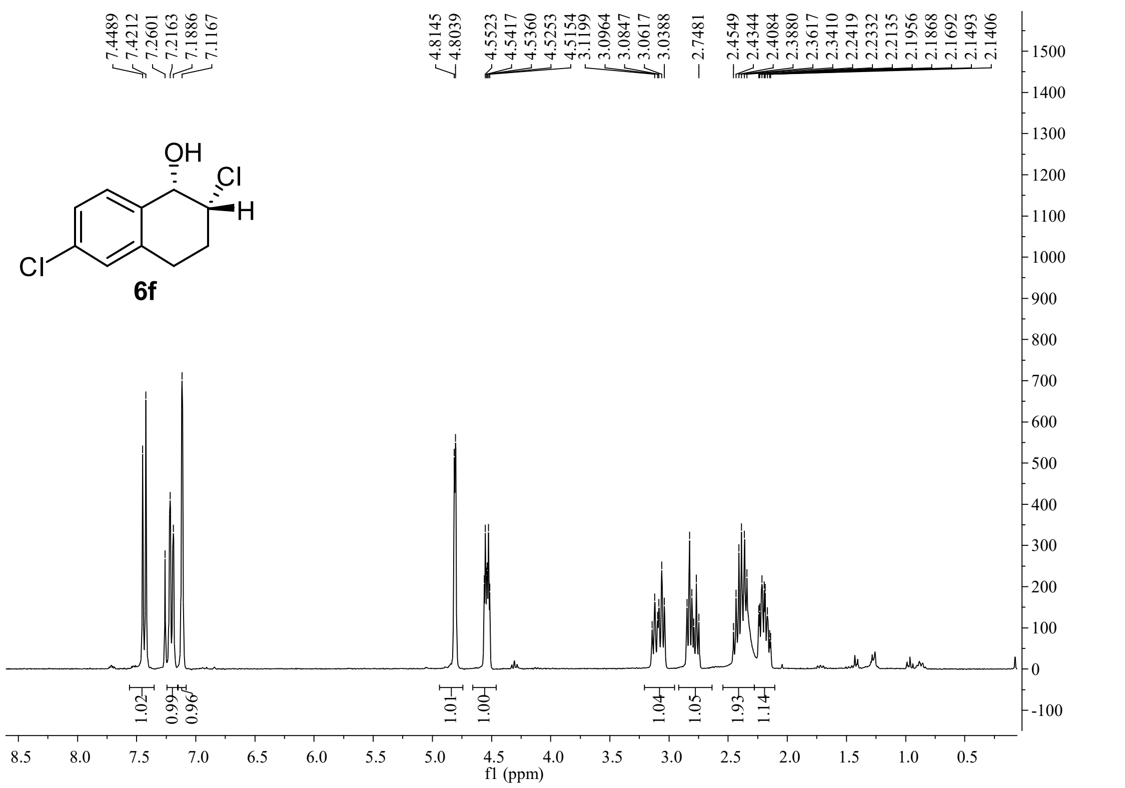
7.4489
7.4212
7.2601
7.2163
7.1886
7.1167
4.8145
4.8039
4.5523
4.5417
4.5360
4.5253
4.5154
3.1199
3.0964
3.0847
3.0617
3.0388
2.7481
2.4549
2.4344
2.4084
2.3880
2.3617
2.3410
2.2419
2.2332
2.2135
2.1956
2.1868
2.1692
2.1493
2.1406
OH
Cl
H
Cl
6f
1.02
0.99
0.96
1.01
1.00
1.04
1.05
1.93
1.14
f1 (ppm)

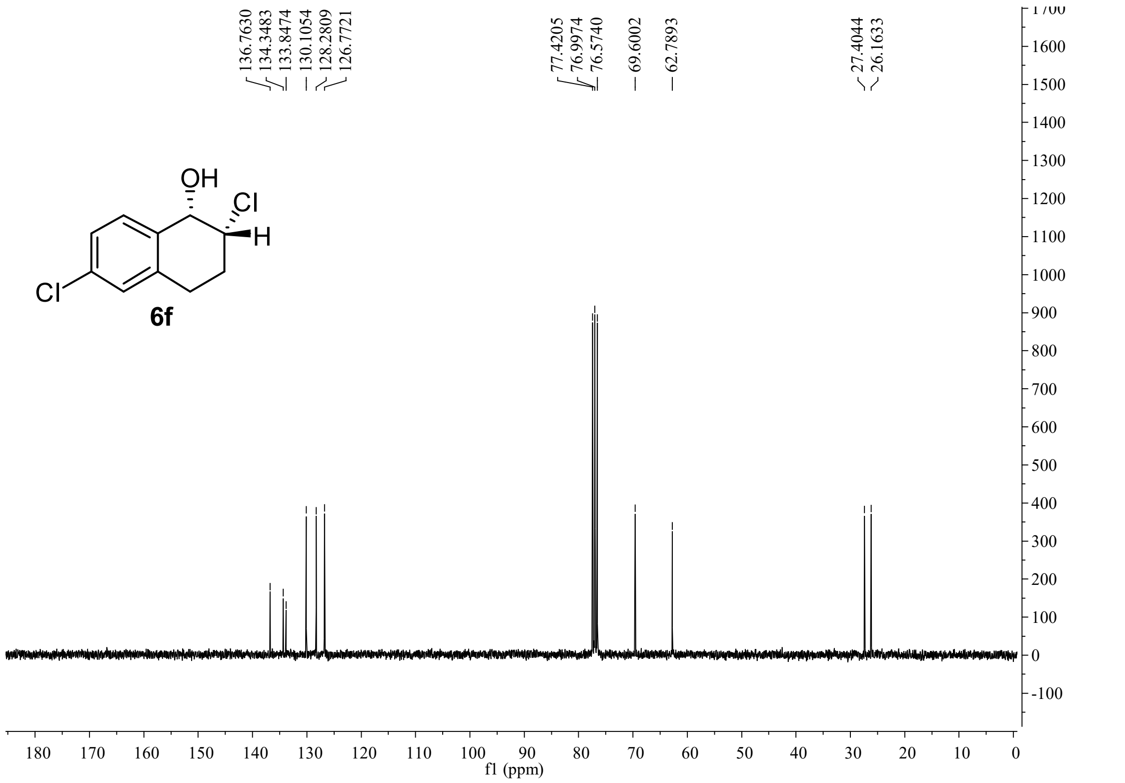
136.7630
134.3483
133.8474
130.1054
128.2809
126.7721
77.4205
76.9974
76.5740
69.6002
62.7893
27.4044
26.1633
OH
Cl
H
Cl
6f
f1 (ppm)

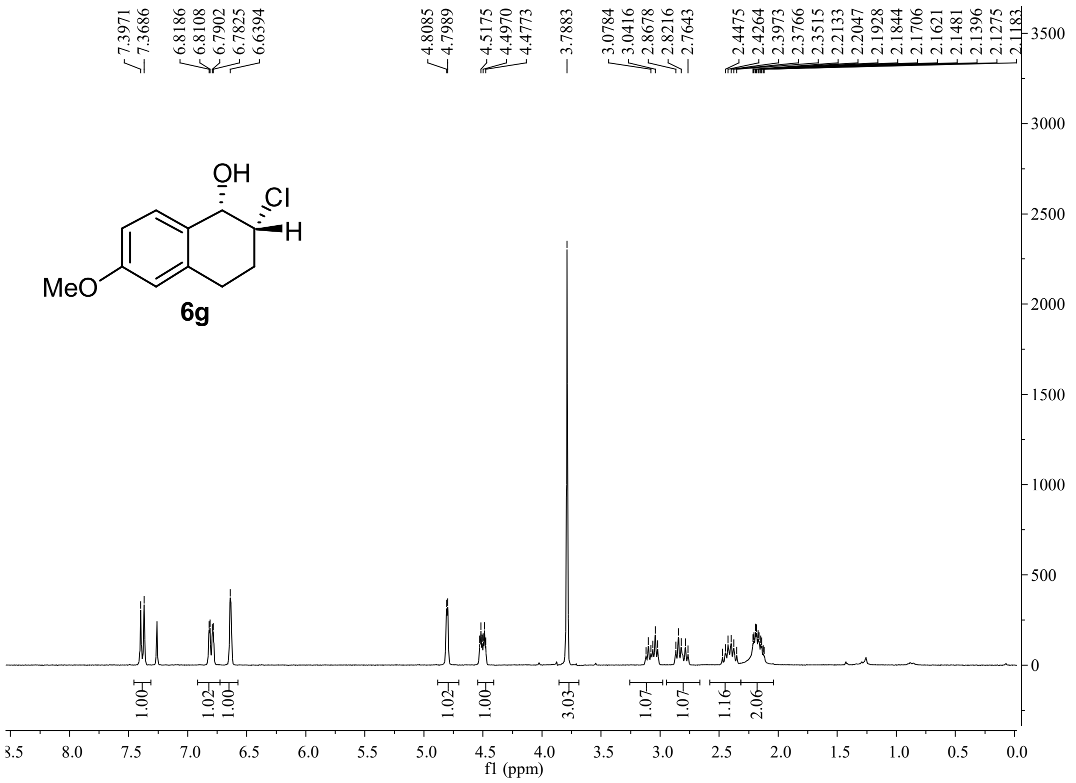

7.3971
7.3686
6.8186
6.8108
6.7902
6.7825
6.6394
4.8085
4.7989
4.5175
4.4970
4.4773
3.7883
3.0784
3.0416
2.8678
2.8216
2.7643
2.4475
2.4264
2.3973
2.3766
2.3515
2.2133
2.2047
2.1928
2.1844
2.1706
2.1621
2.1481
2.1396
2.1275
2.1183
OH
Cl
H
MeO
6g
1.00
1.02
1.00
1.02
1.00
3.03
1.07
1.07
1.16
2.06
f1 (ppm)

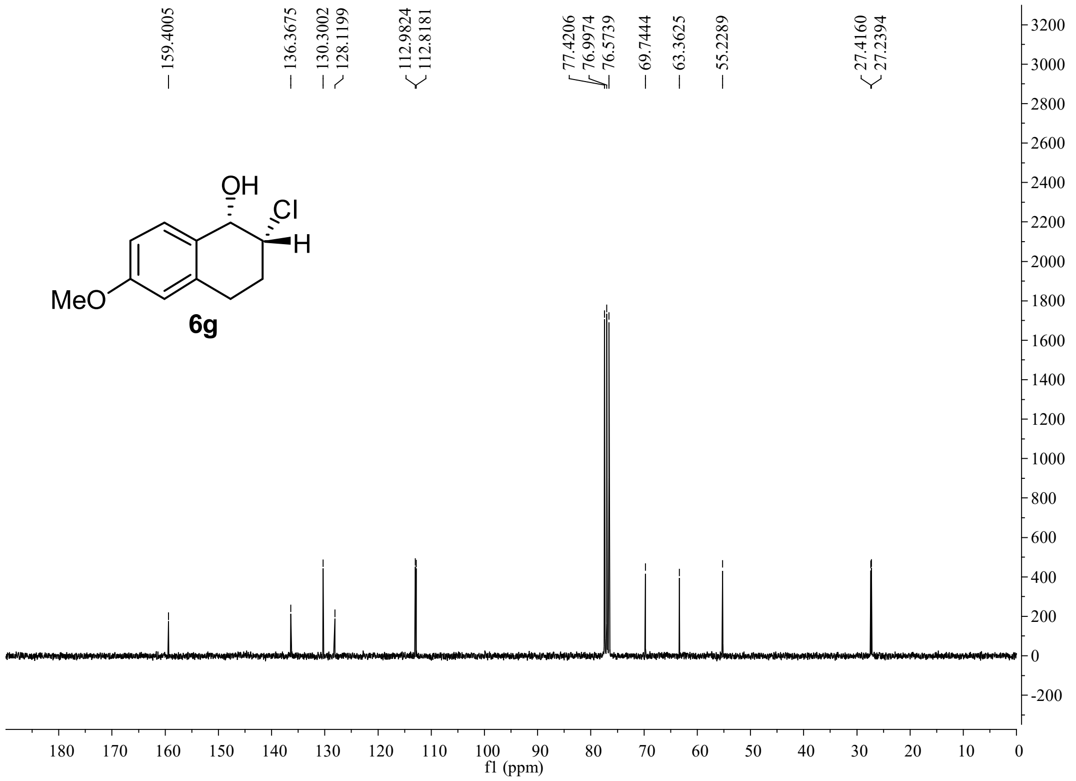

159.4005
136.3675
130.3002
128.1199
112.9824
112.8181
77.4206
76.9974
76.5739
69.7444
63.3625
55.2289
27.4160
27.2394
OH
Cl
H
MeO
6g
f1 (ppm)

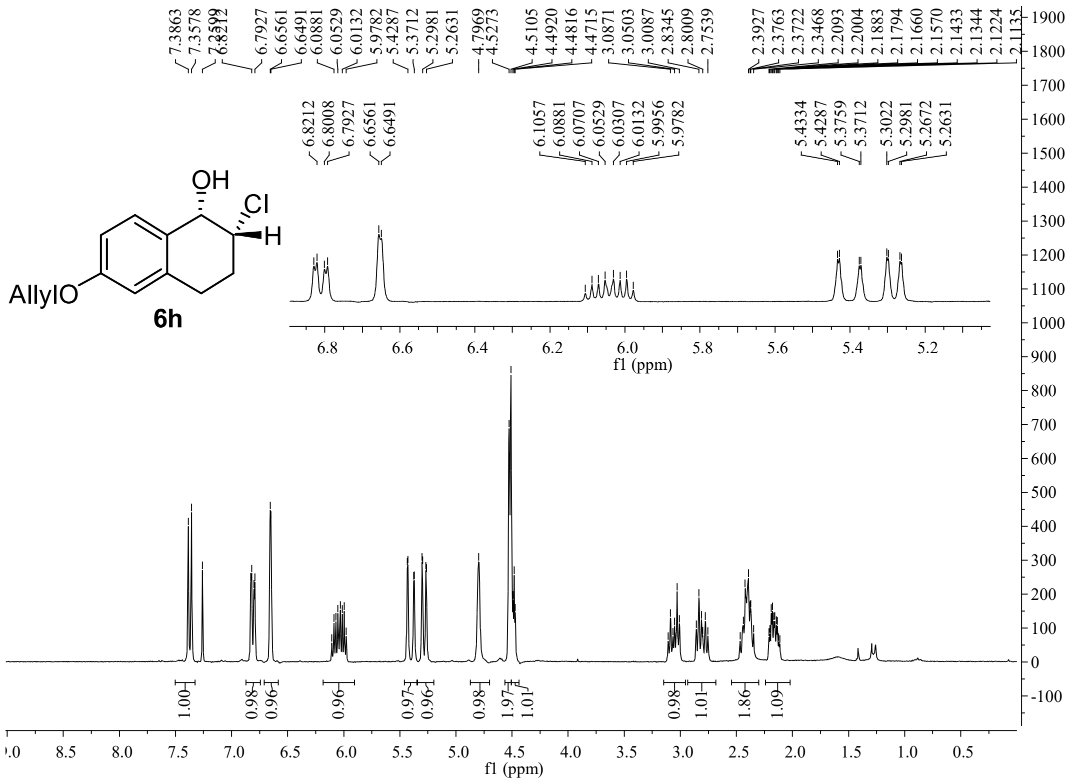
7.3863
7.3578
7.2599
6.8212
6.7927
6.6561
6.6491
6.0881
6.0529
6.0132
5.9782
5.4287
5.3712
5.2981
5.2631
4.7969
4.5273
4.5105
4.4920
4.4816
4.4715
3.0871
3.0503
3.0087
2.8345
2.8009
2.7539
2.3927
2.3763
2.3722
2.3468
2.2093
2.2004
2.1883
2.1794
2.1660
2.1570
2.1433
2.1344
2.1224
2.1135
6.8212
6.8008
6.7927
6.6561
6.6491
6.1057
6.0881
6.0707
6.0529
6.0307
6.0132
5.9956
5.9782
5.4334
5.4287
5.3759
5.3712
5.3022
5.2981
5.2672
5.2631
OH
Cl
H
AllylO
6h
1.00
0.98
0.96
0.96
0.97
0.96
0.98
1.97
1.01
0.98
1.01
1.86
1.09
f1 (ppm)

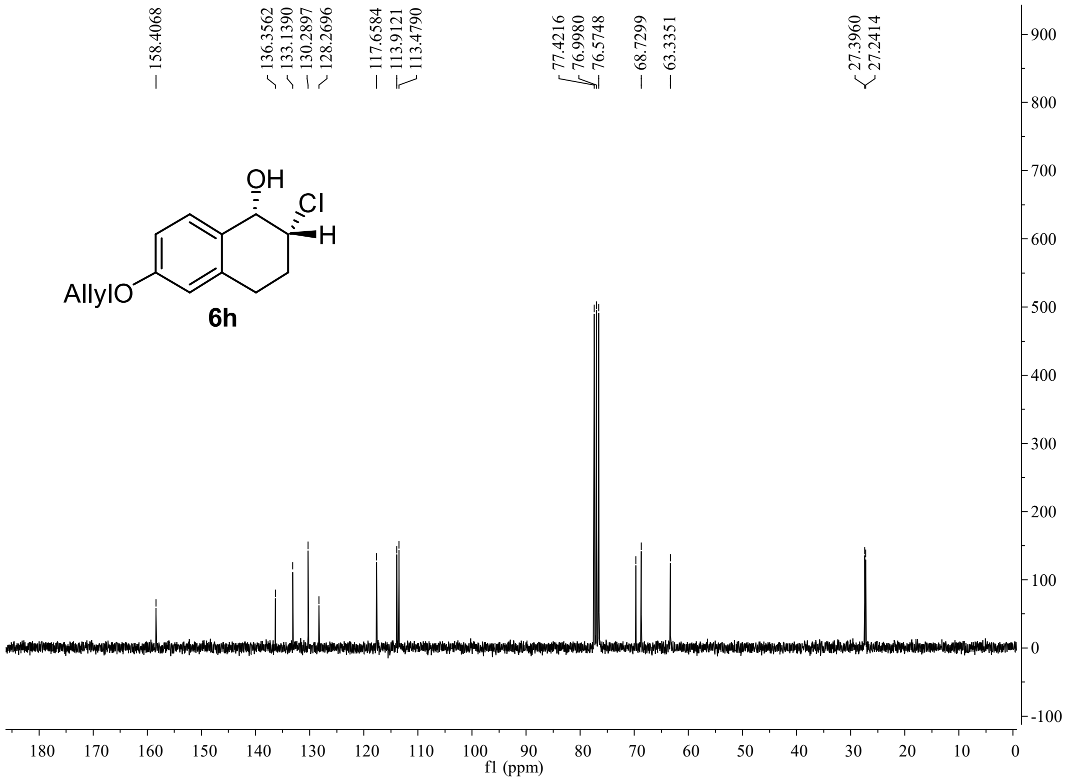
158.4068
136.3562
133.1390
130.2897
128.2696
117.6584
113.9121
113.4790
77.4216
76.9980
76.5748
68.7299
63.3351
27.3960
27.2414
OH
Cl
H
AllylO
6h
f1 (ppm)

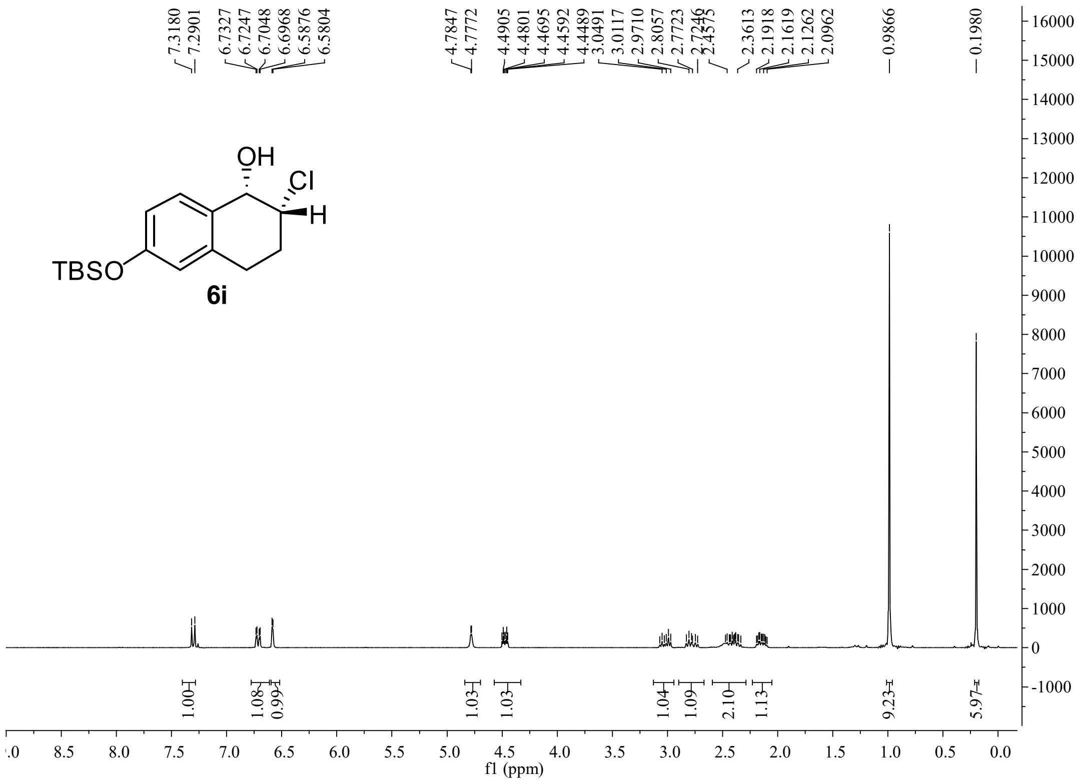

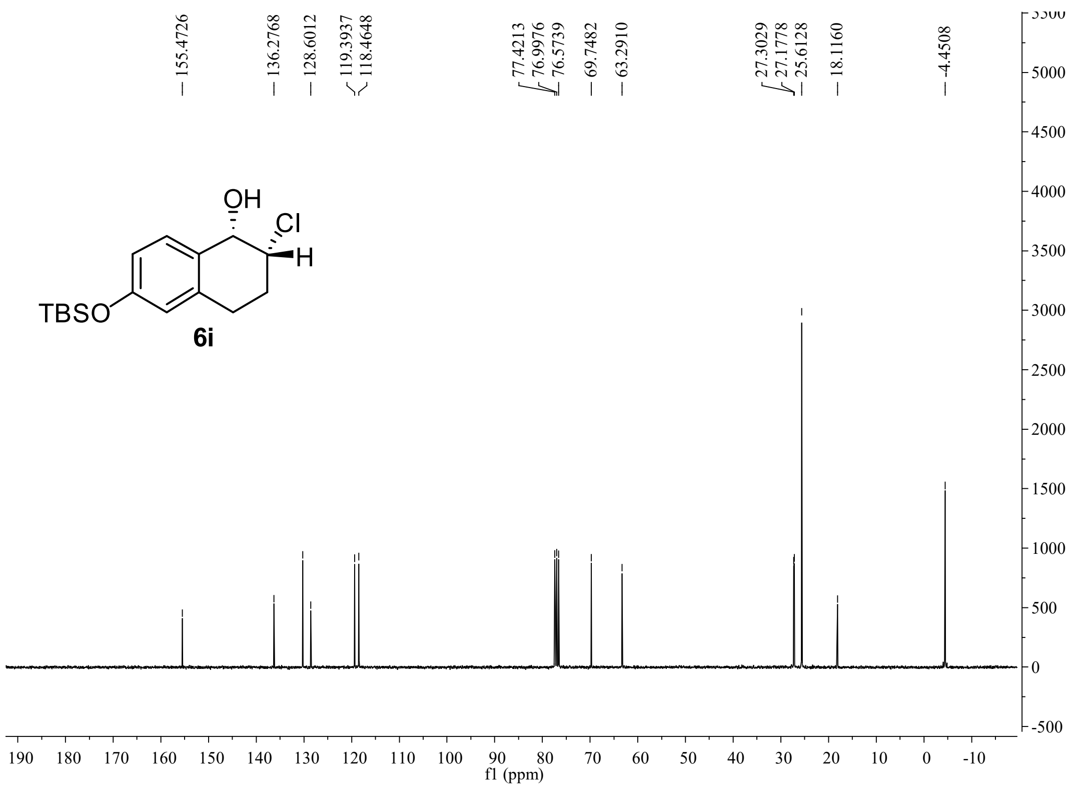

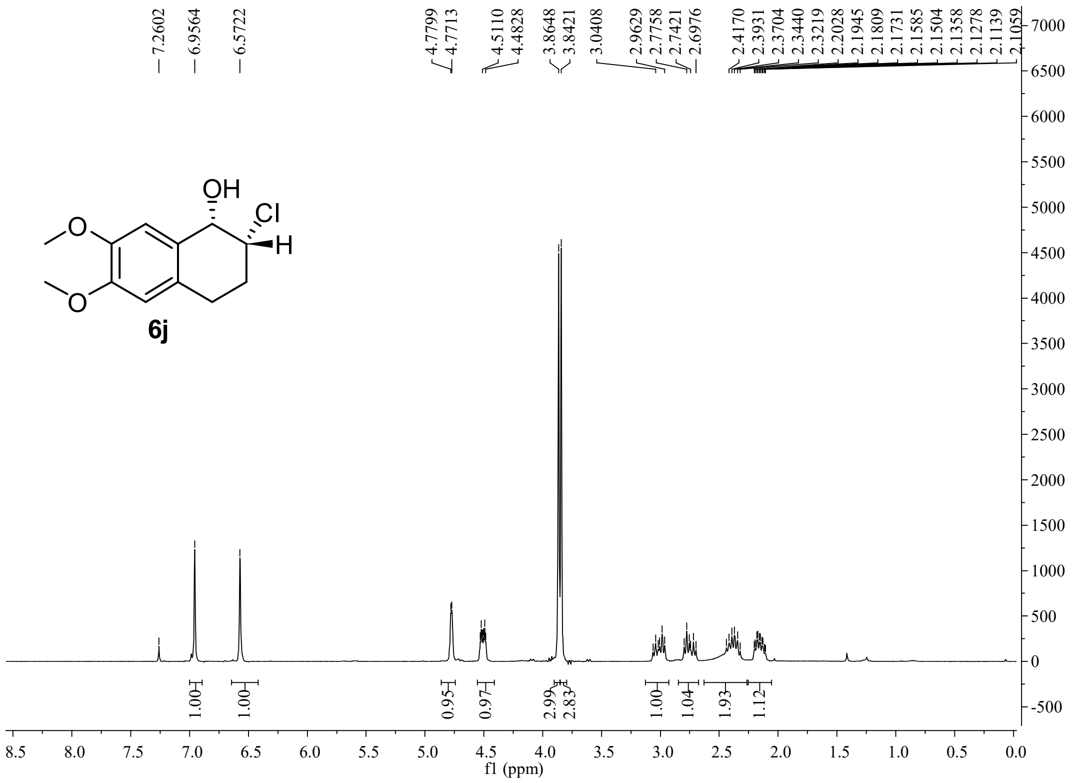

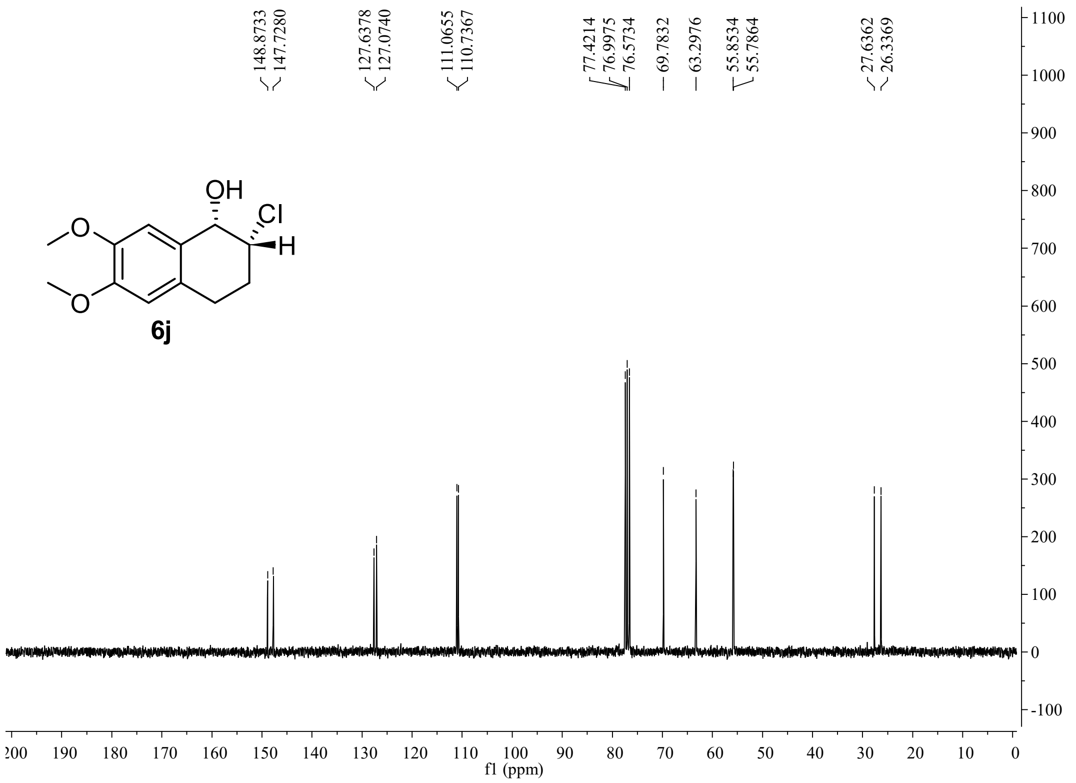

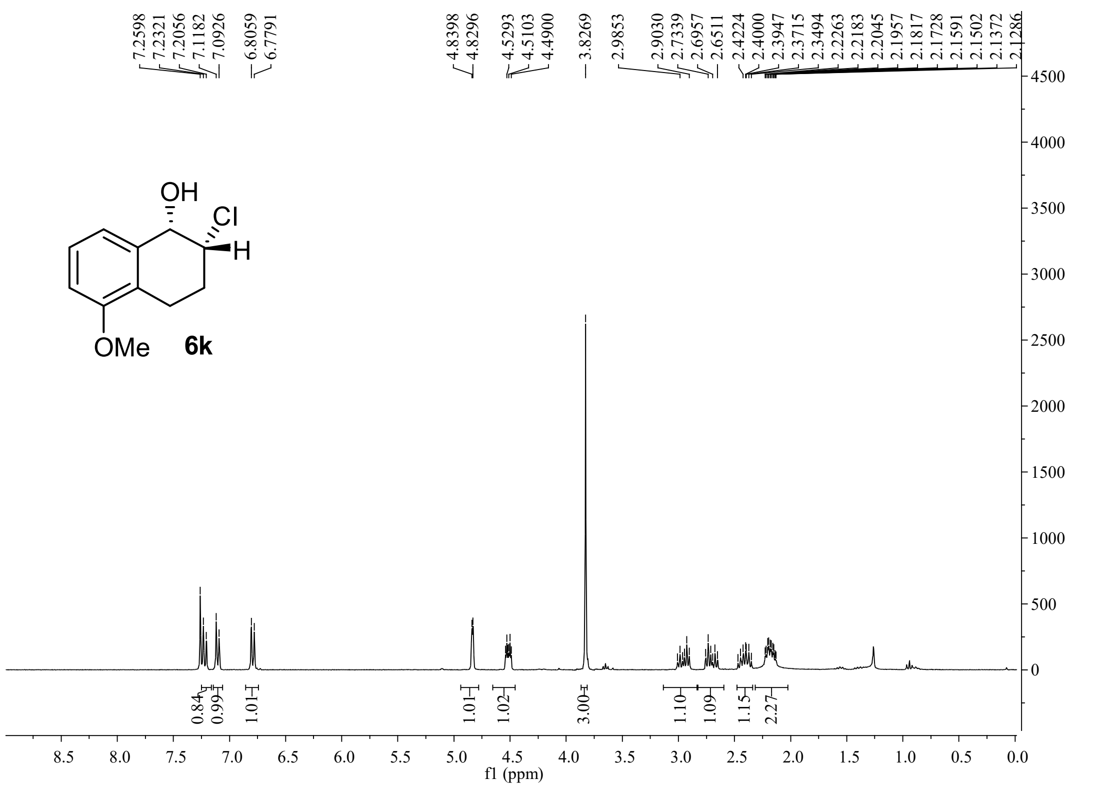
7.2598
7.2321
7.2056
7.1182
7.0926
6.8059
6.7791
4.8398
4.8296
4.5293
4.5103
4.4900
3.8269
2.9853
2.9030
2.7339
2.6957
2.6511
2.4224
2.4000
2.3947
2.3715
2.3494
2.2263
2.2183
2.2045
2.1957
2.1817
2.1728
2.1591
2.1502
2.1372
2.1286
OH
Cl
H
OMe
6k
0.84
0.99
1.01
1.01
1.02
3.00
1.10
1.09
1.15
2.27
fl (ppm)

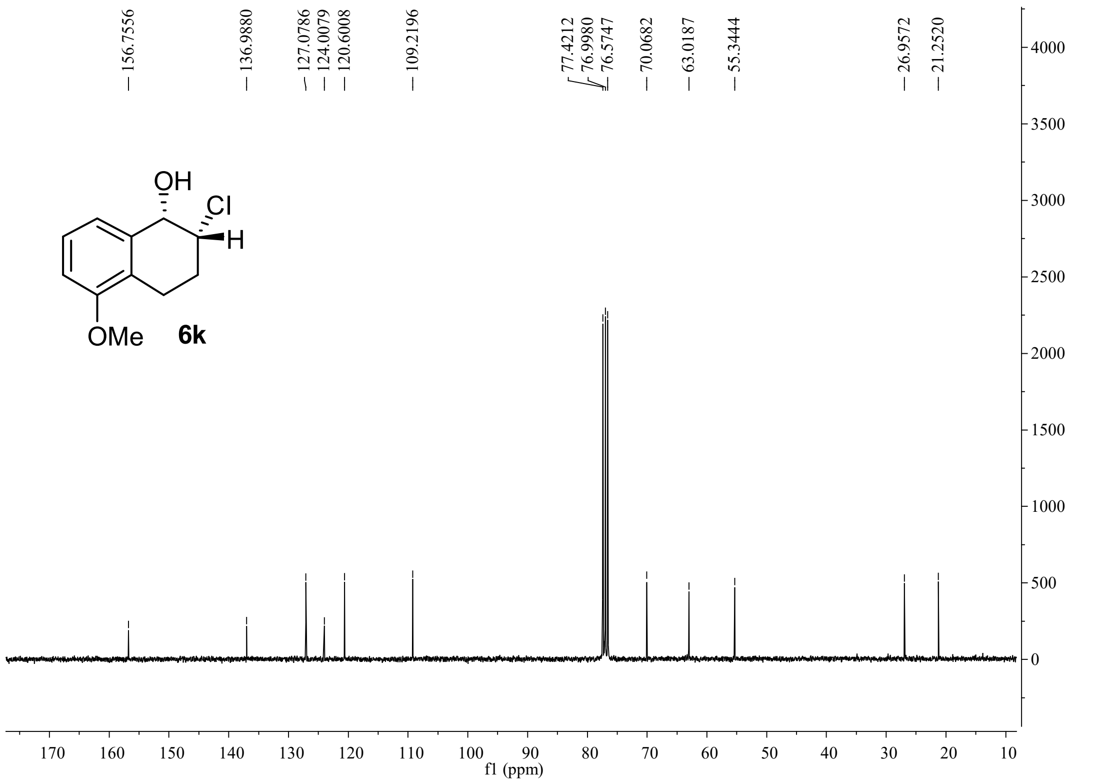
156.7556
136.9880
127.0786
124.0079
120.6008
109.2196
77.4212
76.9980
76.5747
70.0682
63.0187
55.3444
26.9572
21.2520
OH
Cl
H
OMe
6k
fl (ppm)

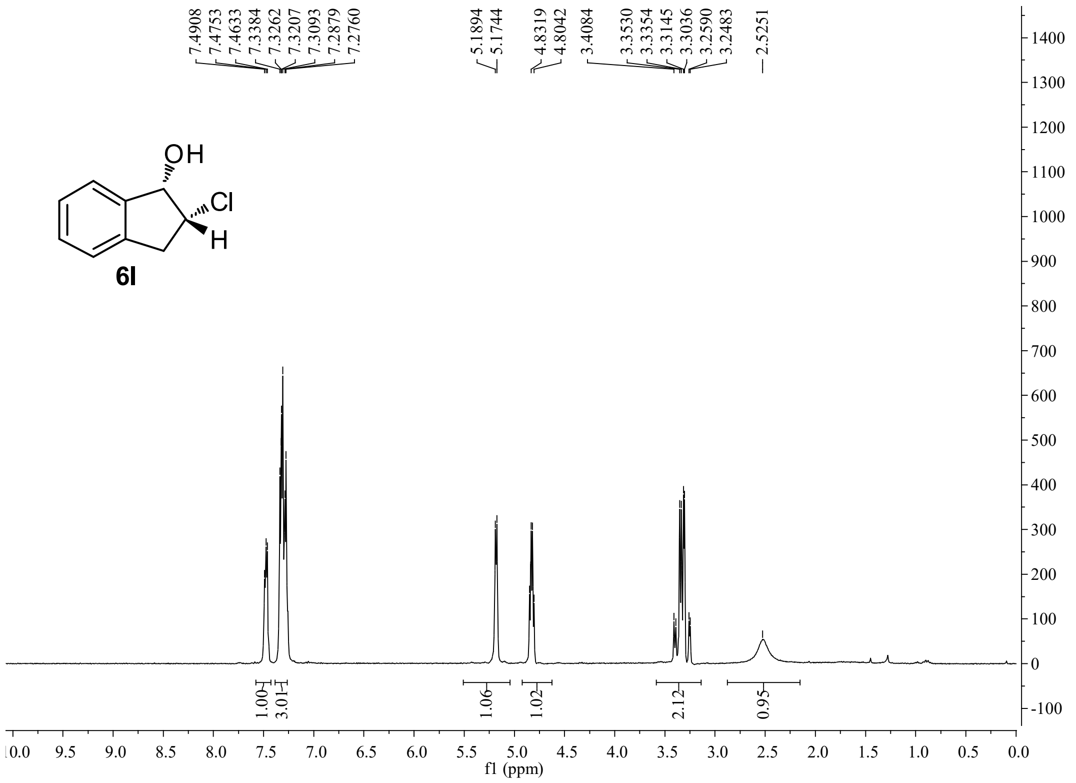
7.4908
7.4753
7.4633
7.3384
7.3262
7.3207
7.3093
7.2879
7.2760
5.1894
5.1744
4.8319
4.8042
3.4084
3.3530
3.3354
3.3145
3.3036
3.2590
3.2483
2.5251
OH
Cl
H
6l
1.00
3.01
1.06
1.02
2.12
0.95
f1 (ppm)

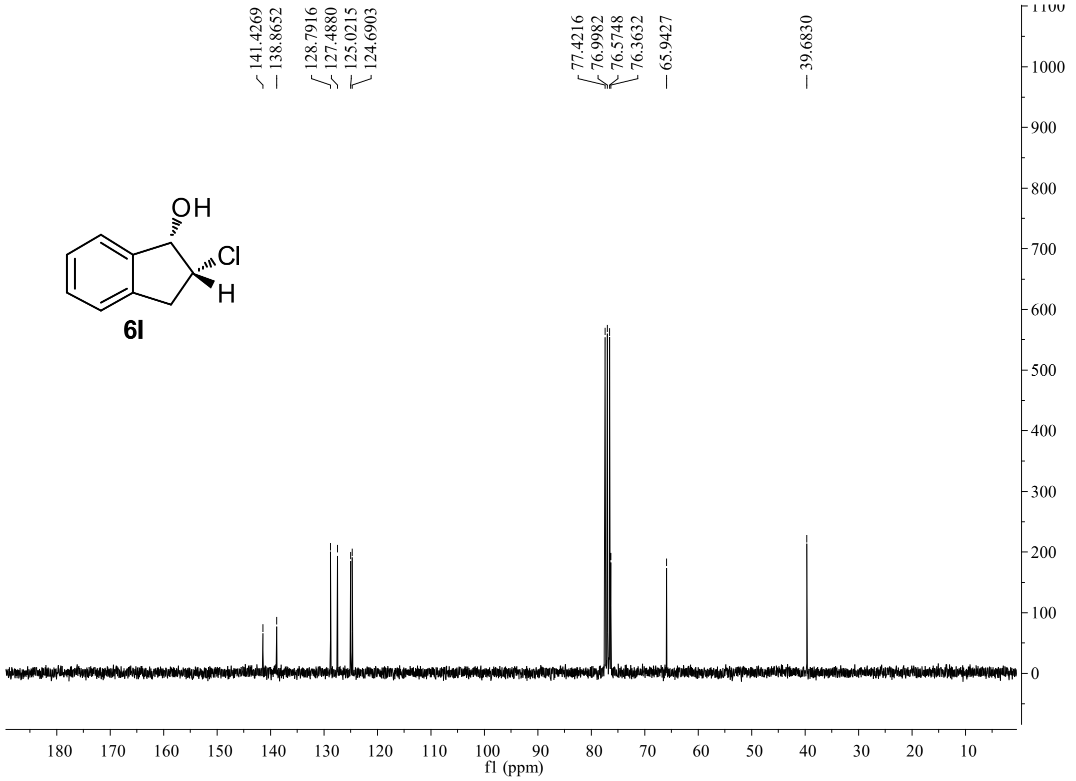
141.4269
138.8652
128.7916
127.4880
125.0215
124.6903
77.4216
76.9982
76.5748
76.3632
65.9427
39.6830
OH
Cl
H
6l
f1 (ppm)

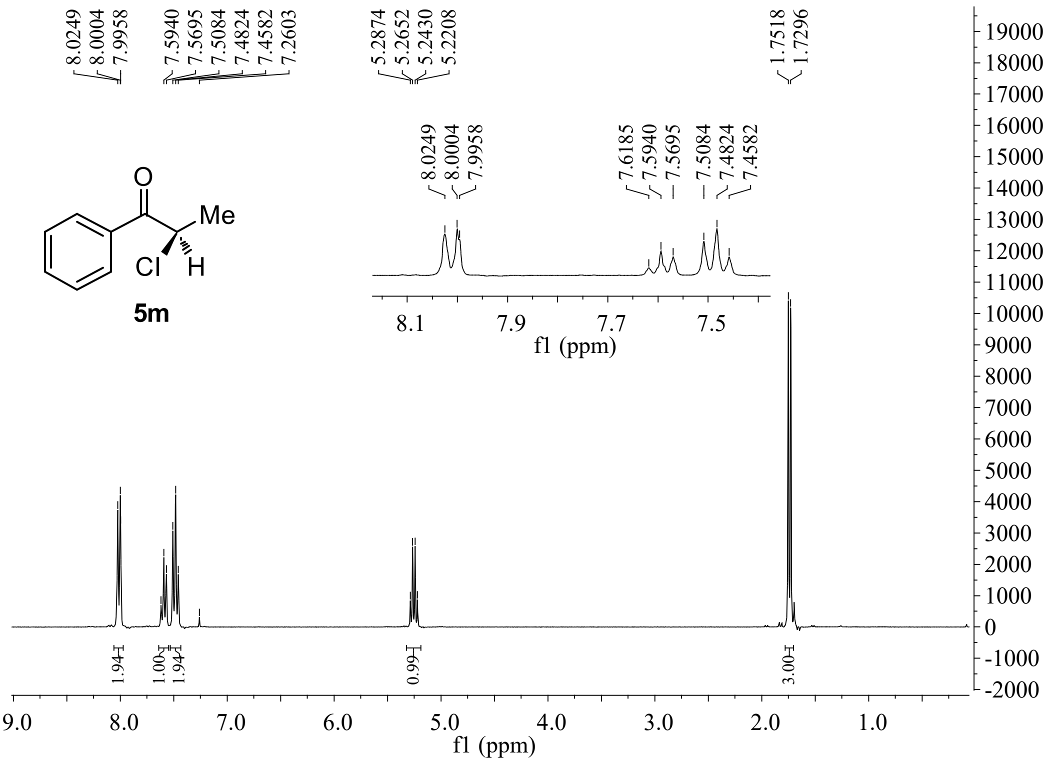
8.0249
8.0004
7.9958
7.5940
7.5695
7.5084
7.4824
7.4582
7.2603
5.2874
5.2652
5.2430
5.2208
1.7518
1.7296
O
Me
Cl
H
5m
8.0249
8.0004
7.9958
7.6185
7.5940
7.5695
7.5084
7.4824
7.4582
8.1
7.9
7.7
7.5
f1 (ppm)
1.94
1.00
1.94
0.99
3.00
9.0
8.0
7.0
6.0
5.0
4.0
3.0
2.0
1.0
f1 (ppm)
19000
18000
17000
16000
15000
14000
13000
12000
11000
10000
9000
8000
7000
6000
5000
4000
3000
2000
1000
0
-1000
-2000

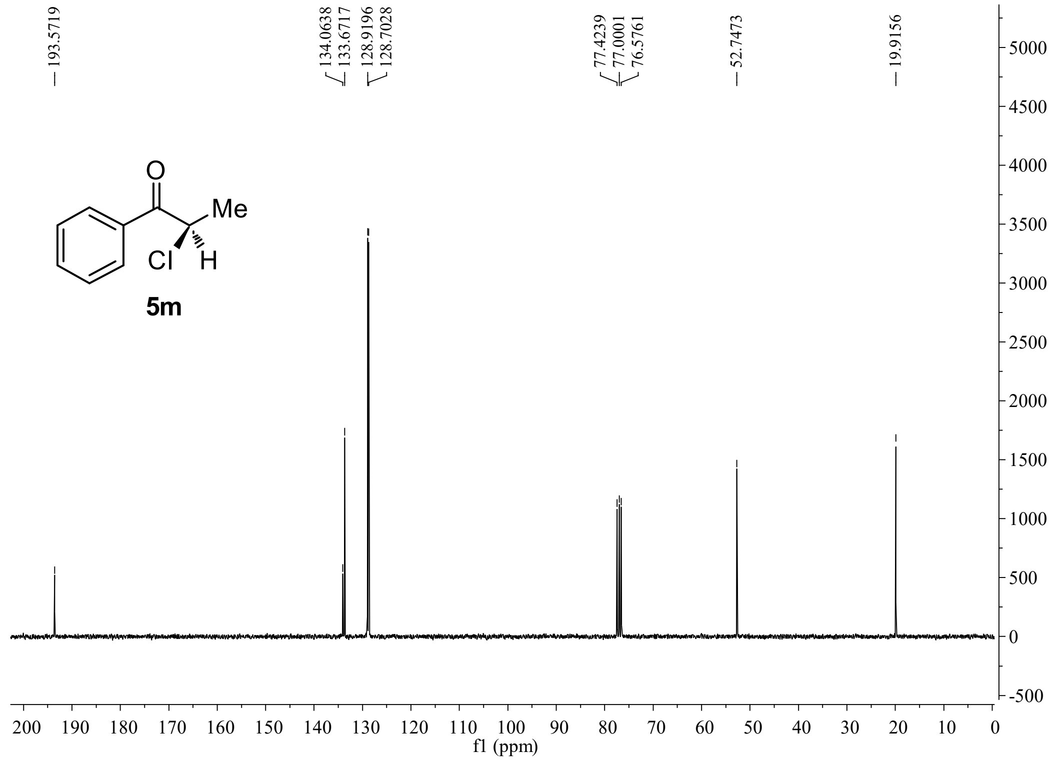
193.5719
134.0638
133.6717
128.9196
128.7028
77.4239
77.0001
76.5761
52.7473
19.9156
O
Me
Cl
H
5m
200
190
180
170
160
150
140
130
120
110
100
90
80
70
60
50
40
30
20
10
0
f1 (ppm)
5000
4500
4000
3500
3000
2500
2000
1500
1000
500
0
-500

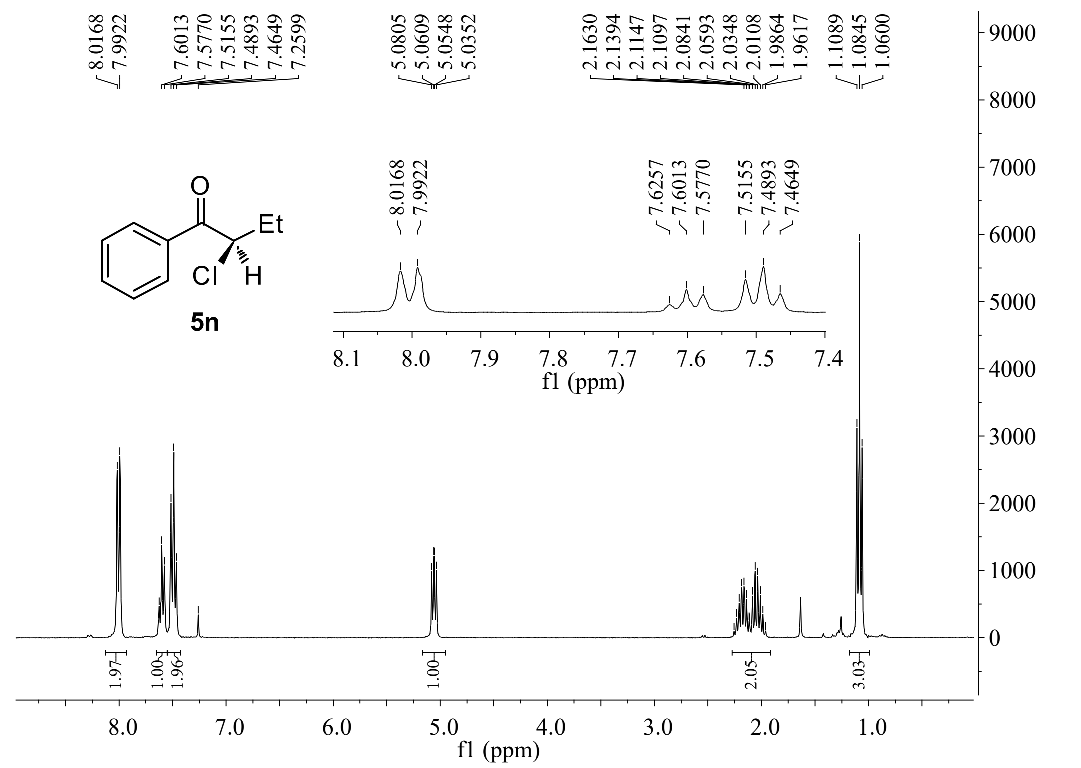
8.0168
7.9922
7.6013
7.5770
7.5155
7.4893
7.4649
7.2599
5.0805
5.0609
5.0548
5.0352
2.1630
2.1394
2.1147
2.1097
2.0841
2.0593
2.0348
2.0108
1.9864
1.9617
1.1089
1.0845
1.0600
O
Et
Cl
H
5n
7.6257
f1 (ppm)
1.97
1.00
1.96
1.00
2.05
3.03
f1 (ppm)

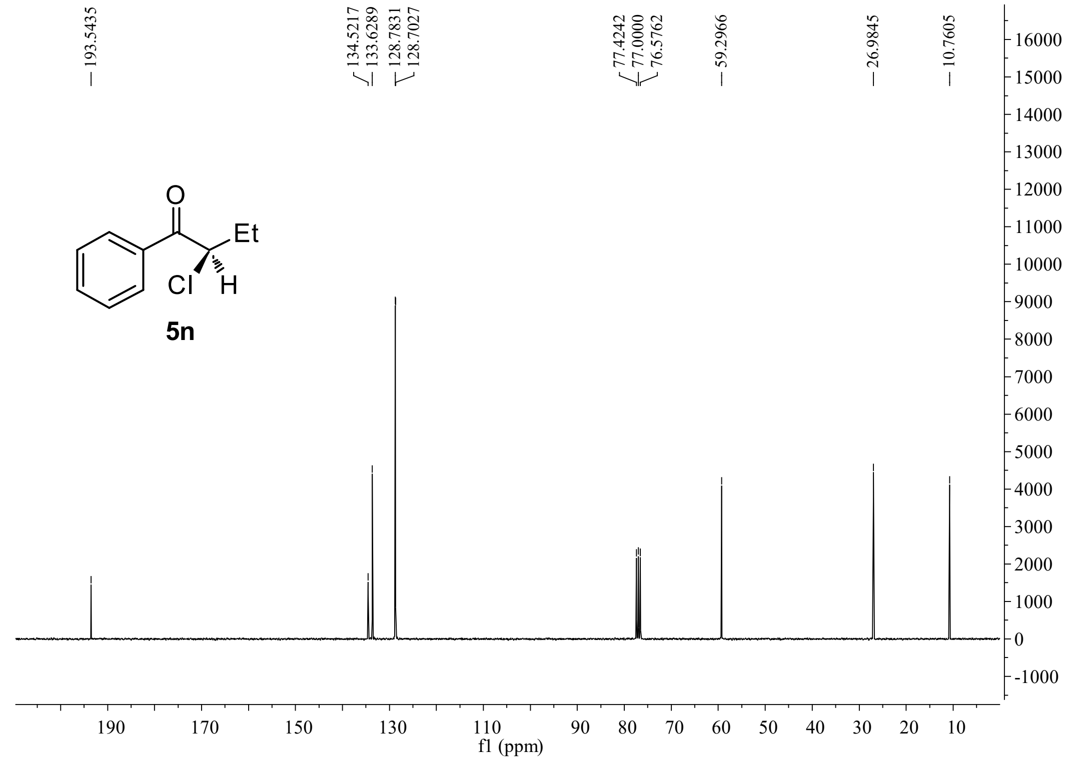
193.5435
134.5217
133.6289
128.7831
128.7027
77.4242
77.0000
76.5762
59.2966
26.9845
10.7605
O
Et
Cl
H
5n
f1 (ppm)

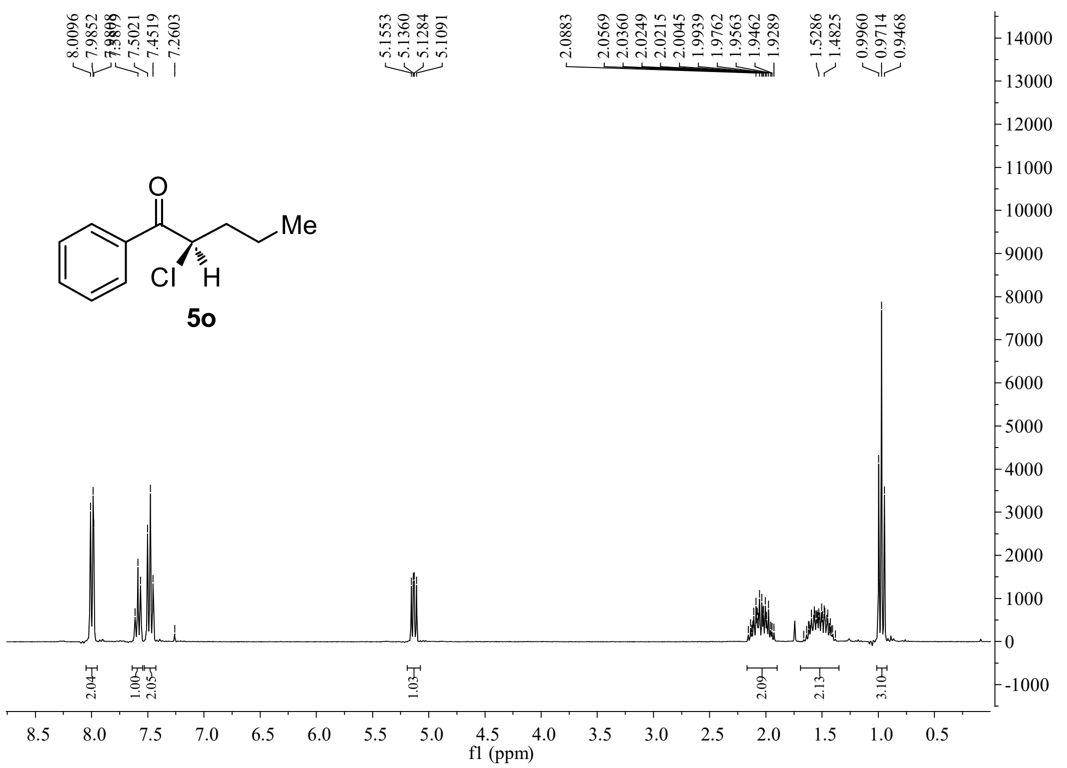
8.0096
7.9852
7.5021
7.4519
7.2603
5.1553
5.1360
5.1284
5.1091
2.0883
2.0569
2.0360
2.0249
2.0215
2.0045
1.9939
1.9762
1.9563
1.9462
1.9289
1.5286
1.4825
0.9960
0.9714
0.9468
O
Me
Cl
H
5o
2.04
1.00
2.05
1.03
2.09
2.13
3.10
f1 (ppm)

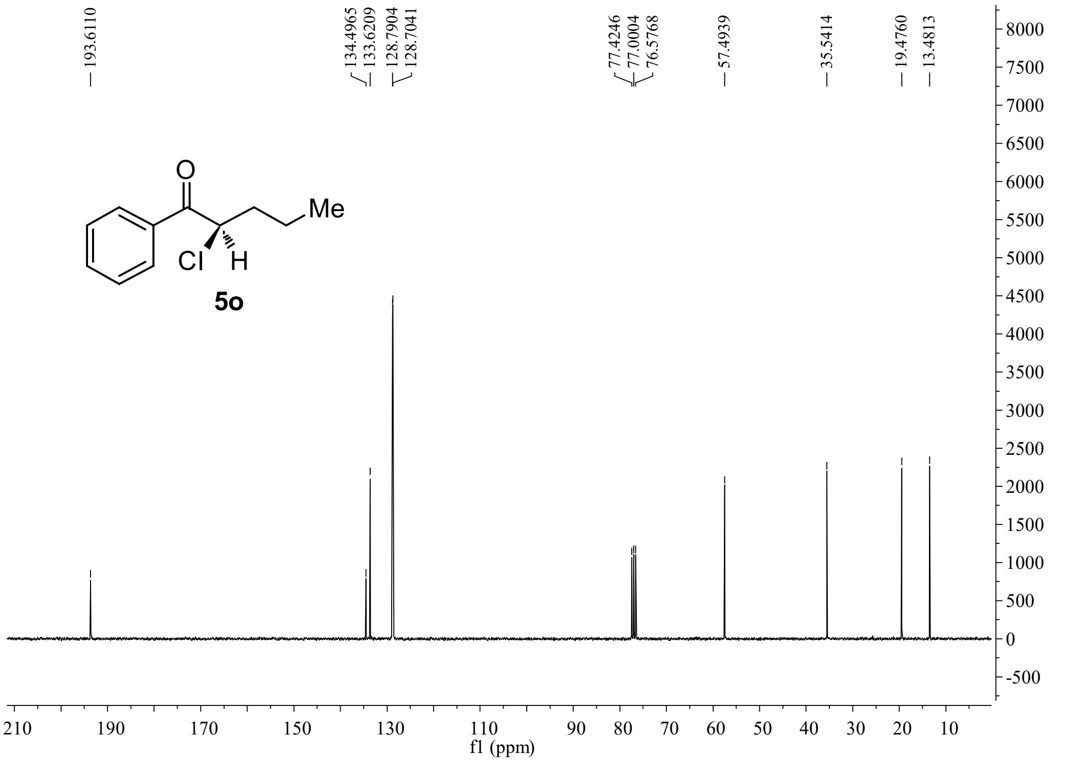
193.6110
134.4965
133.6209
128.7904
128.7041
77.4246
77.0004
76.5768
57.4939
35.5414
19.4760
13.4813
O
Me
Cl
H
5o
f1 (ppm)

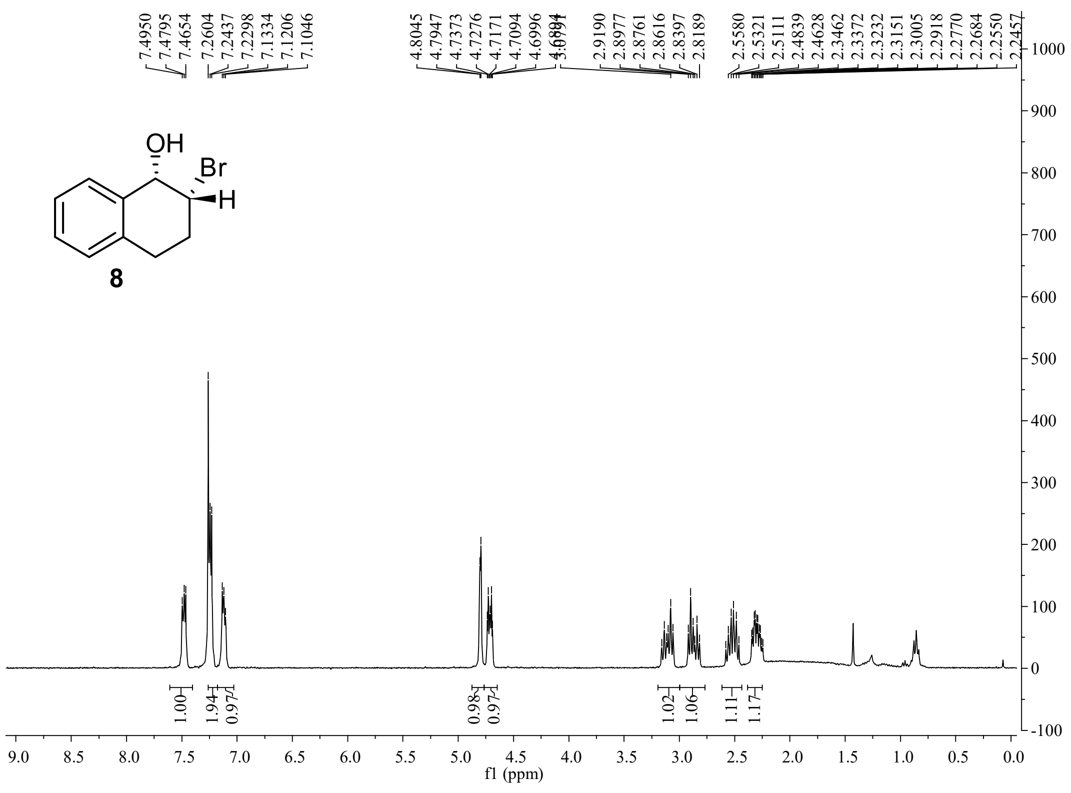

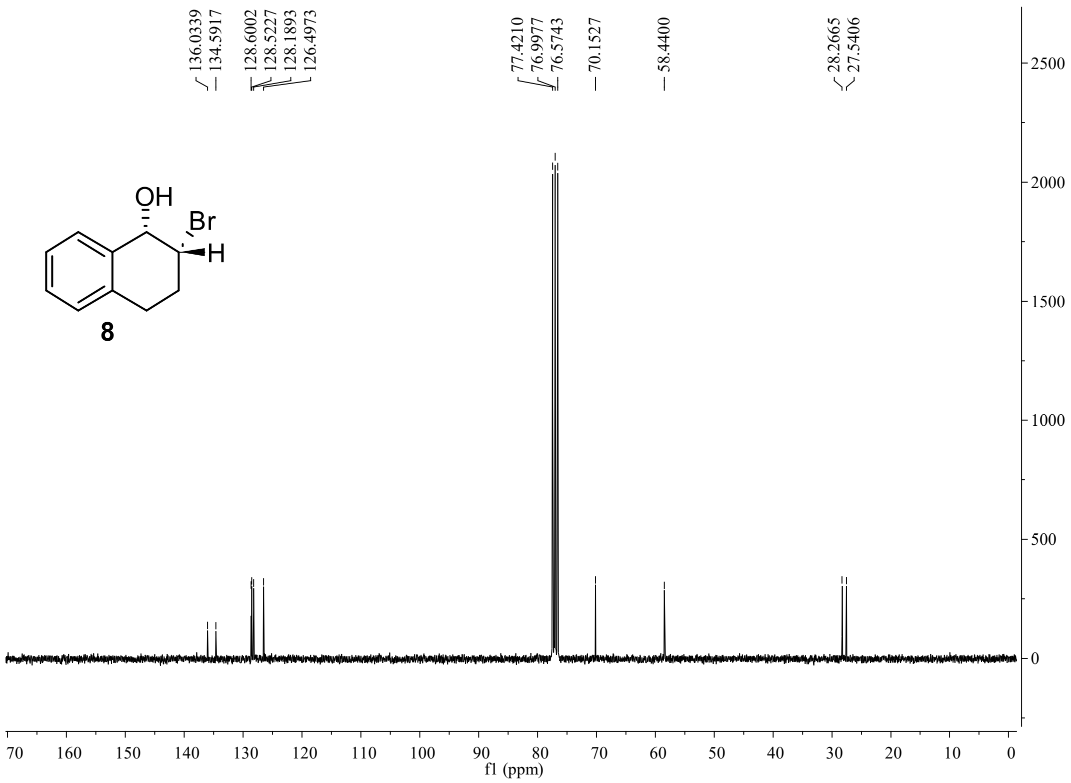

Supplement: Supplementary file 1 [file SC-010-C9SC02000D-s001.pdf]
